# Iridium-catalyzed reductive Ugi-type reactions of tertiary amides

**DOI:** 10.1038/s41467-018-05192-7

**Published:** 2018-07-19

**Authors:** Lan-Gui Xie, Darren J. Dixon

**Affiliations:** Department of Chemistry, Chemistry Research Laboratory, University of Oxford, Mansfield Road, Oxford, OX1 3TA UK

## Abstract

Amides are ubiquitous in the fine chemical, agrochemical and pharmaceutical industries, but are rarely exploited as substrates for homologous amine synthesis. By virtue of their high chemical stability, they are essentially inert to all but the harshest of chemical reagents and to the majority of chemical transformations routinely used in organic synthesis. Accordingly, the development of chemoselective carbon−carbon bond-forming methodologies arising from the functionalization of the amide functionality should find widespread use across academia and industry. We herein present our findings on a series of Ugi-type reactions of tertiary amides enabled by an initial chemoselective iridium-catalyzed partial reduction, followed by reaction with isocyanide and (thio)acetic acid or trimethylsilyl azide, thus providing a multicomponent synthesis of α-amino (thio)amide or α-amino tetrazole derivatives. The reductive Ugi-type reactions are amenable to a broad range of amides and isocyanides, and are applicable to late-stage functionalization of various bioactive molecules and pharmaceutical compounds.

## Abstract (web summary)

Chemical transformation of amides is normally occurring under harsh conditions. Here, the authors report a mild iridium-catalyzed reductive Ugi-type coupling of tertiary amides, isocyanides and (thio)acetic acid or trimethylsilyl azide to give homologous, bioactive amine products.

## Introduction

**Fig. 1:**
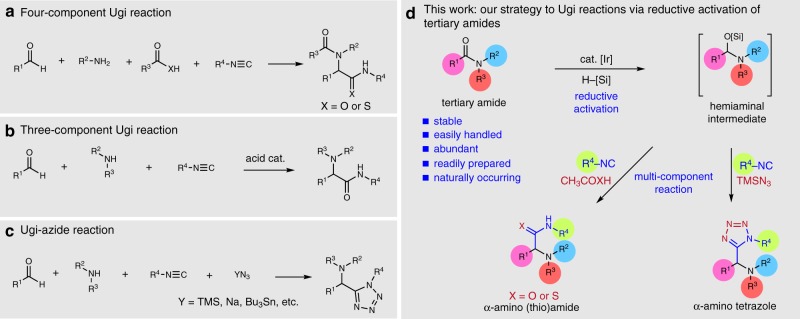
Proposed reductive Ugi-type reactions of tertiary amides. **a** Classical four-, **b** three-component Ugi reactions, and **c** Ugi-azide reaction, **d** our proposed reductive Ugi-type reactions of tertiary amides

Multicomponent reactions (MCRs) have long been among the most attractive reactions in organic and medicinal chemistry, given their power of rapidly generating structural and chemical diversity^1,2^. Ugi reactions (Fig. 1a–c), which command a privileged place in the development of modern MCRs^3–6^, classically include three-component (3-MCRs) and four-component (4-MCRs) types according to the number of distinct reactant classes involved^7^. These reactions require reactive aldehydes and amines (mainly primary)^8,9^ to form the key imine/iminium intermediates, which are subsequently trapped by isocyanide nucleophiles, and afford either α-amino thioamides or α-amino amides depending on the nature of the carboxylic (thio)acid used to promote the reaction^10,11^. In a related variant, the combination of aldehyde, amine, and trimethylsilyl azide (TMSN_3_) can produce efficiently α-amino tetrazole derivatives. This MCR—generally referred to as the Ugi-azide reaction—has become established as one of the most reliable and versatile strategies to access such compounds^12^. Tetrazoles are an important class of compound commonly used within drug discovery programs^13,14^, as bioisosteres for the carboxylate group and, for example, as blockers for the Angiotensin II receptor^15^. Furthermore, various tetrazole-containing molecules have found use in organocatalysis^16^ and organometallic chemistry^17^, as well as in material science^18^.

Tertiary amides are an under-exploited class of nitrogen-containing compound that is widespread throughout the chemical, pharmaceutical and agrochemical industries. They can be readily prepared from commonly occurring and abundant carboxylic acids and amines through well-defined, reliable coupling methods^19,20^, and—due to their high chemical stability and low inherent reactivity—they are essentially inert to all but the harshest of reaction types. Accordingly, new strategies for the chemoselective functionalization of amides, leading to new carbon−carbon bond-forming methodologies, would find tremendous application in synthesis, late-stage functionalization, and rapid generation of molecular diversity^21–42^.

Our group^43–45^ has been working on developing new methodologies for the synthesis of amine-containing products from reactive hemiaminal intermediates, generated by the highly chemoselective, catalytic reduction of tertiary amides under mild conditions^46–50^. Looking to expand the diversity of product types accessible we considered isocyanides^51–53^ as potential nucleophiles. Based on the known interaction between hemiaminals^10^ and isocyanides and our own prior experience with both partners^43–45,54–56^, we hypothesized that isocyanides might, in the presence of an acid promoter, intercept the in situ formed iminium species, thus generating versatile nitrilium intermediates applicable for various Ugi-type reactions (Fig. 1d). In related context, Zheng and Huang have reported that secondary amides undergo Ugi-amide formation when activated with triflic anhydride^30^.

Herein we report our findings leading to an iridium-catalyzed reductive Ugi-type MCR of tertiary amides, isocyanide and (thio)acetic acid or trimethylsilyl azide (TMSN_3_), affording α-amino (thio)amide and α-amino tetrazole structures respectively.

## Results

### Optimization study

As a proof-of-concept study, 4-methylbenzoxyl amide (1.0 eq) was subjected to standard amide partial reduction condition using IrCl(CO)(PPh_3_)_2_ (Vaska’s complex, 1 mol%) and tetramethyldisiloxane (TMDS, 2.0 eq) in dichloromethane at room temperature for 20 min, followed by the addition of *tert*-butyl isocyanide (2.0 eq) and formic acid (1.0 eq). We were pleased to detect the desired α-amino amide product **2** in significant amounts (Table 1, entry 1). The reaction was also successful using acetic acid (1.0 eq) in place of formic acid; ^1^H NMR analysis indicated a 75% yield of the product (Table 1, entry 2). Further experiments demonstrated a slight excess of acetic acid resulted in the formation of **2** in 78% isolated yield (Table 1, entries 3−5). Reaction concentration (Table 1, entries 6, 7), solvents (Table 1, entries 8, 9) and acids (Table 1, entries 10−13) were also screened, but the optimal conditions were those as described in entry 4.

**Table 1:** Discovery and optimization


Entry	Solvent (M)	Acid (eq)	Yield (%)^a^
1	CH_2_Cl_2_ (0.1)	HCOOH (1.0)	35
2	CH_2_Cl_2_ (0.1)	CH_3_COOH (1.0)	75
3	CH_2_Cl_2_ (0.1)	CH_3_COOH (0.2)	31
4	CH_2_Cl_2_ (0.1)	CH_3_COOH (1.2)	82 (78^b^)
5	CH_2_Cl_2_ (0.1)	CH_3_COOH (2.0)	78
6	CH_2_Cl_2_ (0.2)	CH_3_COOH (1.2)	80
7	CH_2_Cl_2_ (0.05)	CH_3_COOH (1.2)	20
8	DCE (0.1)	CH_3_COOH (1.2)	76
9	toluene (0.1)	CH_3_COOH (1.2)	78
10	CH_2_Cl_2_ (0.1)	diphenyl phosphate (1.2)	<10
11	CH_2_Cl_2_ (0.1)	camphorsulphonic acid (1.2)	trace
12	CH_2_Cl_2_ (0.1)	4-nitrophenol (1.2)	N.O.
13	CH_2_Cl_2_ (0.1)	ZnCl_2_ (1.2)	N.O.

Reactions were performed on 0.3 mmol of amide

N.O. not observed

^a^NMR yields with 1-bromo-2-methoxylnaphthlene as internal standard

^b^Isolated yield. DCE: 1,2-dichloroethane

### Substrate scope

**Fig. 2:**
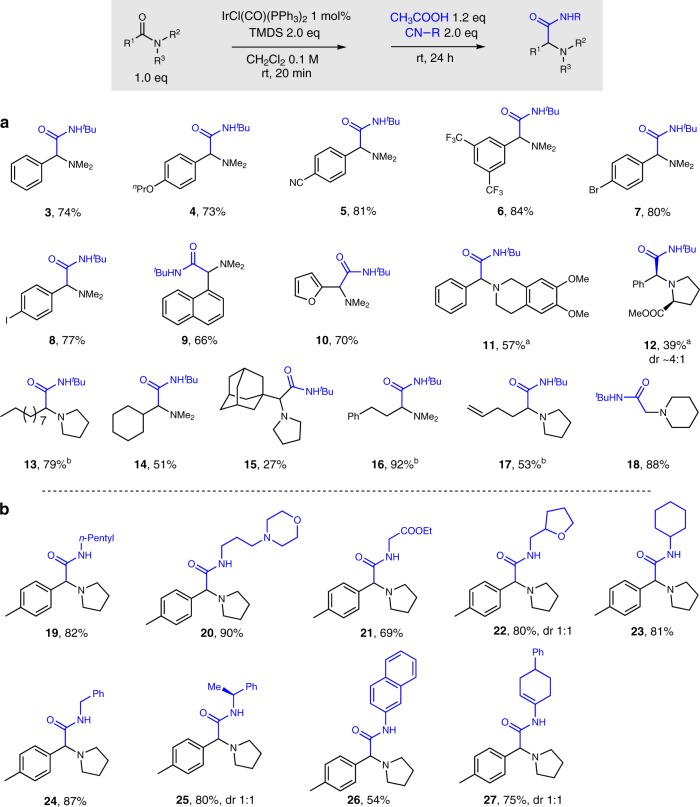
Synthesis of α-amino amides. **a** Scope with respect to the tertiary amide. **b** Scope with respect to the isocyanide. Standard condition: amide 0.3 mmol, IrCl(CO)(PPh_3_)_2_ 1 mol%, TMDS 0.6 mmol, CH_2_Cl_2_ 3 mL, isocyanide 0.6 mmol, CH_3_COOH 0.36 mmol; yields of purified product following flash column chromatography; ^a^reaction was performed at 50 °C after the addition of *tert*-butyl isocyanide and CH_3_COOH; ^b^with 0.9 mmol CH_3_COOH

With optimal conditions in hand, we then explored the scope of this reductive Ugi reaction by subjecting a broad range of carboxylic amides to this protocol. As shown in Fig. 2a, aryl amides with both electron-donating and electron-withdrawing groups on the phenyl ring were good substrates affording the corresponding amino amides (**3**−**8**) in good yields. The toleration of aryl bromide and aryl iodide residues indicates the possibility of combining this protocol with standard metal-catalyzed cross-coupling chemistry. Naphthalene and furan-derived amides underwent the transformation smoothly (**9**, **10**). Amides derived from dimethoxy-1,2,3,4-tetrahydroisoquinoline and proline also proved amenable and allowed the synthesis of amino amide (**11**, **12**) albeit using a slightly increased temperature (50 °C). Linear alkyl, α-branched and fully substituted alkyl amides were all viable partners, delivering the corresponding products (**13**−**15**). Diminished yields were observed when the substrates were sterically hindered. Pendant phenyl and vinyl groups in the carboxylic acid residue were also tolerated and delivered products (**16**, **17**). Moreover, the use of a formic acid-derived amide led to the unsubstituted α-amino amide in excellent yield (**18**). Unfortunately, attempts to apply lactam substrates, such as 1-butylazepan-2-one and 1-benzylpiperidin-2-one, to this reductive Ugi-amide formation were unsuccessful.

Subsequently, the scope of the reaction with respect to the isocyanide component was examined under the standard reaction conditions (Fig. 2b). Linear alkyl isocyanides bearing morpholino, ester, and tetrahydrofuranyl functionality were excellent partners allowing the ready assembly of amino amides (**19**−**22**). Branched cyclohexyl and benzyl isocyanide afforded good yields of the respective MCR products (**23**, **24**). Good reactivity, albeit with no diastereoselectivity, was found when enantiopure α-methylbenzyl isocyanide was applied (**25**). Furthermore, both aryl and vinyl amides were obtained in good yields (**26**, **27**) when the corresponding isocyanides were submitted to the standard reaction conditions.

**Fig. 3:**
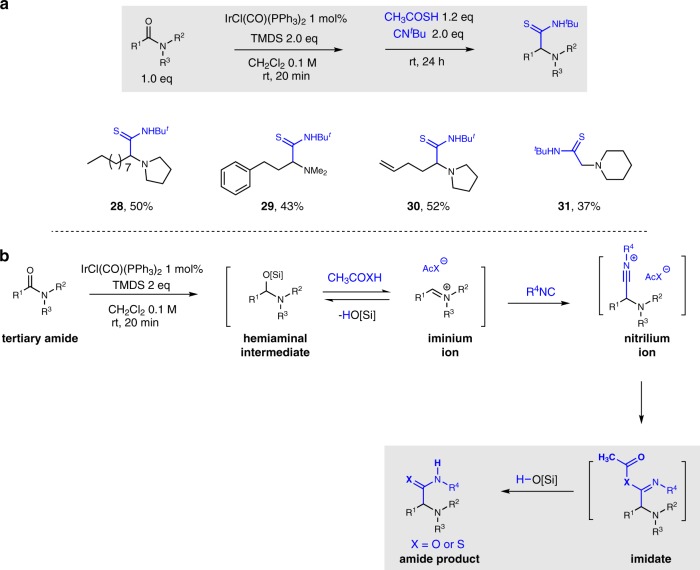
Synthesis of α-amino thioamides and mechanism of the reductive Ugi reactions. **a** Synthesis of α-amino thioamide derivatives from tertiary amides. Standard condition: amide 0.3 mmol, IrCl(CO)(PPh_3_)_2_ 1 mol%, TMDS 0.6 mmol, CH_2_Cl_2_ 3 mL, *tert*-butyl isocyanide 0.6 mmol, CH_3_COSH 0.36 mmol; yields of purified product following flash column chromatography. **b** Proposed mechanism of the reductive Ugi reactions

As is classically known for Ugi-type reactions, use of thioacetic acid as a substitute for acetic acid should result in the generation of thioamide functionality in the reaction product^8^. To our delight, the corresponding thioamides were indeed isolated with reasonable yields when a range of tertiary amide substrates were submitted to the standard condition, but with 1.2 eq CH_3_COSH as the acid to promote the Ugi reaction (**28**−**31**) (Fig. 3a). These results indicate further synthetic applications of this reductive Ugi protocol.

Regarding the reaction mechanism for the formation of α-amino (thio)amides and the role of (thio)acetic acid throughout the transformation, a plausible reaction pathway is proposed in Fig. 3b. The silylated hemiaminal intermediate is initially generated by the standard reductive condition from the amide starting material. Then by adding the isocyanide and (thio)acetic acid, the hemiaminal is transformed into the highly reactive nitrilium intermediate^10^, either directly or via the corresponding iminium ion. Nucleophilic addition by acetate or thioacetate then affords the (thio)imidate intermediate, which after rearrangement, leads to the observed α-amino amide or thioamide product^8^.

These exciting successes inspired us to hypothesize that the formed reactive nitrilium intermediate might also be poised to undergo the Ugi-azide reaction: trapping by an appropriate azide source should facilitate a 1,3-dipolar cyclization, thus delivering α-amino tetrazole as the product^57–60^. This reaction would transform stable, readily available tertiary amides into homologated high value α-amino tetrazole motifs in a single pot. The idea was initially tested by mixing *tert*-butyl isocyanide, acetic acid, and TMSN_3_ with the in situ generated hemiaminal from amide **1** under the standard and mild reaction conditions. Indeed the Ugi-azide reaction proceeded smoothly, leading to the α-amino tetrazole product **32** in 90% yield. Further follow-up experiments demonstrated that without the addition of acetic acid, the tetrazole product was obtained in quantitative yield (98%).

**Fig. 4:**
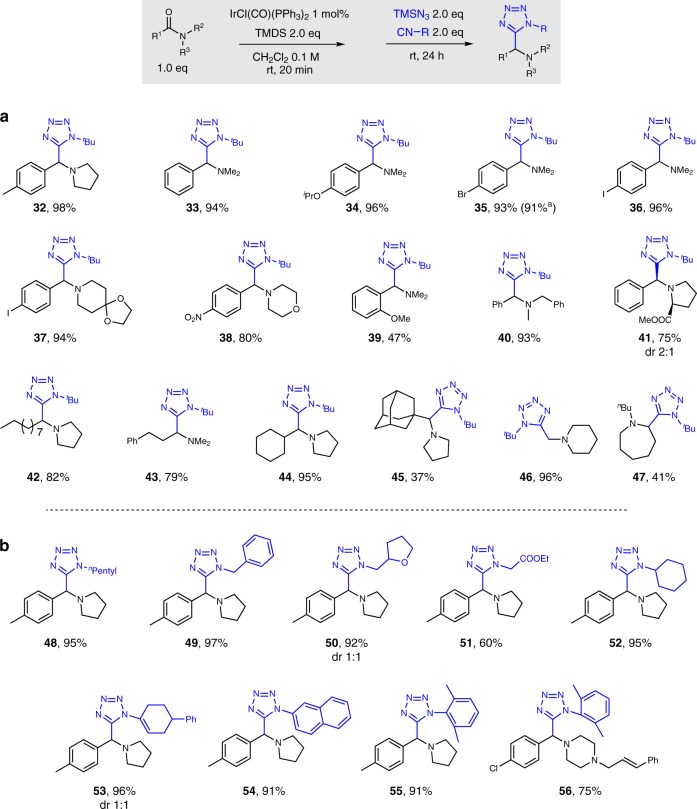
Synthesis of α-amino tetrazoles. **a** Scope with respect to the tertiary amide. **b** Scope with respect to the isocyanide. Standard condition: amide 0.3 mmol, IrCl(CO)(PPh_3_)_2_ 1 mol%, TMDS 0.6 mmol, CH_2_Cl_2_ 3 mL, isocyanide 0.6 mmol, trimethylsilyl azide 0.6 mmol; yields of purified product following flash column chromatography; ^a^reaction was carried out with 1.14 g of amide

Figure 4a depicts the full scope of tertiary amides compatible with this α-amino tetrazole synthesis. Both electron-rich and electron-deficient phenyl derived amides smoothly coupled with azide and isocyanide under this protocol, delivering excellent yields of the desired products (**32**−**38**). Steric hindrance, at the ortho-position of benzoyl amide derivatives, led to a decline in yield (**39**). *N*-Benzyl amine-derived amide proved to be a suitable partner for the amide Ugi-azide reaction (**40**). It is worth noting that *N*-benzoyl methylprolinate was also a good substrate, giving proline-derived tetrazole product in 75% yield (dr 2:1) (**41**). The tetrazole synthesis also tolerated linear and α-branched alkyl amides (**42**−**44**). Tertiary alkyl amide, as exemplified by adamantanyl dimethyl amide, was also amenable to the protocol (**45**), although a lower yield of product was obtained. An excellent yield of α-amino tetrazole product was obtained when a formamide substrate was employed (**46**). Importantly, we found a lactam substrate was also applicable to this reaction, although only a moderate yield of product **47** was obtained. In addition, 91% of the tetrazole product **35** was isolated upon exposing 1.14 g of the corresponding amide to the standard reaction condition, proving that the reaction can be readily carried out on gram scale.

Further studies focused on the scope with respect to the isocyanide component of this reductive Ugi-azide reaction of tertiary amides (Fig. 4b). Primary isocyanides bearing functional groups such as phenyl, tetrahydrofuran, and ester functionality were well-tolerated under the standard conditions (**48**−**51**). Cyclohexyl isocyanide (an example of secondary alkyl system) delivered the tetrazole product with 95% yield (**52**). Olefinic and aryl isocyanides afforded the corresponding products in excellent yields (**53**−**55**); even sterically demanding systems such as the 2,6-dimethylphenyl isocyanide had no deleterious effect on the efficiency of this reaction (**55**). This amide reductive Ugi-azide reaction will likely find various applications for the direct synthesis of pharmaceutical agents, and to this end here we have demonstrated the one-pot preparation of tetrazole compound **56**, an MCH1 receptor antagonist^61^, in 75% yield starting from a stable, readily available amide.

### Synthetic utility

**Fig. 5:**
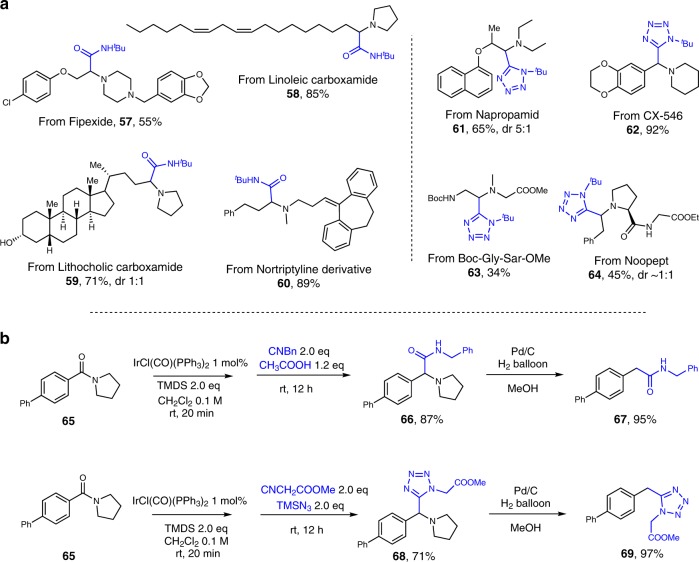
Synthetic application. **a** Late-stage functionalization of bioactive molecules and derivatives using amide reductive Ugi-type reactions. **b** Synthesis of bioactive molecules via reductive Ugi-type reactions followed by hydrogenation. Standard condition: amide 0.3 mmol, IrCl(CO)(PPh_3_)_2_ 1 mol%, TMDS 0.6 mmol, CH_2_Cl_2_ 3 mL, isocyanide 0.6 mmol, CH_3_COOH 0.36 mmol or trimethylsilyl azide 0.6 mmol; yields of purified product following flash column chromatography

Given the prevalence of tertiary amides and their precursors in bioactive molecules, and the surprisingly broad scope tolerated in these amide reductive Ugi-type (thio)amide and tetrazole syntheses, further synthetic potential of this chemistry is demonstrated by the late-stage functionalization of various bioactive molecules and derivatives (Fig. 5a). Fipexide^62^, a psychoactive drug, could be readily coupled with *tert*-butyl isocyanide in the presence of acetic acid (**57**). Linoleic carboxamide, readily prepared from one of the essential fatty acids, linoleic acid^63^, underwent the α-amino amide formation in 85% yield (**58**). Lithocholic acid^64^ (LCA), known for its ability to selectively kill neuroblastoma cells, could be transformed into the corresponding Ugi amide product in a similar fashion (**59**). While the carboxamide derivative of the amine drug nortriptyline^65^, used for treatment of clinical depression, was successfully modified, delivering the desired product in 89% yield (**60**). Submission of the selective systemic amide herbicide, napropamid^66,^ to the amide reductive Ugi-azide reaction resulted in 65% yield of its tetrazole derivative **61**, with 5:1 ratio of diastereoisomers. The reductive coupling of CX-546, a drug candidate for schizophrenia^67^, with *tert*-butyl isocyanide and TMSN_3_ led to the corresponding product in excellent yield (**62**). The success of dipeptide Boc-Gly-Sar-OMe modification under these conditions (**63**) encouraged us to apply this procedure to the peptide drug noopept (omberacetam, a nootropic)^68^. Thus, we were pleased to obtain the tetrazole derivative of noopept in 45% yield (**64**).

Furthermore, by submitting amide **65** (Fig. 5b) to the standard procedure of α-amino amide synthesis, the corresponding amide product was obtained. Removal of pyrrolidine from compound **66** took place nearly quantitatively in the presence of palladium on carbon under an atmosphere of hydrogen, enabling the synthesis of **67** (patented name: IND116065), which exhibits activity in cell viability inhibition^69^. Analogously, regioselective synthesis of 1,5-disubstituted tetrazole compound **69**, previously made as an enzyme inhibitor^70^, could be achieved by carrying out our reductive Ugi-azide reaction, in the presence of corresponding isocyanide and TMSN_3_, followed by the hydrogenation.

In summary, a series of reductive Ugi-type reactions, leading to homologated α-amino (thio)amide and tetrazole motifs, by merging hemiaminal intermediates, isocyanides and (thio)acetic acid or TMSN_3_, has been developed. These reactions benefit from the strategic use of stable and readily available tertiary amides as abundant and under-exploited nitrogen-containing starting materials. The broad scope of tertiary amides and isocyanides that can be tolerated demonstrate the practicality of these multicomponent reactions. Combining this with the illustrated synthetic utility by late-stage functionalization of various bioactive molecules and derivatives, as well as the synthesis of bioactive compounds possessing amide and tetrazole functionality, this powerful α-amino amide and α-amino tetrazole synthesis strategy will likely find numerous applications across academia and industry.

## Methods

### General procedure for the synthesis of α-amino amides and tetrazoles from tertiary amides

Vaska’s catalyst (2.4 mg, 1 mol%) and amide (0.3 mmol, 1.0 eq) were charged into a dry 25 mL flask. Vacuum and N_2_ refilling were repeated for three times. Dry CH_2_Cl_2_ (3 mL, 0.1 M) was injected by syringe, and then TMDS (0.6 mmol, 2.0 eq), while stirring was maintained at room temperature. The resulting mixture was stirred for 20 min, then isocyanide (0.6 mmol, 2.0 eq) and acetic acid (0.36 mmol, 1.2 eq) or TMSN_3_ (0.6 mmol, 2.0 eq) were added sequentially. The solution was stirred overnight at room temperature and was then quenched with saturated aqueous NaHCO_3_ solution and extracted with CH_2_Cl_2_ (3 × 10 mL). The combined organic layers were dried over Na_2_SO_4_ and filtered. The solvent was removed under vacuum. The residue was purified by flash column chromatography.

### Data availability

The authors declare that the data supporting the findings of this study are available within the paper and the Supplementary Information, as well as from the authors upon reasonable request.

## Electronic supplementary material

Supplementary Information
