# Supplementary material for: Iridium-catalyzed reductive Ugi-type reactions of tertiary amides
Source: Nat Commun. 2018 Jul 19;9:2841. doi: 10.1038/s41467-018-05192-7 (PMC6053461; doi:10.1038/s41467-018-05192-7)
Supplement: Supplementary file 1 — Supplementary Information [file 41467_2018_5192_MOESM1_ESM.pdf]

Supplementary Information

Iridium-Catalyzed Reductive Ugi-type Reactions of Tertiary Amides

Lan-Gui Xie and Darren Dixon*

Department of Chemistry, Chemistry Research Laboratory

University of Oxford, Mansfield Road, Oxford OX1 3TA, UK

E-mail: darren.dixon@chem.ox.ac.uk

Supplementary Figures

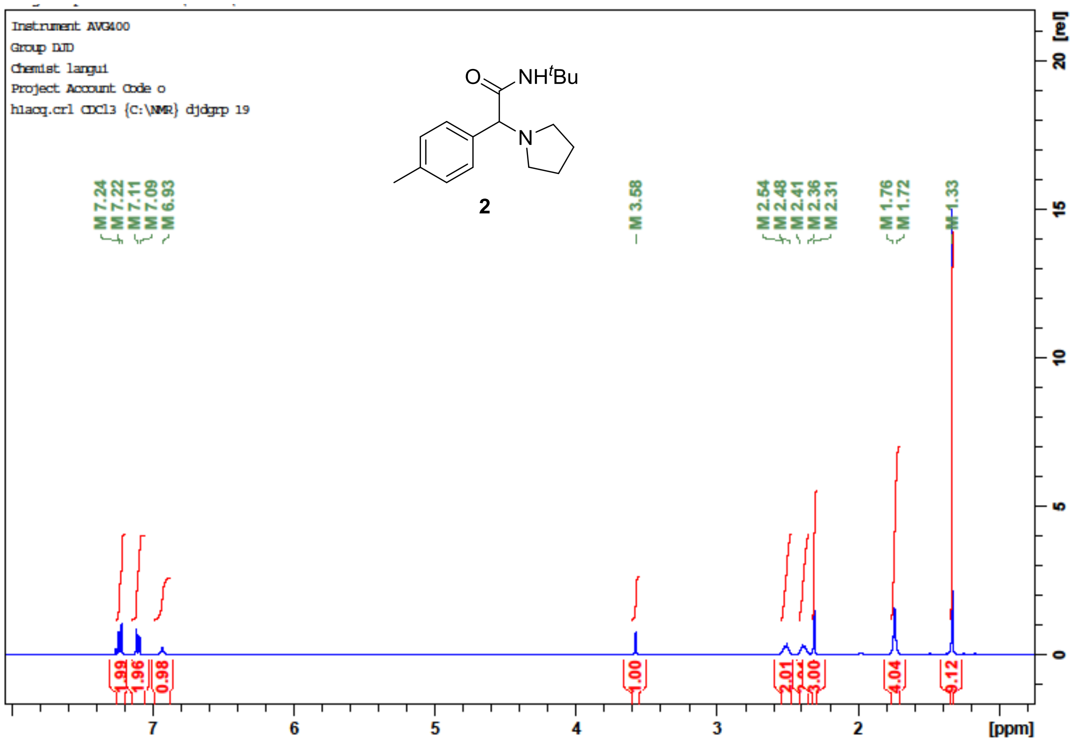

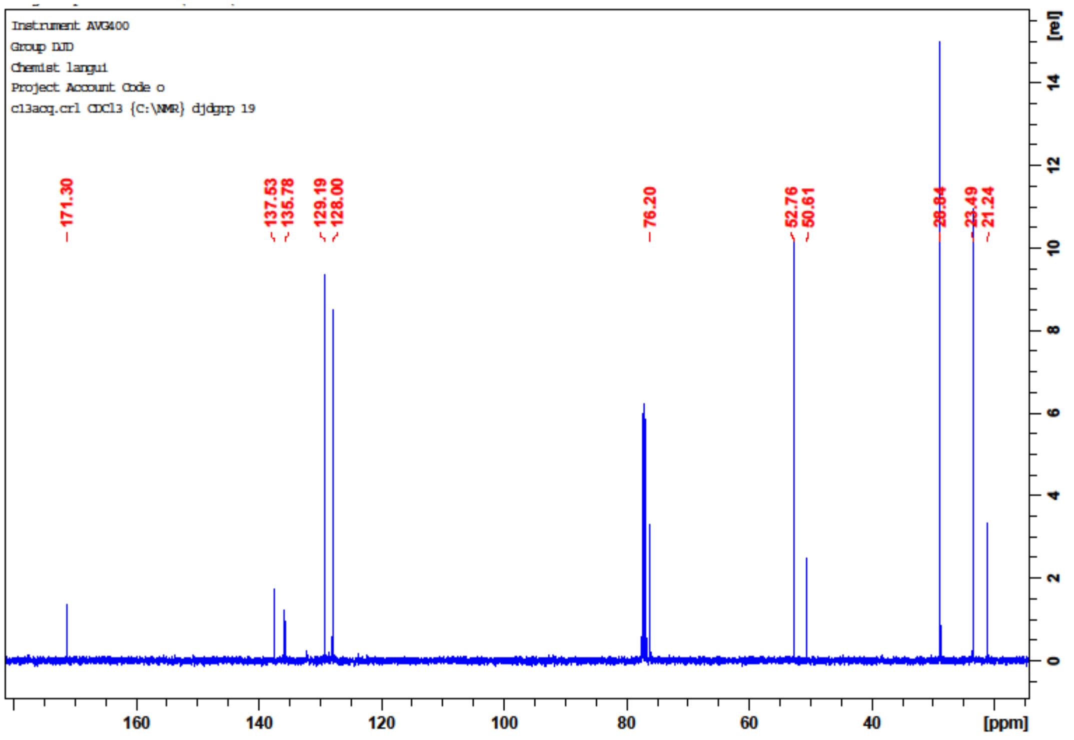

Supplementary Figure 1. ^{1}H NMR and ^{13}C NMR spectra of product **2**.

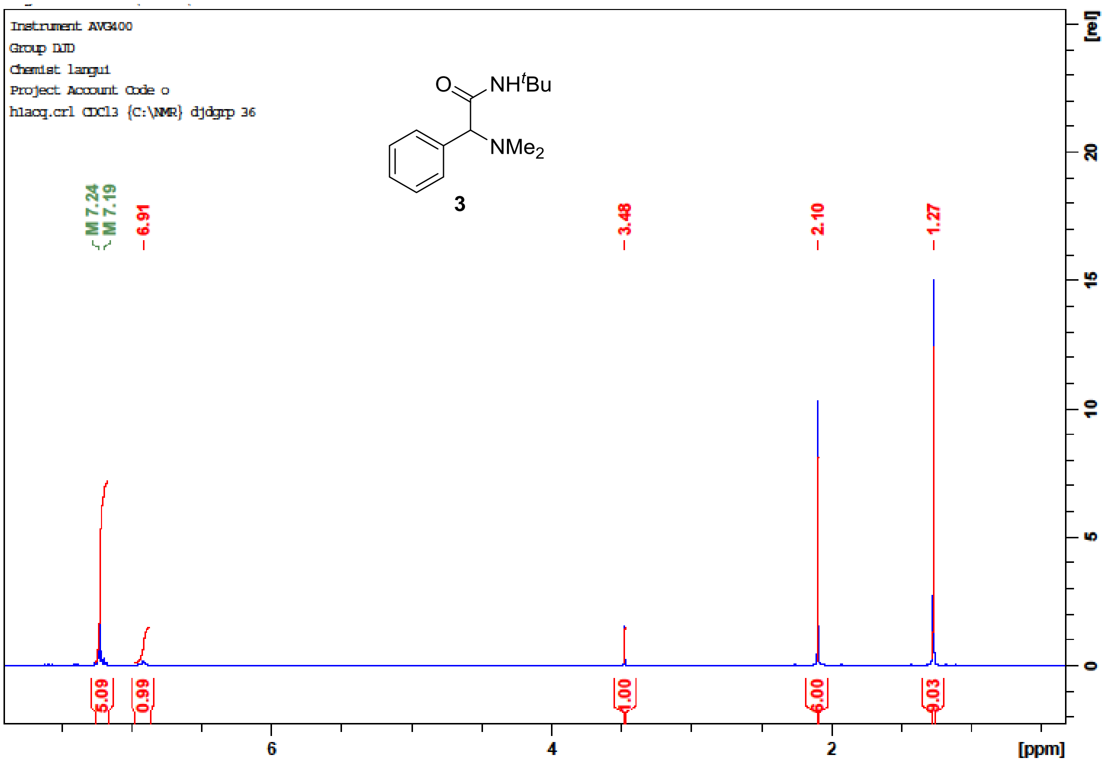

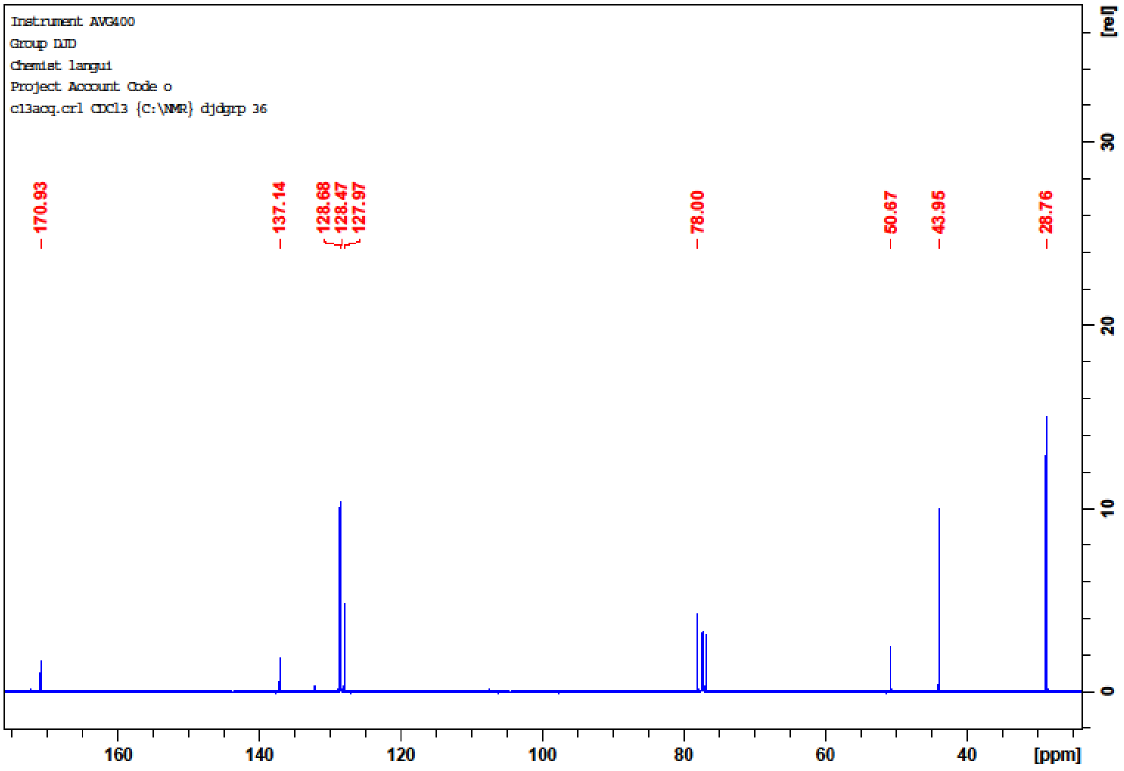

Supplementary Figure 2. ^{1}H NMR and ^{13}C NMR spectra of product **3**.

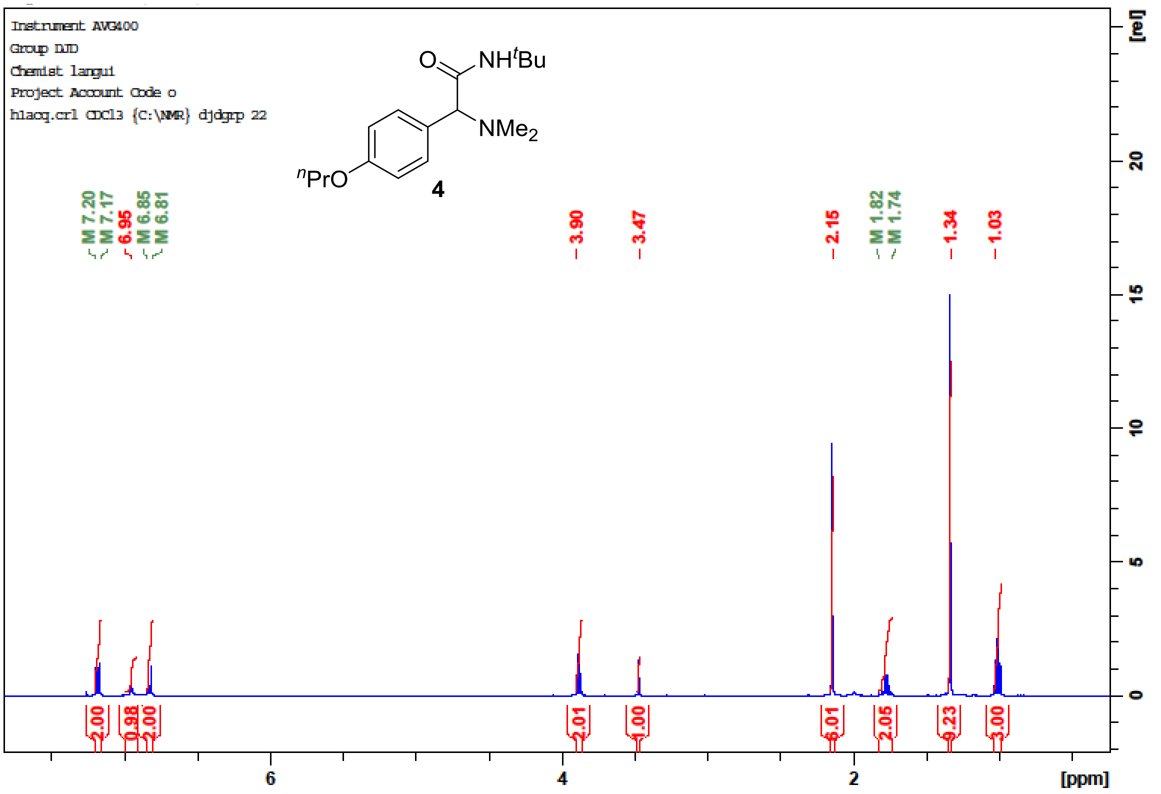

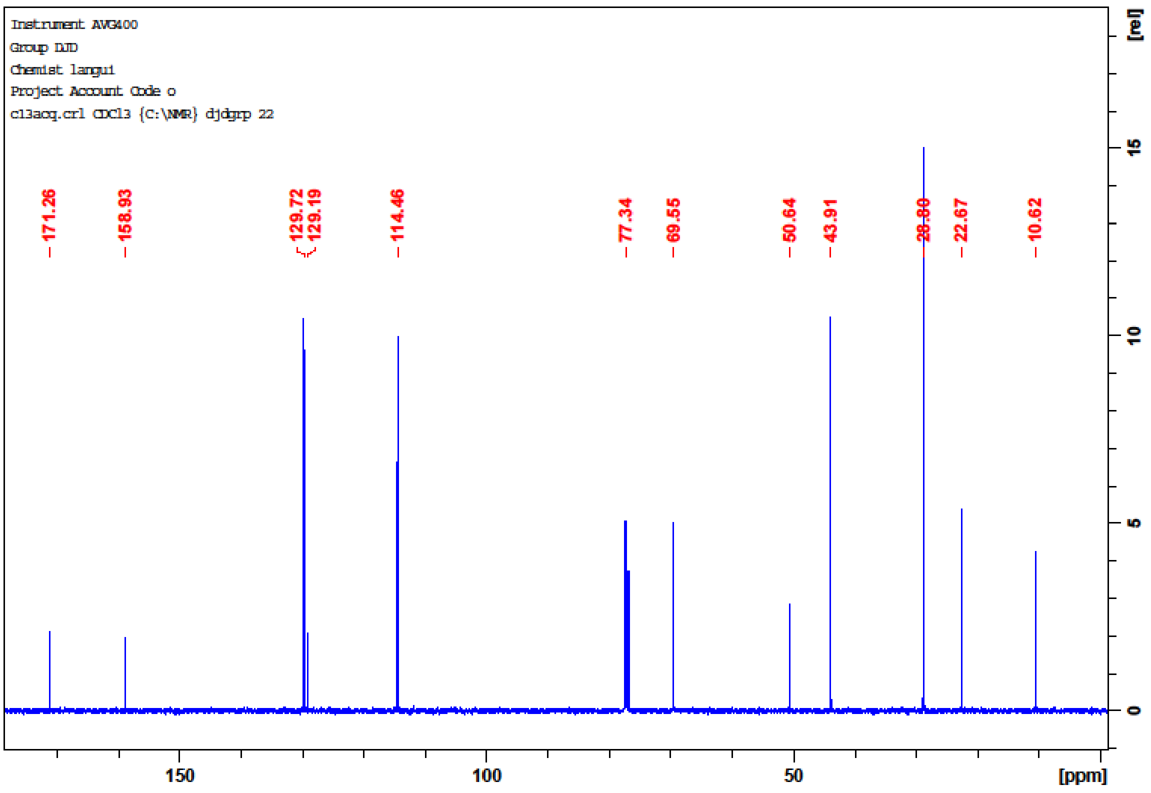

Supplementary Figure 3. ^{1}H NMR and ^{13}C NMR spectra of product **4**.

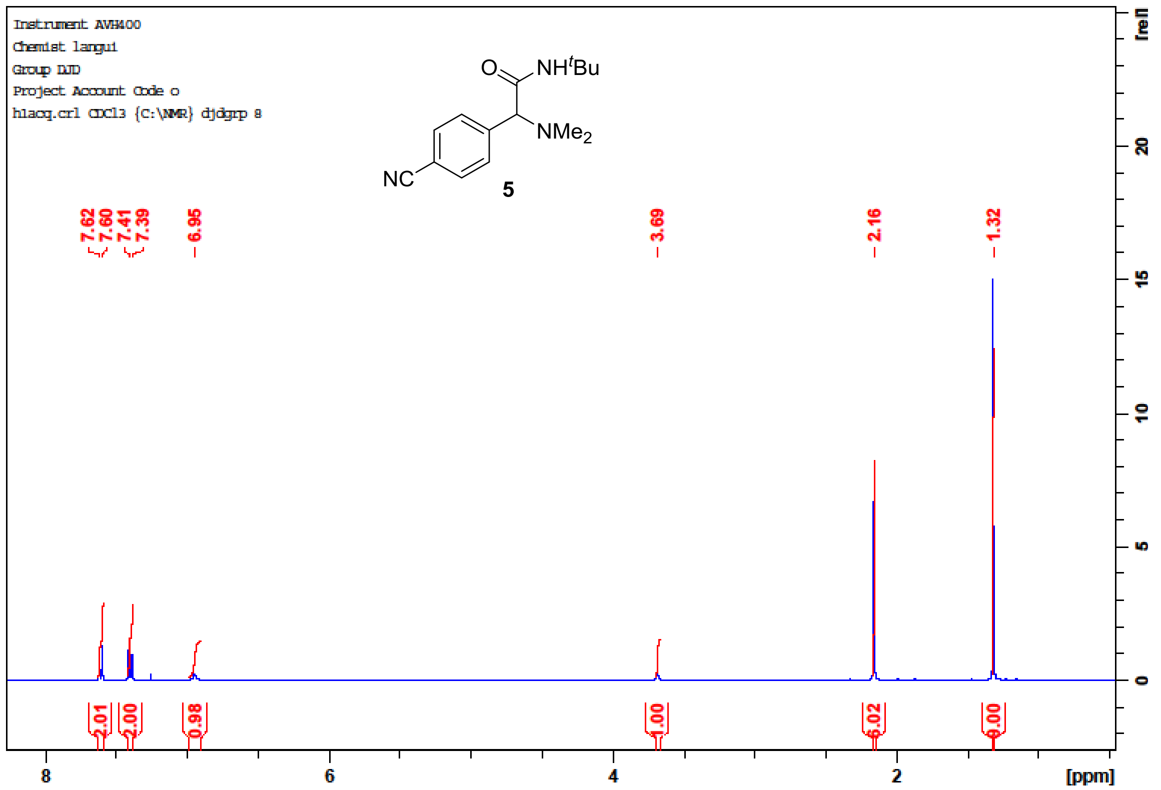

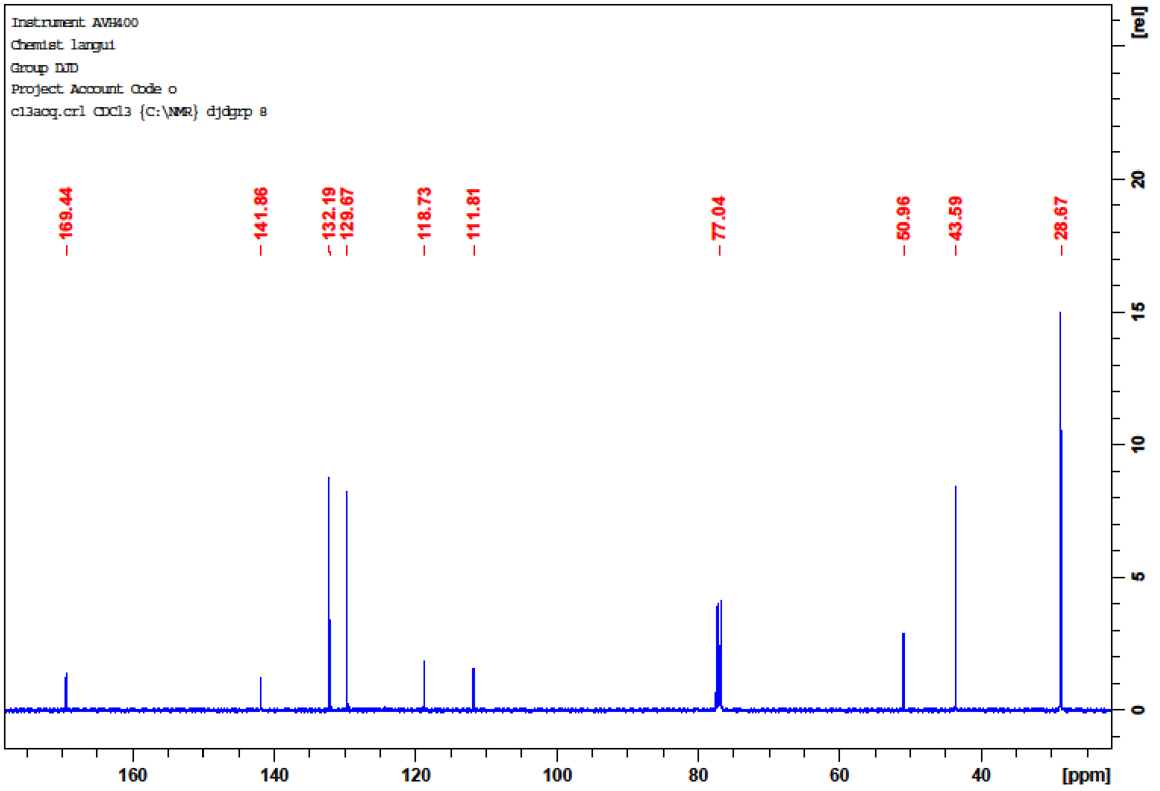

Supplementary Figure 4. ^{1}H NMR and ^{13}C NMR spectra of product **5**.

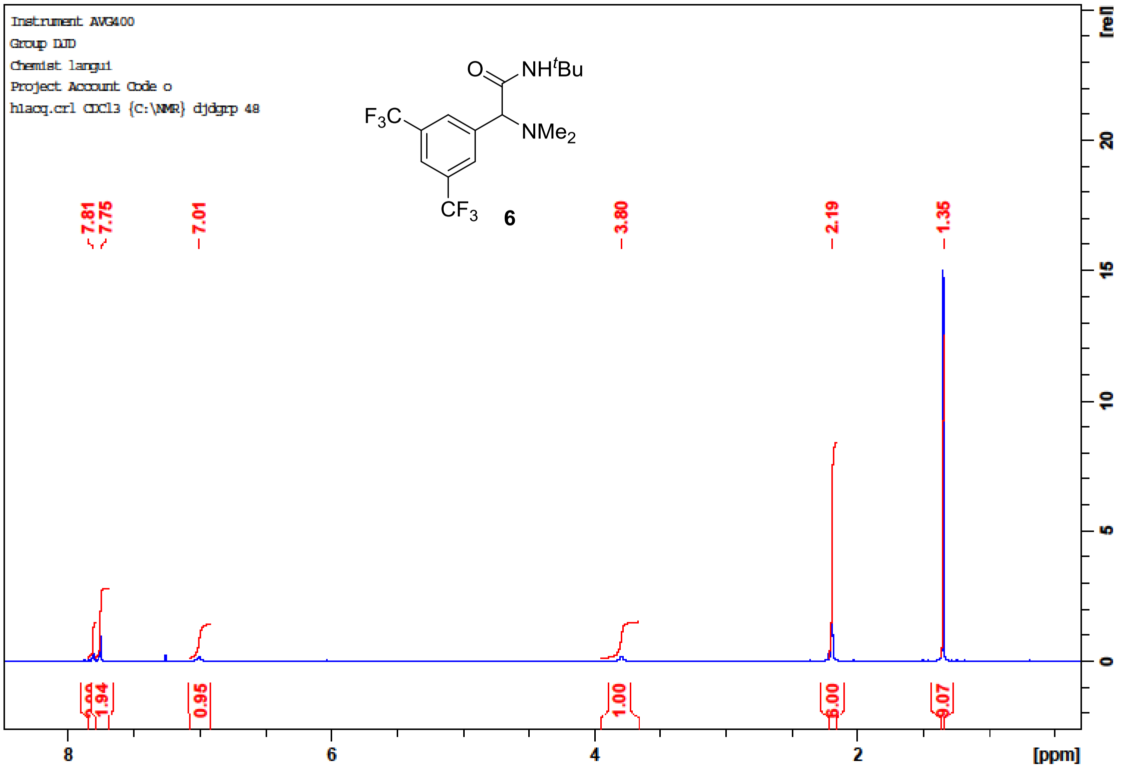

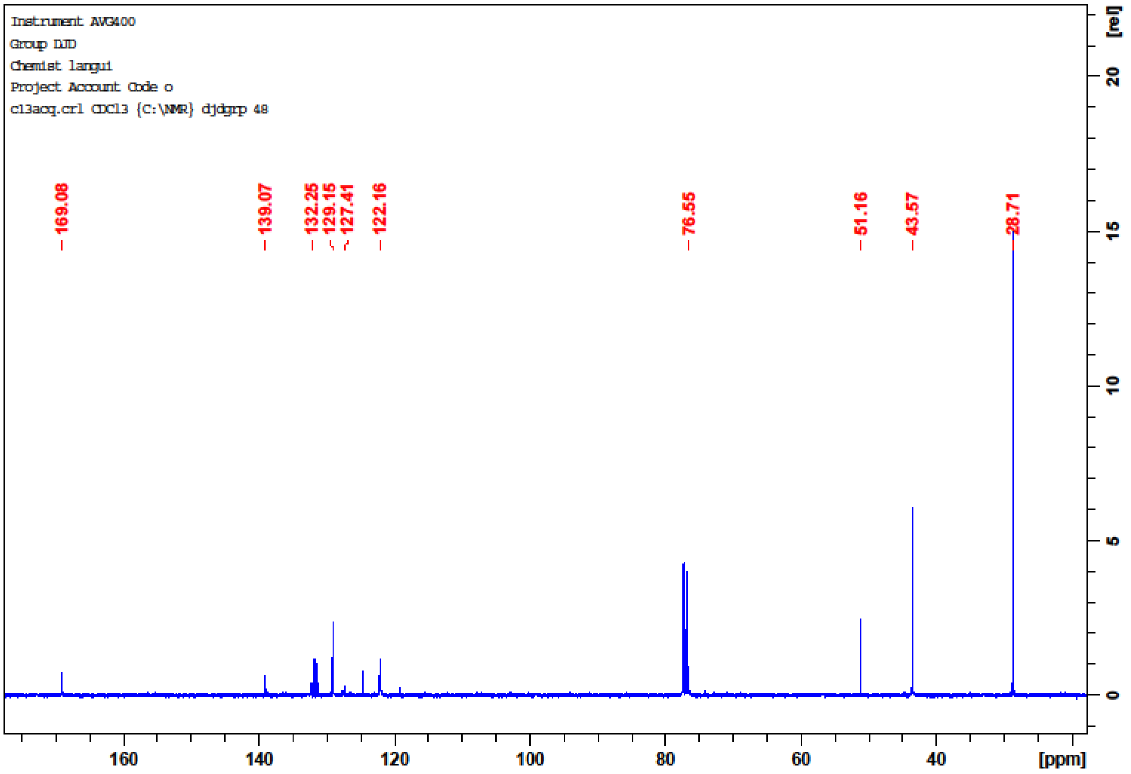

Supplementary Figure 5. ^{1}H NMR and ^{13}C NMR spectra of product **6**.

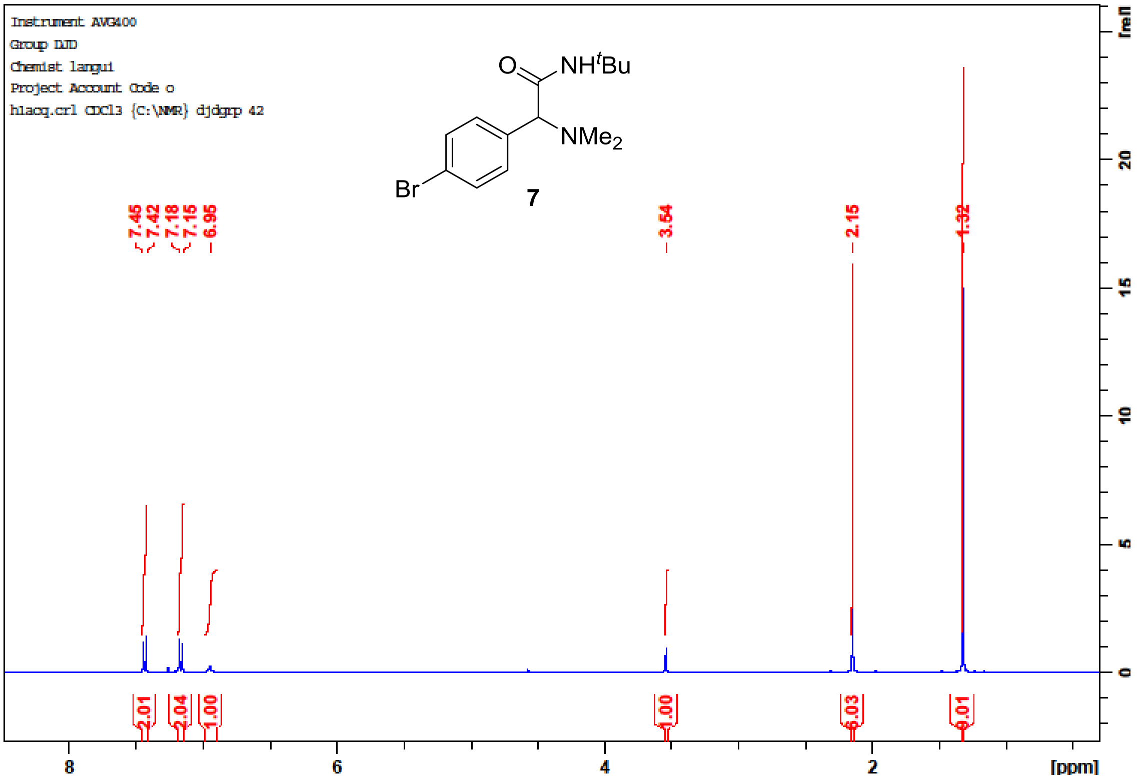

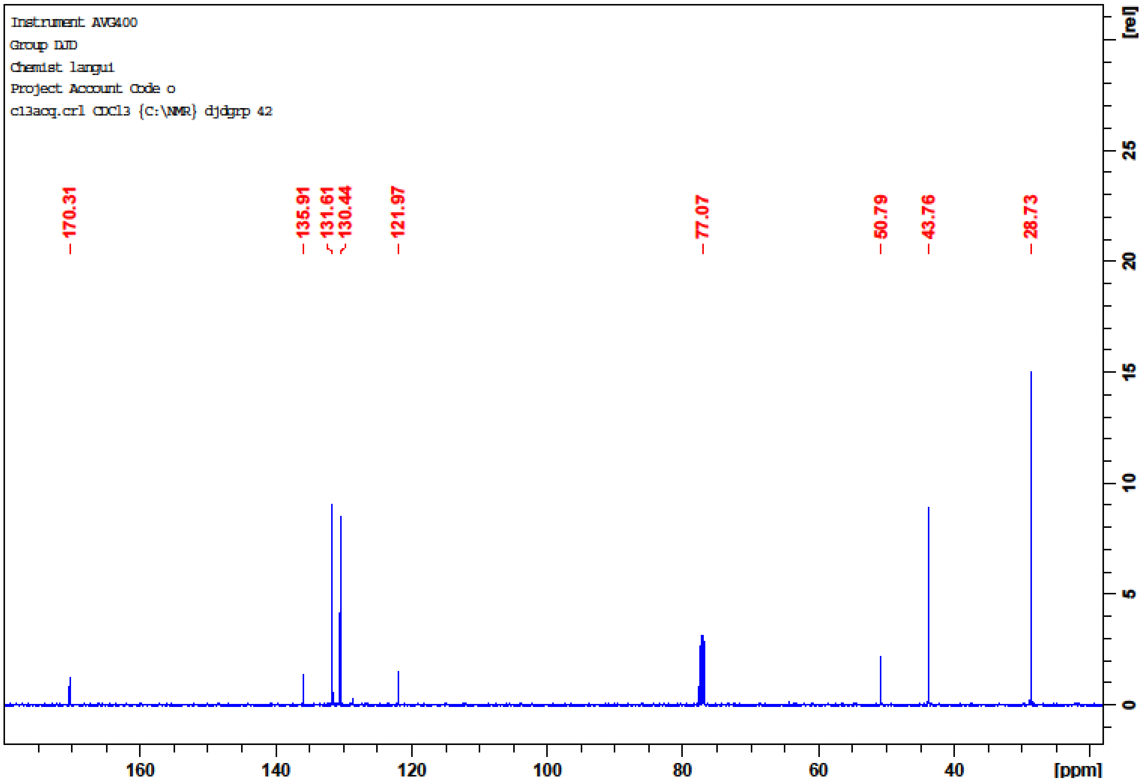

Supplementary Figure 6. ^{1}H NMR and ^{13}C NMR spectra of product **7**.

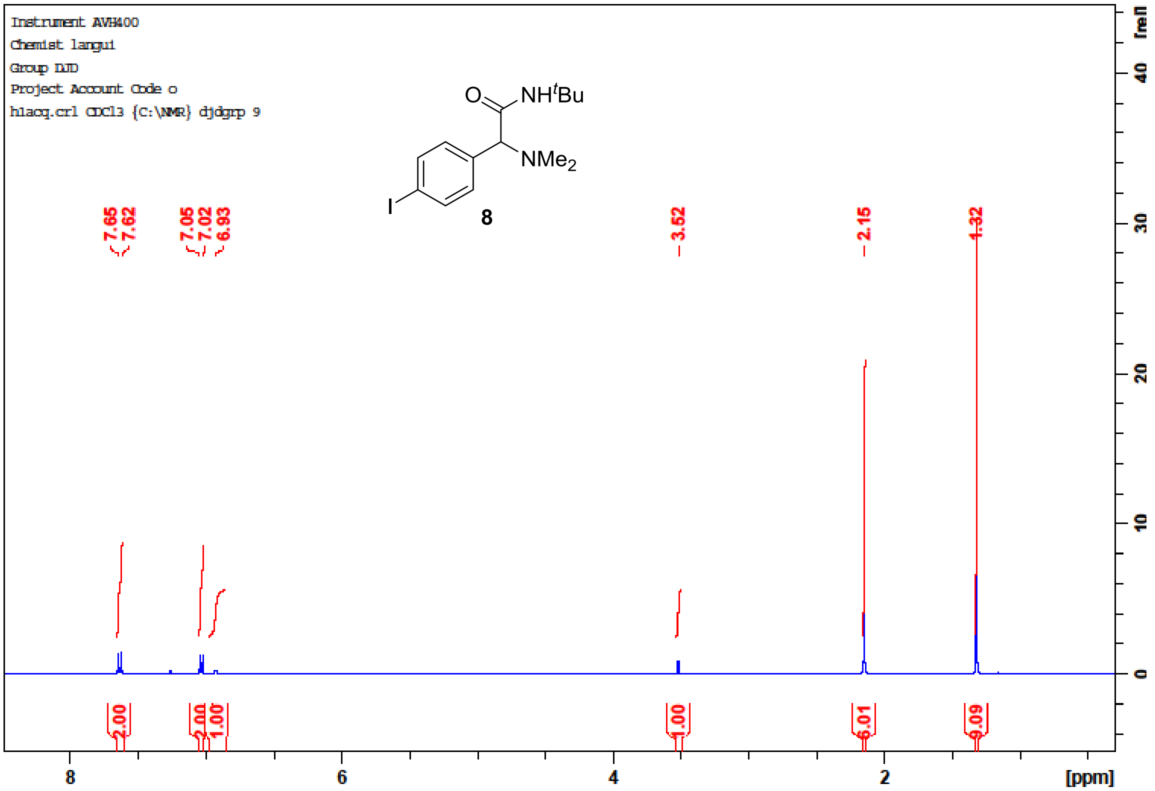

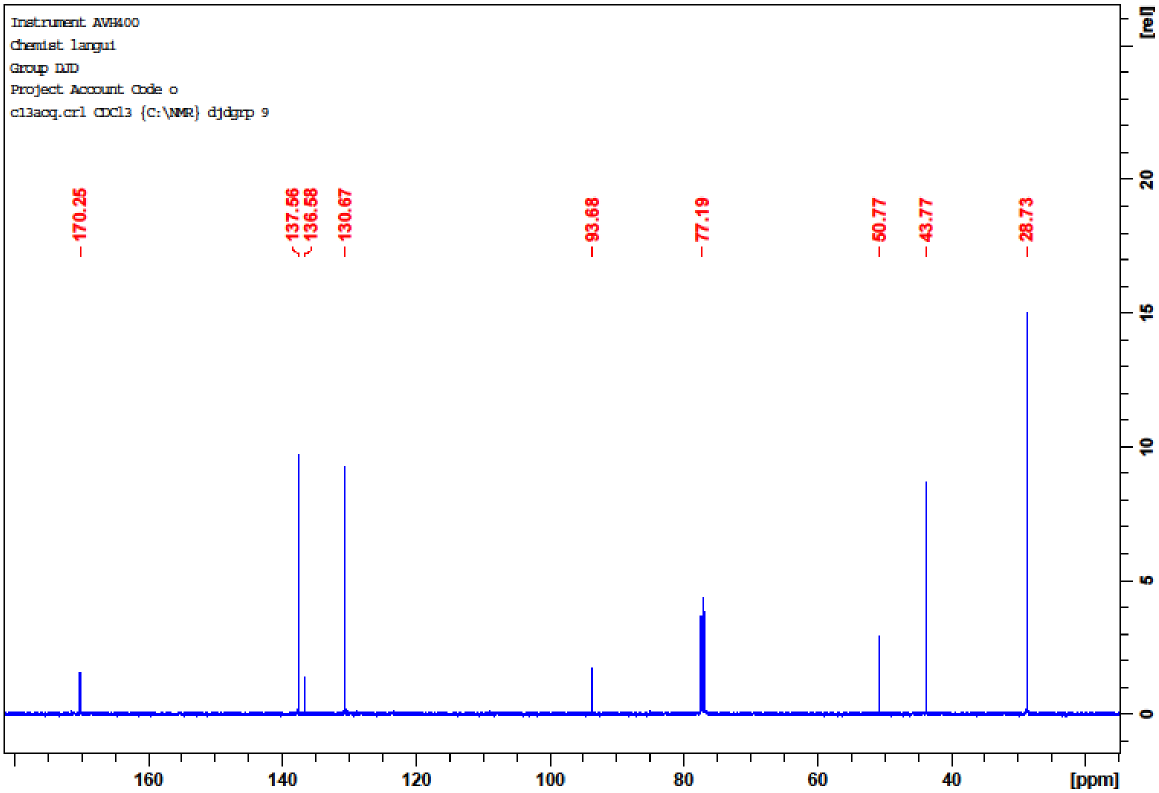

Supplementary Figure 7. ^{1}H NMR and ^{13}C NMR spectra of product **8**.

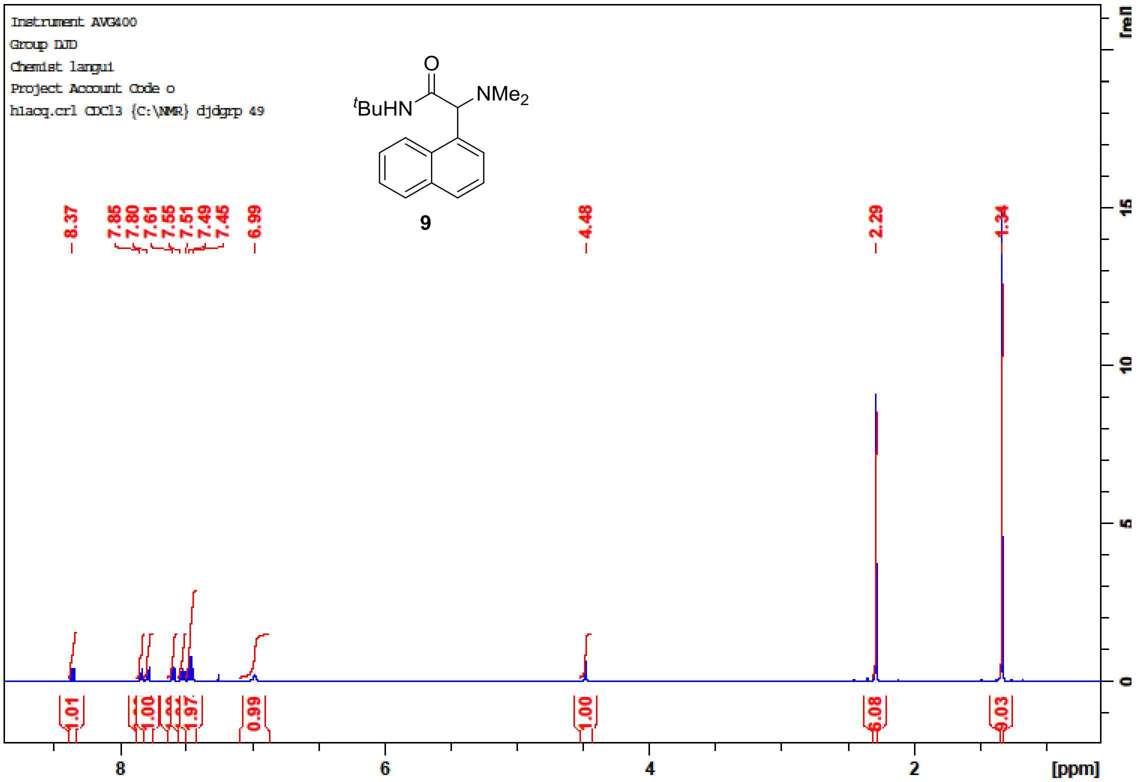

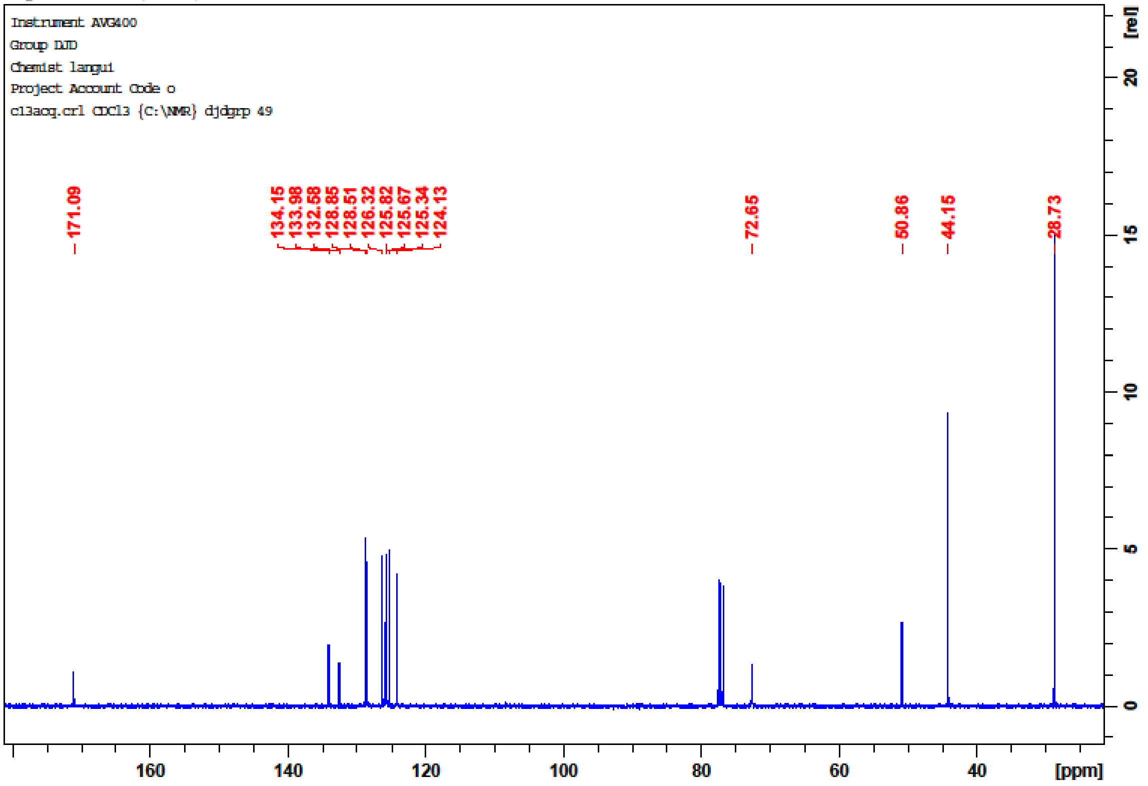

Supplementary Figure 8. ^{1}H NMR and ^{13}C NMR spectra of product **9**.

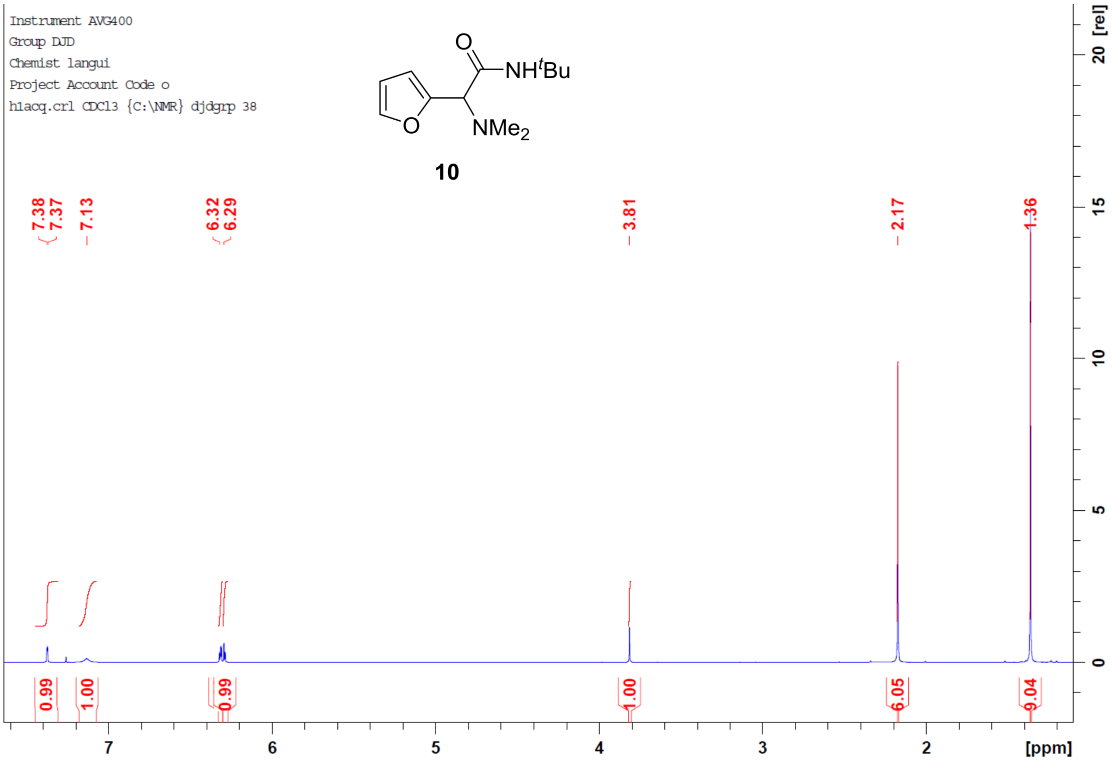

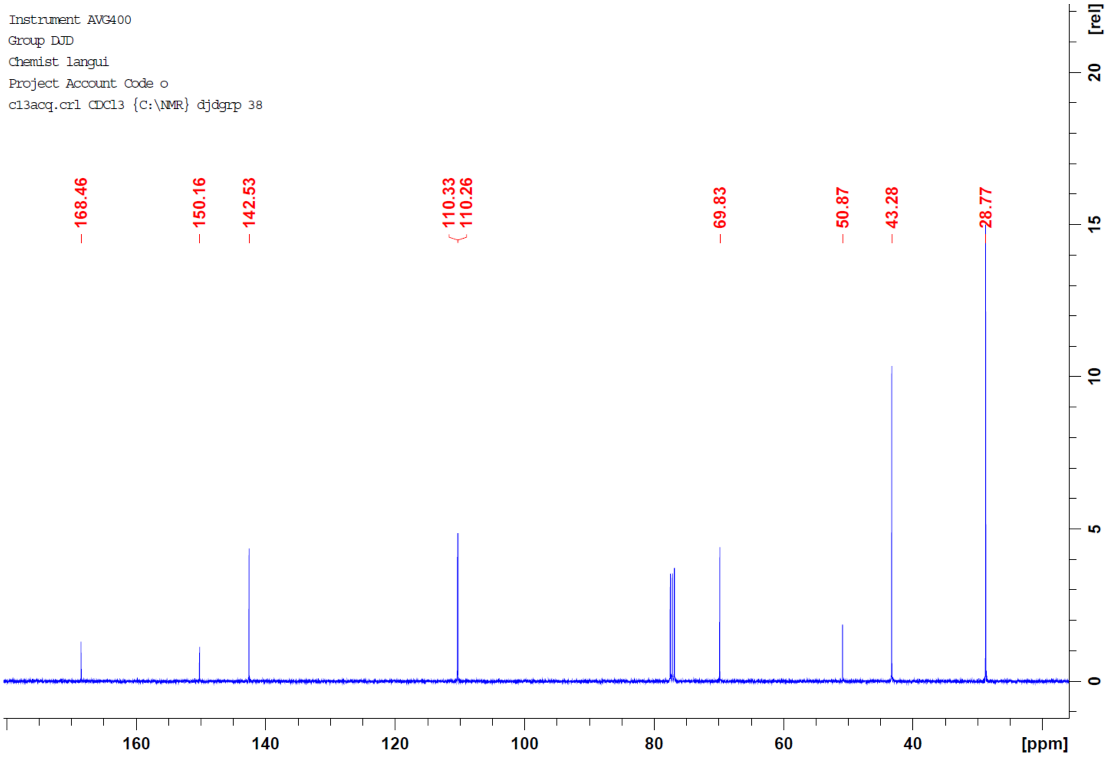

Supplementary Figure 9. ^{1}H NMR and ^{13}C NMR spectra of product **10**.

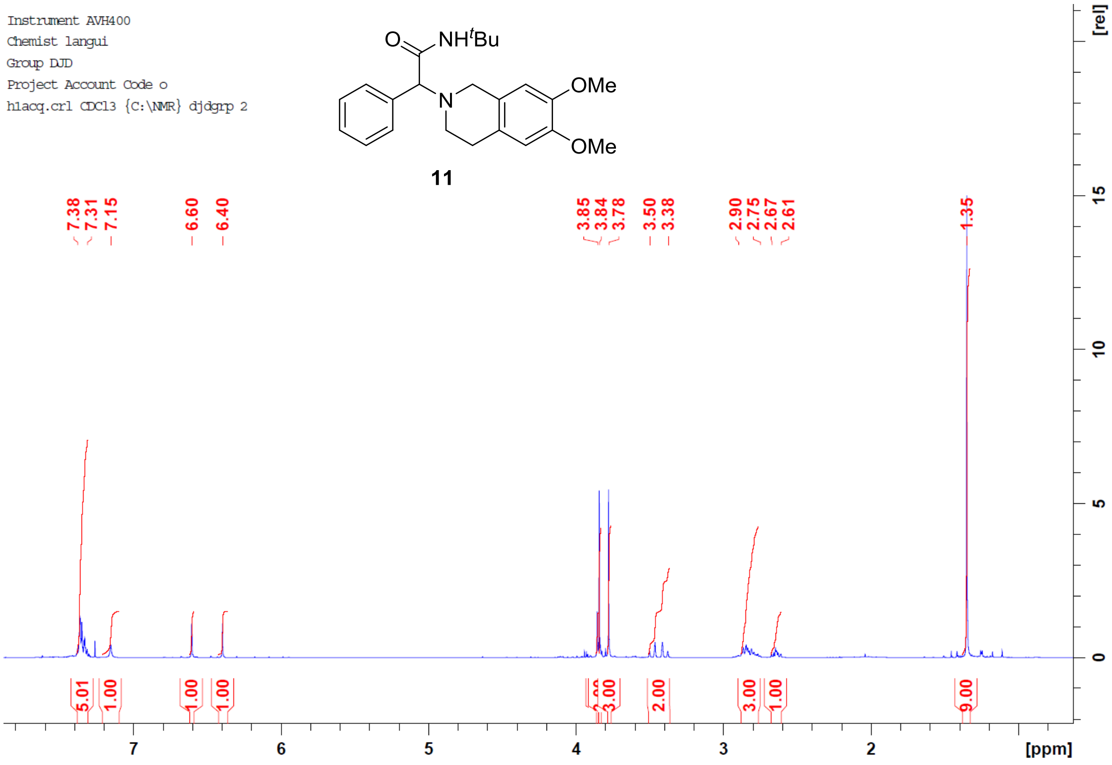

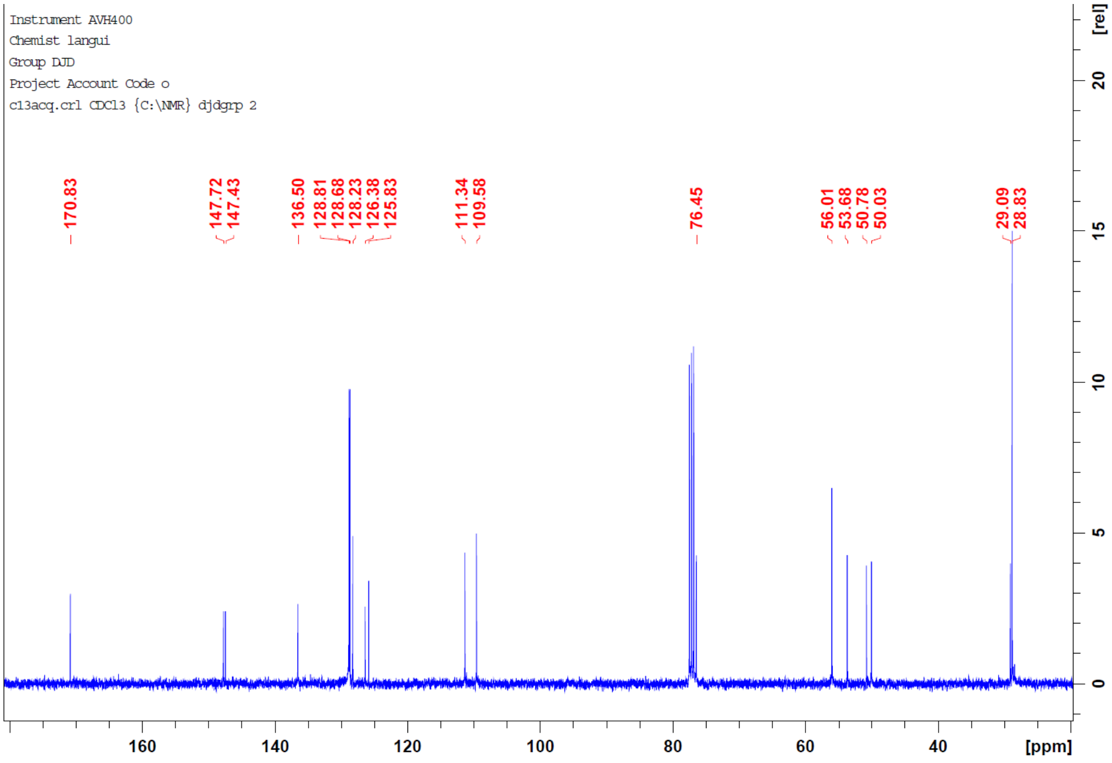

Supplementary Figure 10. ^{1}H NMR and ^{13}C NMR spectra of product **11**.

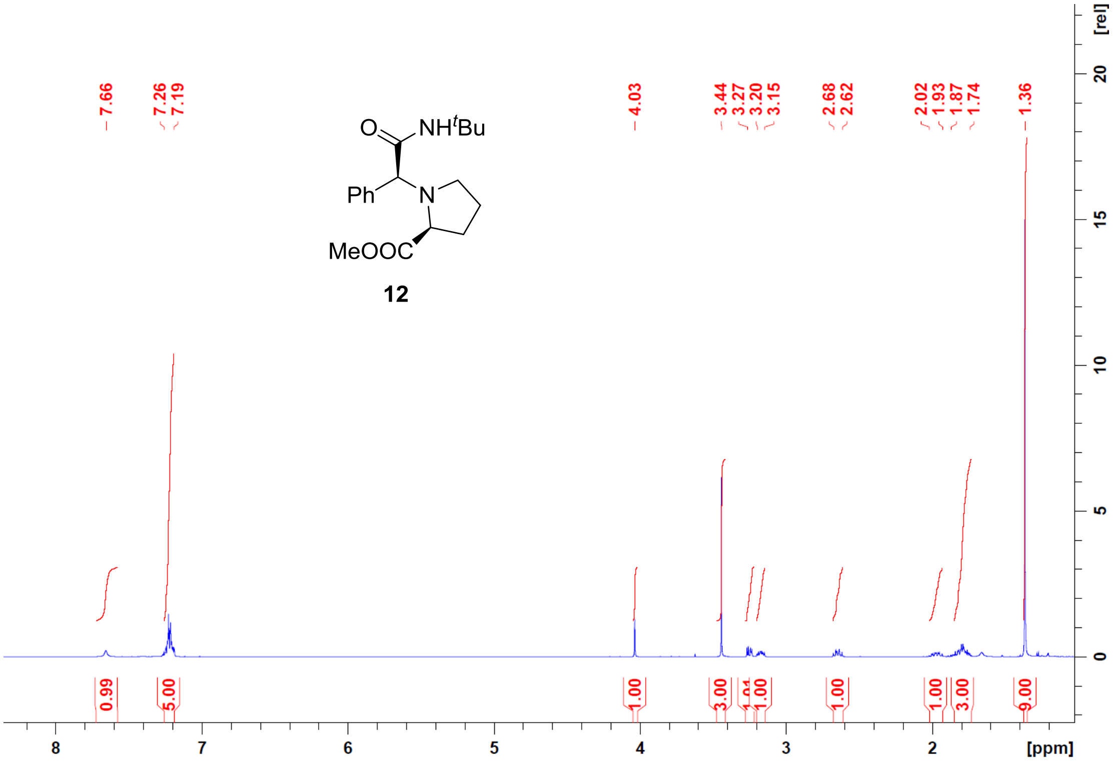

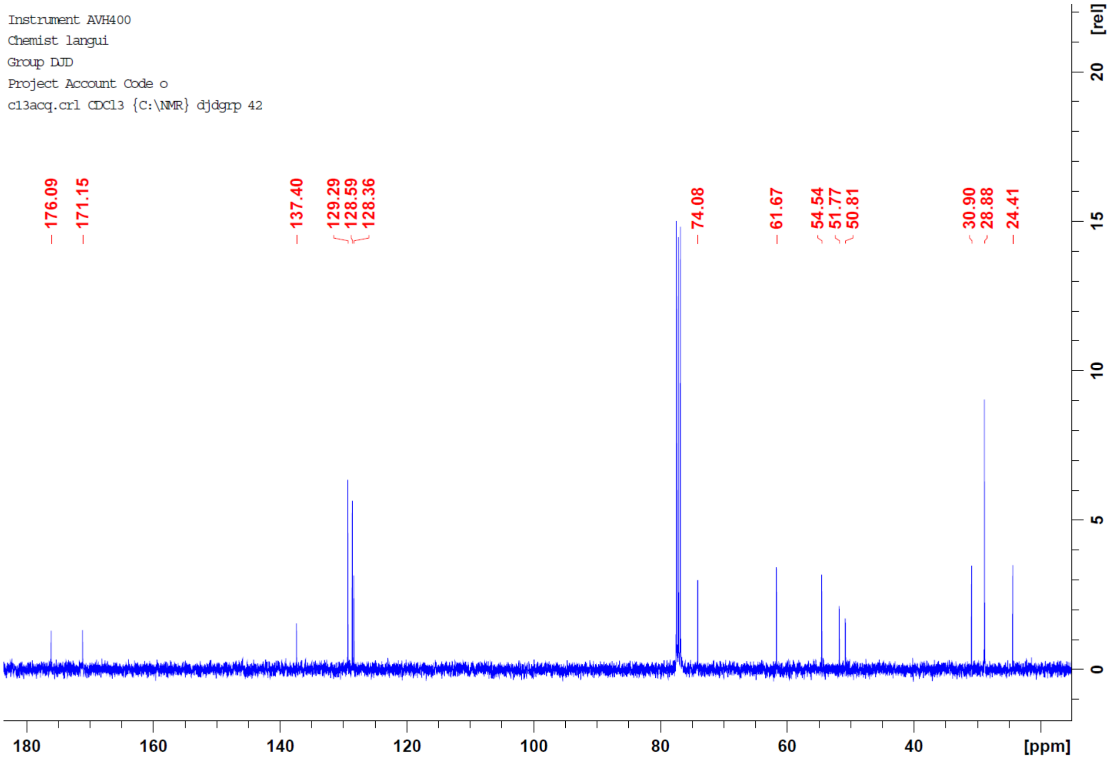

Supplementary Figure 11. ^{1}H NMR and ^{13}C NMR spectra of product **12**.

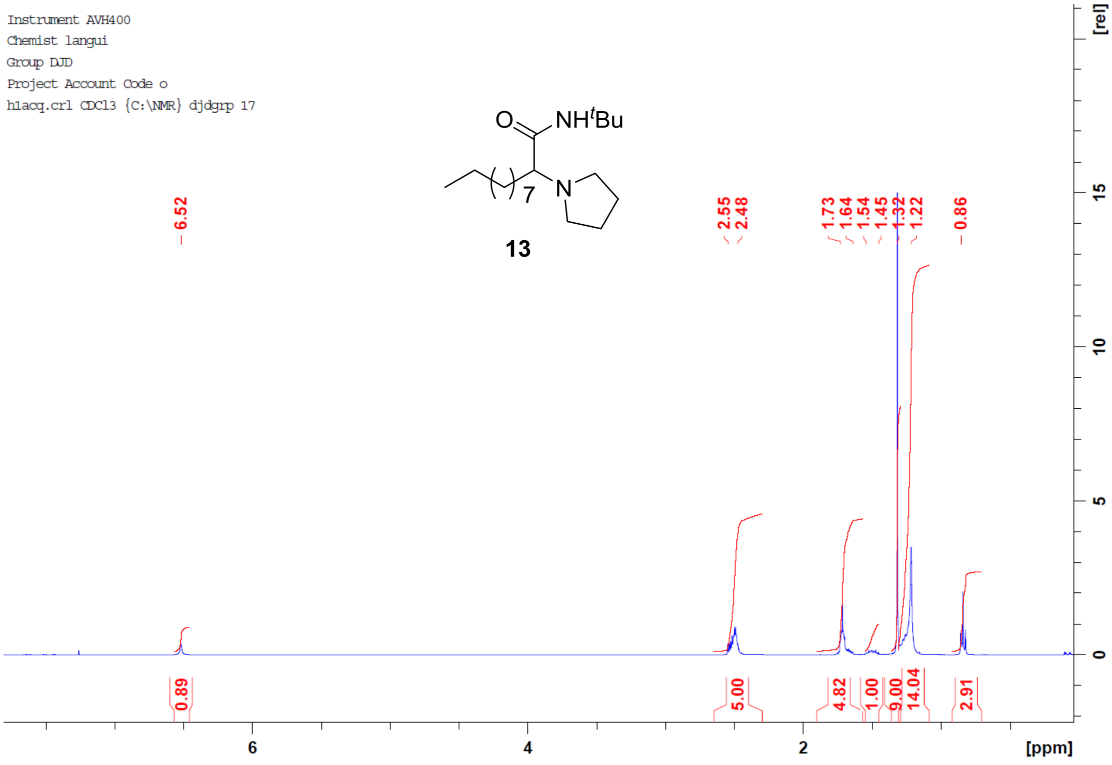

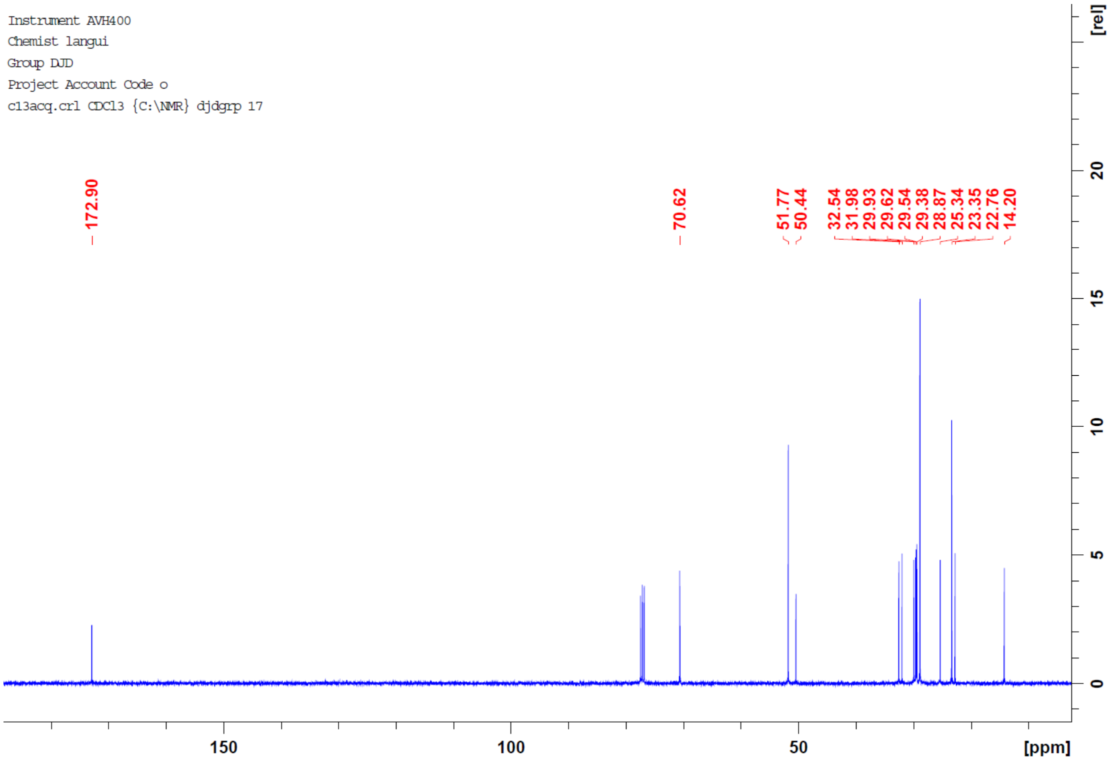

Supplementary Figure 12. ^{1}H NMR and ^{13}C NMR spectra of product **13**.

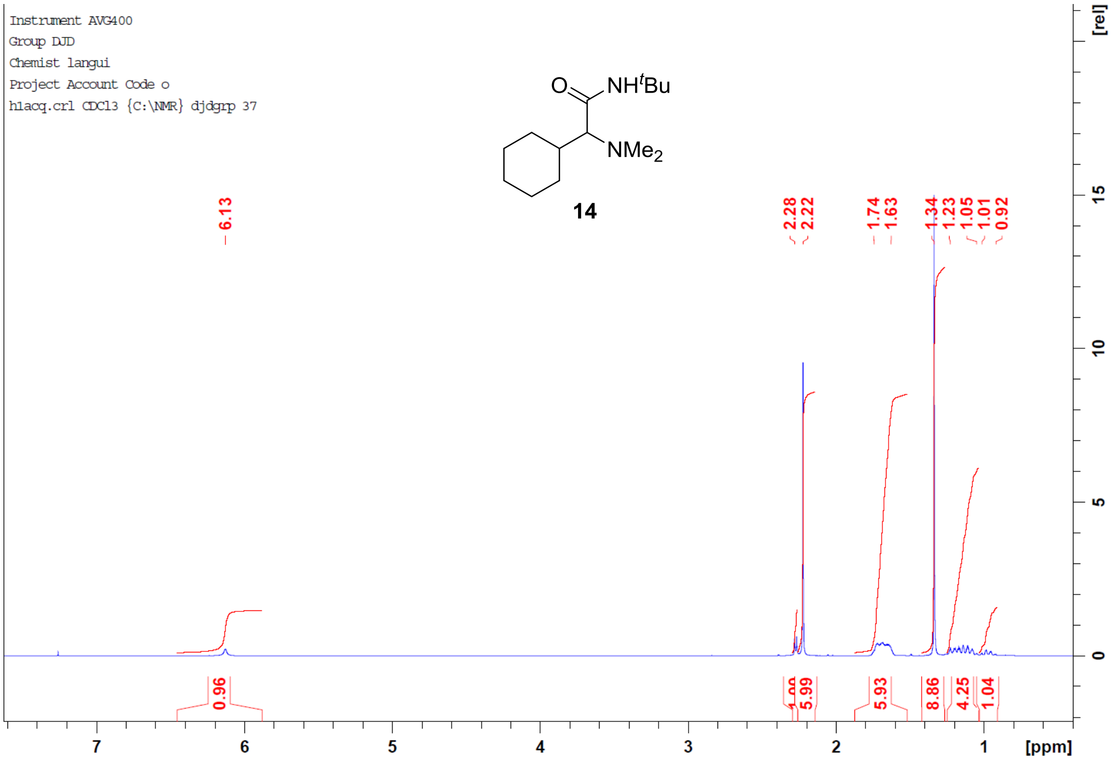

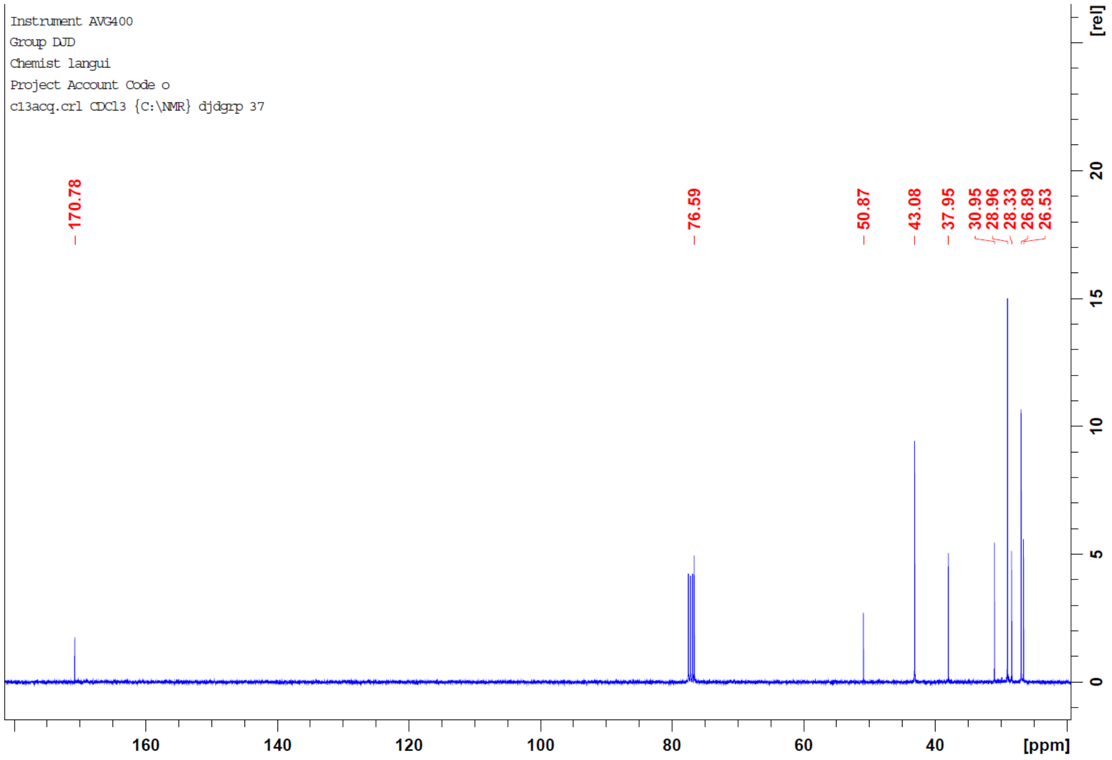

Supplementary Figure 13. ^{1}H NMR and ^{13}C NMR spectra of product **14**.

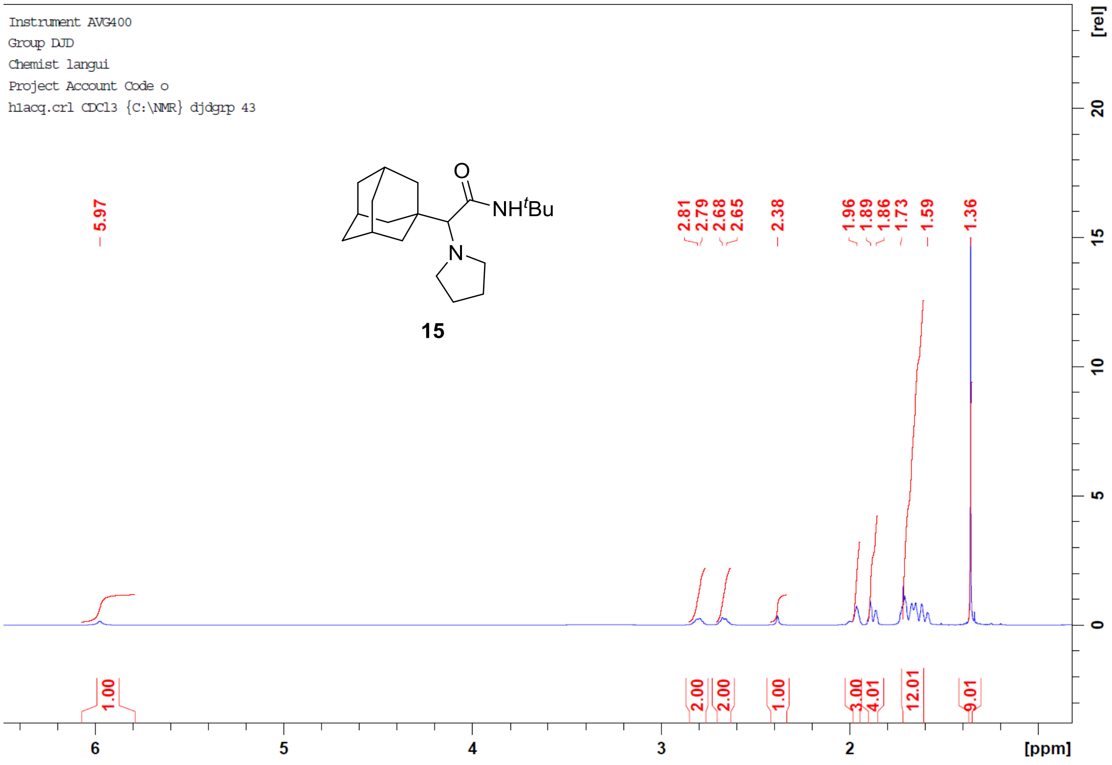

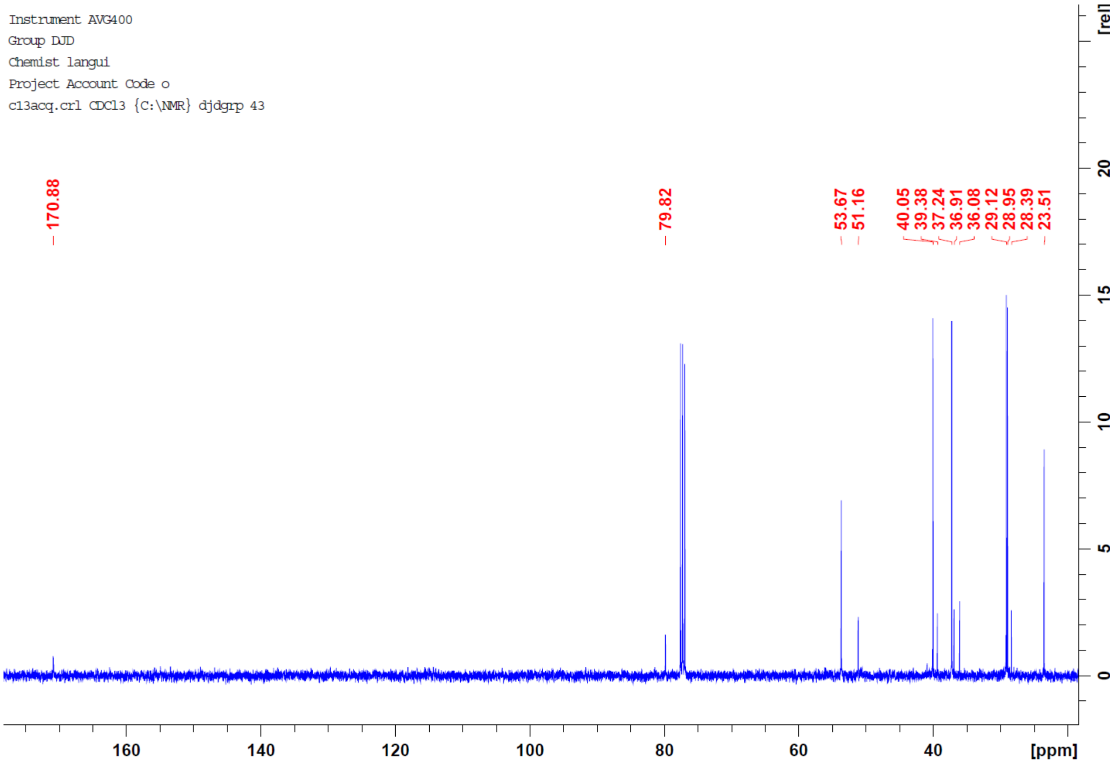

Supplementary Figure 14. ^{1}H NMR and ^{13}C NMR spectra of product **15**.

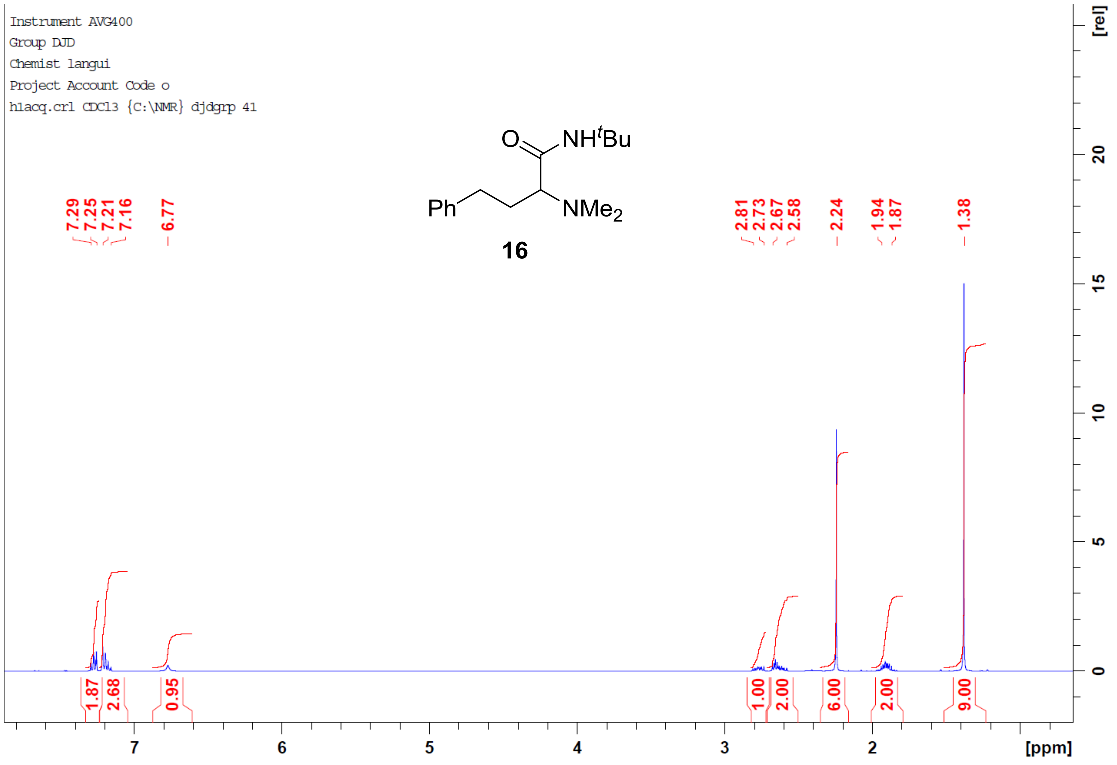

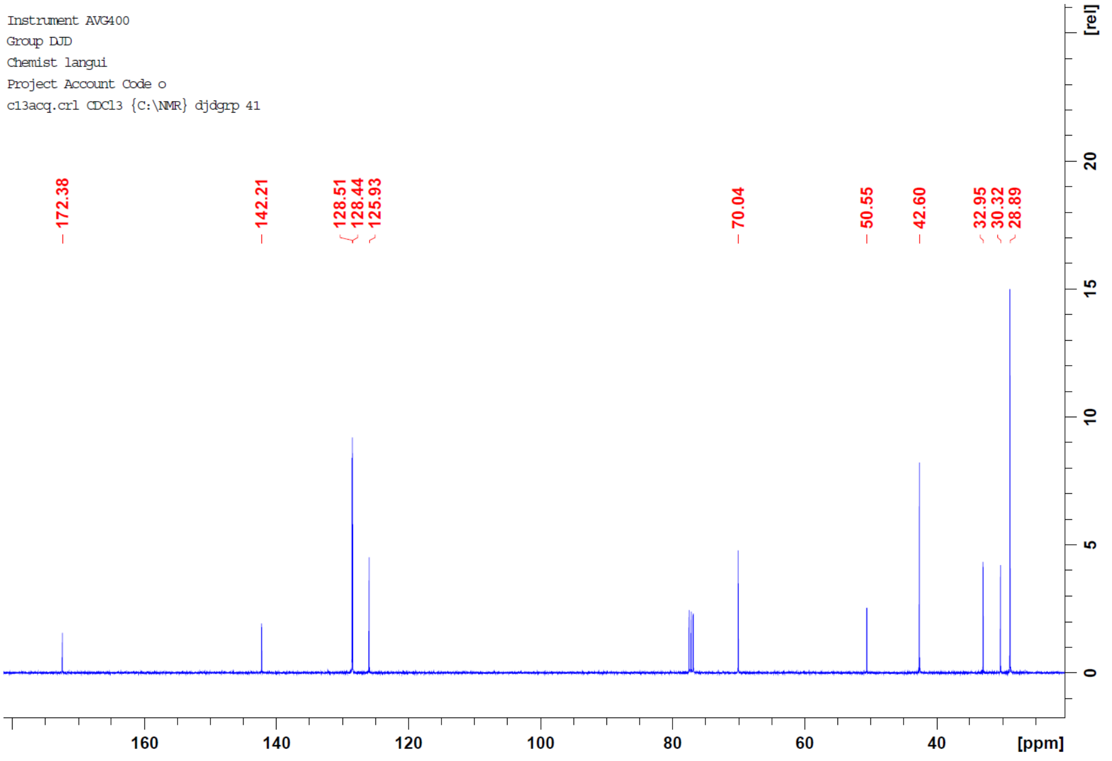

Supplementary Figure 15. ^{1}H NMR and ^{13}C NMR spectra of product **16**.

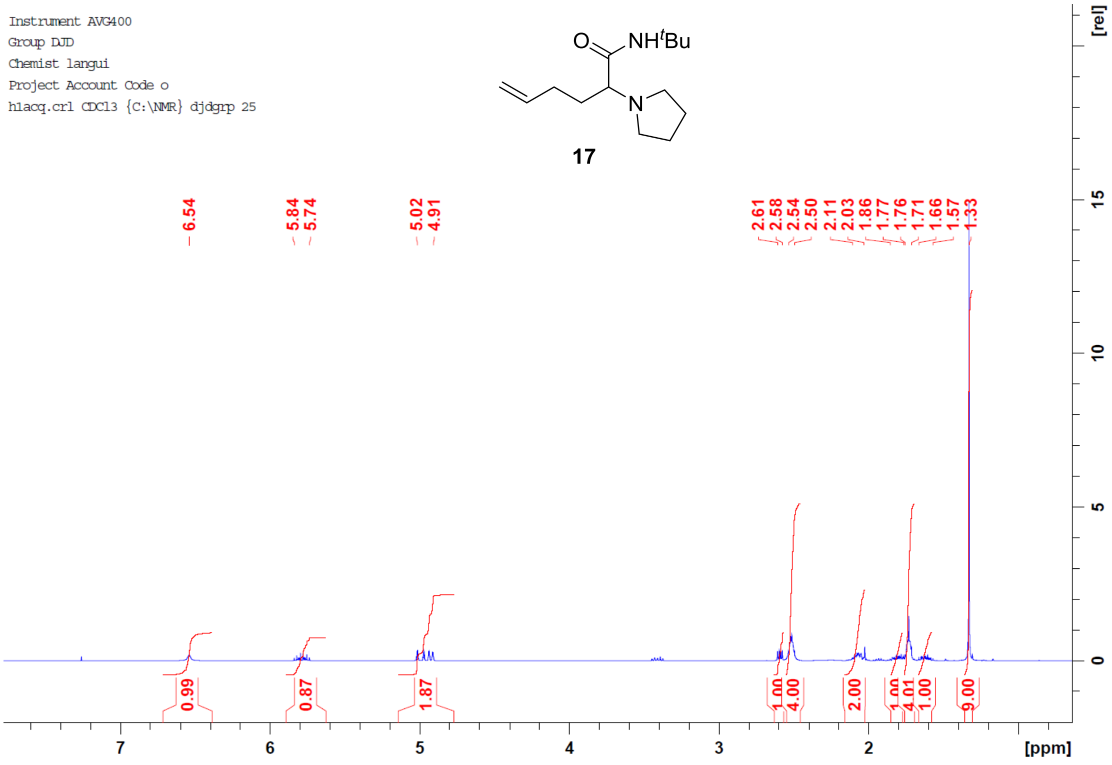

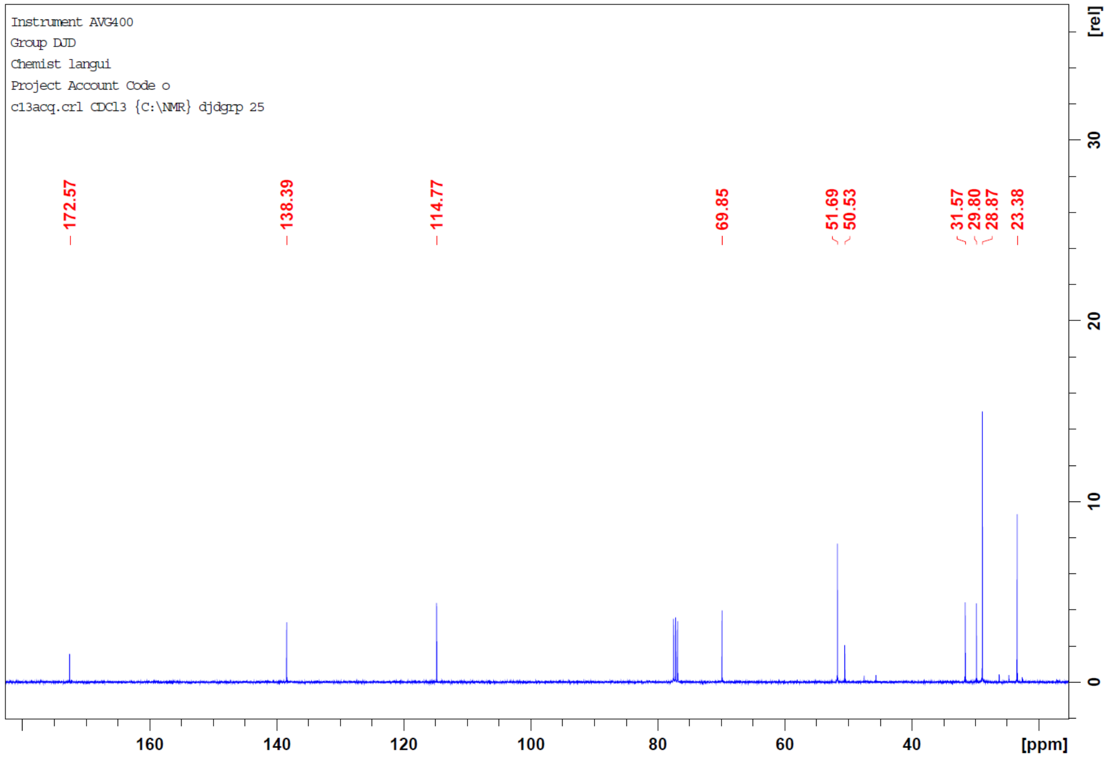

Supplementary Figure 16. ^{1}H NMR and ^{13}C NMR spectra of product **17**.

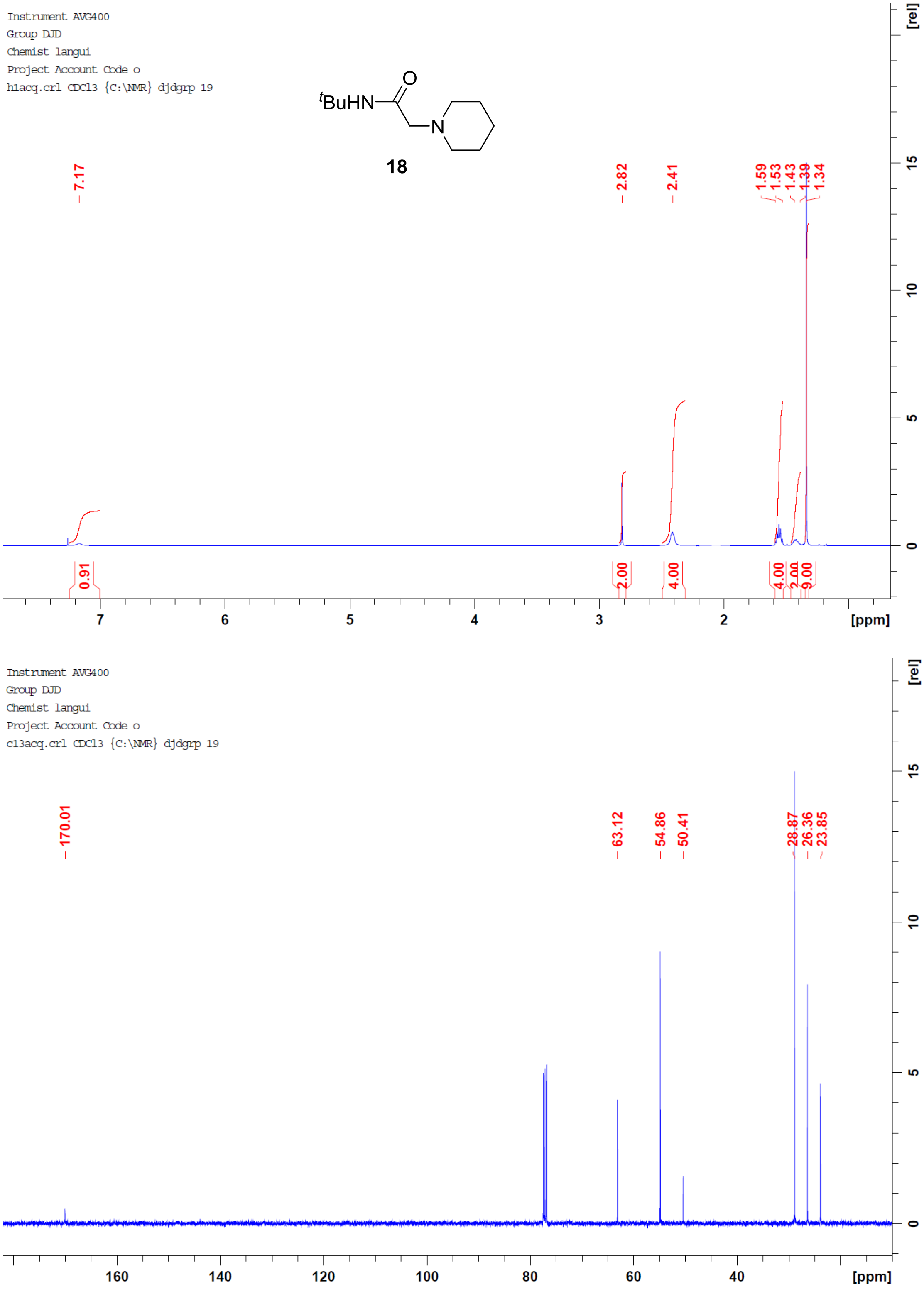

Supplementary Figure 17. ^{1}H NMR and ^{13}C NMR spectra of product **18**.

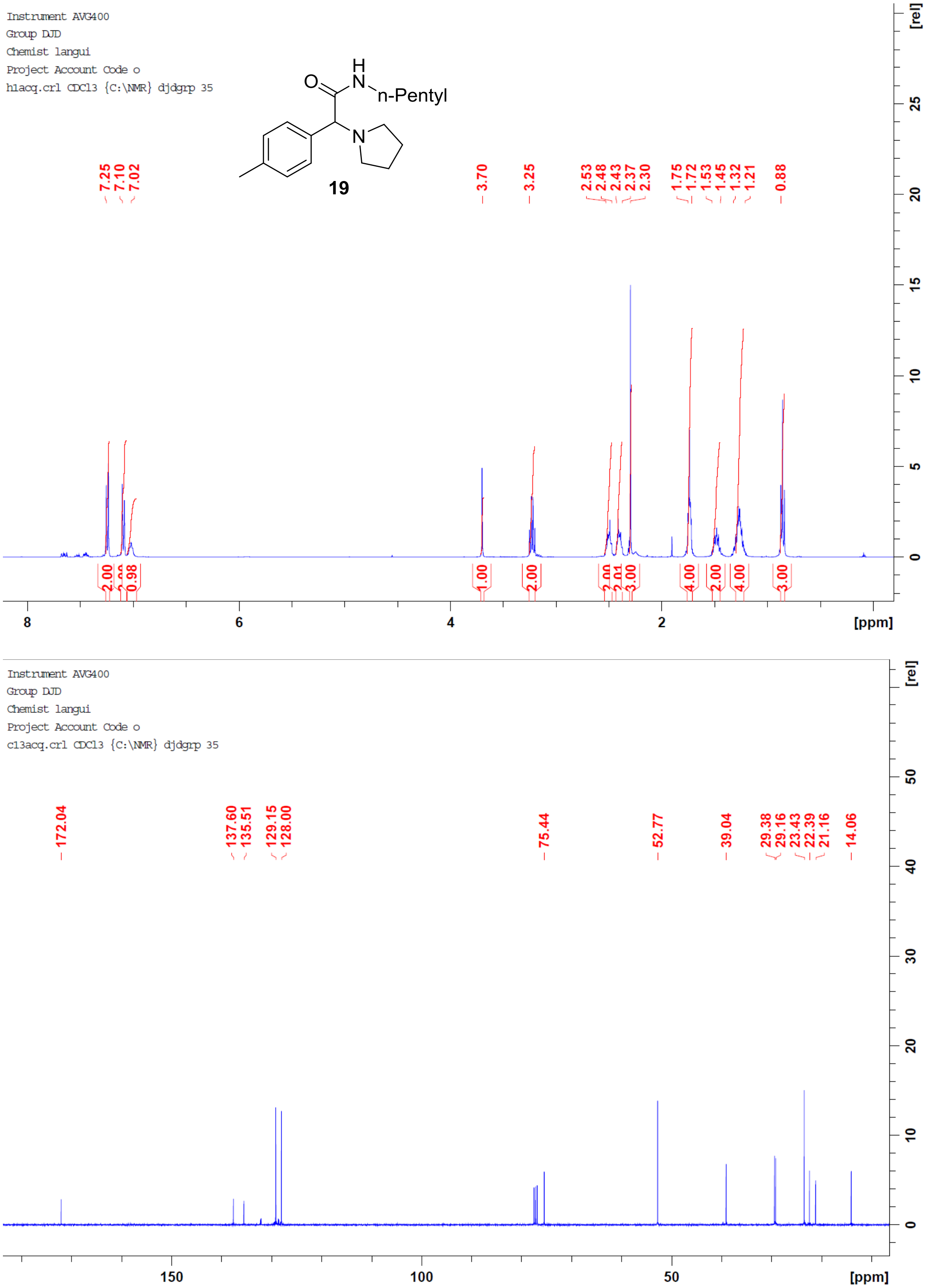

Supplementary Figure 18. ^{1}H NMR and ^{13}C NMR spectra of product **19**.

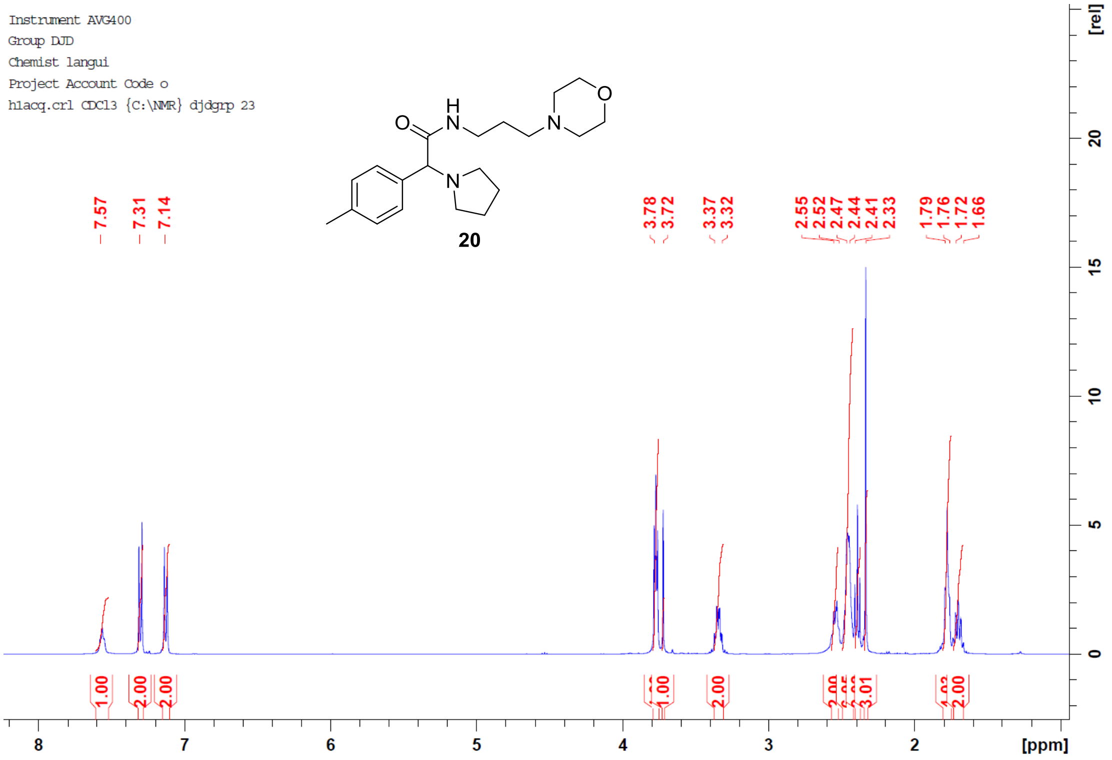

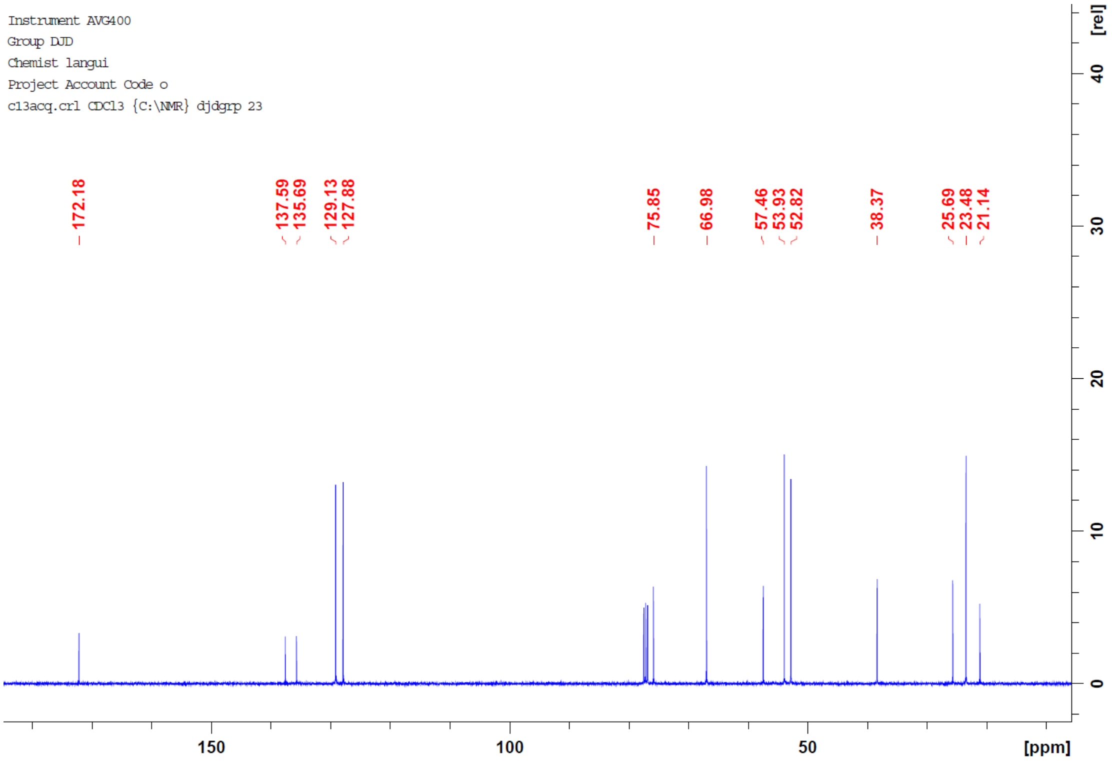

Supplementary Figure 19. ^{1}H NMR and ^{13}C NMR spectra of product **20**.

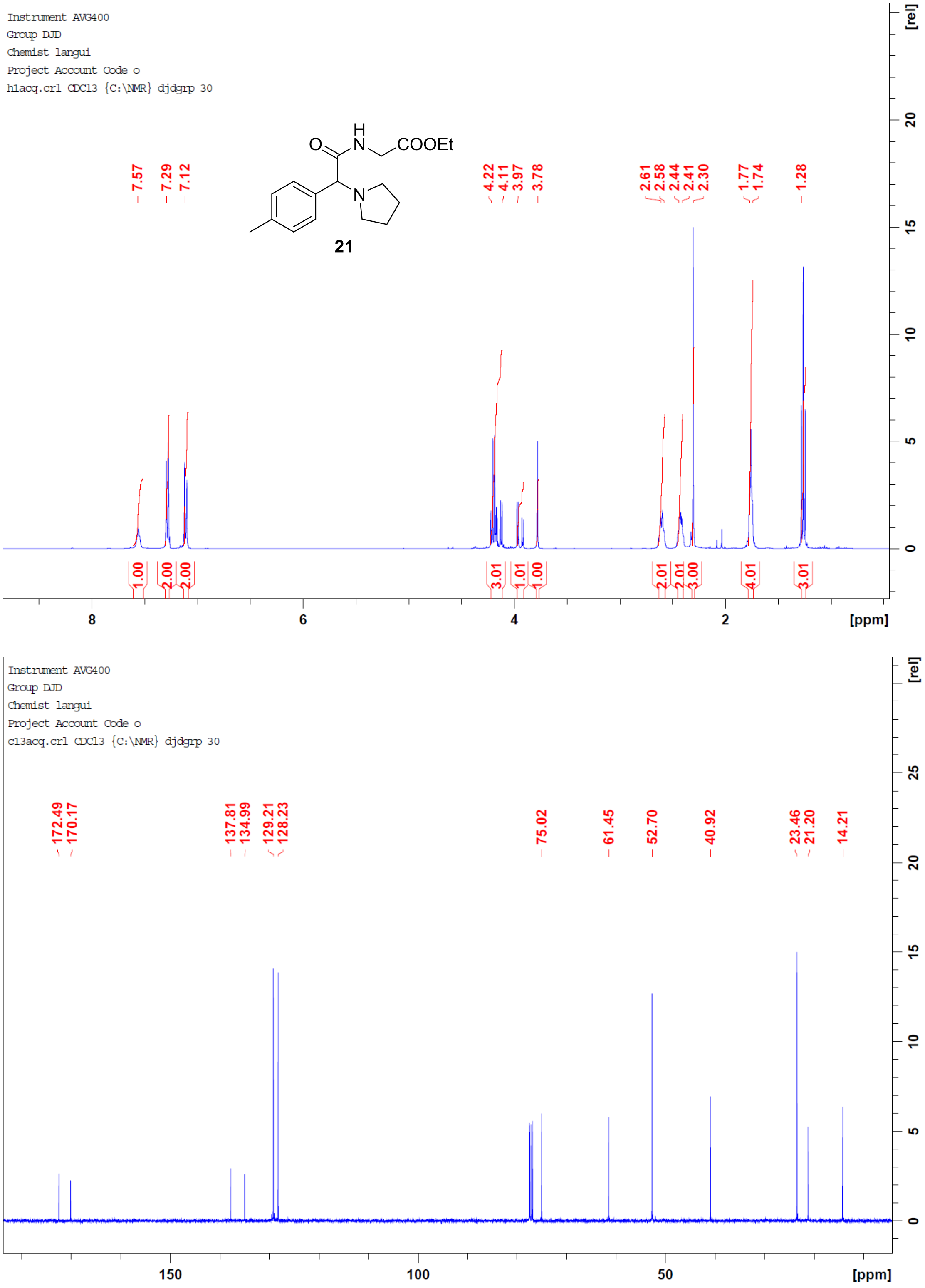

Supplementary Figure 20. ^{1}H NMR and ^{13}C NMR spectra of product **21**.

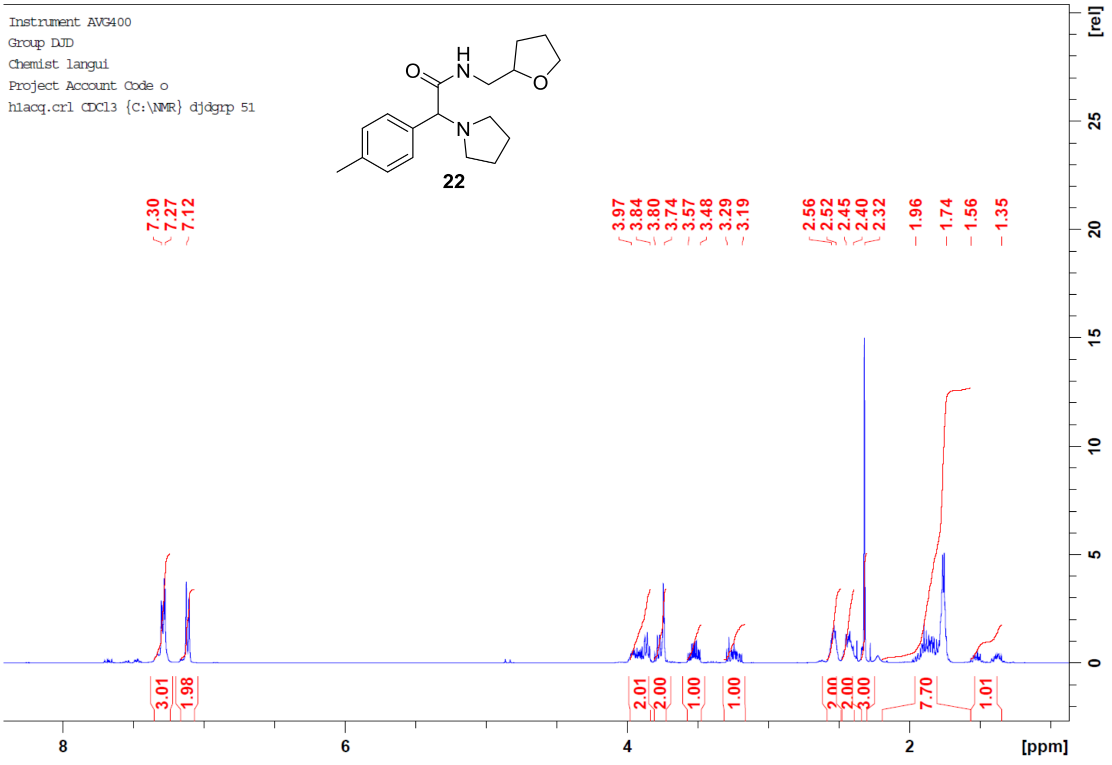

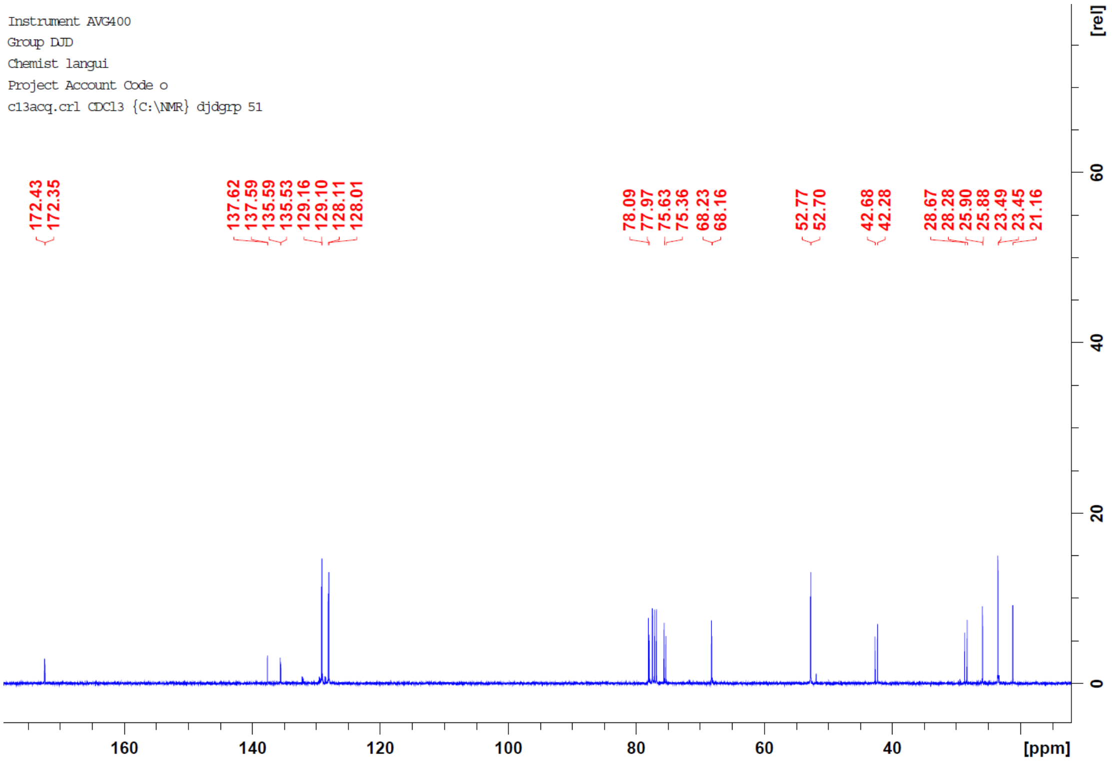

Supplementary Figure 21. ^{1}H NMR and ^{13}C NMR spectra of product **22**.

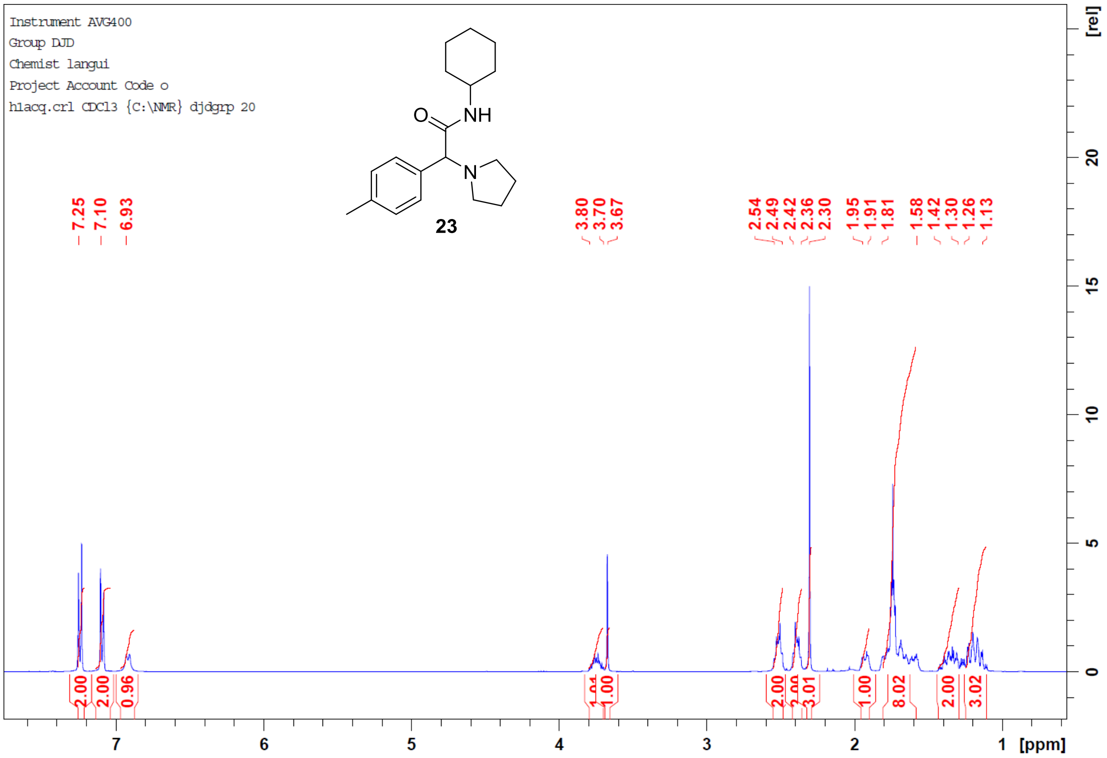

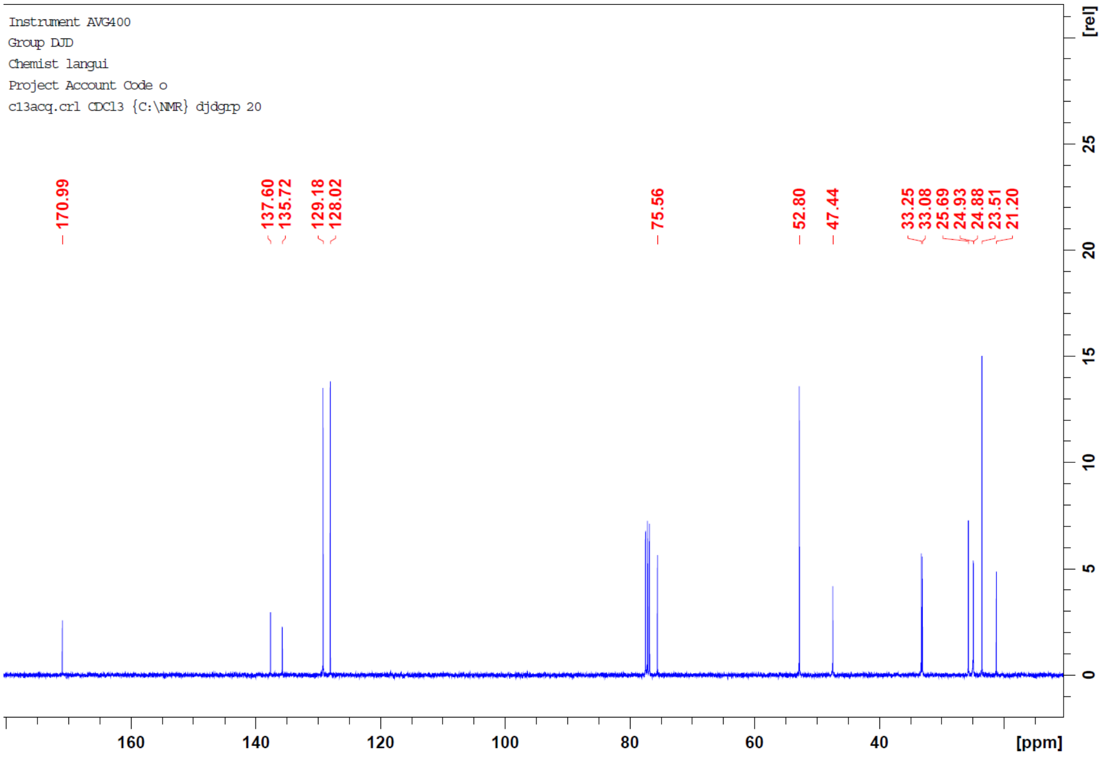

Supplementary Figure 22. ^{1}H NMR and ^{13}C NMR spectra of product **23**.

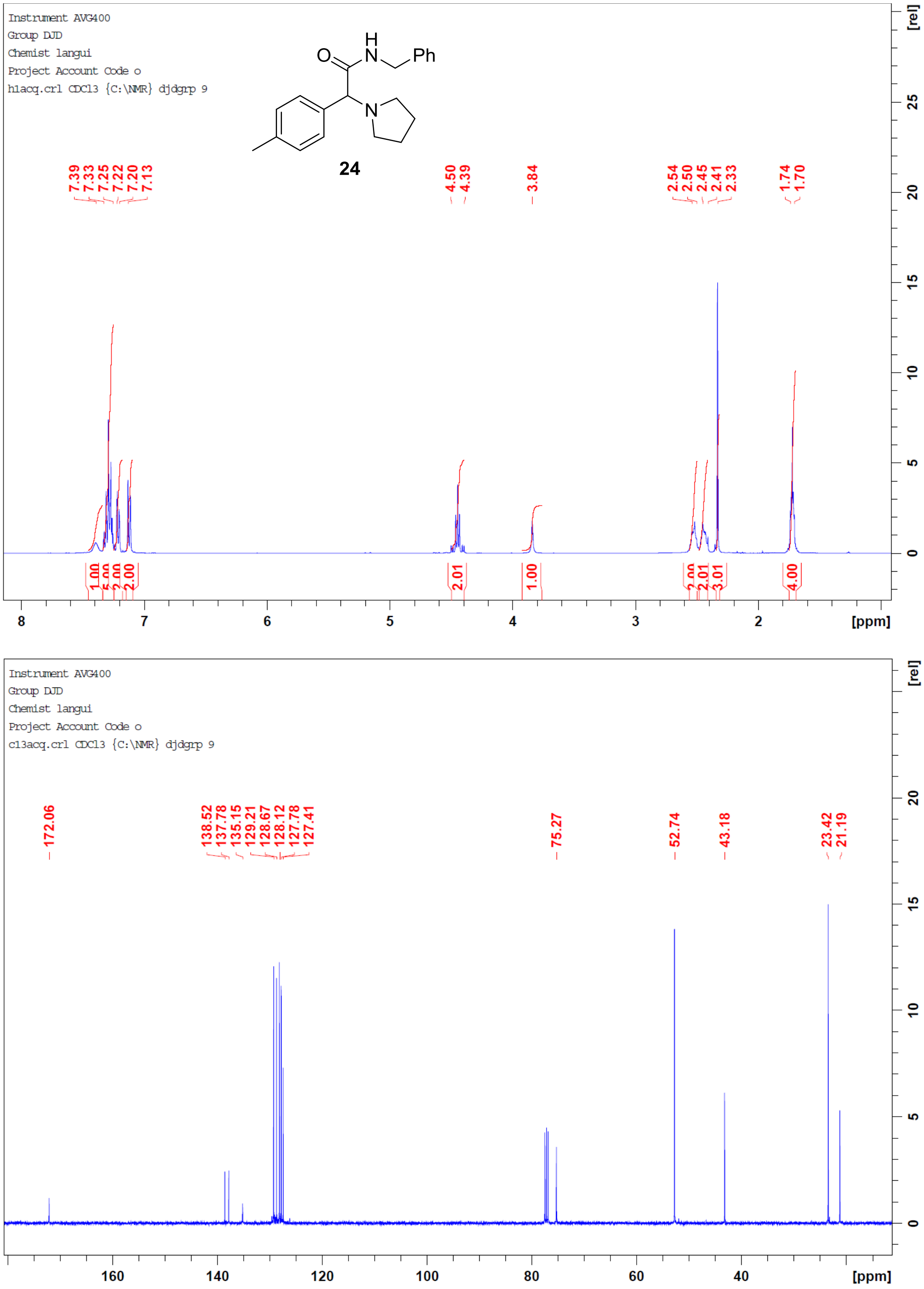

Supplementary Figure 23. ^{1}H NMR and ^{13}C NMR spectra of product **24**.

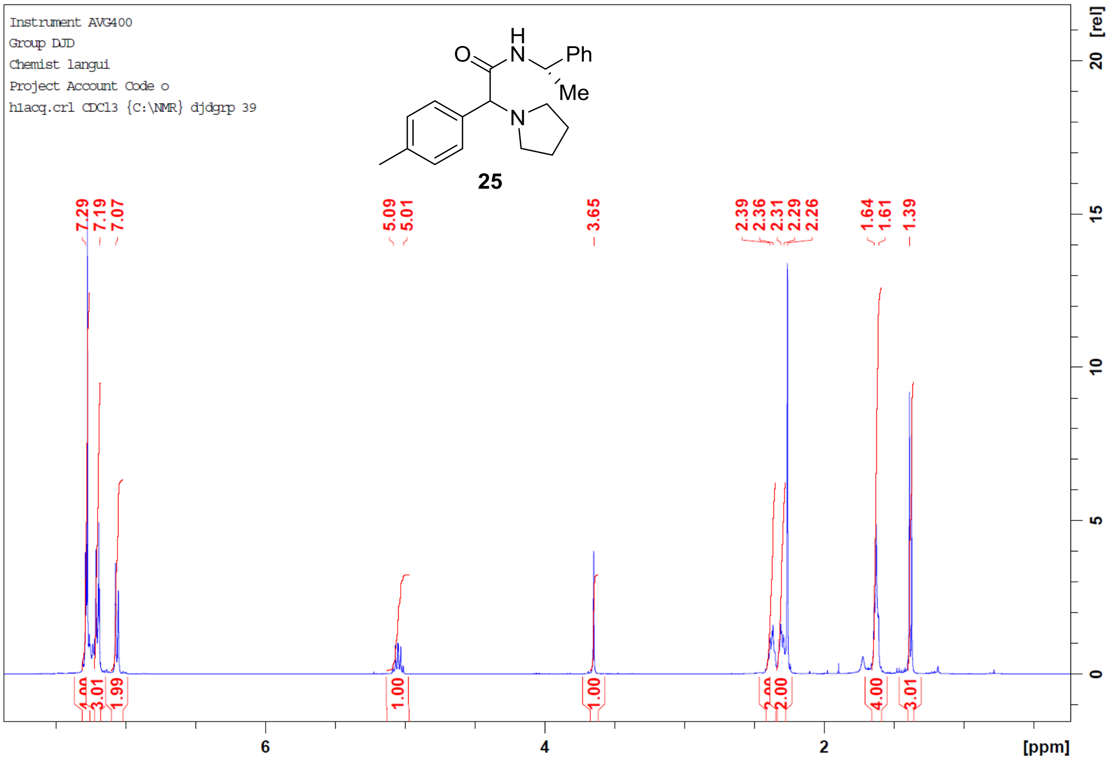

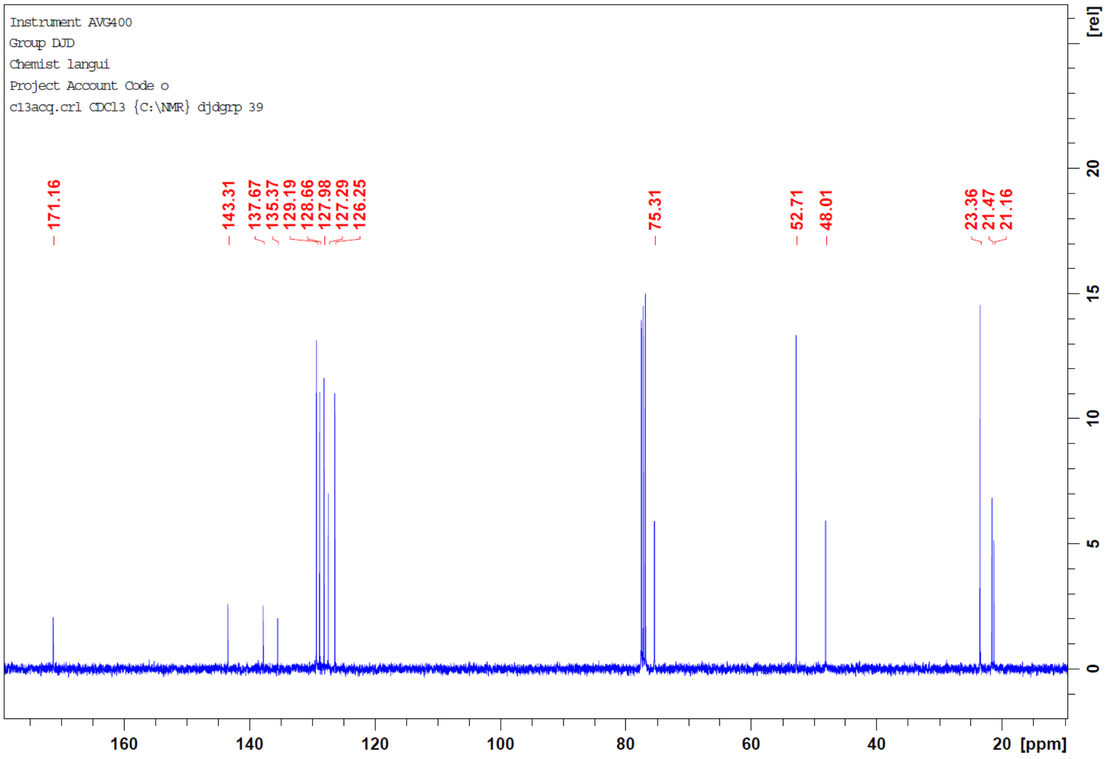

Supplementary Figure 24. ^{1}H NMR and ^{13}C NMR spectra of product **25**.

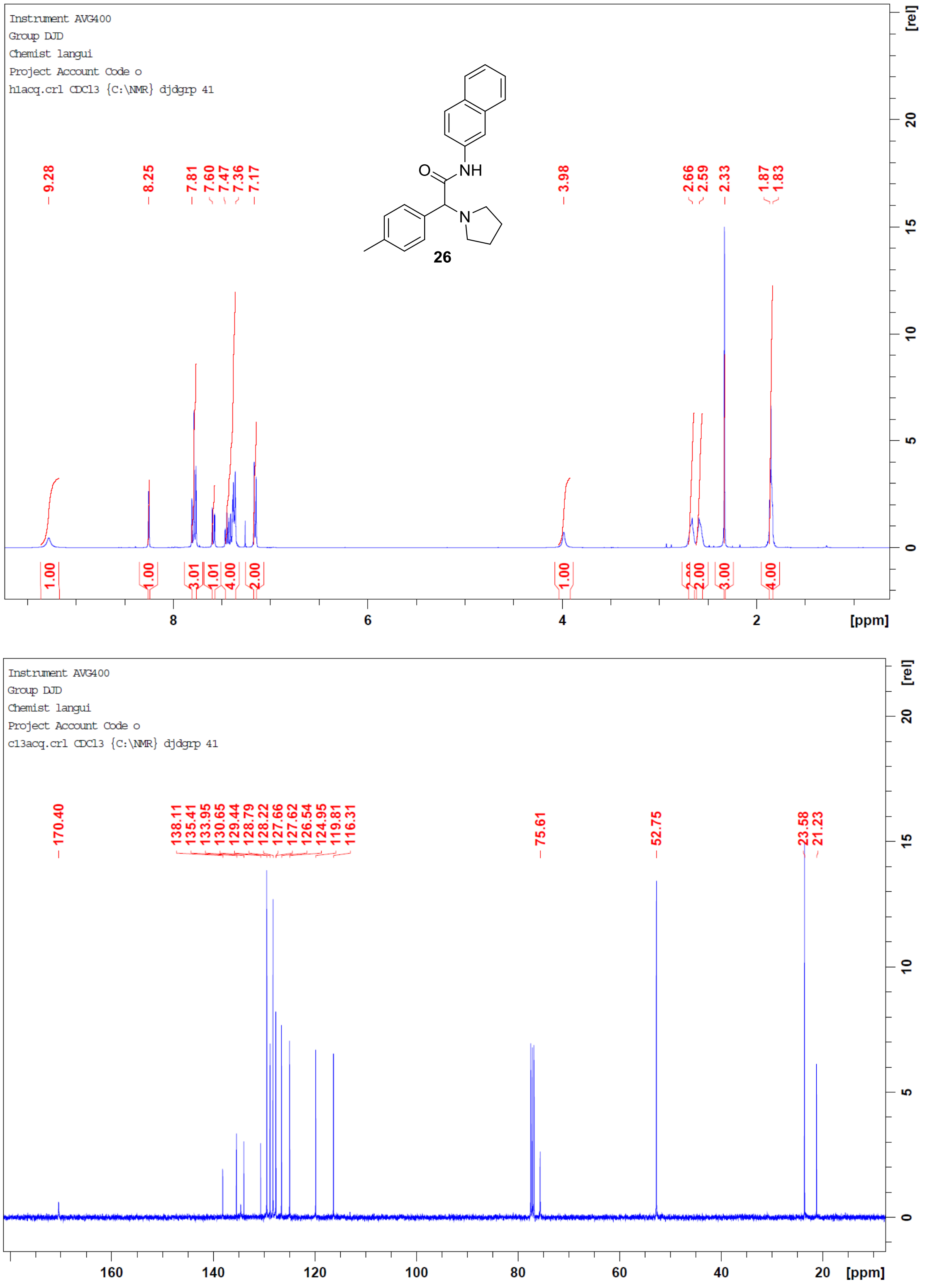

Supplementary Figure 25. ^{1}H NMR and ^{13}C NMR spectra of product **26**.

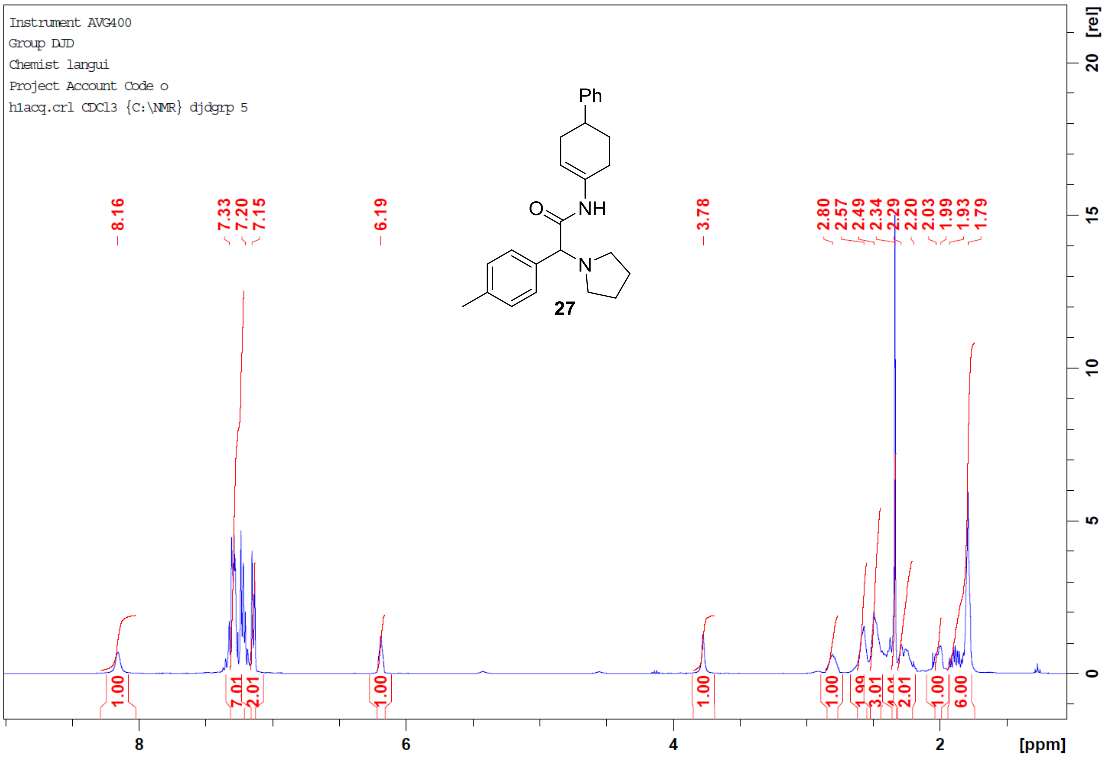

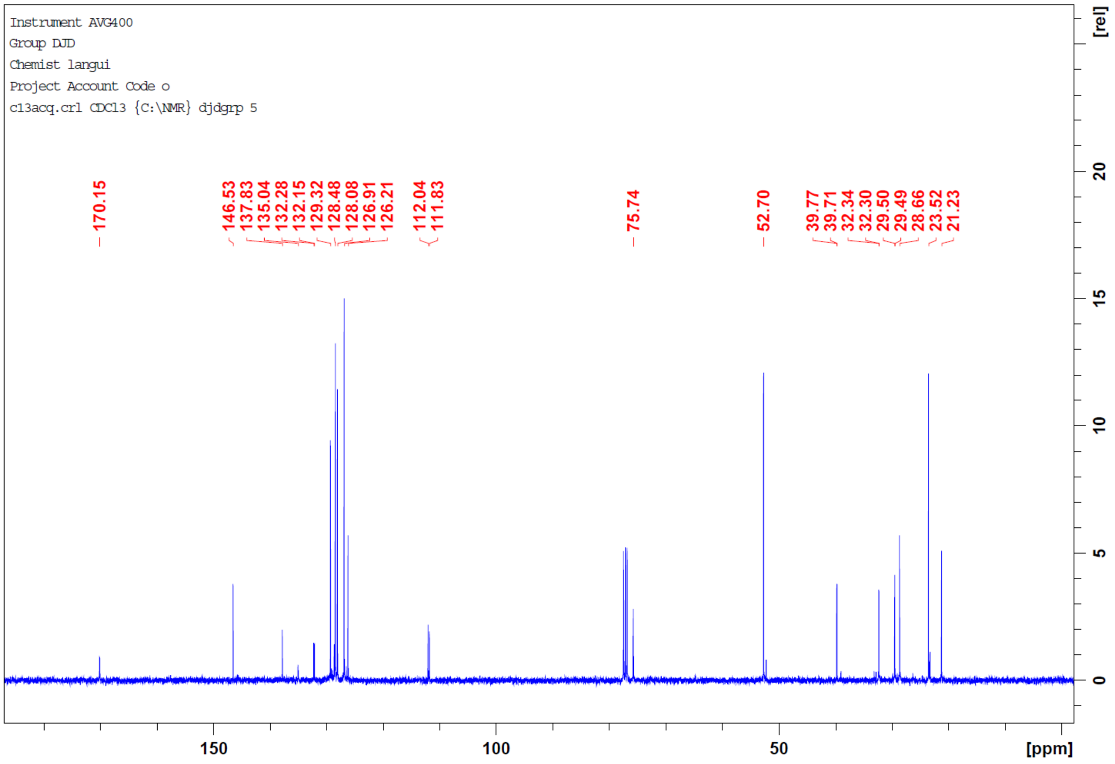

Supplementary Figure 26. ^{1}H NMR and ^{13}C NMR spectra of product **27**.

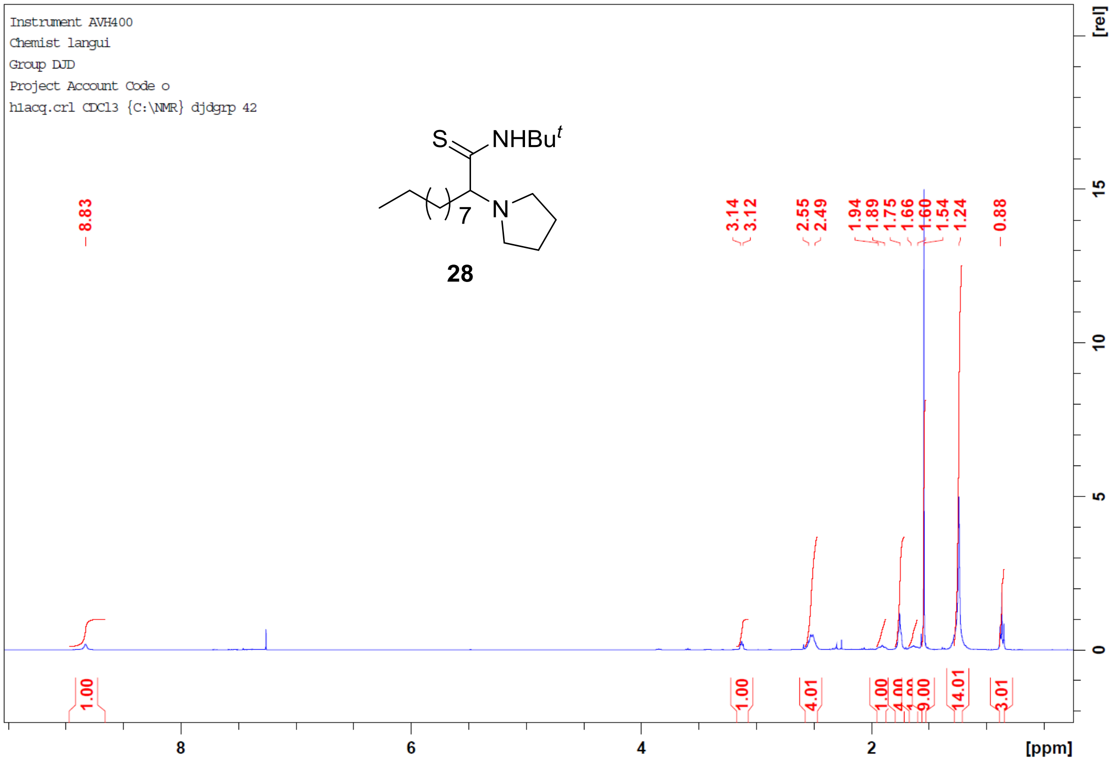

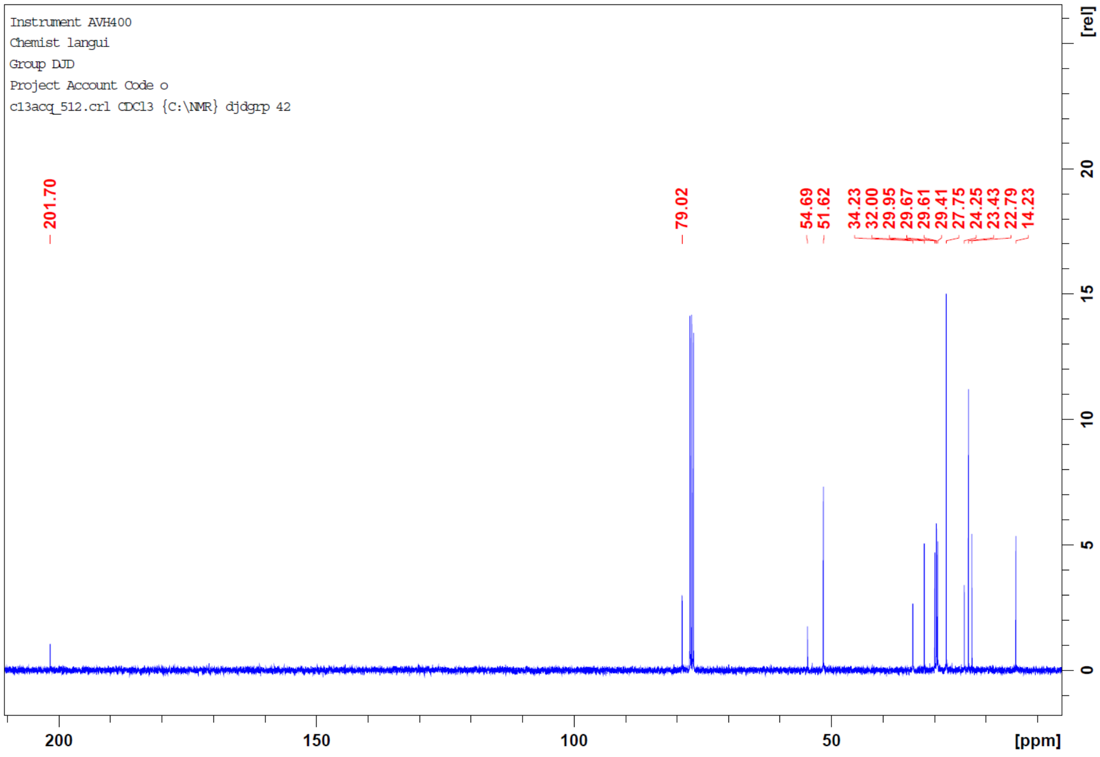

Supplementary Figure 27. ^{1}H NMR and ^{13}C NMR spectra of product **28**.

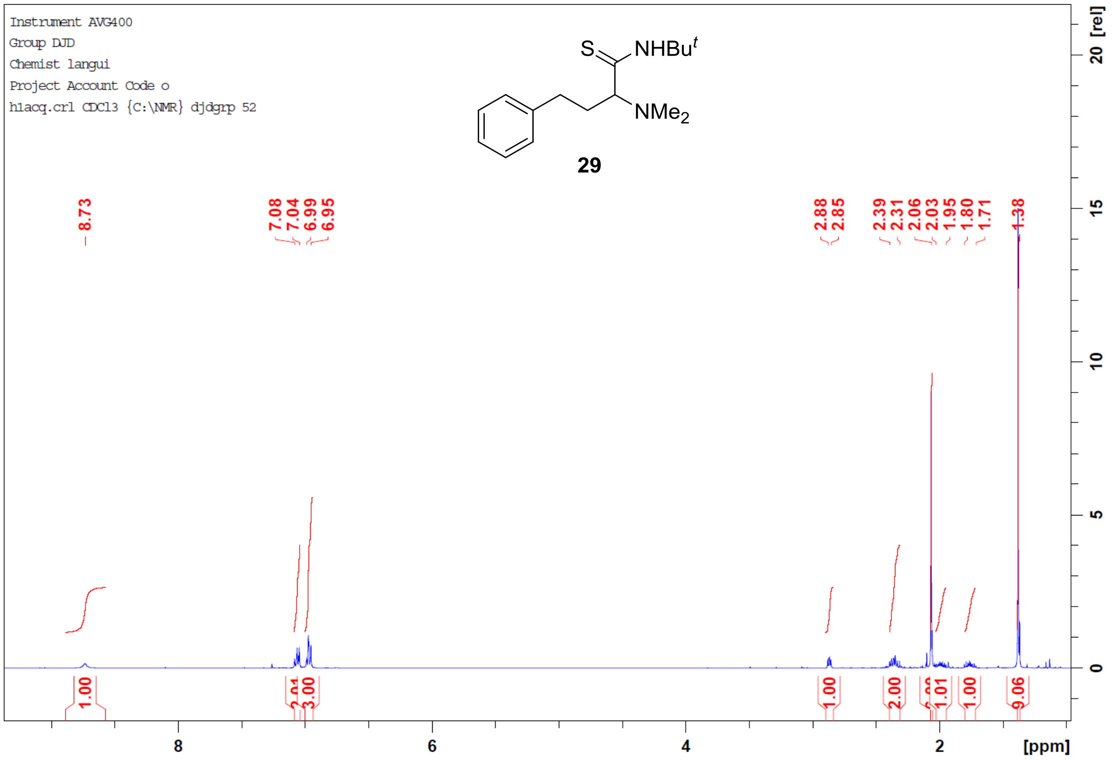

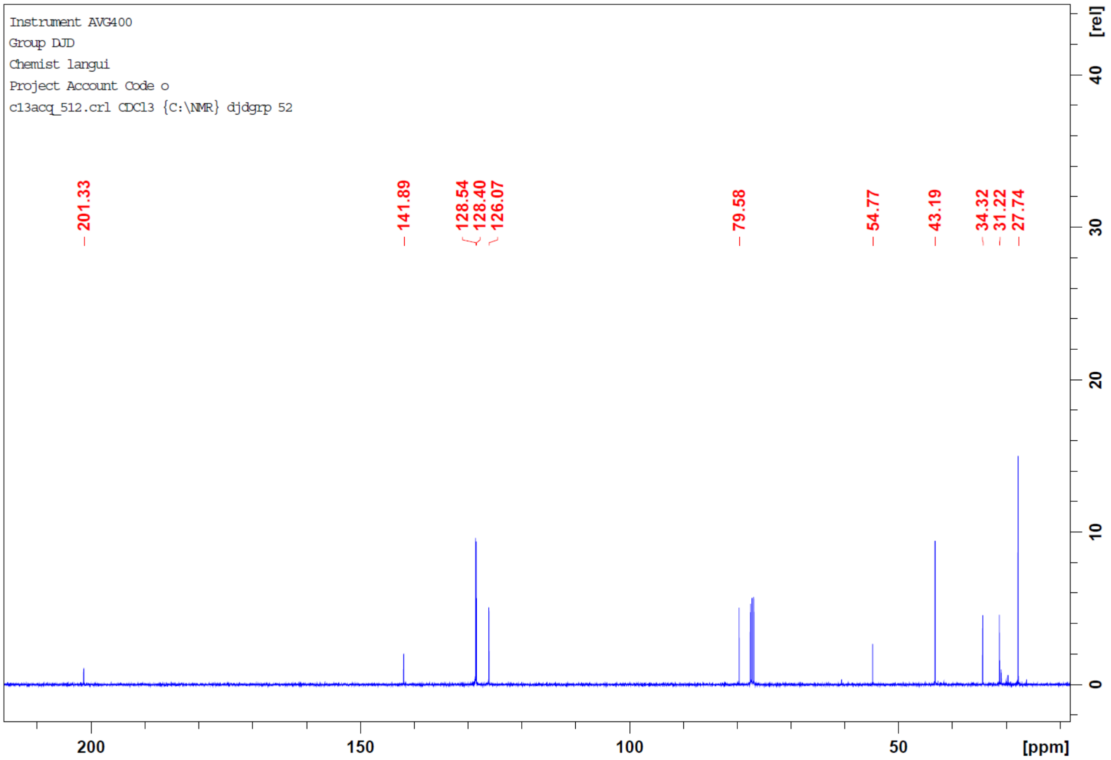

Supplementary Figure 28. ^{1}H NMR and ^{13}C NMR spectra of product **29**.

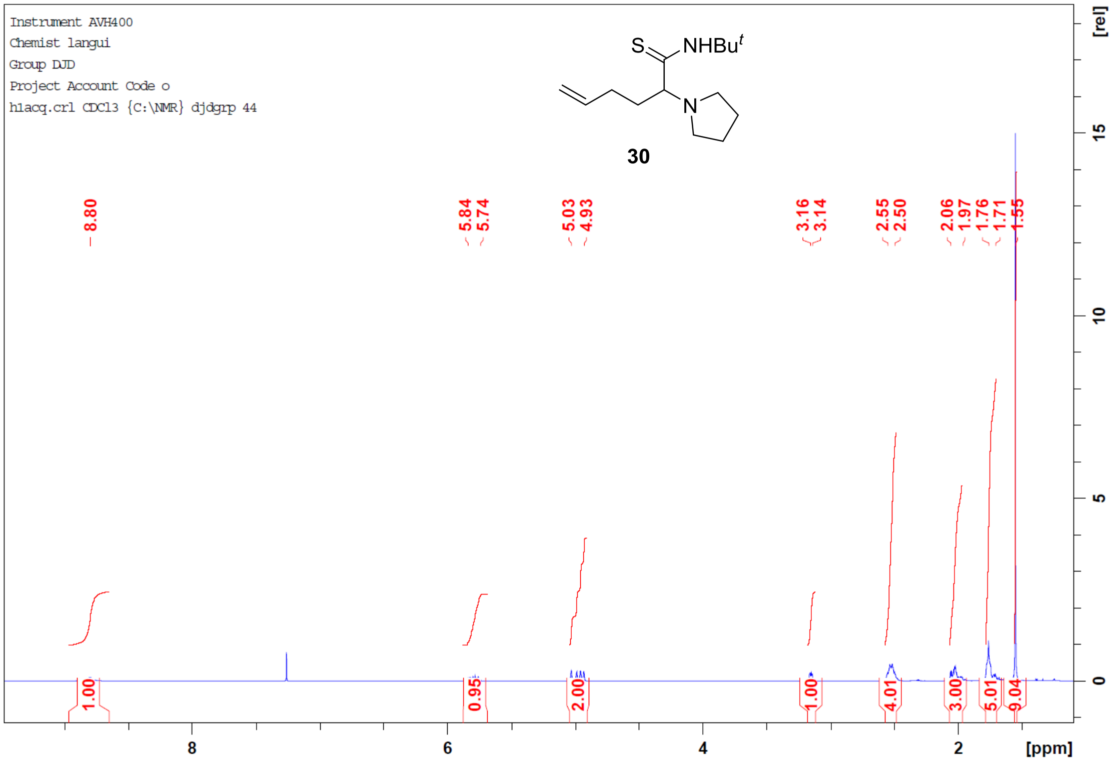

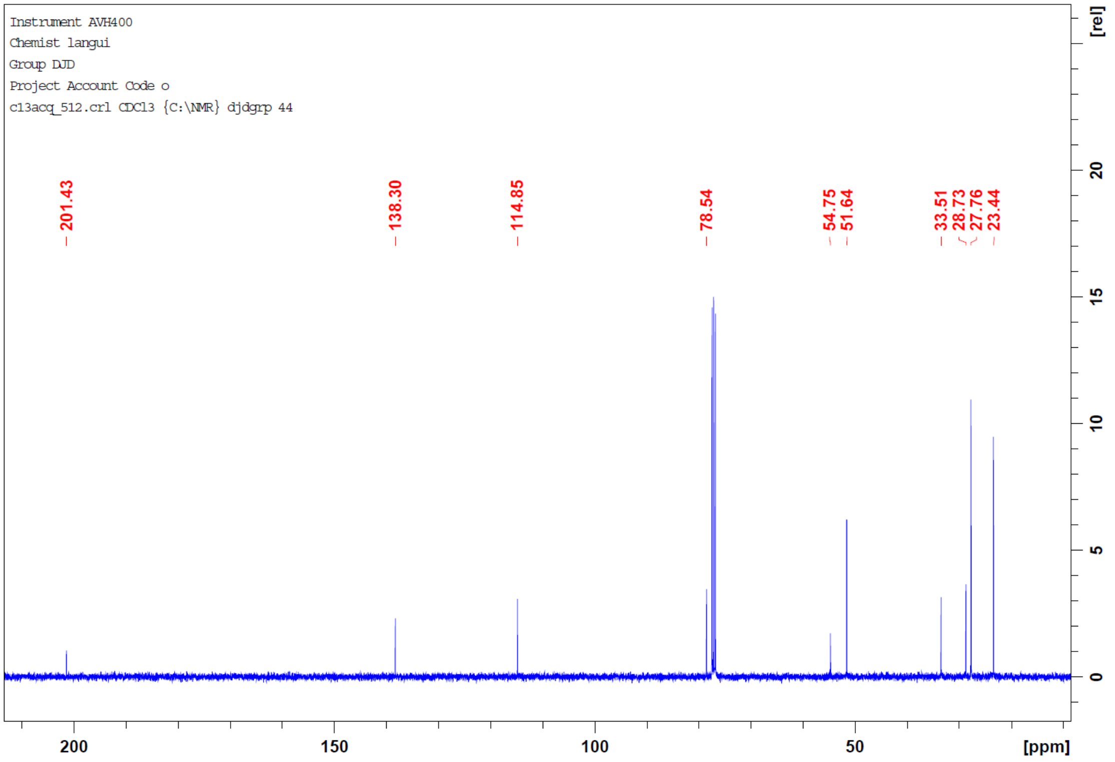

Supplementary Figure 29. ^{1}H NMR and ^{13}C NMR spectra of product **30**.

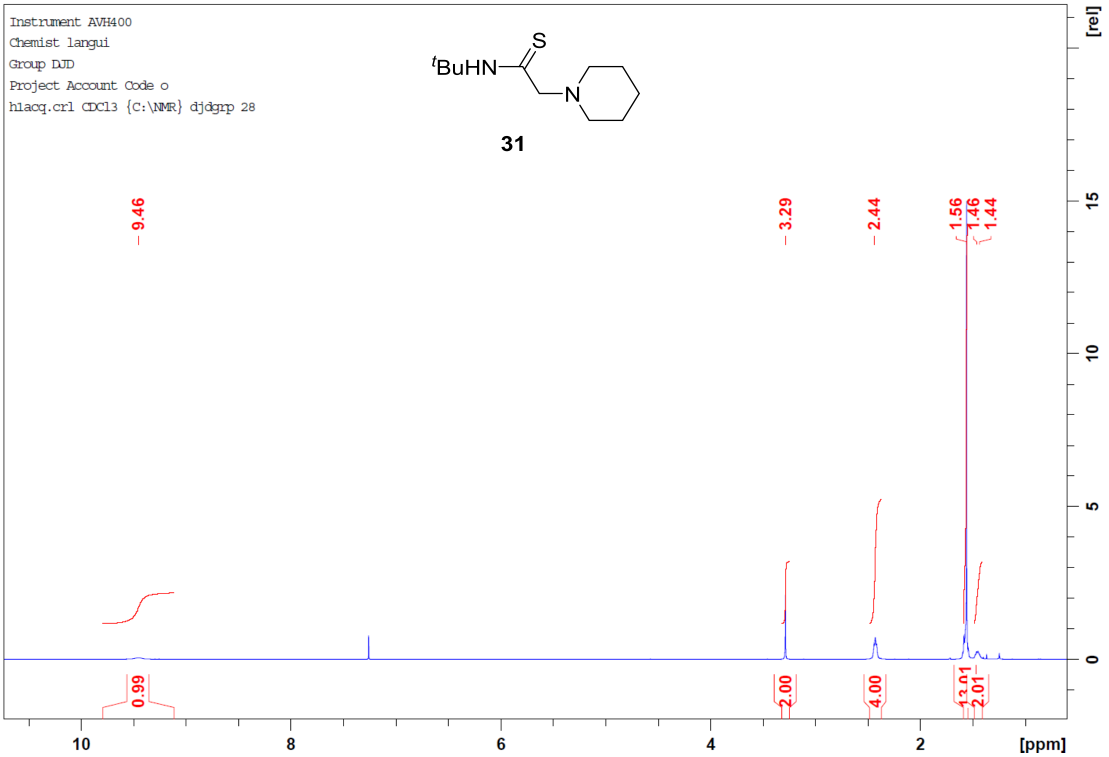

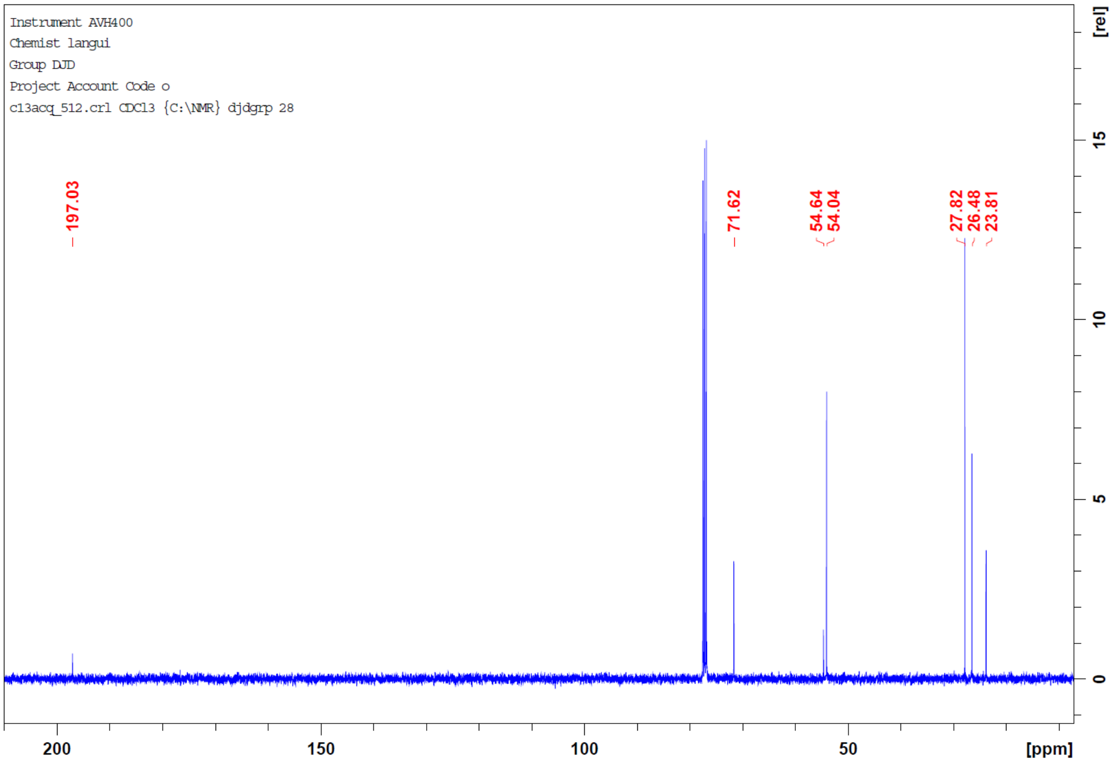

Supplementary Figure 30. ^{1}H NMR and ^{13}C NMR spectra of product **31**.

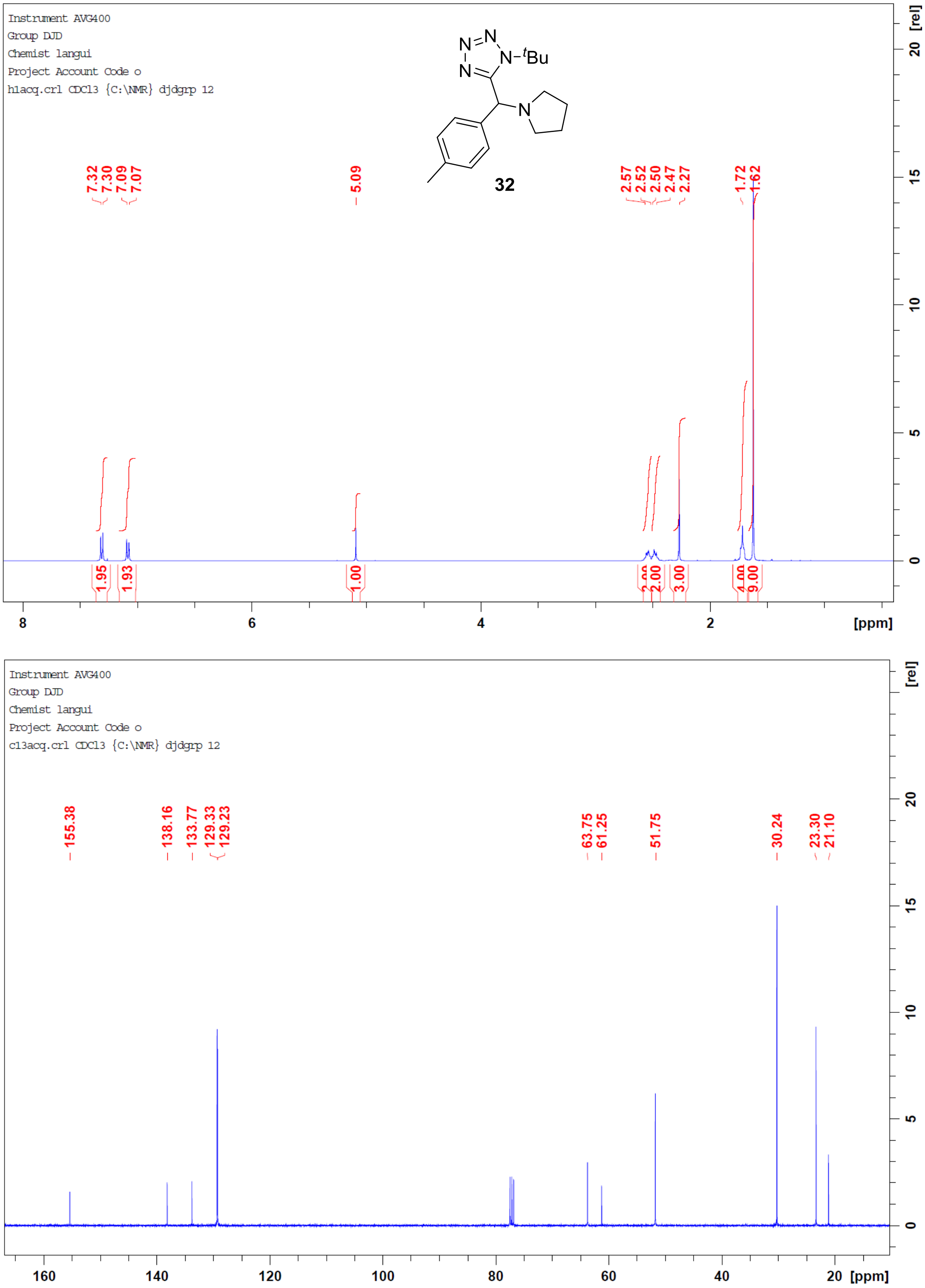

Supplementary Figure 31. ^{1}H NMR and ^{13}C NMR spectra of product **32**.

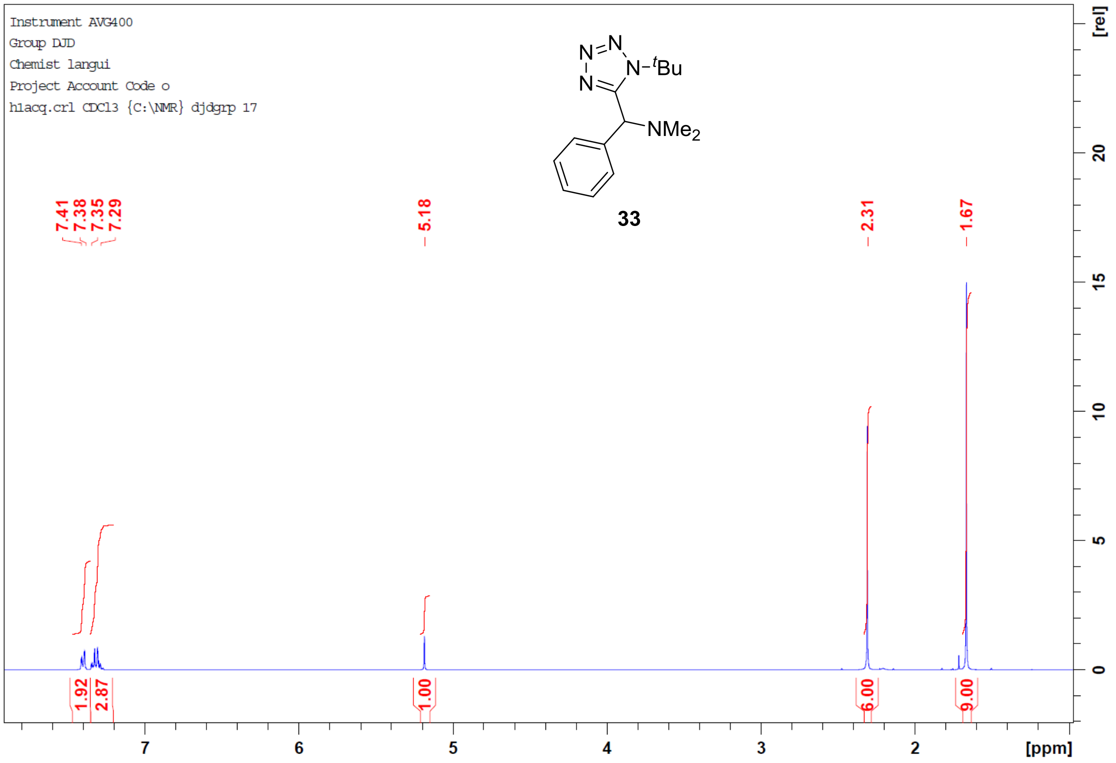

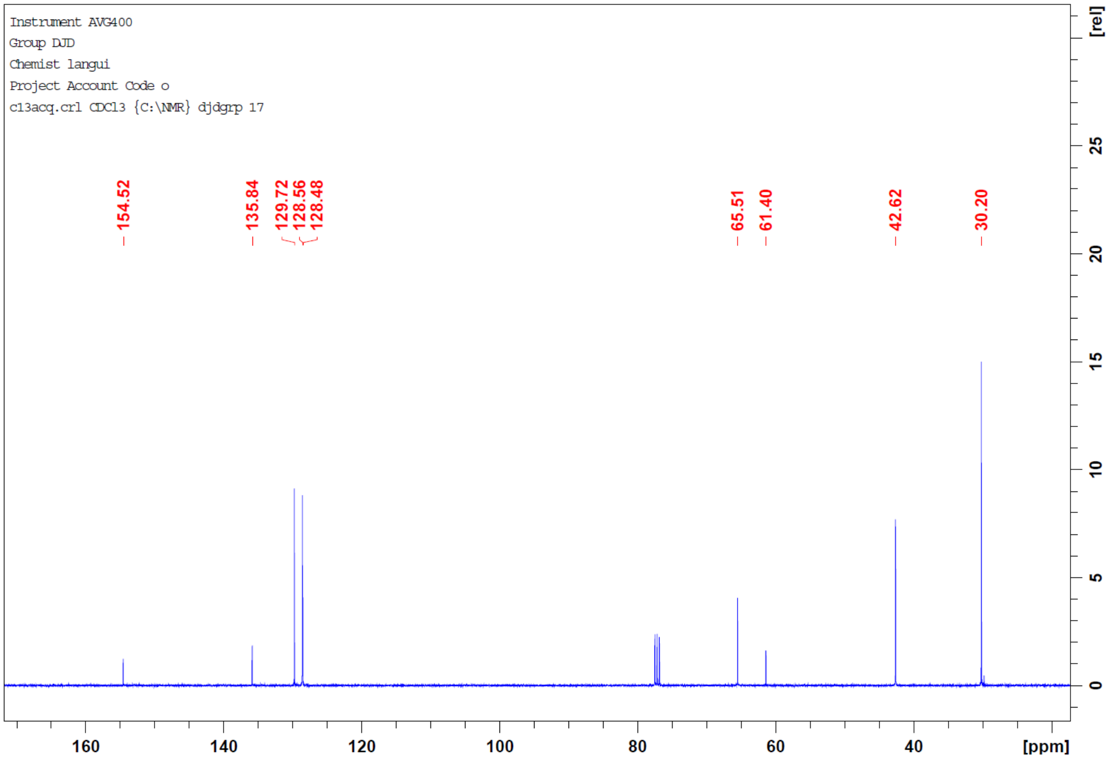

Supplementary Figure 32. ^{1}H NMR and ^{13}C NMR spectra of product **33**.

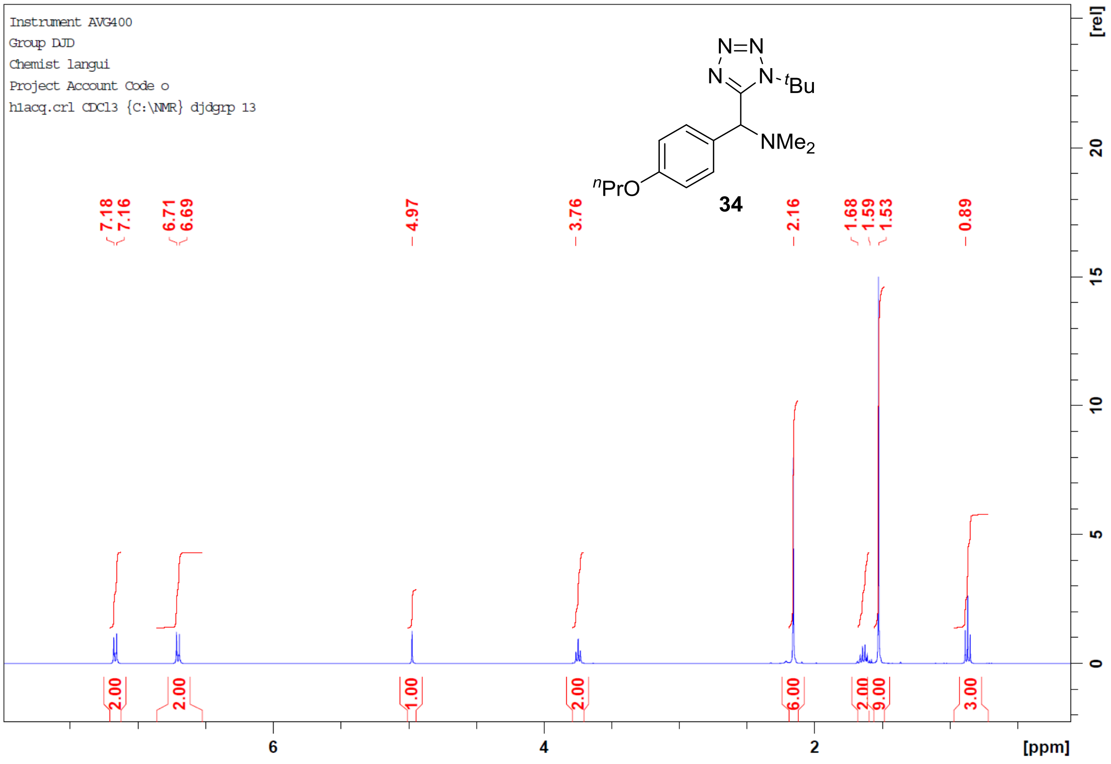

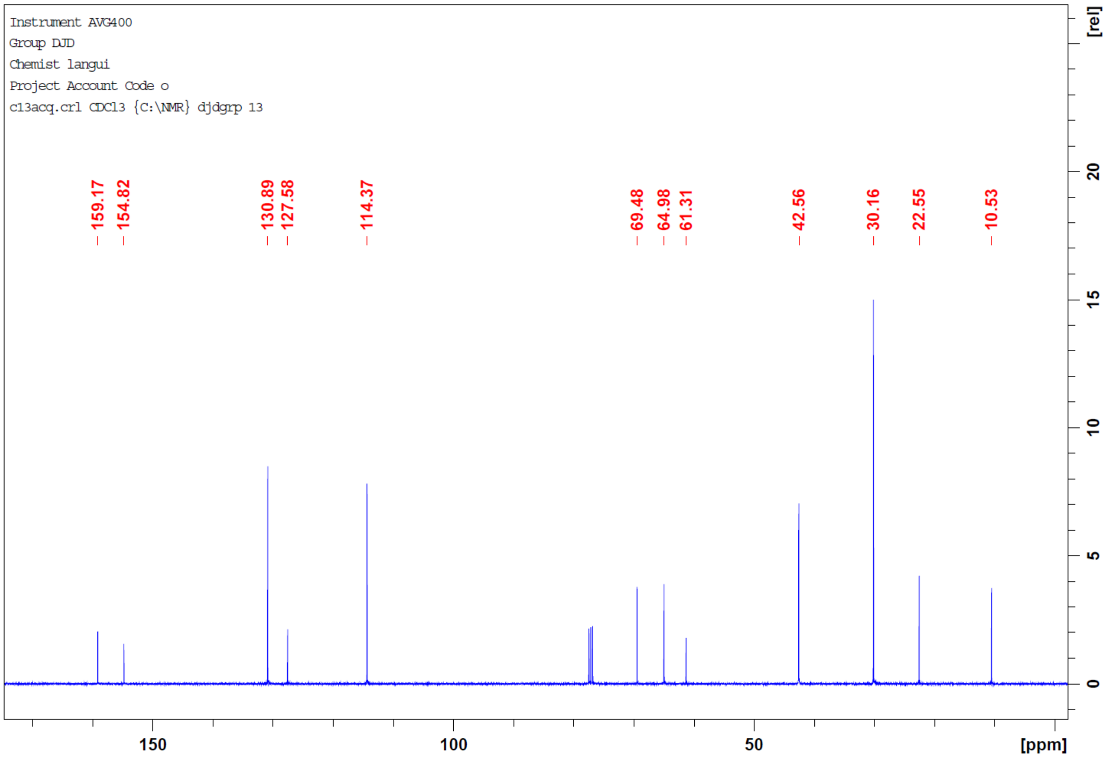

Supplementary Figure 33. ^{1}H NMR and ^{13}C NMR spectra of product **34**.

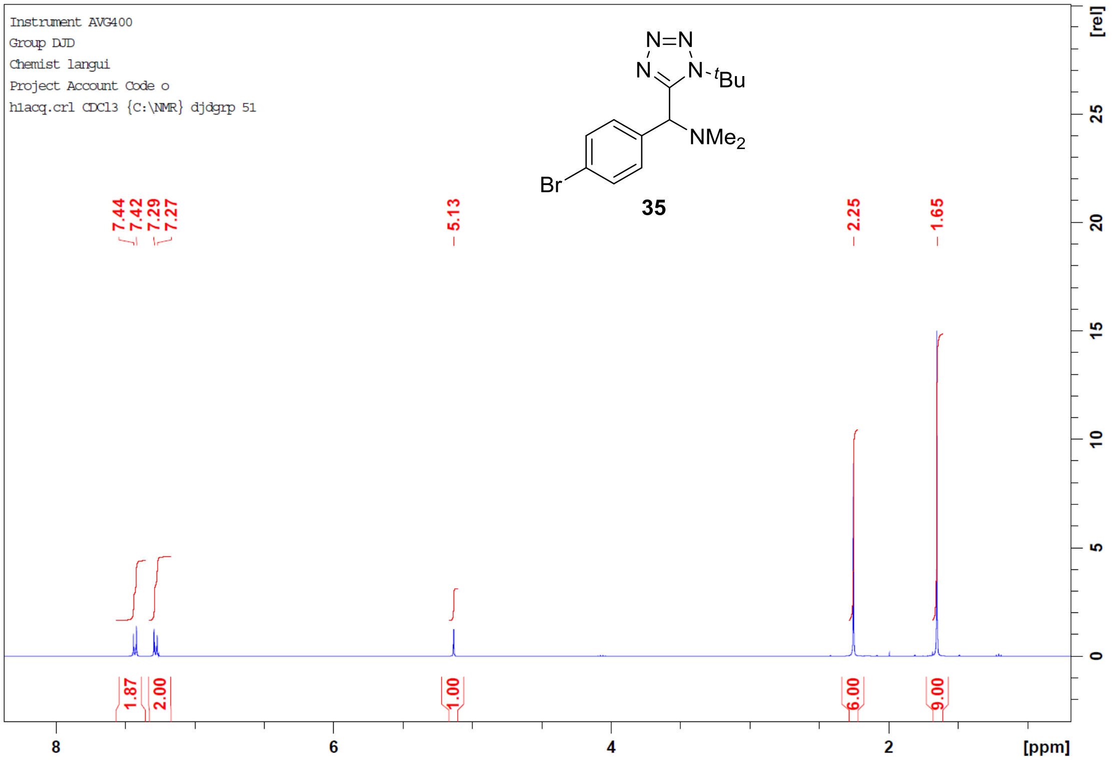

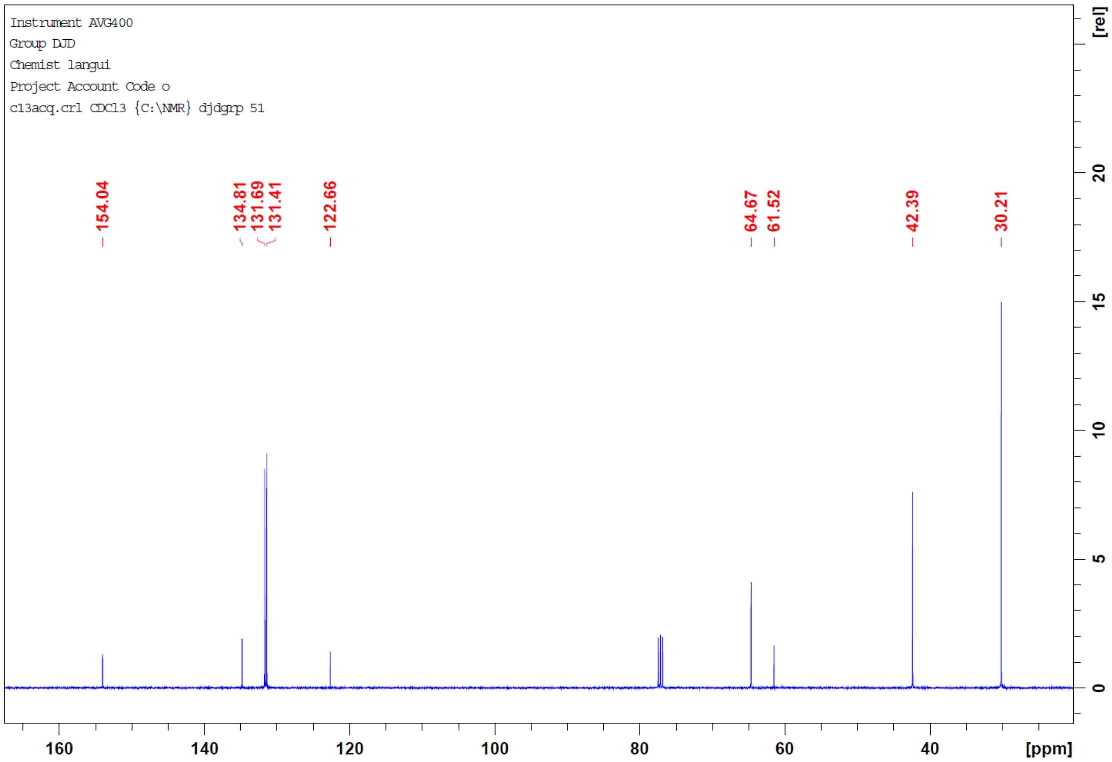

Supplementary Figure 34. ^{1}H NMR and ^{13}C NMR spectra of product **35**.

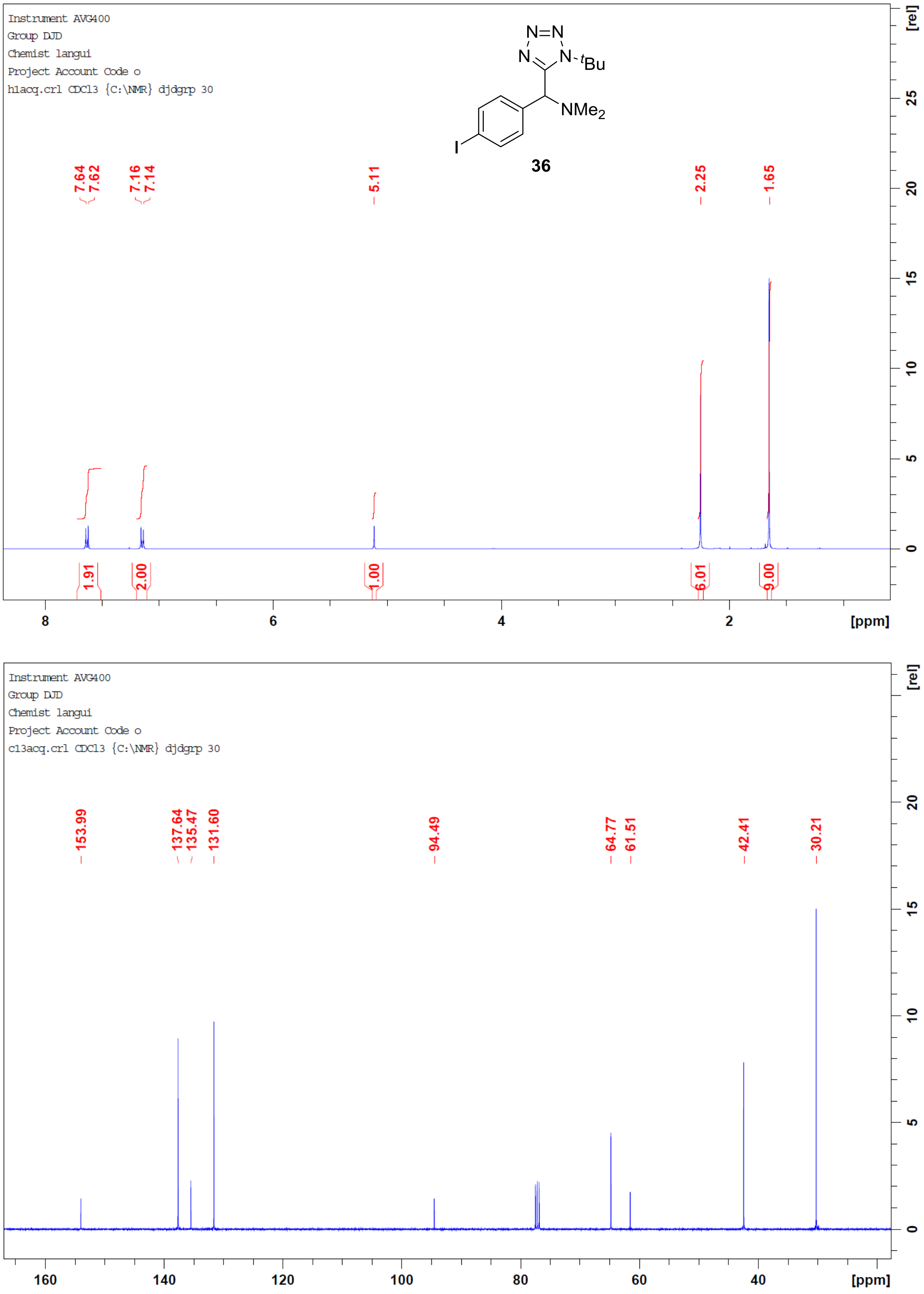

Supplementary Figure 35. ^{1}H NMR and ^{13}C NMR spectra of product **36**.

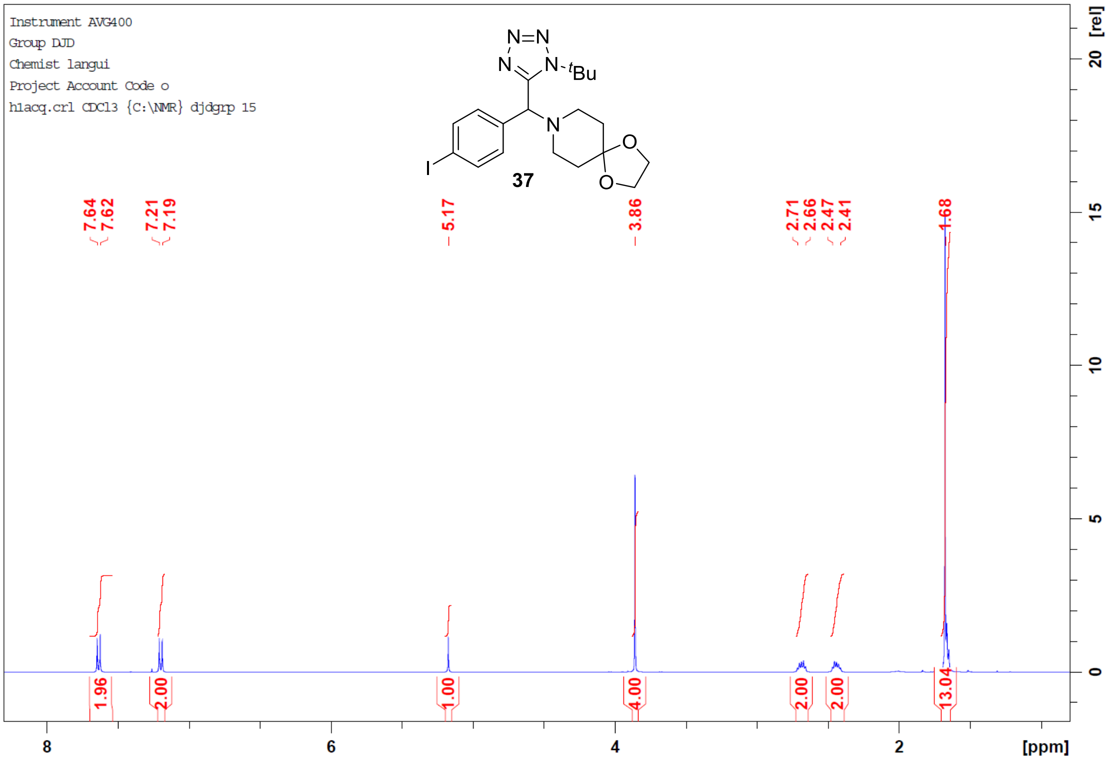

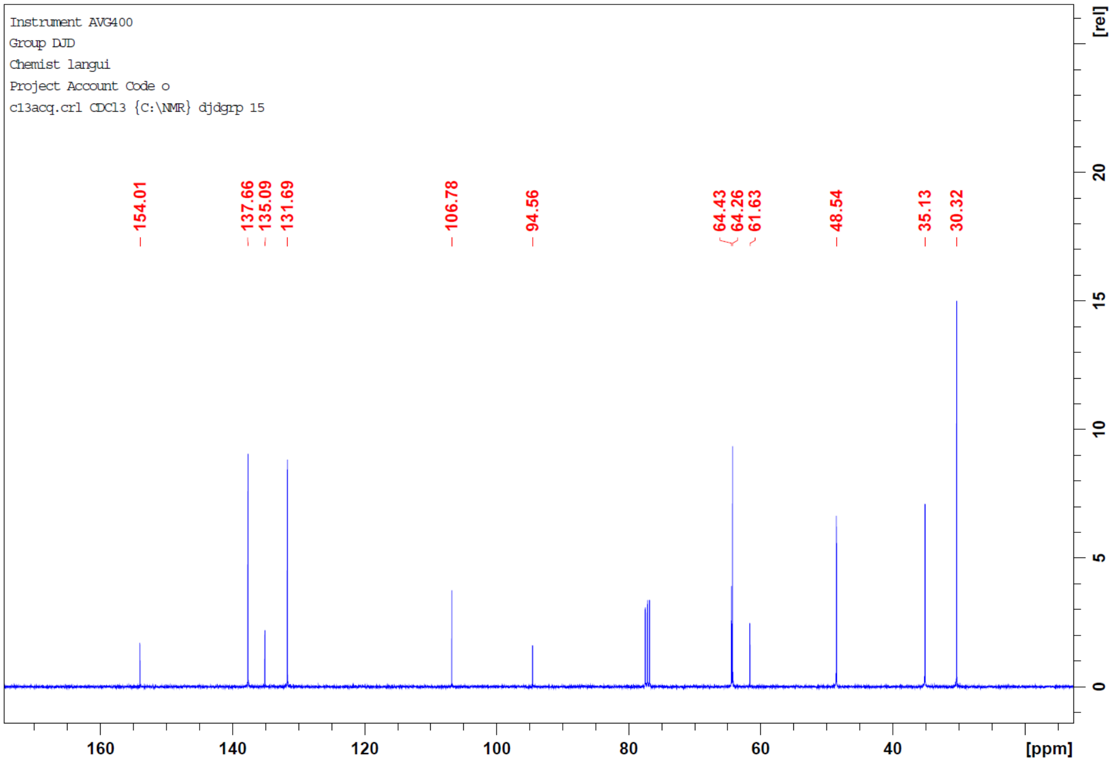

Supplementary Figure 36. ^{1}H NMR and ^{13}C NMR spectra of product **37**.

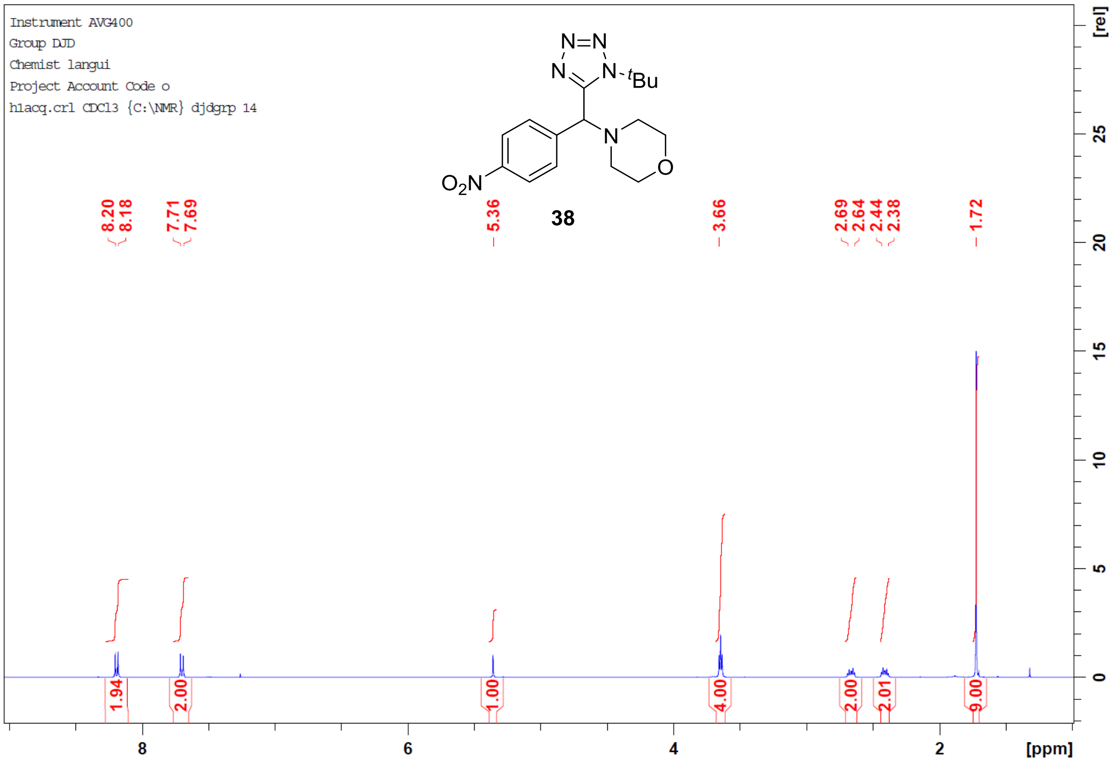

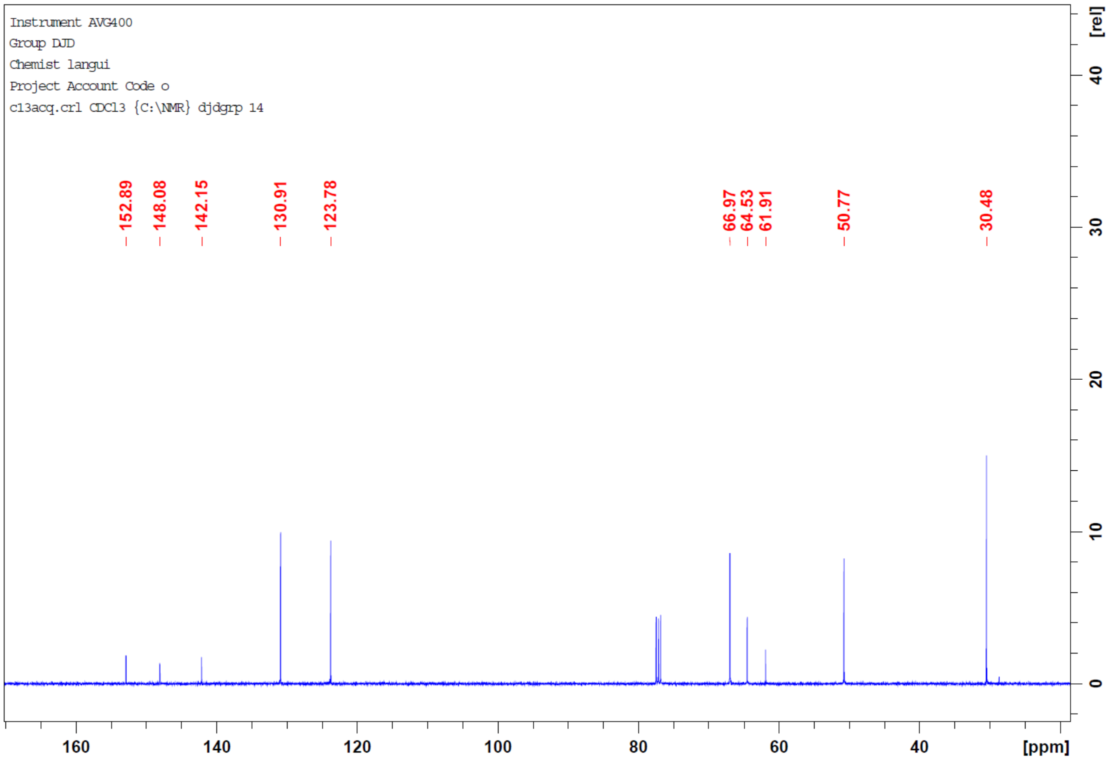

Supplementary Figure 37. ^{1}H NMR and ^{13}C NMR spectra of product **38**.

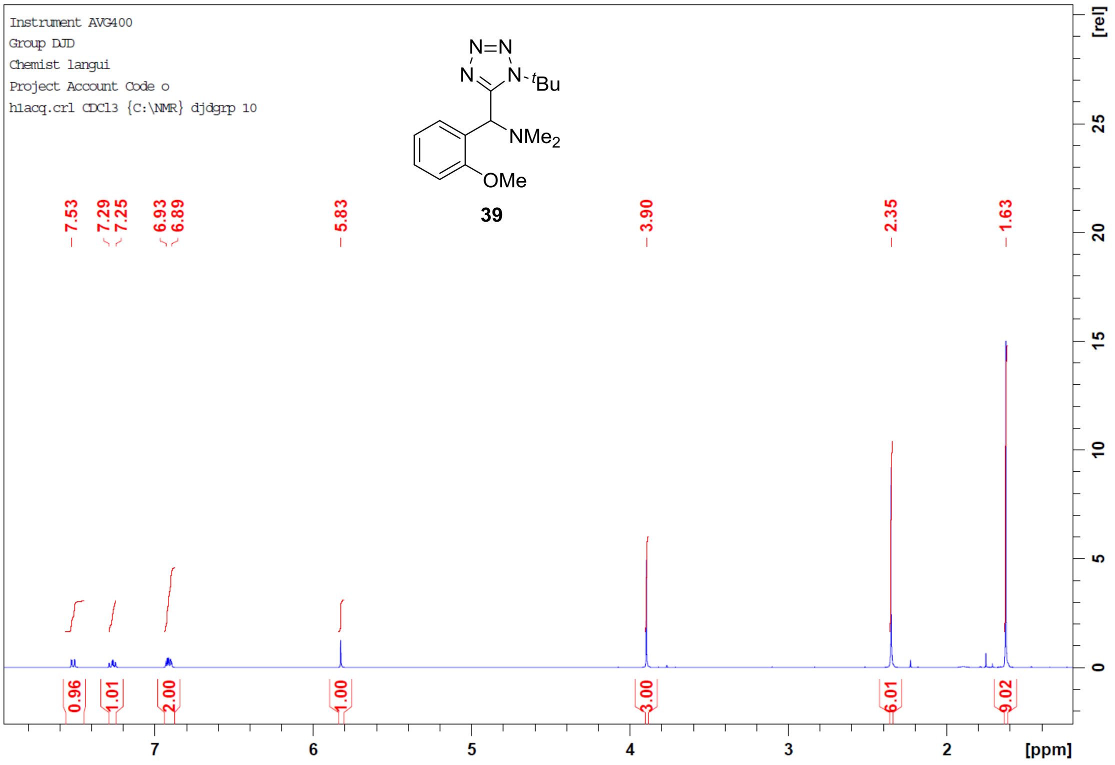

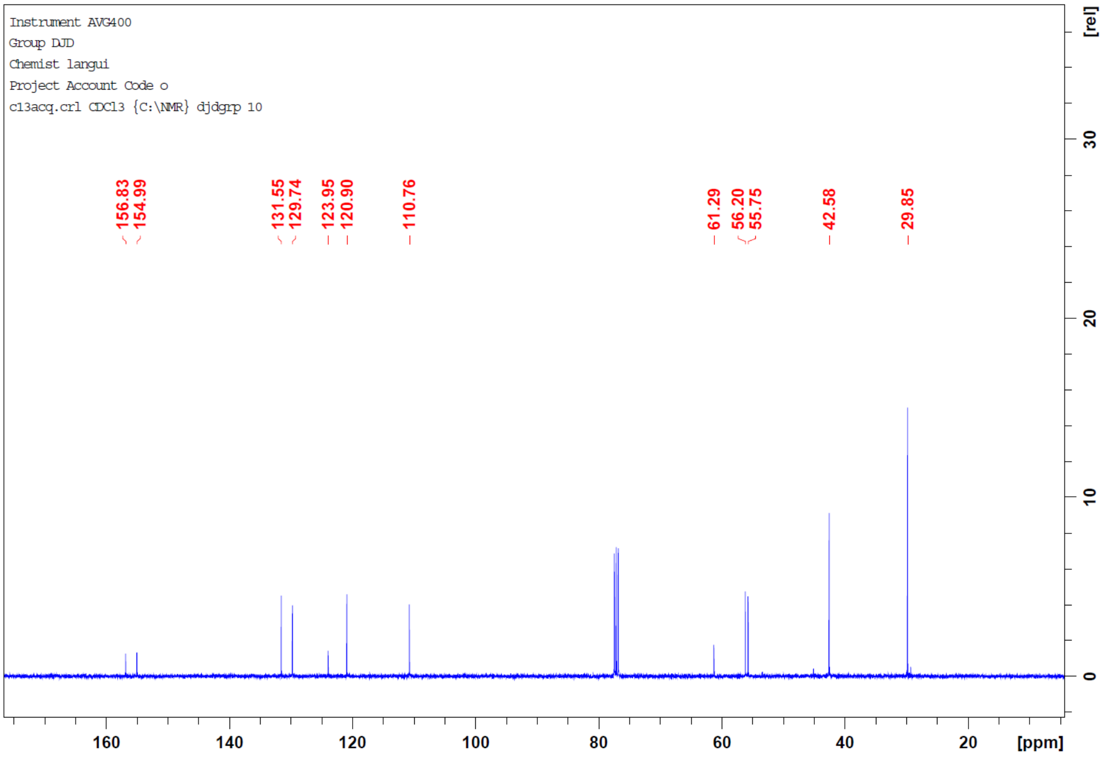

Supplementary Figure 38. ^{1}H NMR and ^{13}C NMR spectra of product **39**.

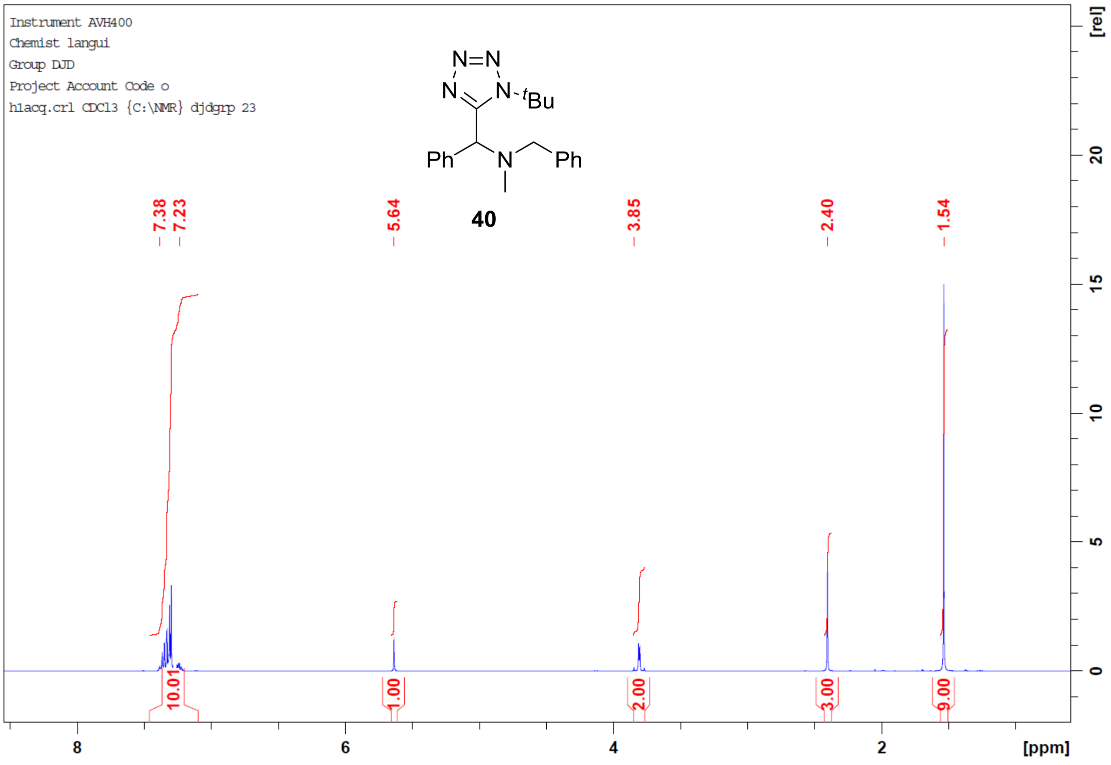

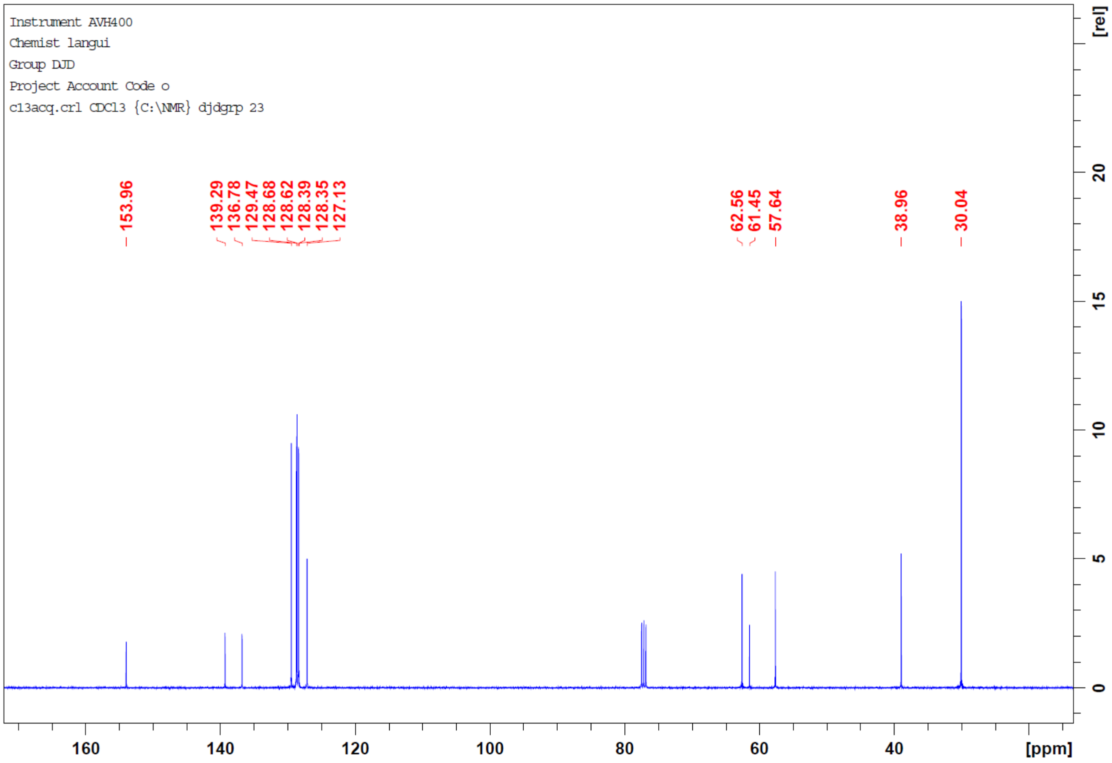

Supplementary Figure 39. ^{1}H NMR and ^{13}C NMR spectra of product **40**.

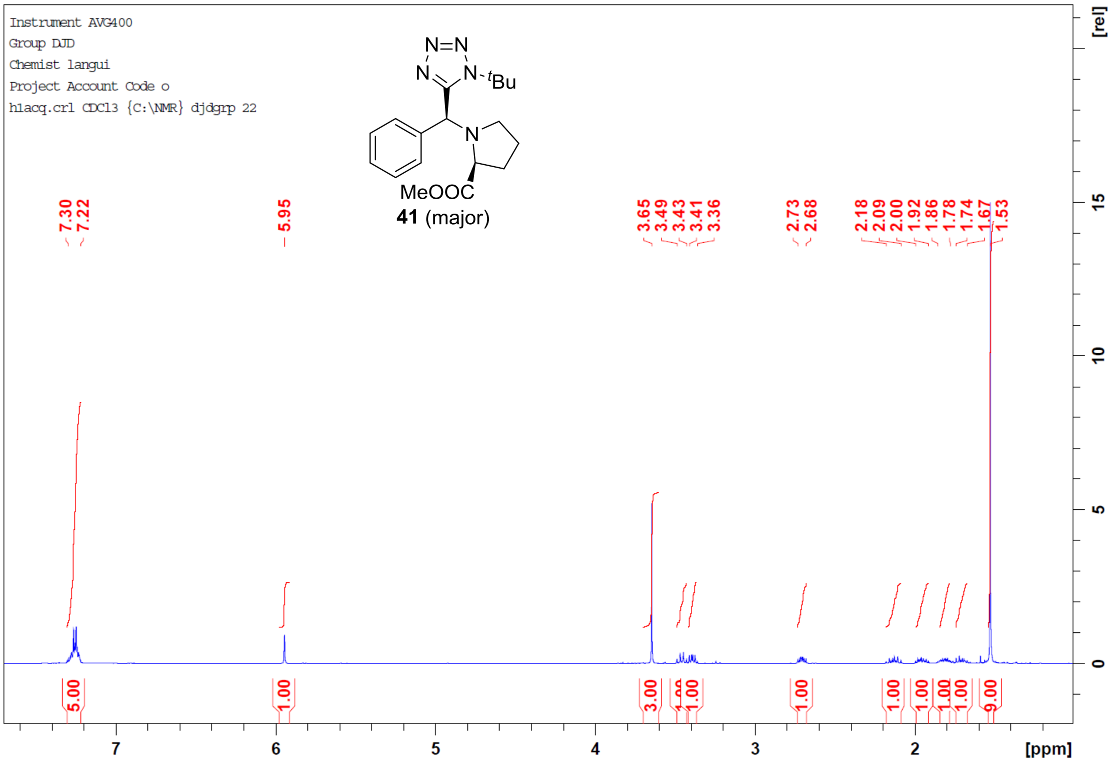

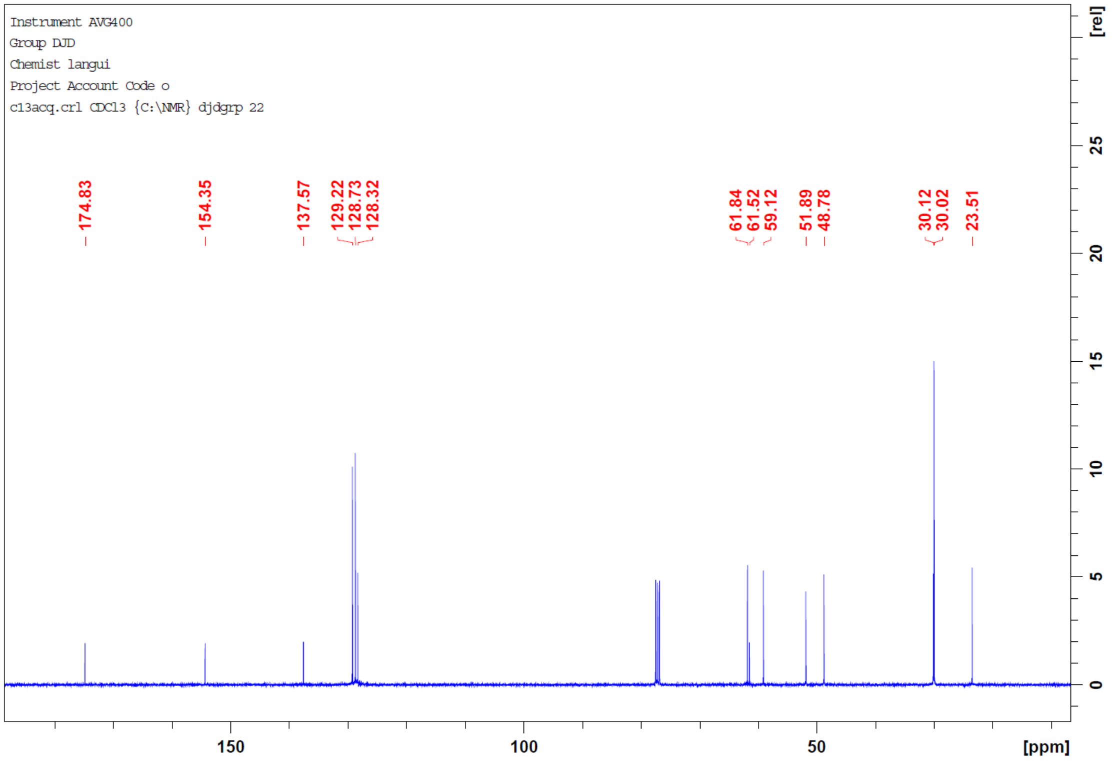

Supplementary Figure 40. ^{1}H NMR and ^{13}C NMR spectra of product **41** (major).

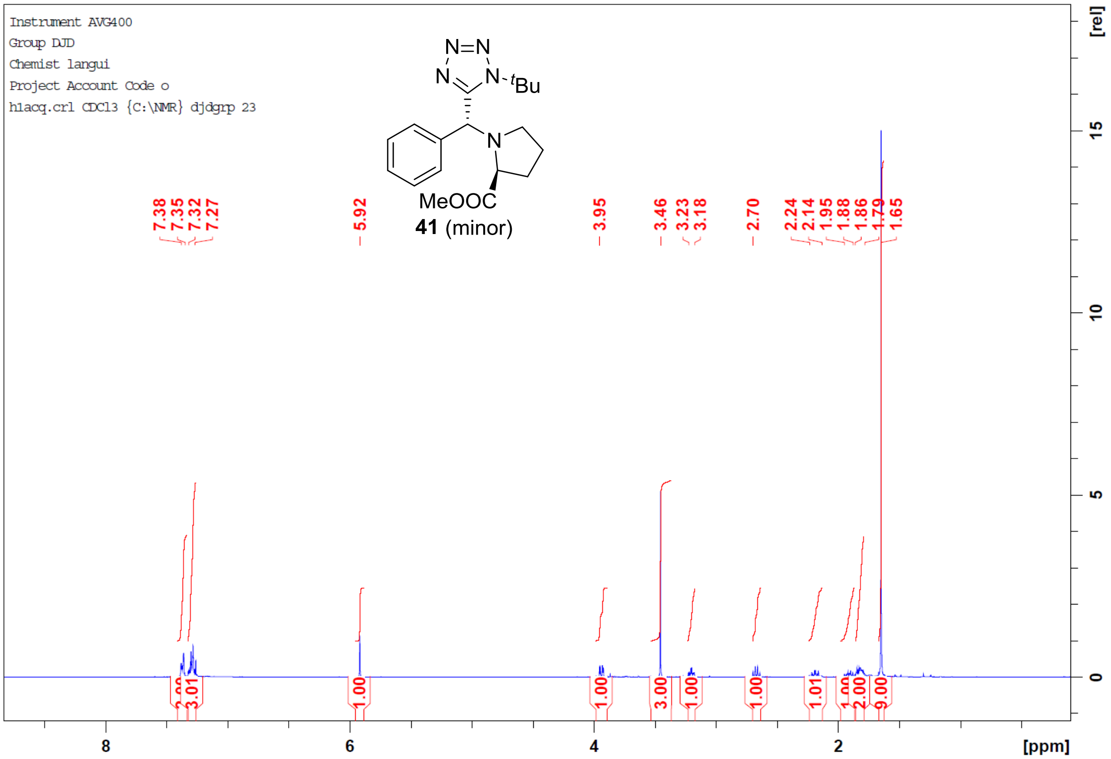

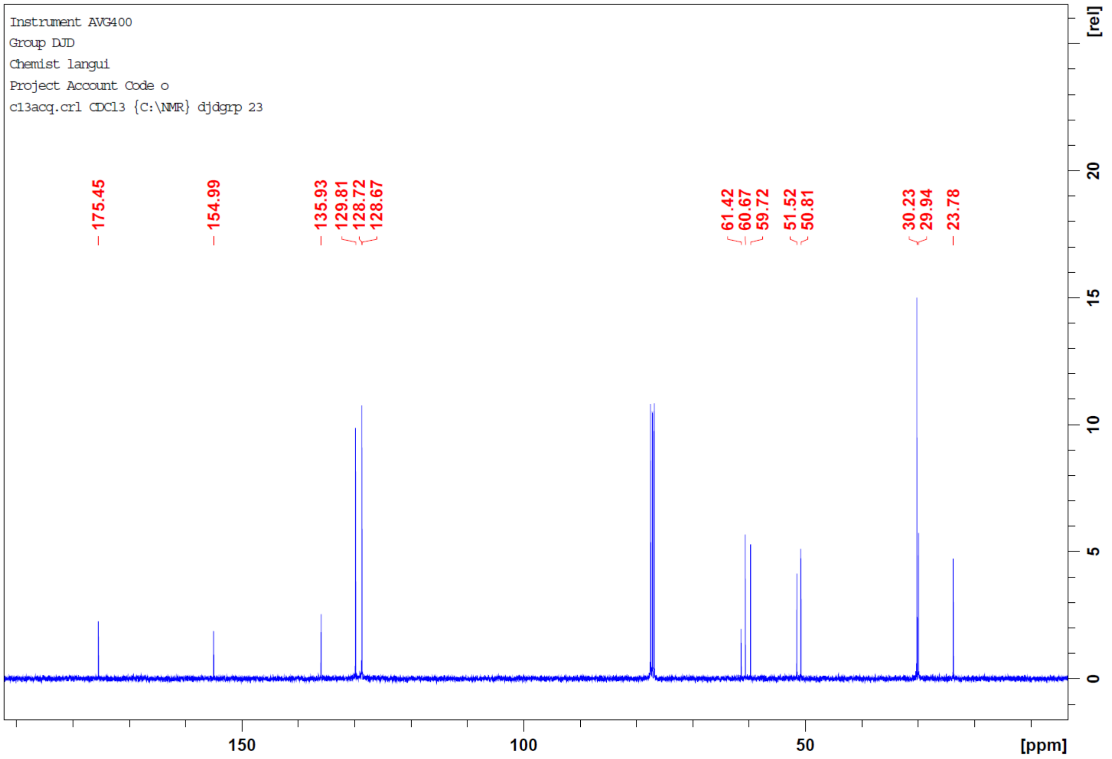

Supplementary Figure 41. ^{1}H NMR and ^{13}C NMR spectra of product **41** (minor).

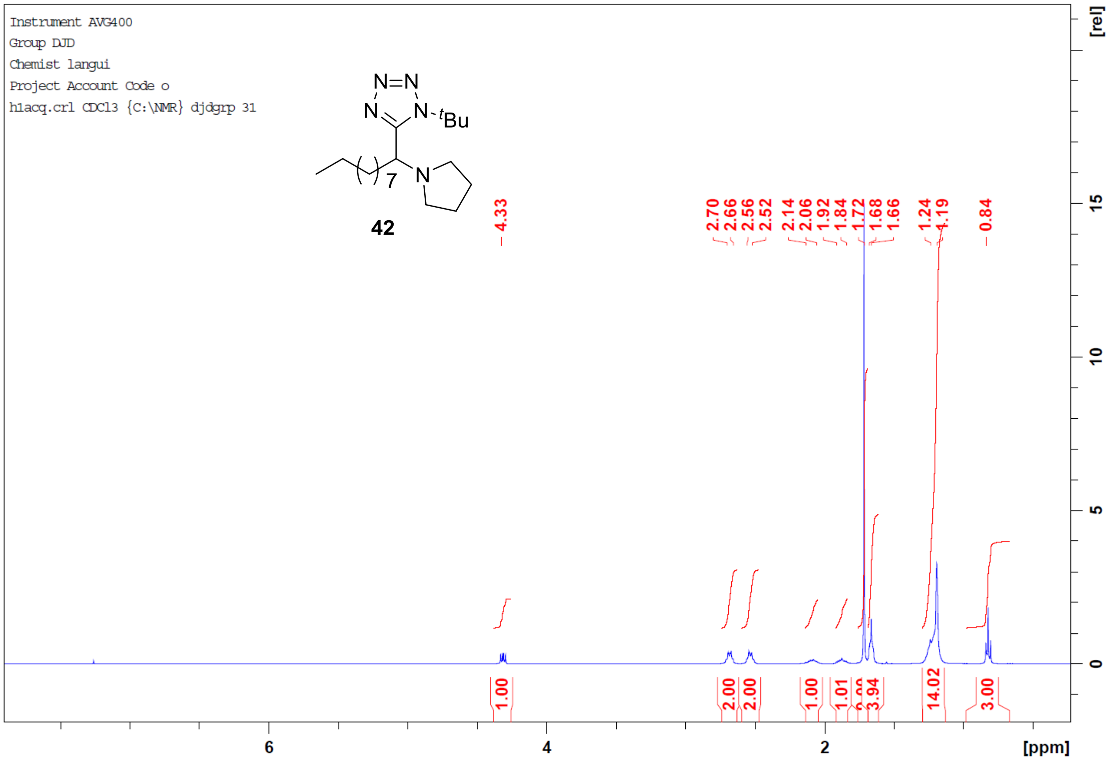

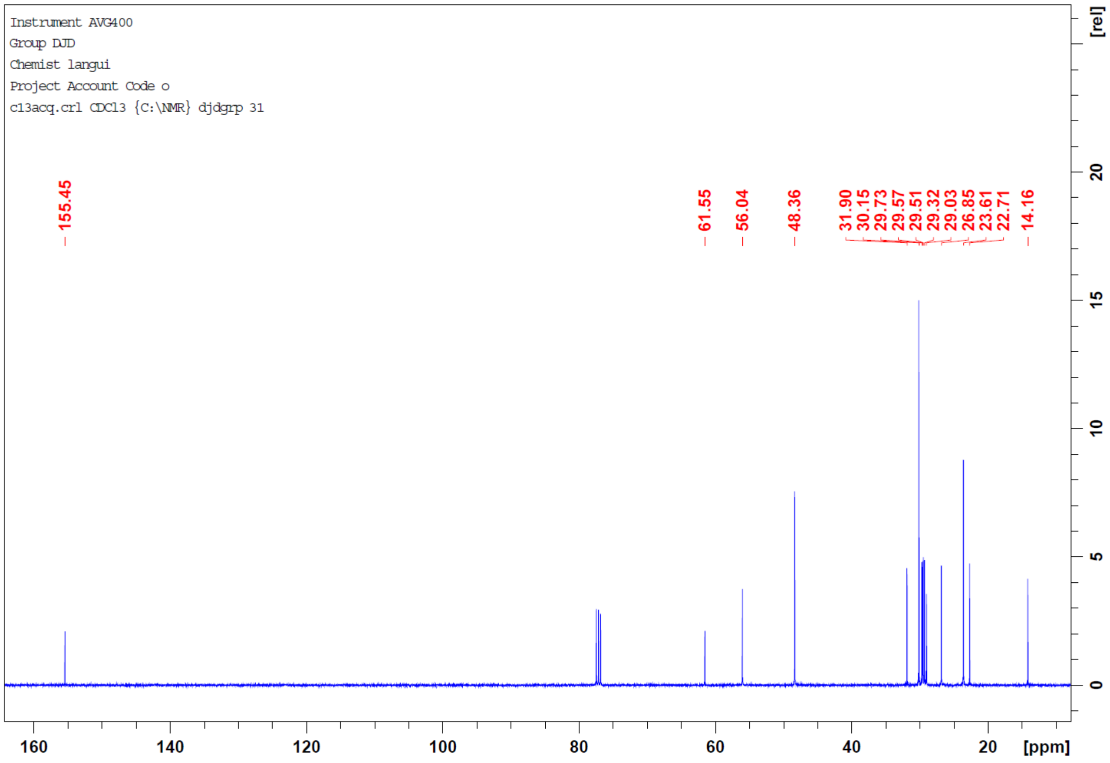

Supplementary Figure 42. ^{1}H NMR and ^{13}C NMR spectra of product **42**.

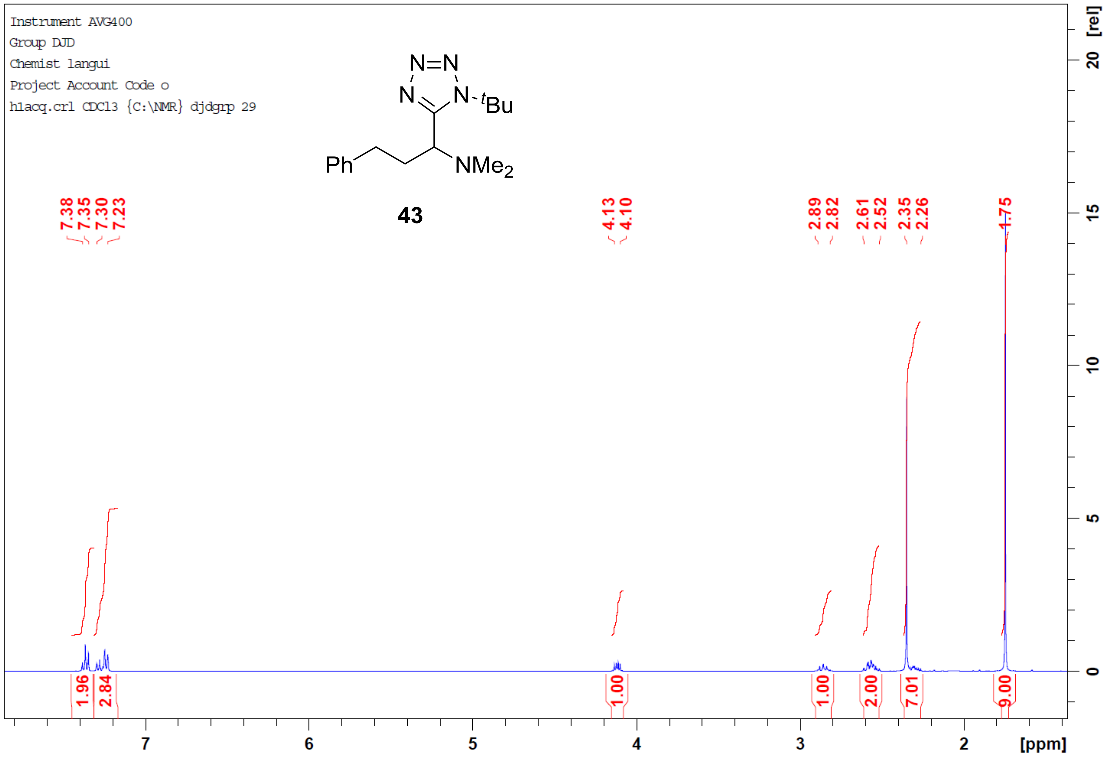

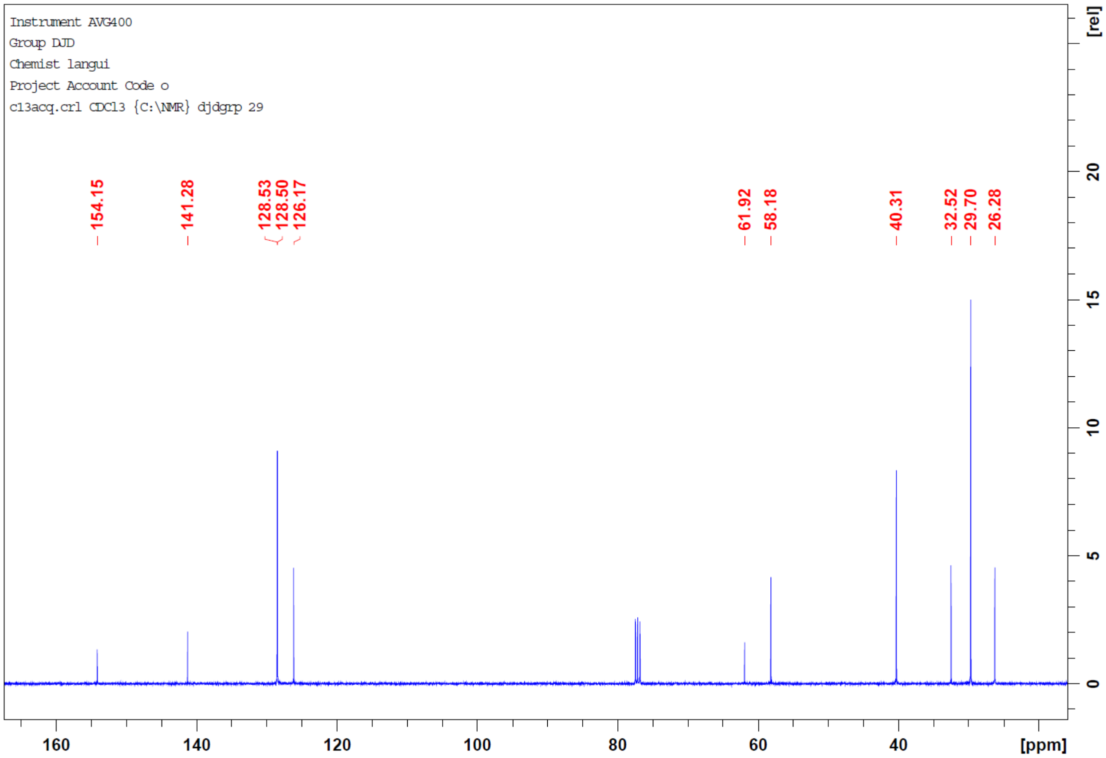

Supplementary Figure 43. ^{1}H NMR and ^{13}C NMR spectra of product **43**.

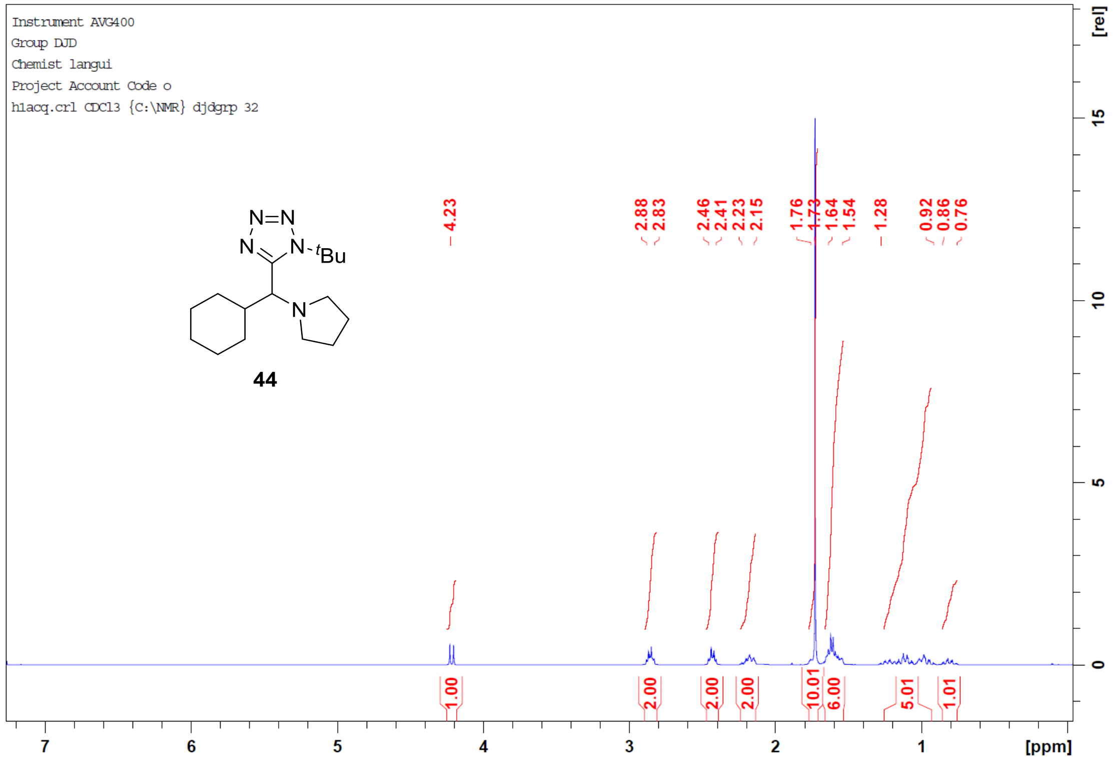

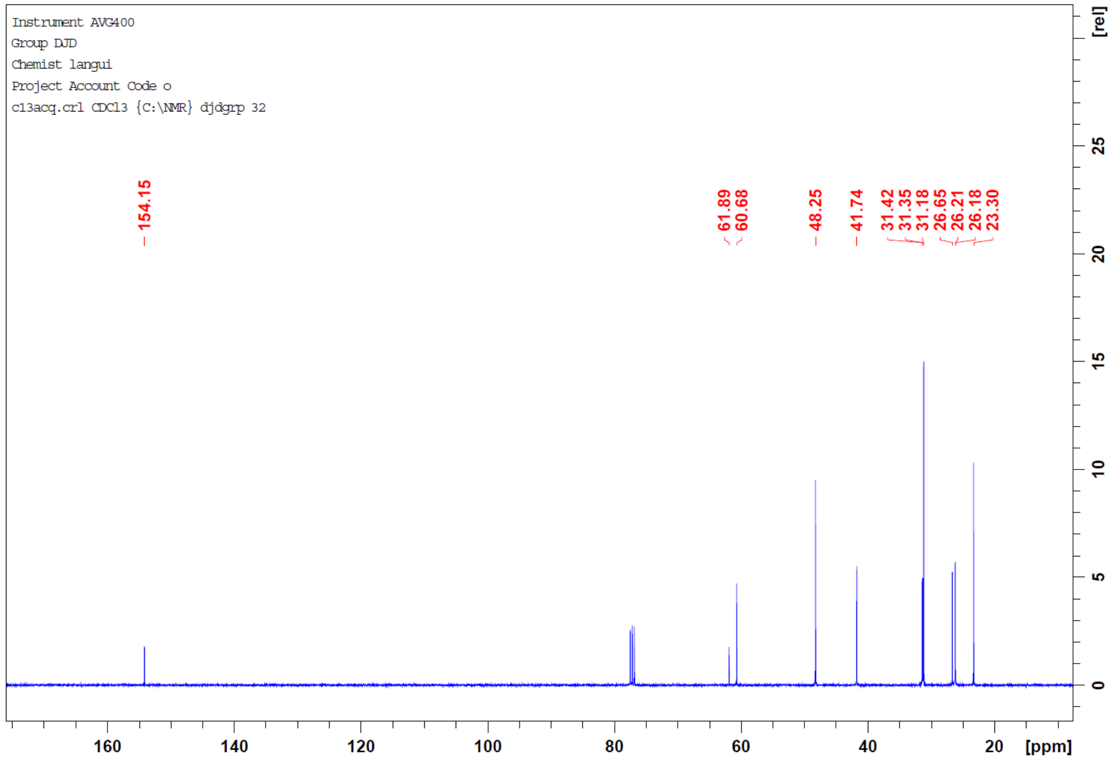

Supplementary Figure 44. ^{1}H NMR and ^{13}C NMR spectra of product **44**.

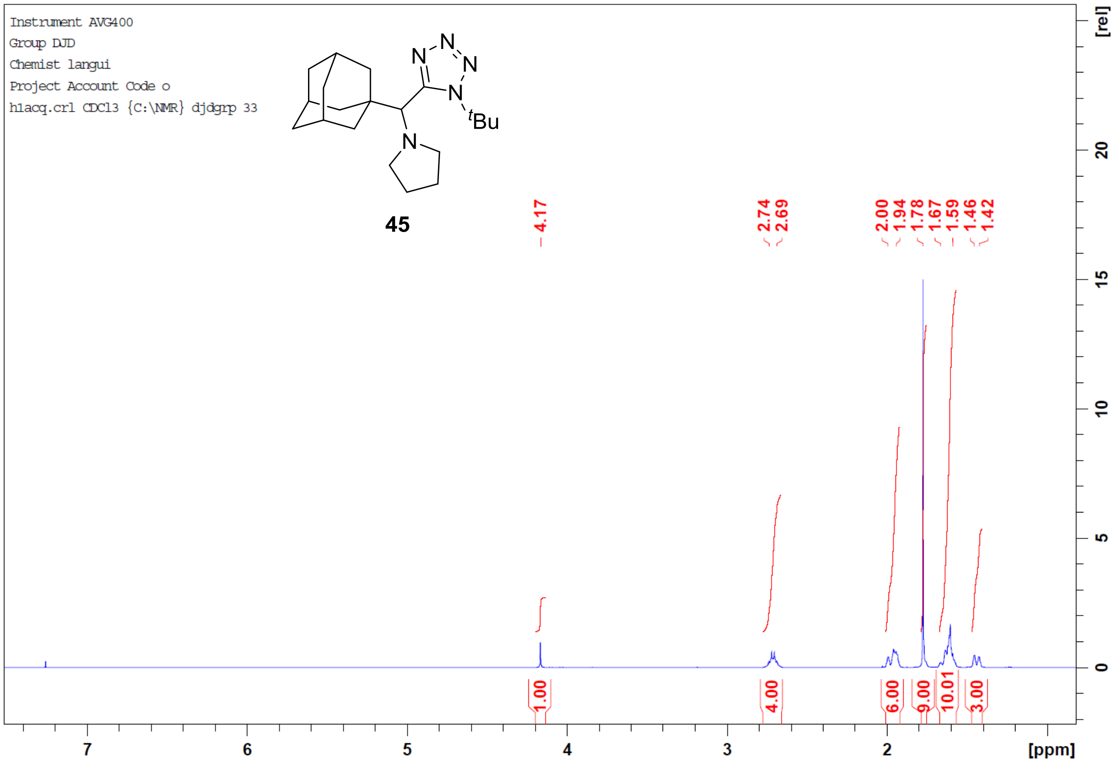

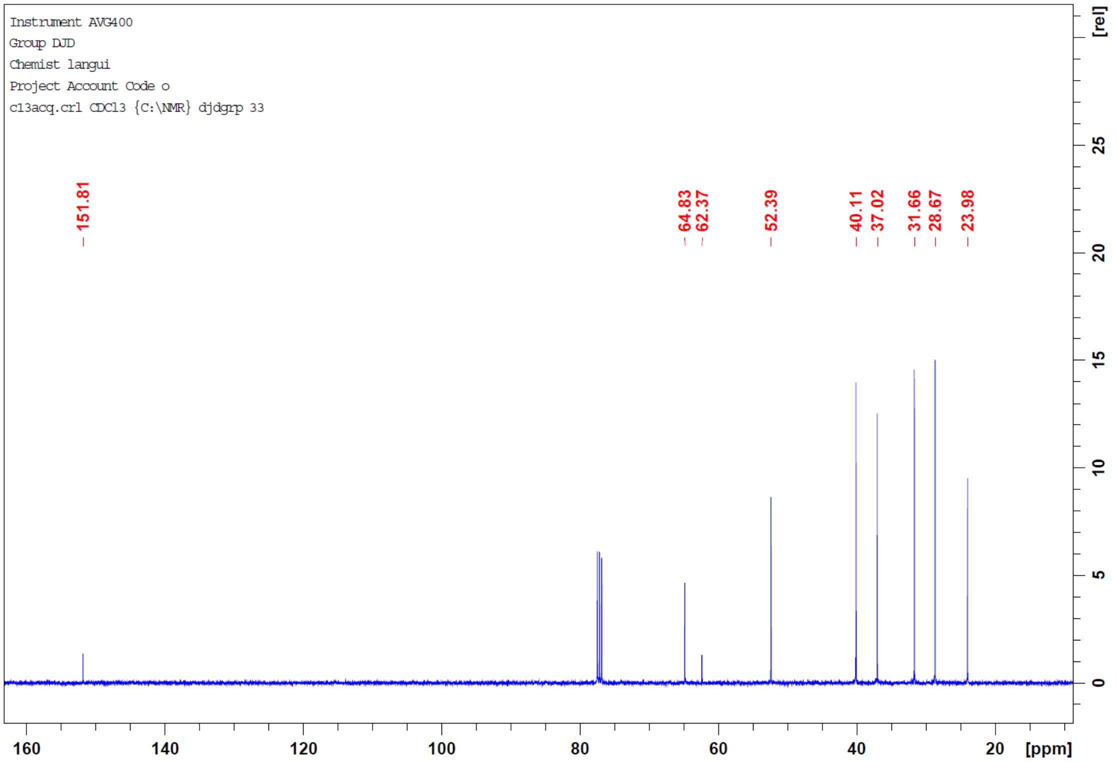

Supplementary Figure 45. ^{1}H NMR and ^{13}C NMR spectra of product **45**.

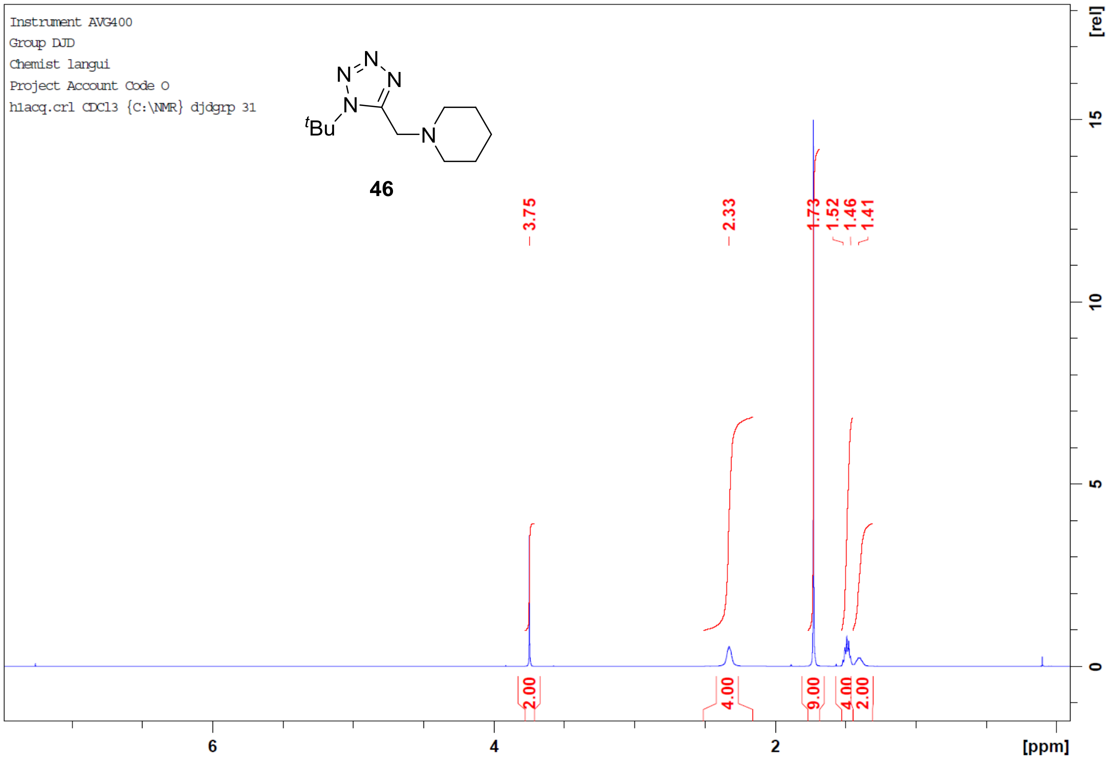

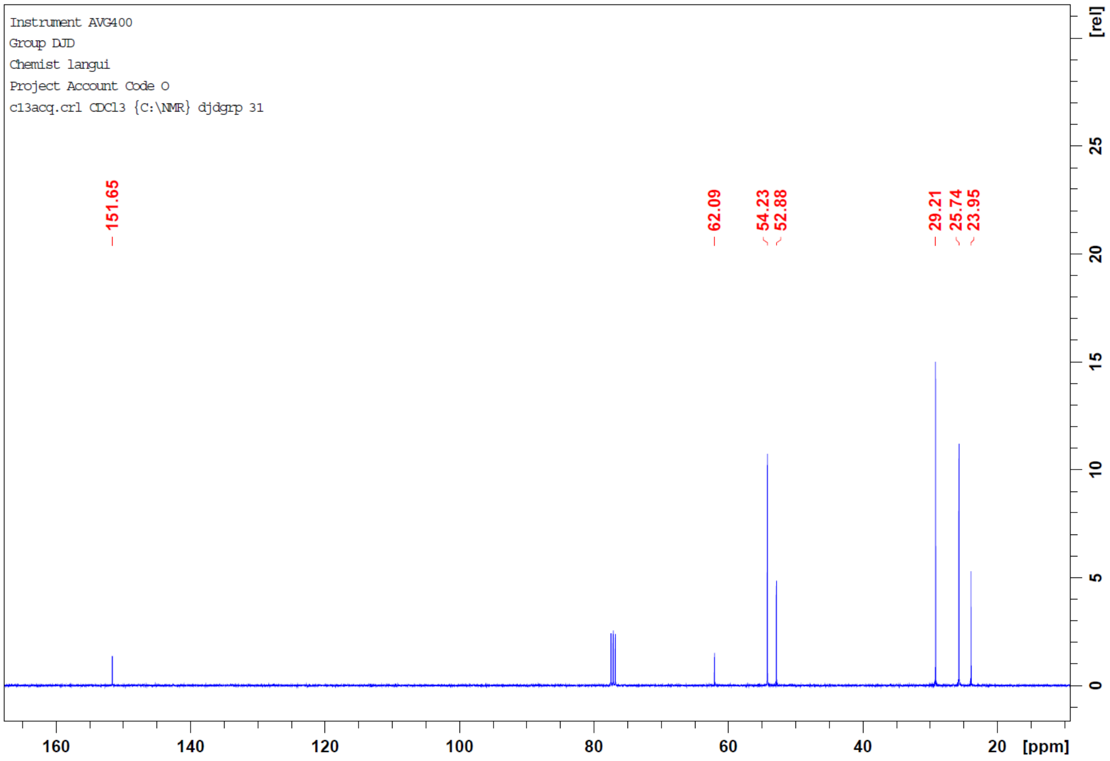

Supplementary Figure 46. ^{1}H NMR and ^{13}C NMR spectra of product **46**.

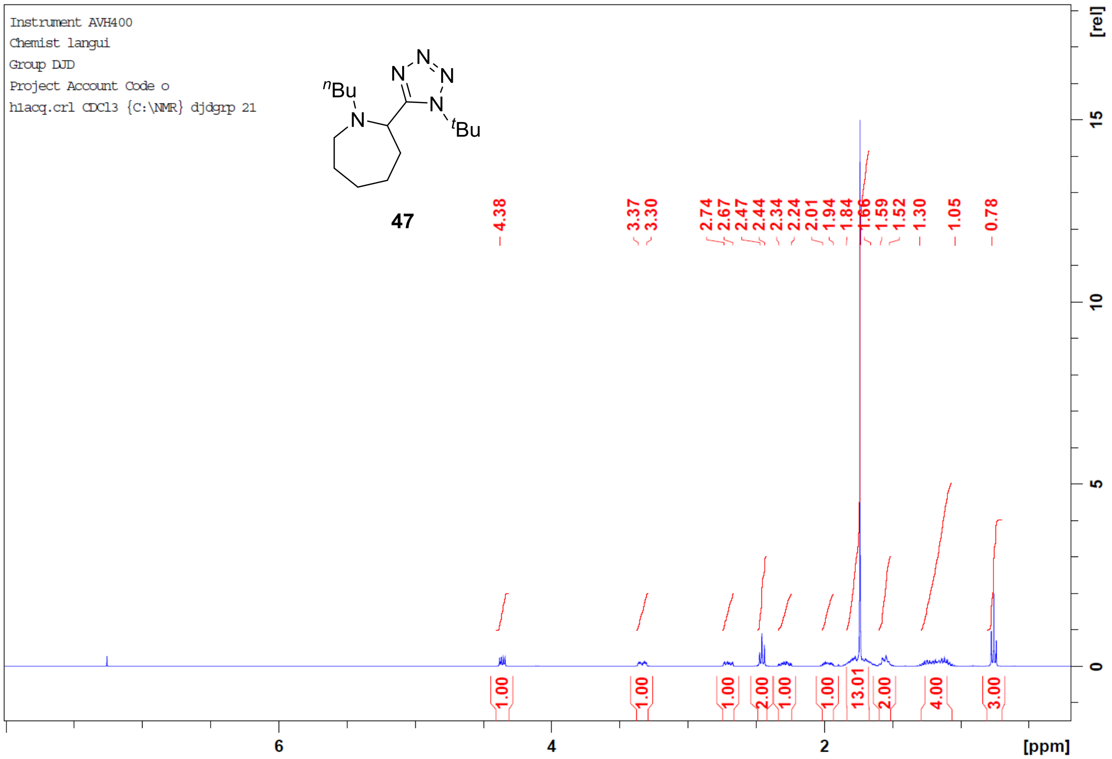

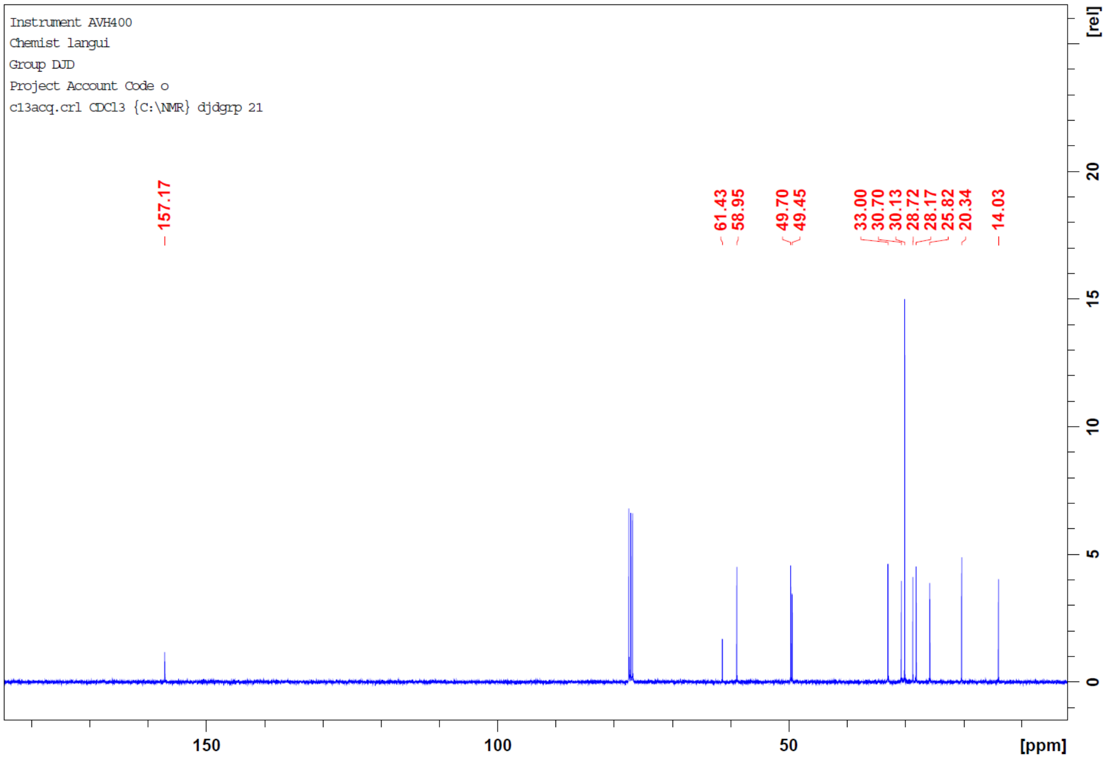

Supplementary Figure 47. ^{1}H NMR and ^{13}C NMR spectra of product **47**.

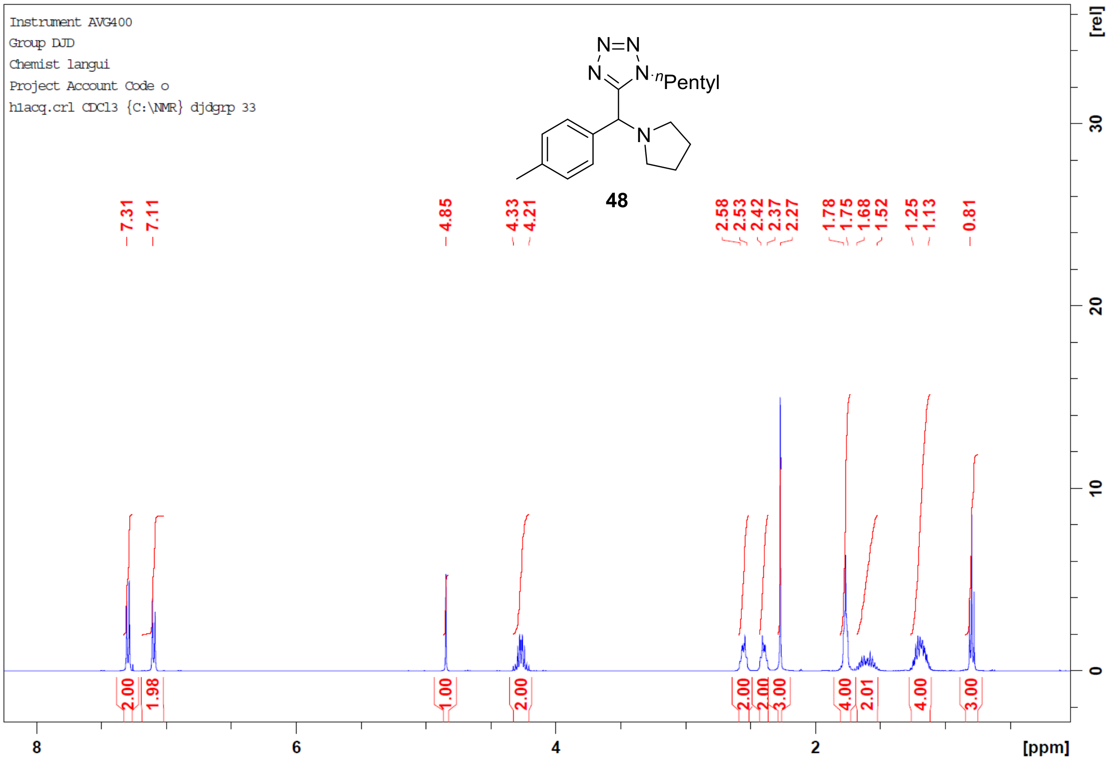

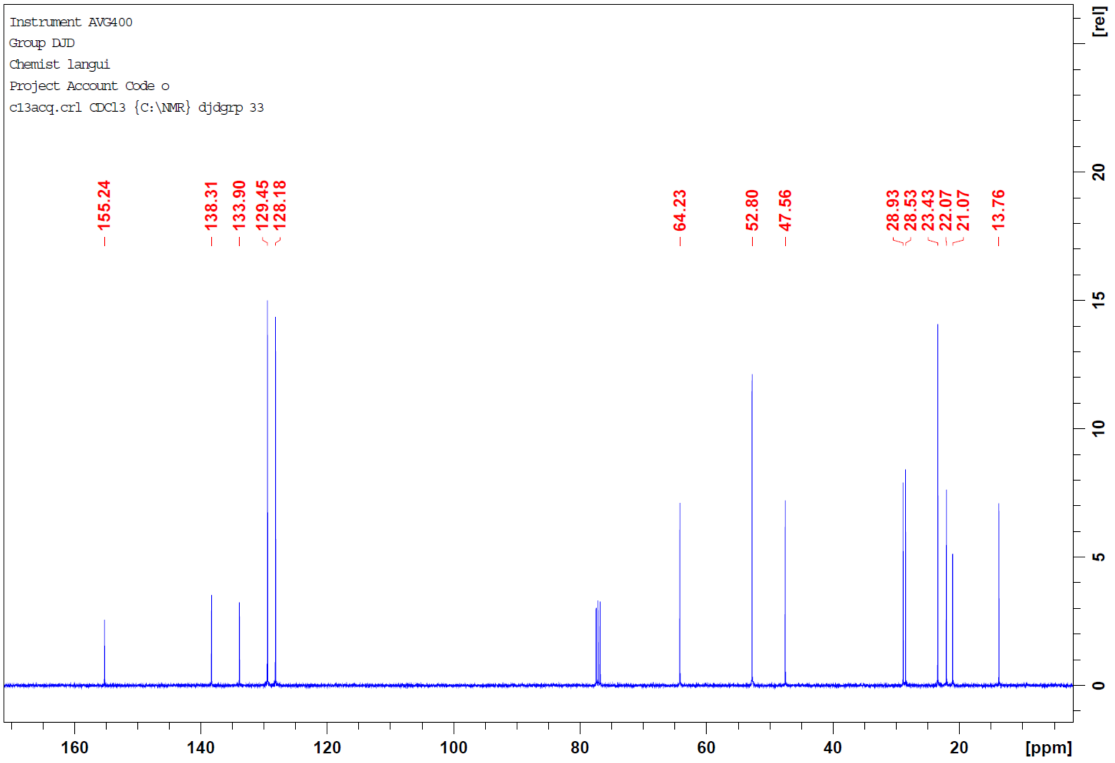

Supplementary Figure 48. ^{1}H NMR and ^{13}C NMR spectra of product **48**.

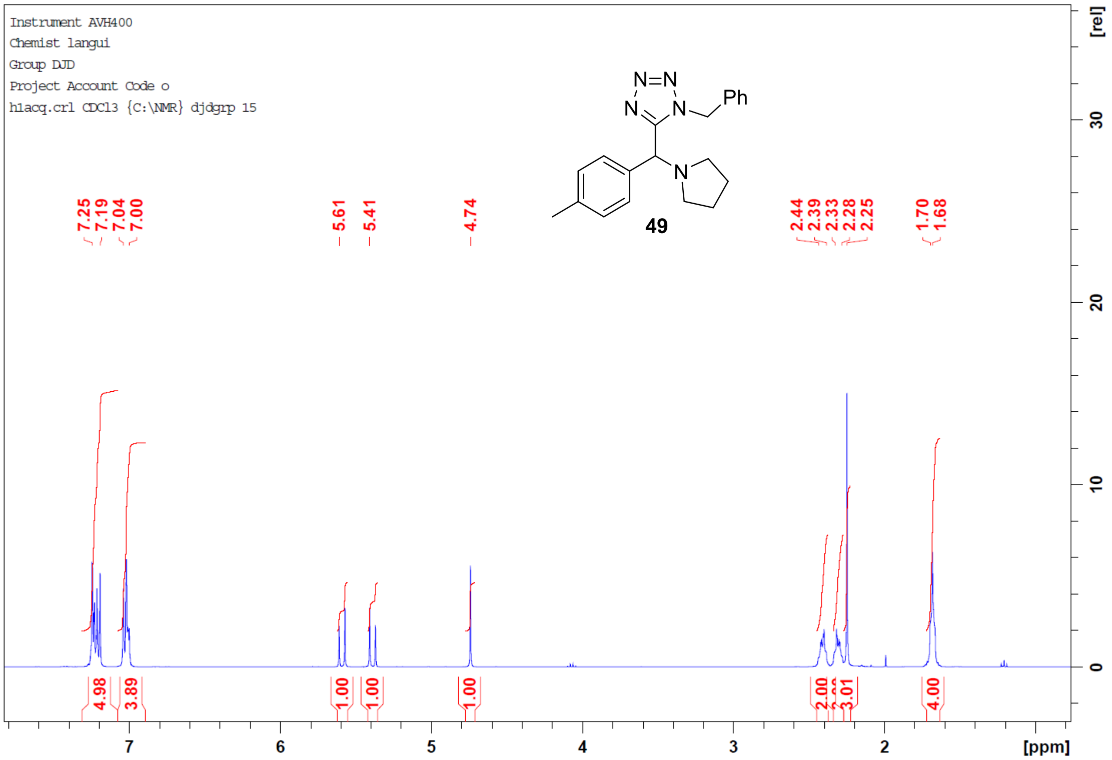

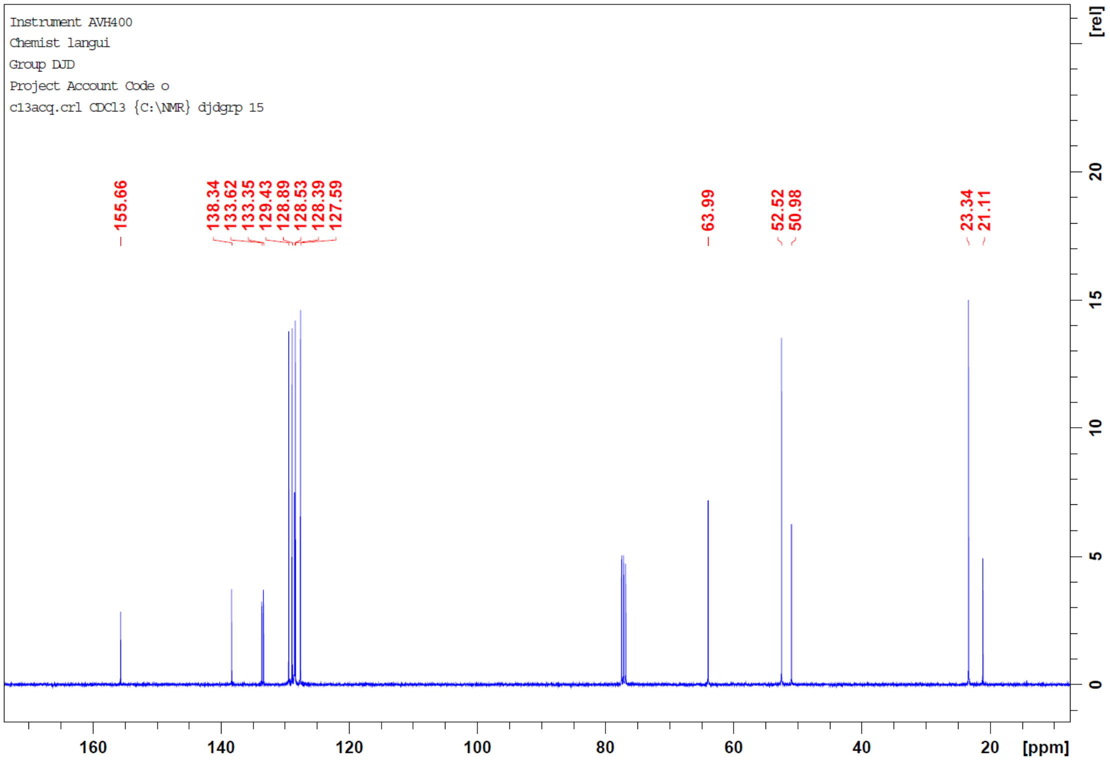

Supplementary Figure 49. ^{1}H NMR and ^{13}C NMR spectra of product **49**.

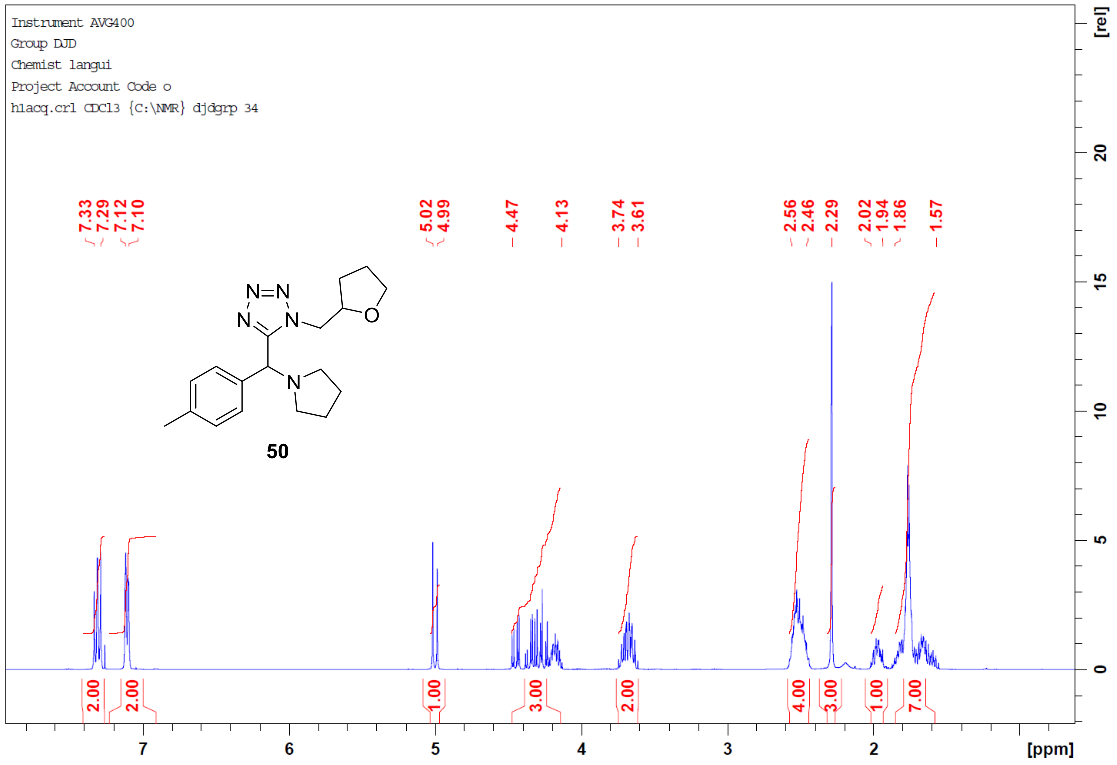

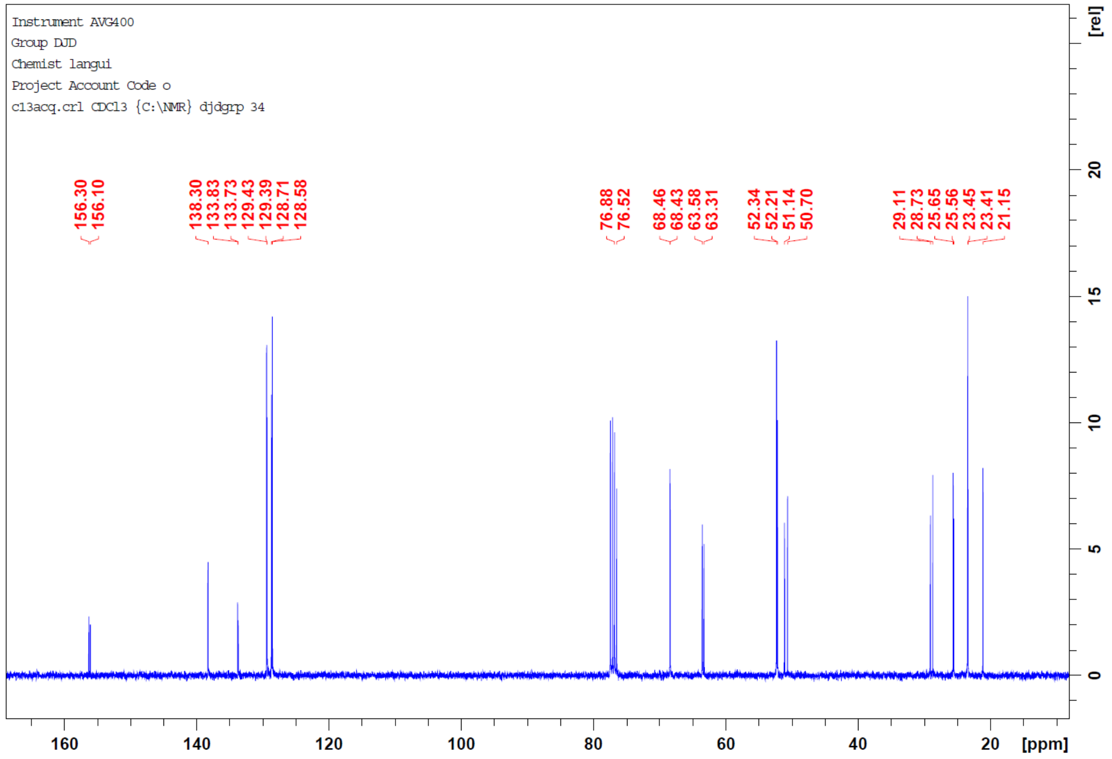

Supplementary Figure 50. ^{1}H NMR and ^{13}C NMR spectra of product **50**.

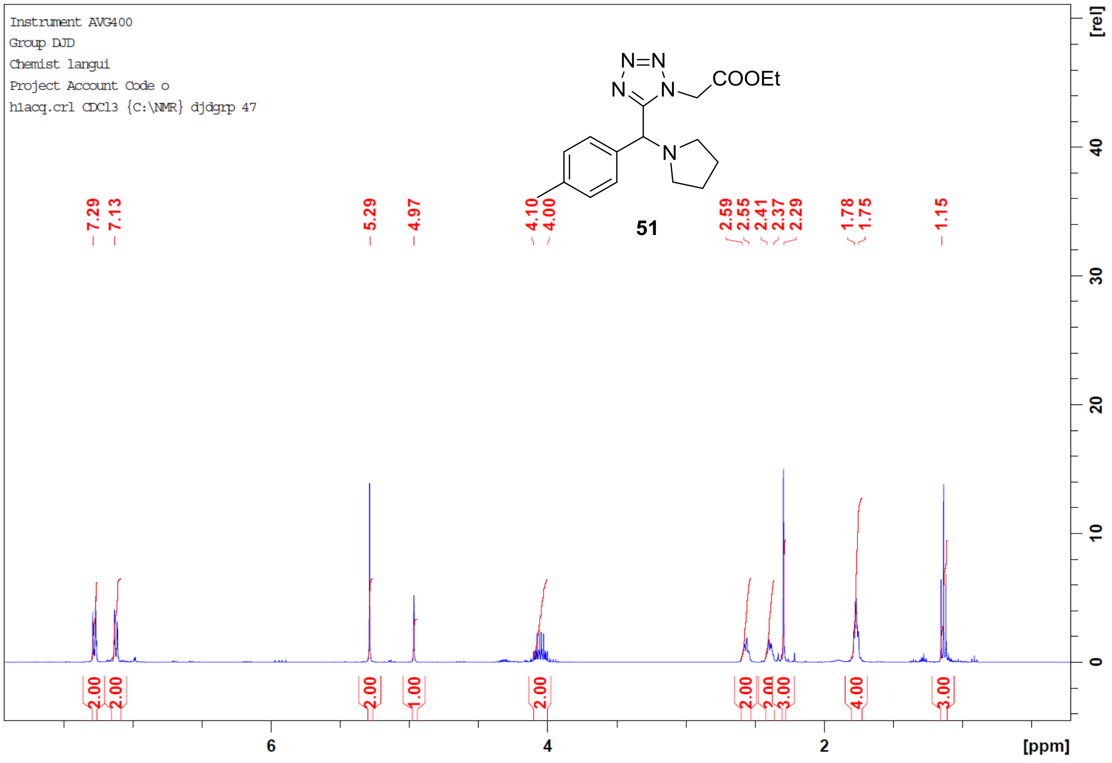

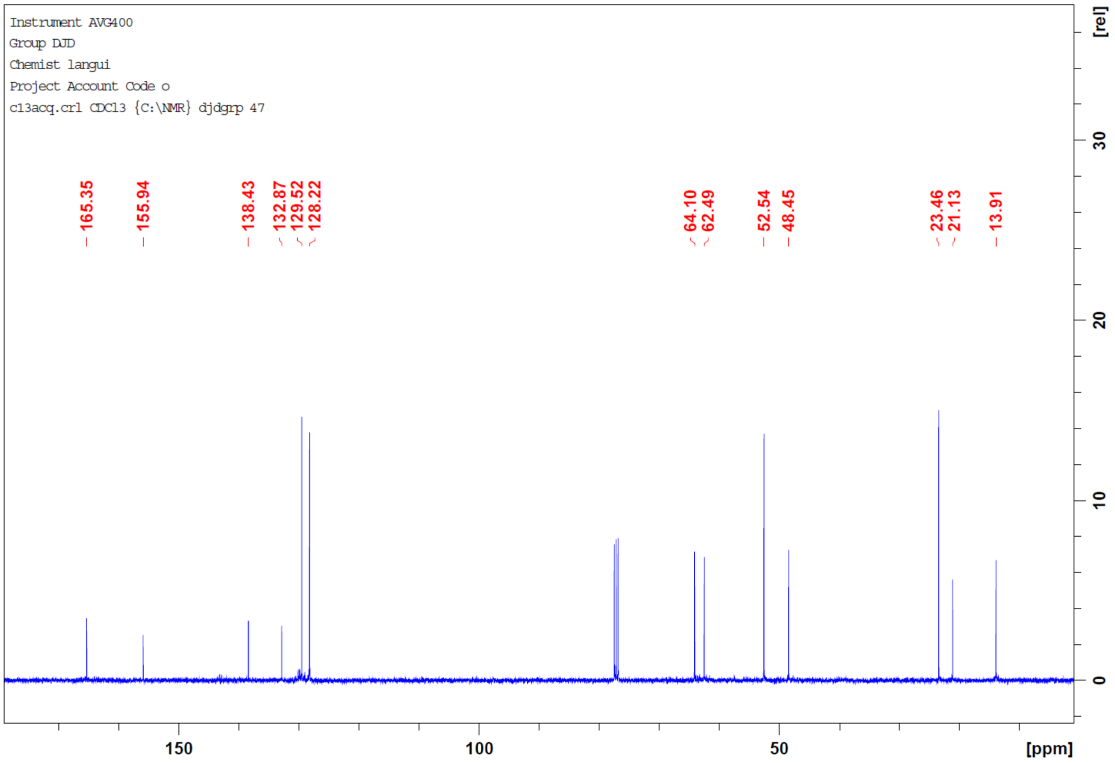

Supplementary Figure 51. ^{1}H NMR and ^{13}C NMR spectra of product **51**.

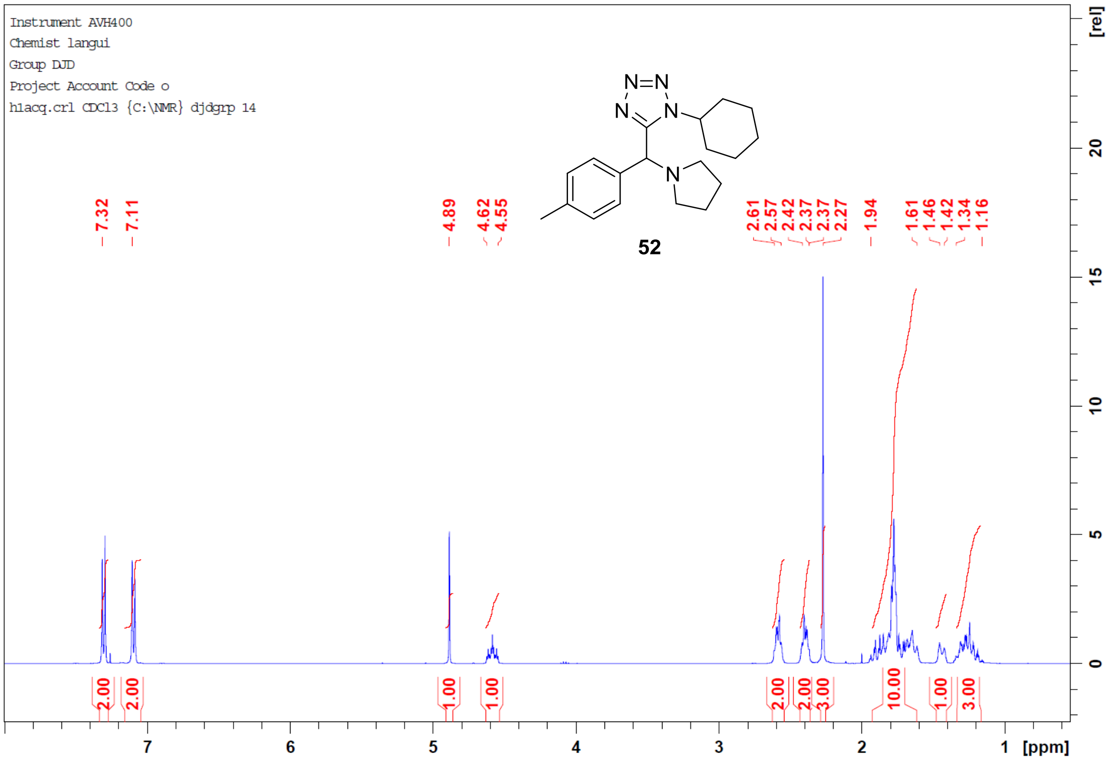

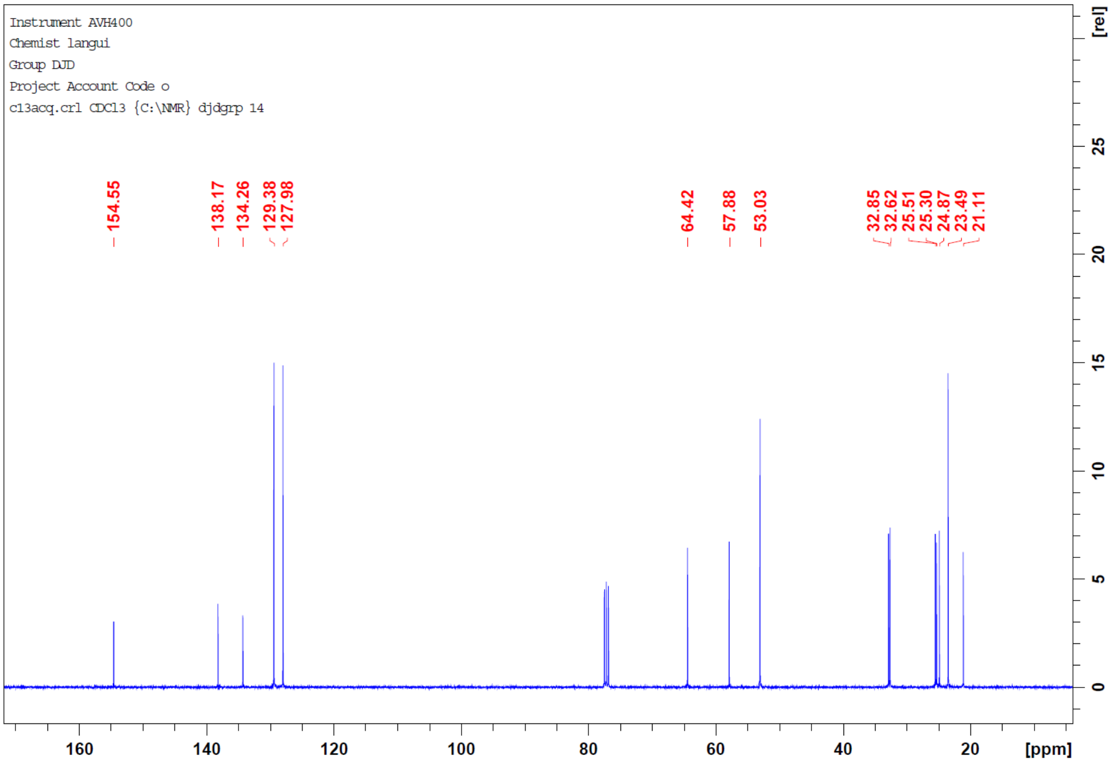

Supplementary Figure 52. ^{1}H NMR and ^{13}C NMR spectra of product **52**.

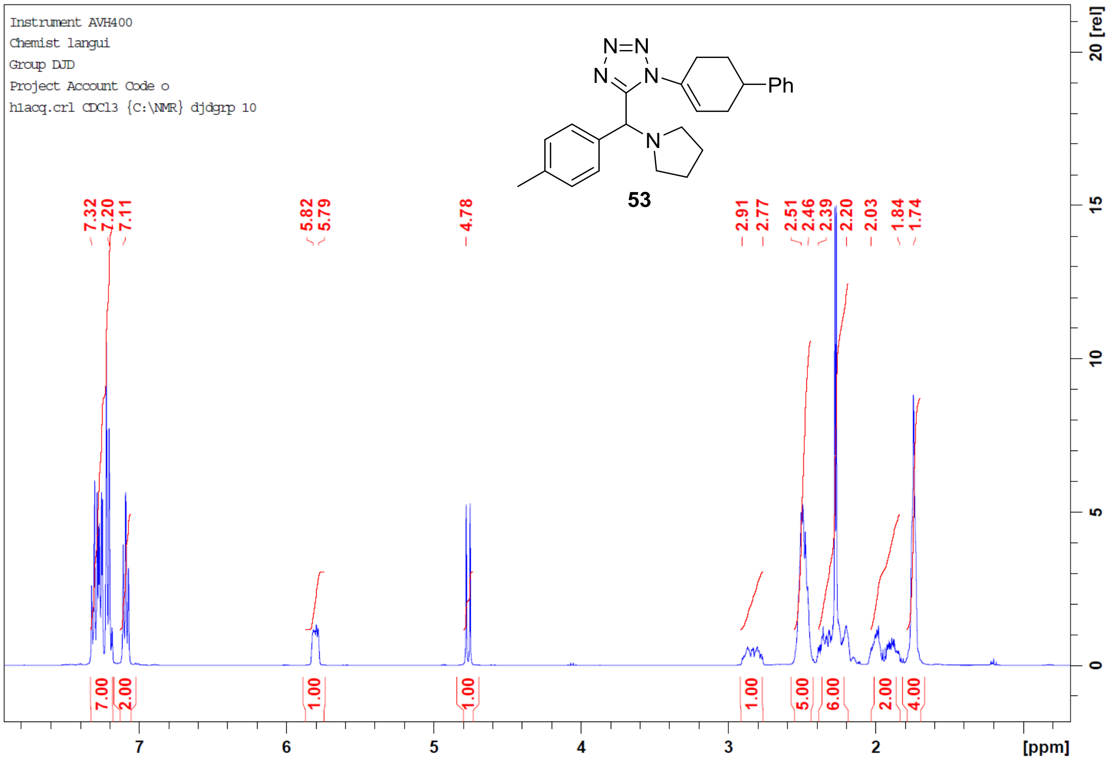

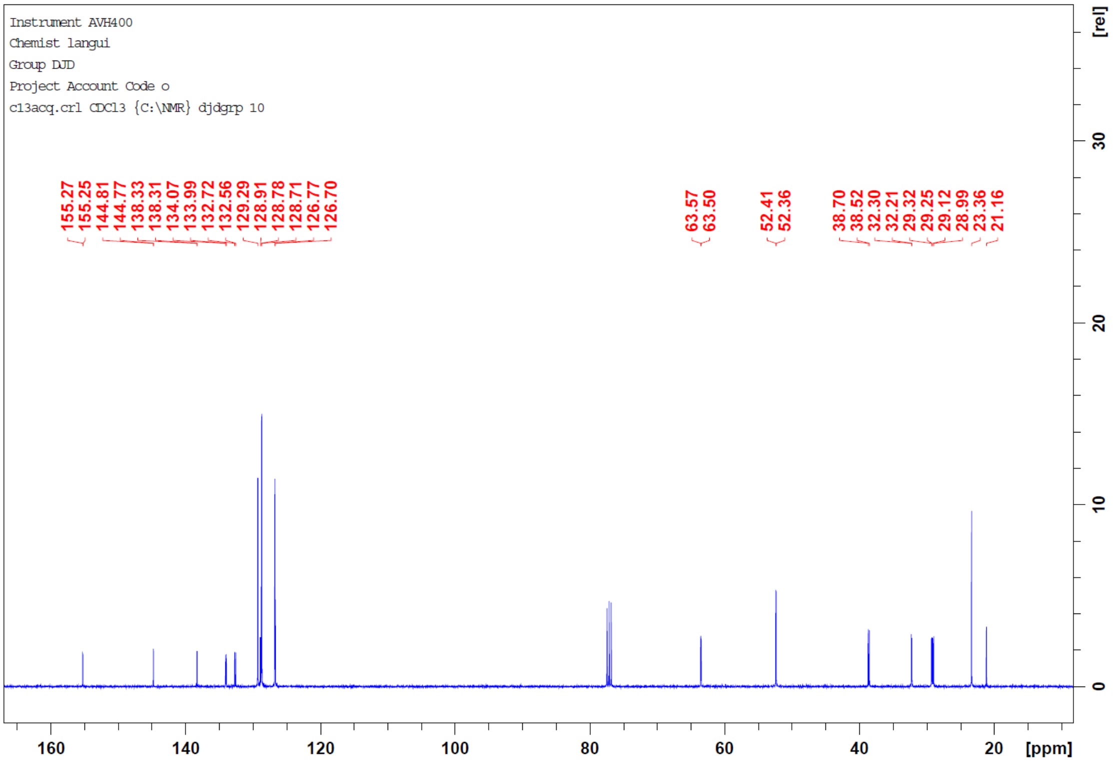

Supplementary Figure 53. ^{1}H NMR and ^{13}C NMR spectra of product **53**.

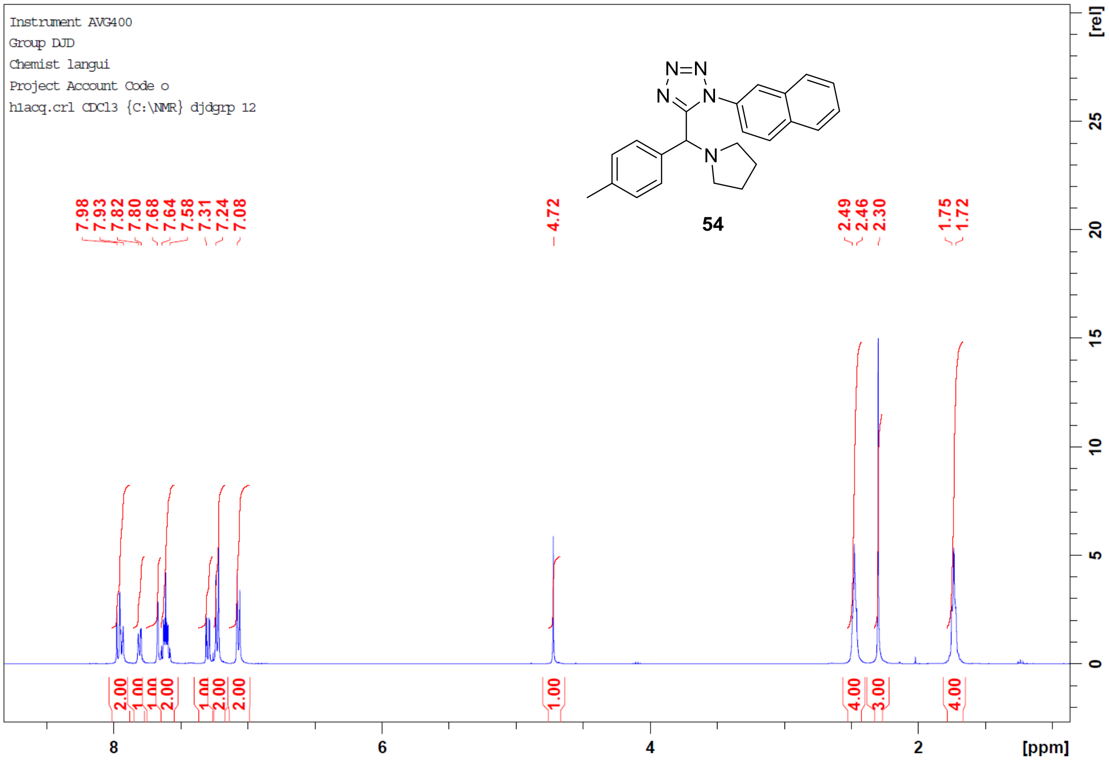

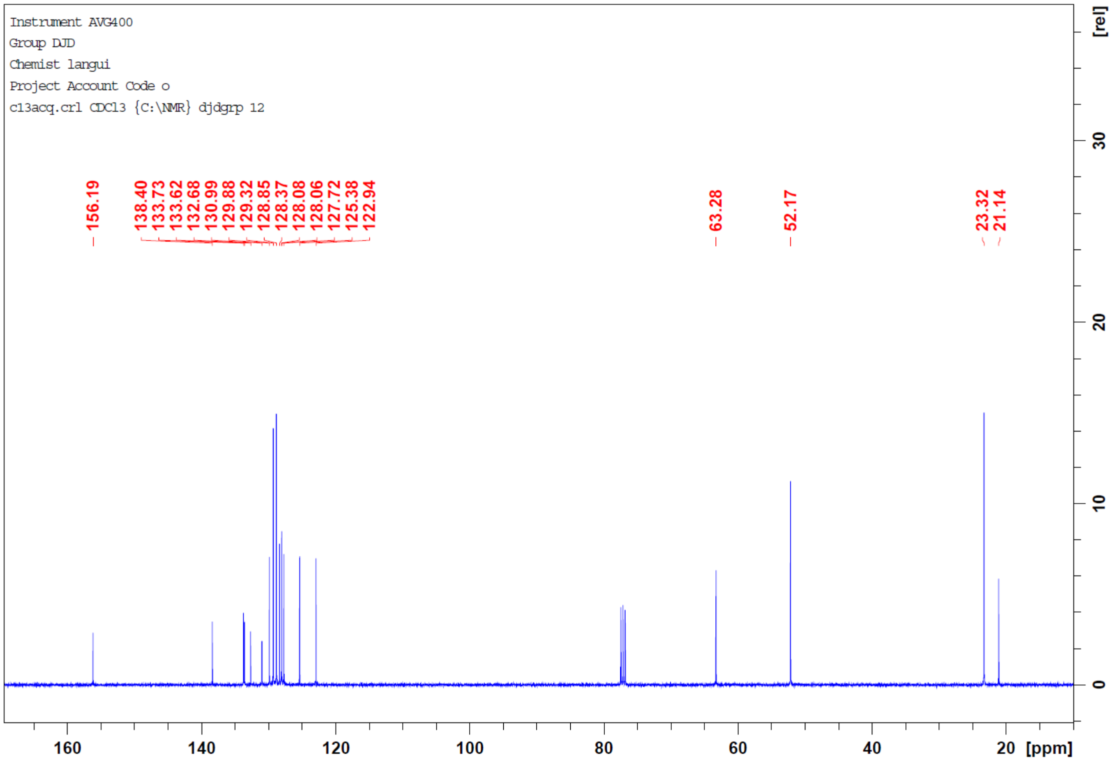

Supplementary Figure 54. ^{1}H NMR and ^{13}C NMR spectra of product **54**.

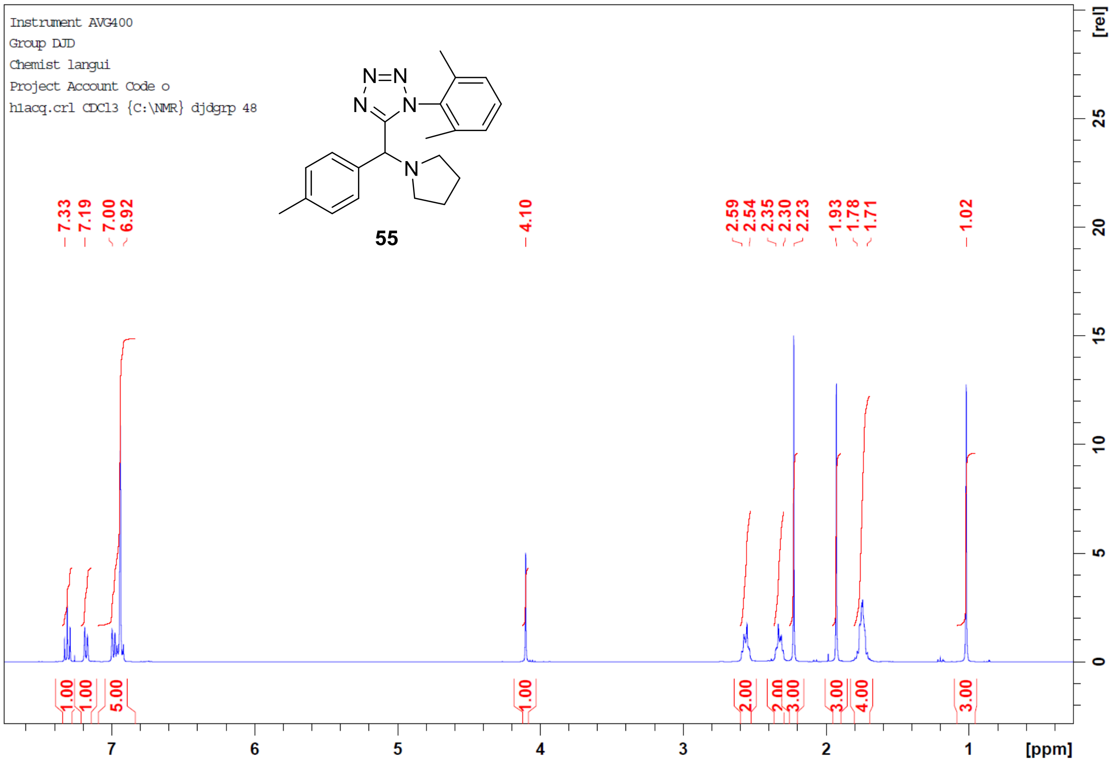

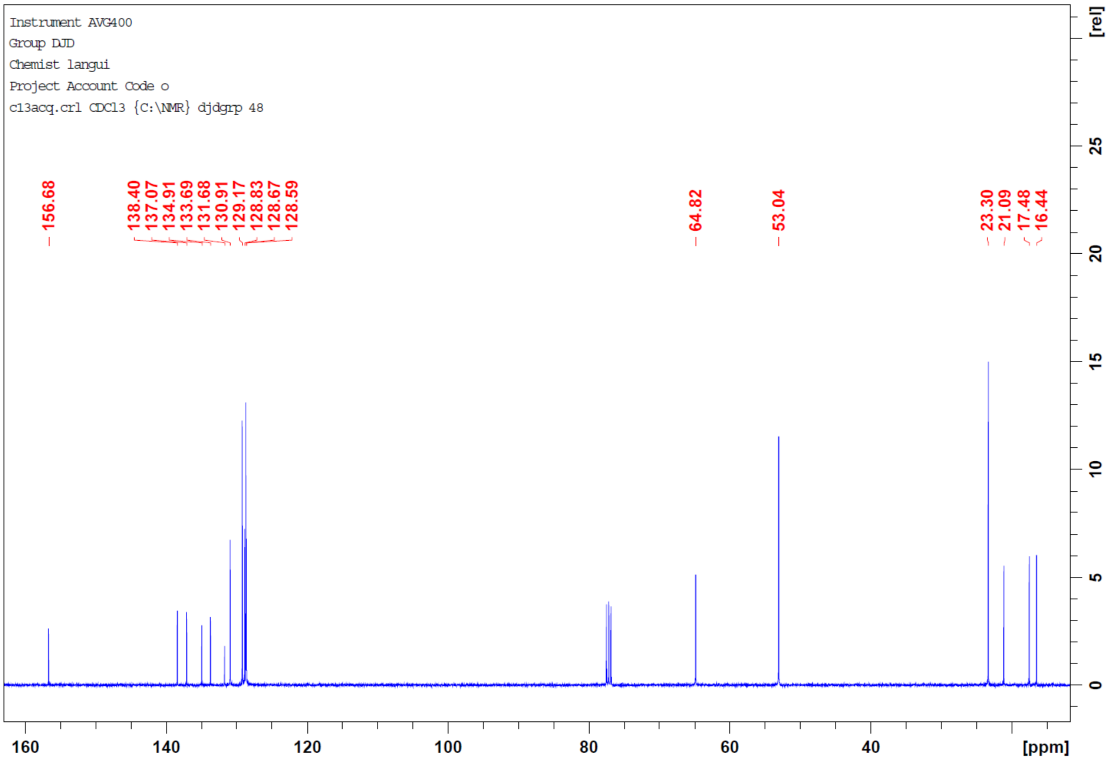

Supplementary Figure 55. ^{1}H NMR and ^{13}C NMR spectra of product **55**.

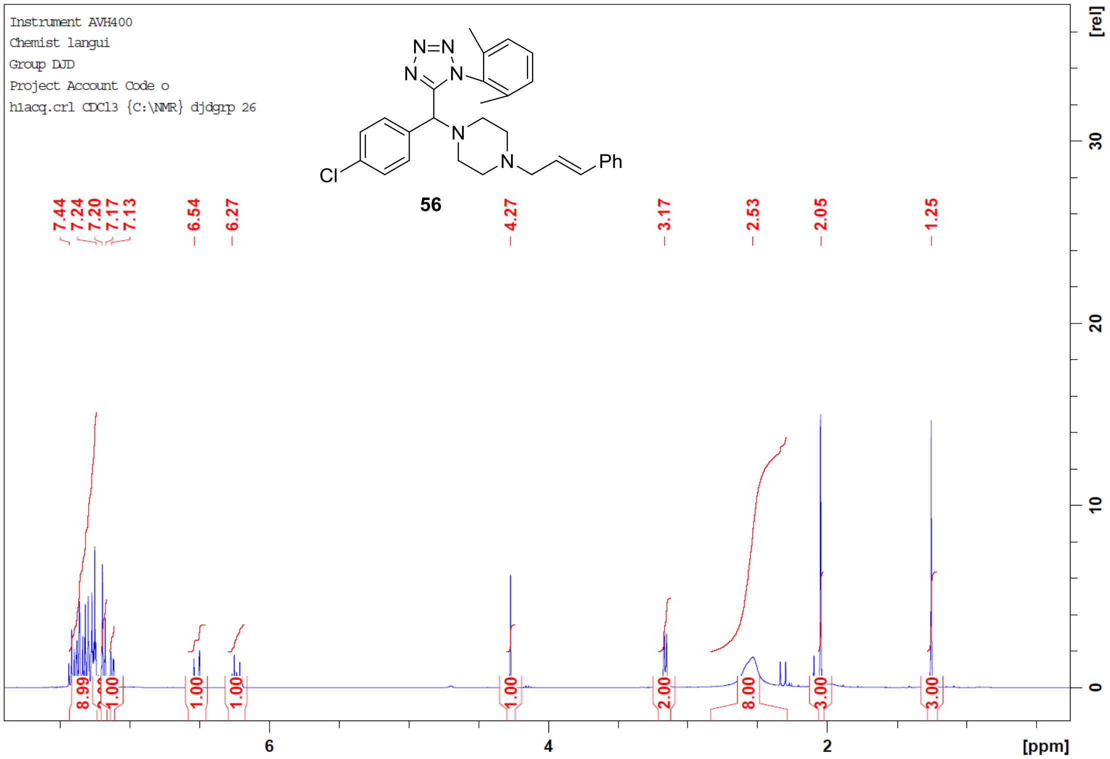

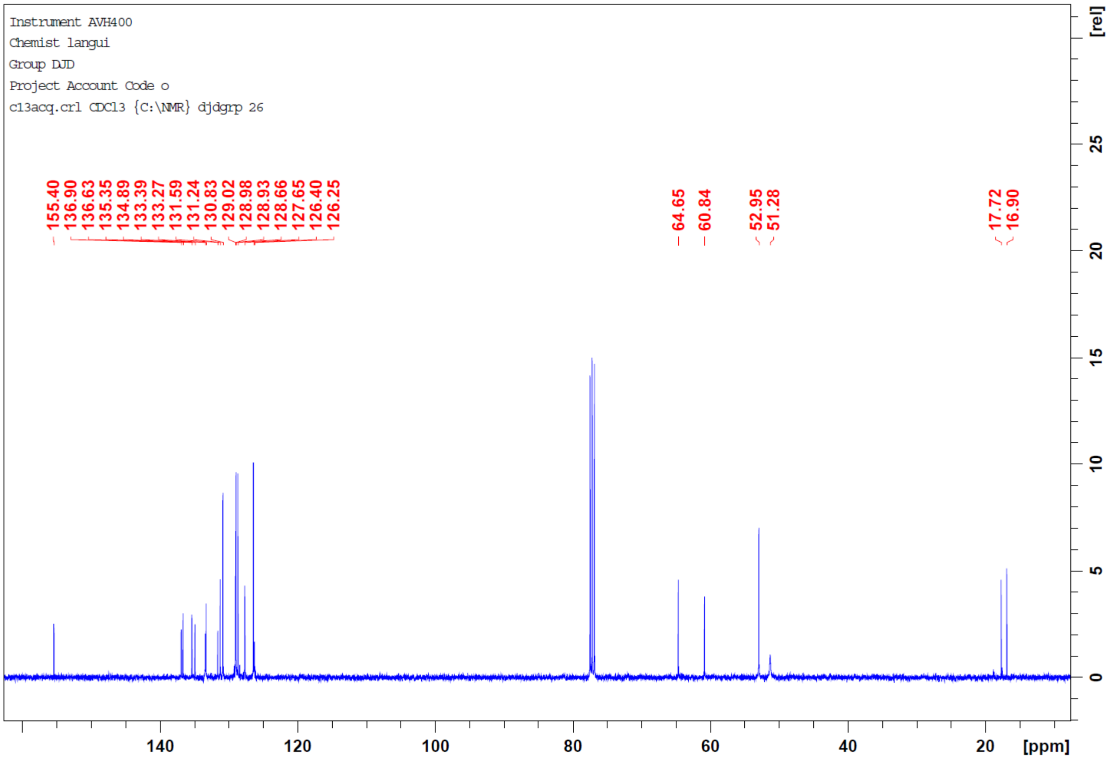

Supplementary Figure 56. ^{1}H NMR and ^{13}C NMR spectra of product **56**.

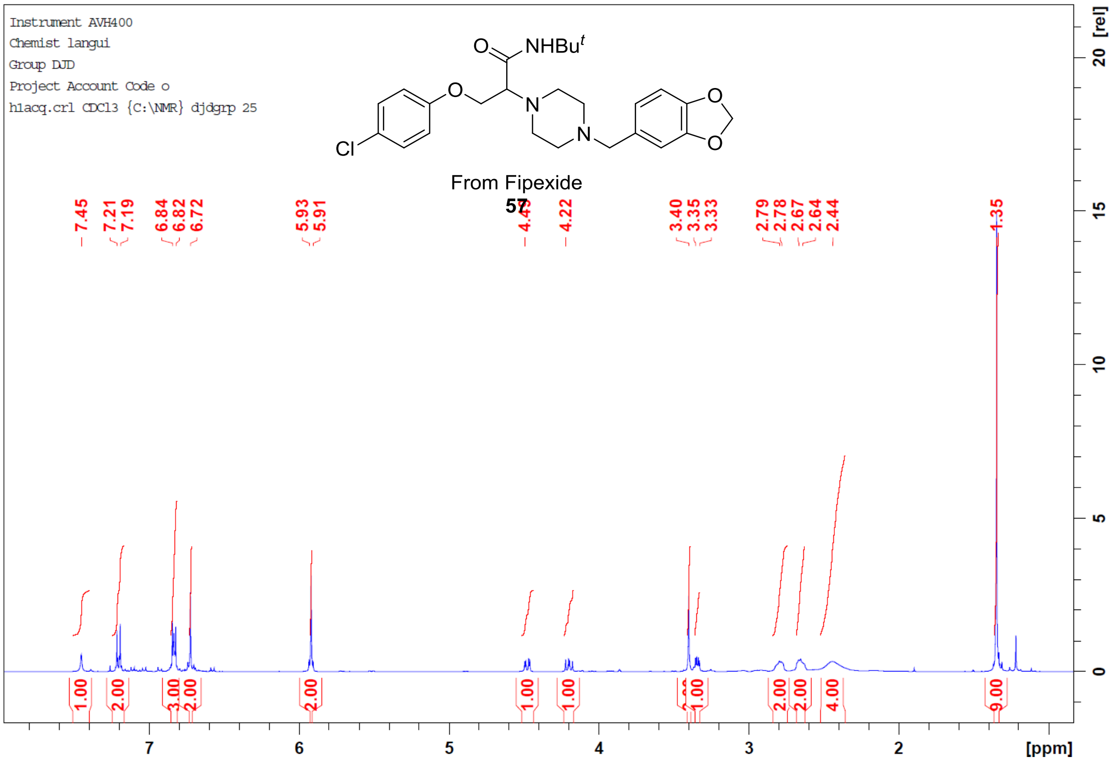

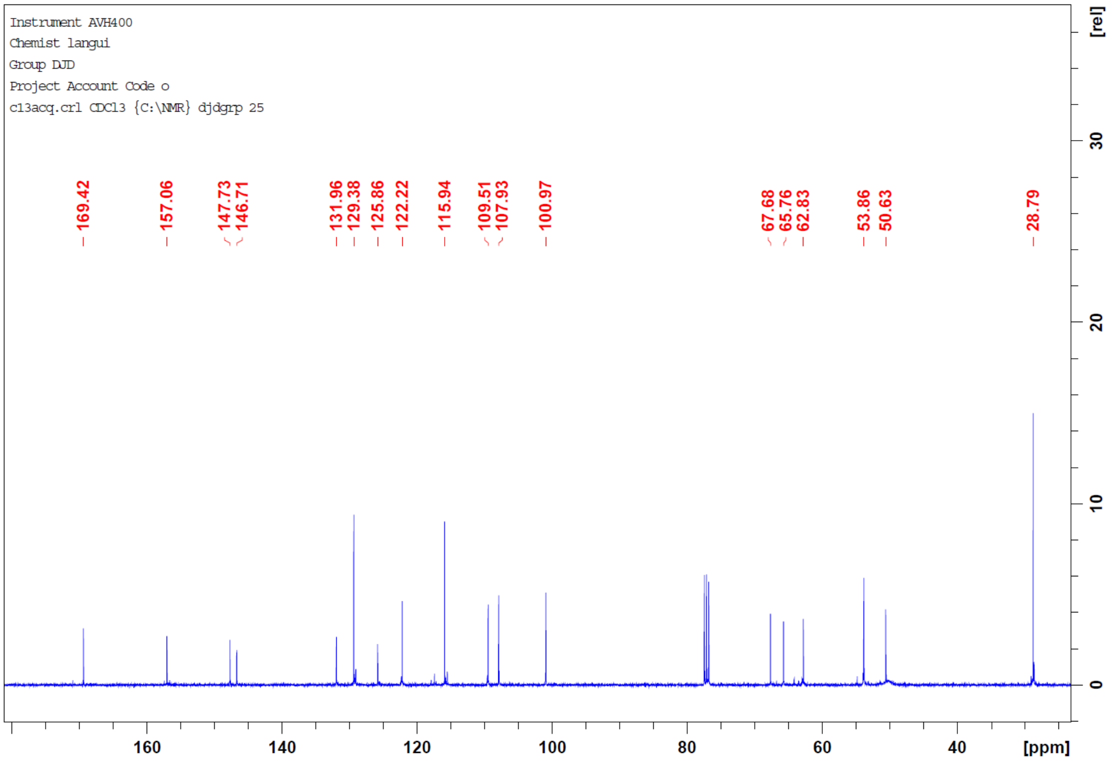

Supplementary Figure 57. ^{1}H NMR and ^{13}C NMR spectra of product **57**.

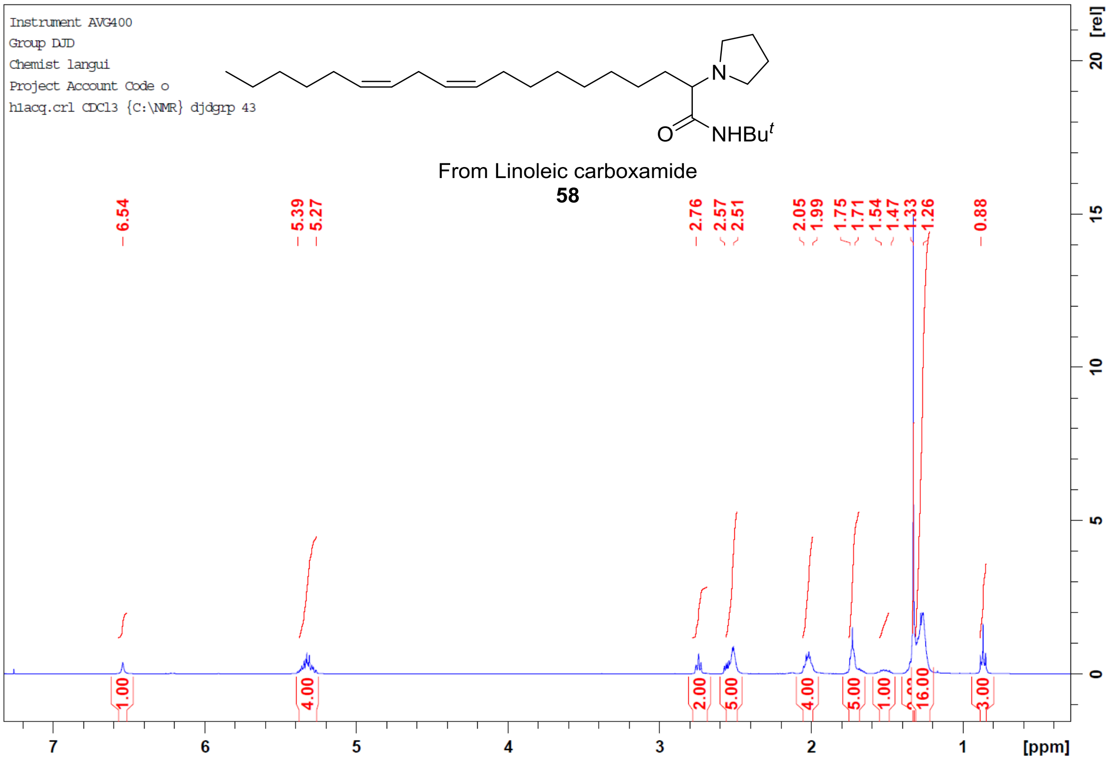

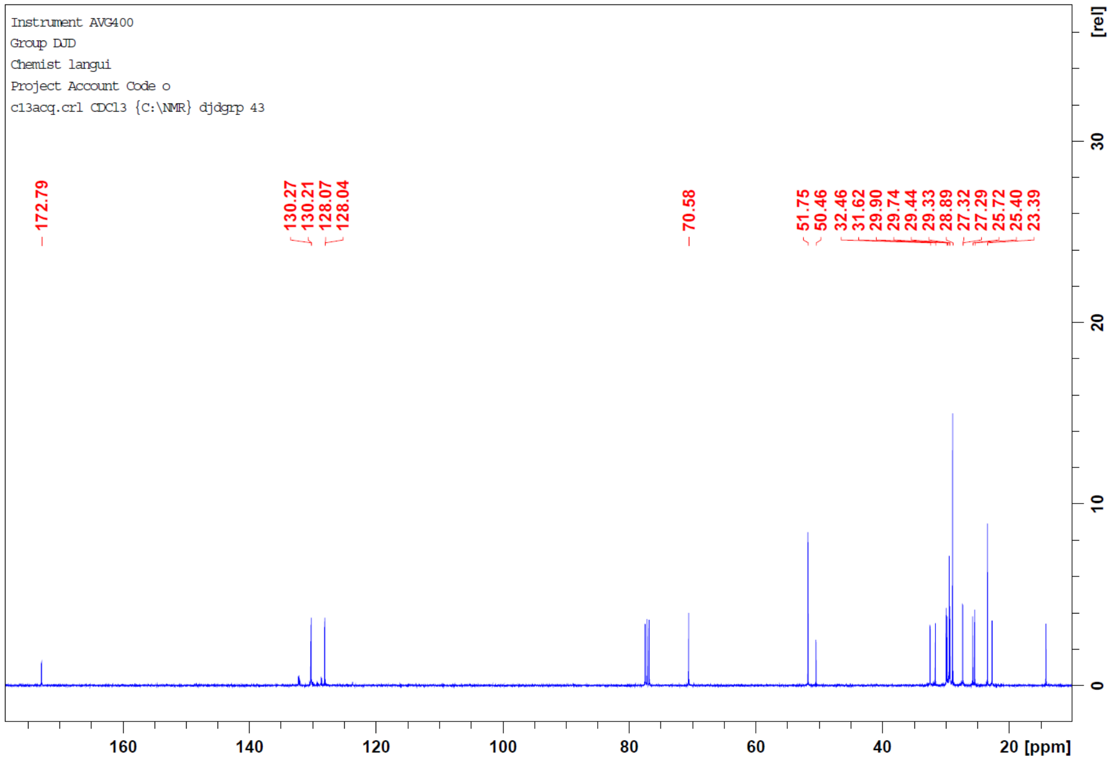

Supplementary Figure 58. ^{1}H NMR and ^{13}C NMR spectra of product **58**.

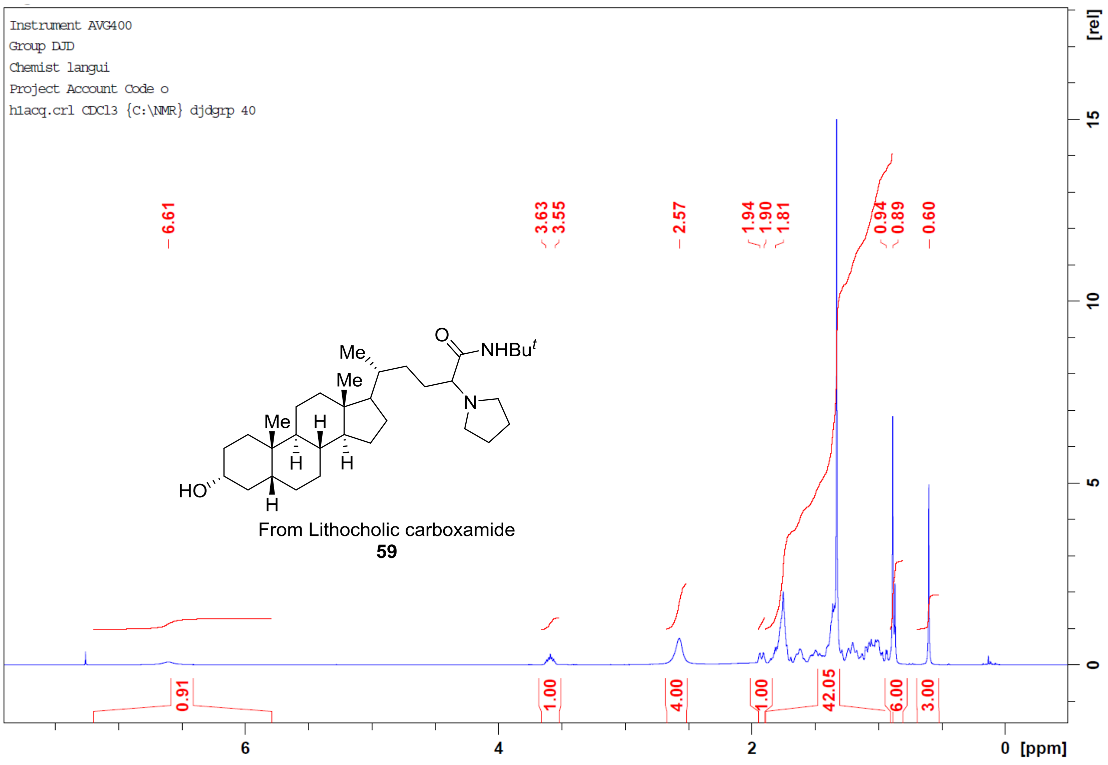

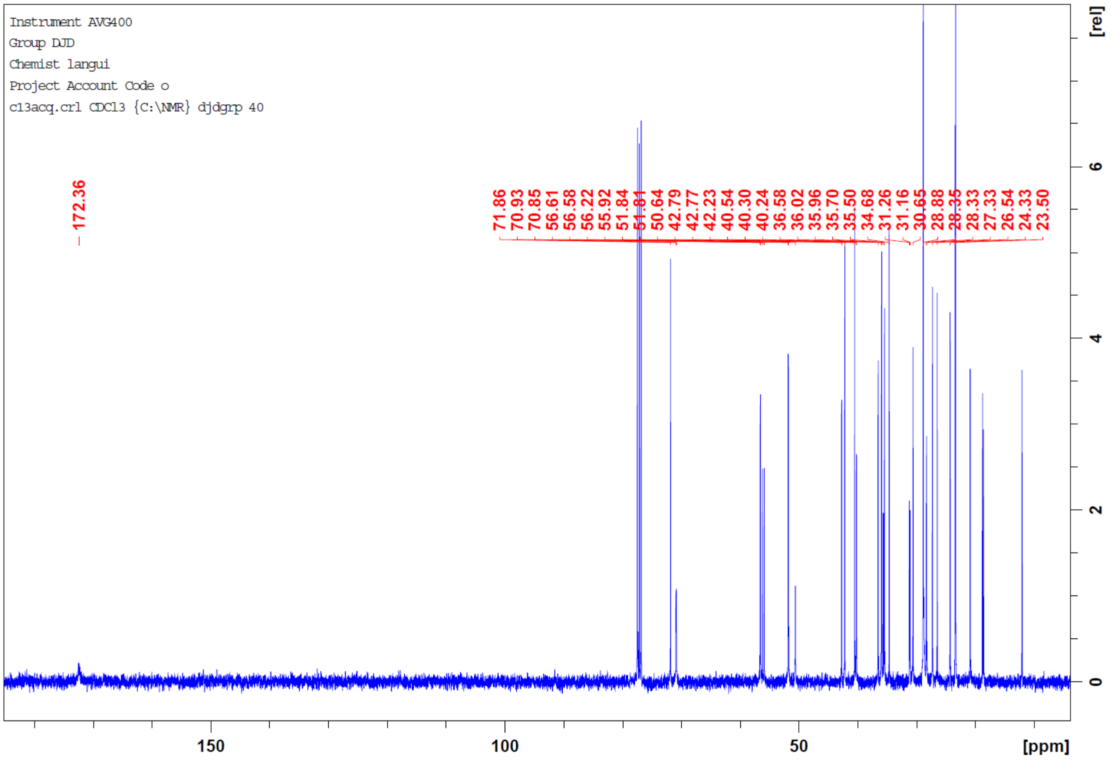

Supplementary Figure 59. ^{1}H NMR and ^{13}C NMR spectra of product **59**.

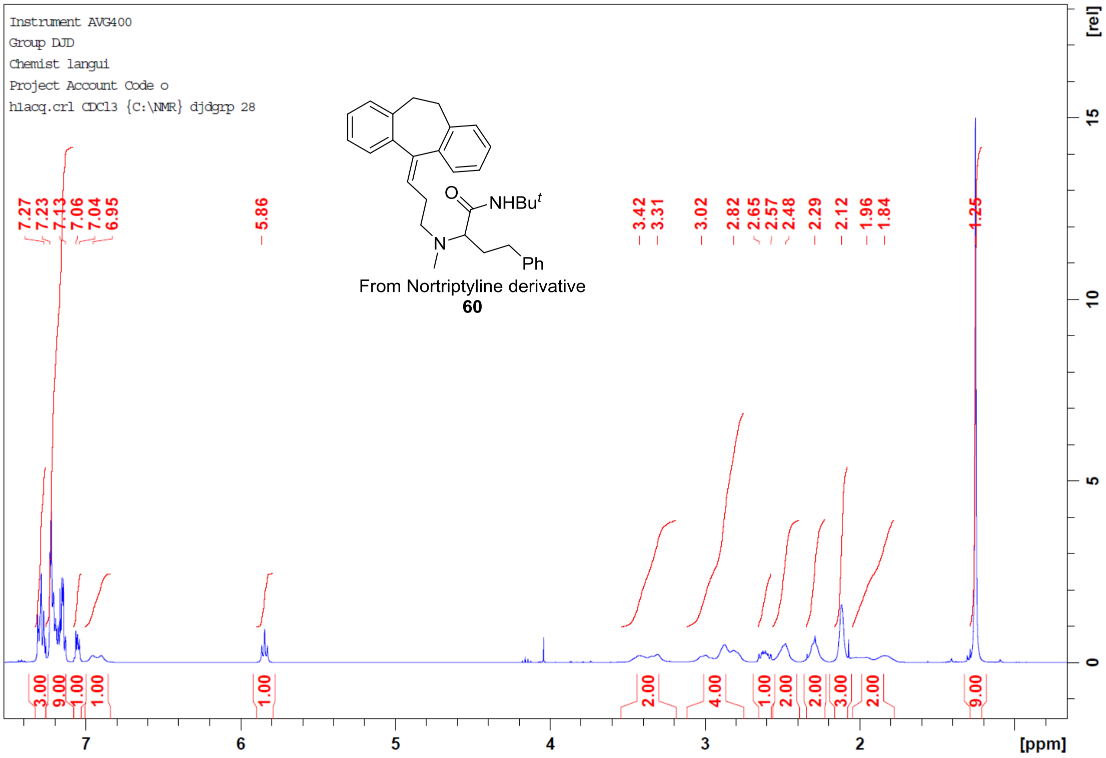

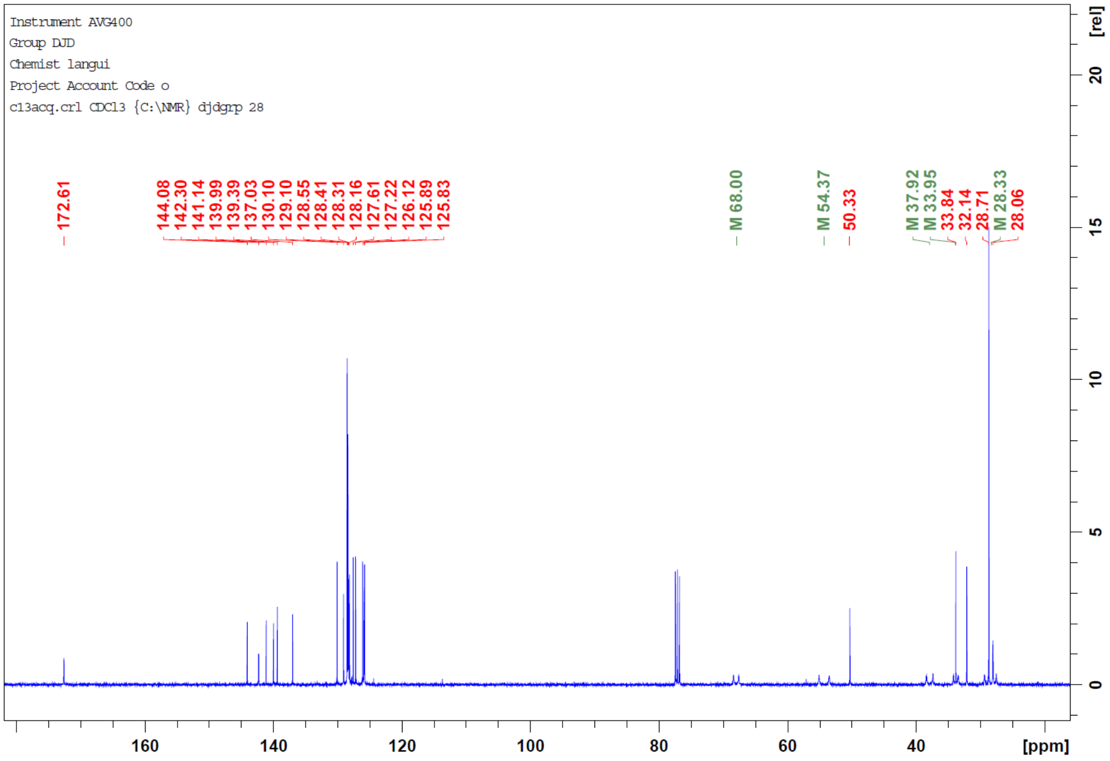

Supplementary Figure 60. ^{1}H NMR and ^{13}C NMR spectra of product **60**.

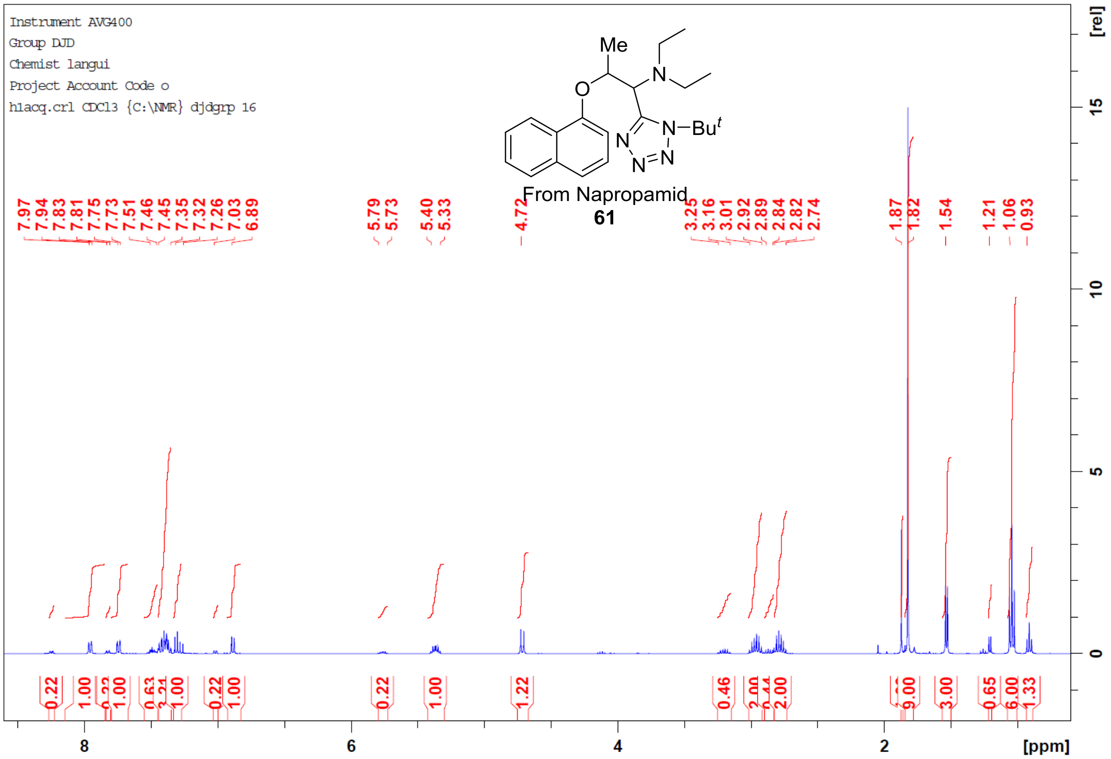

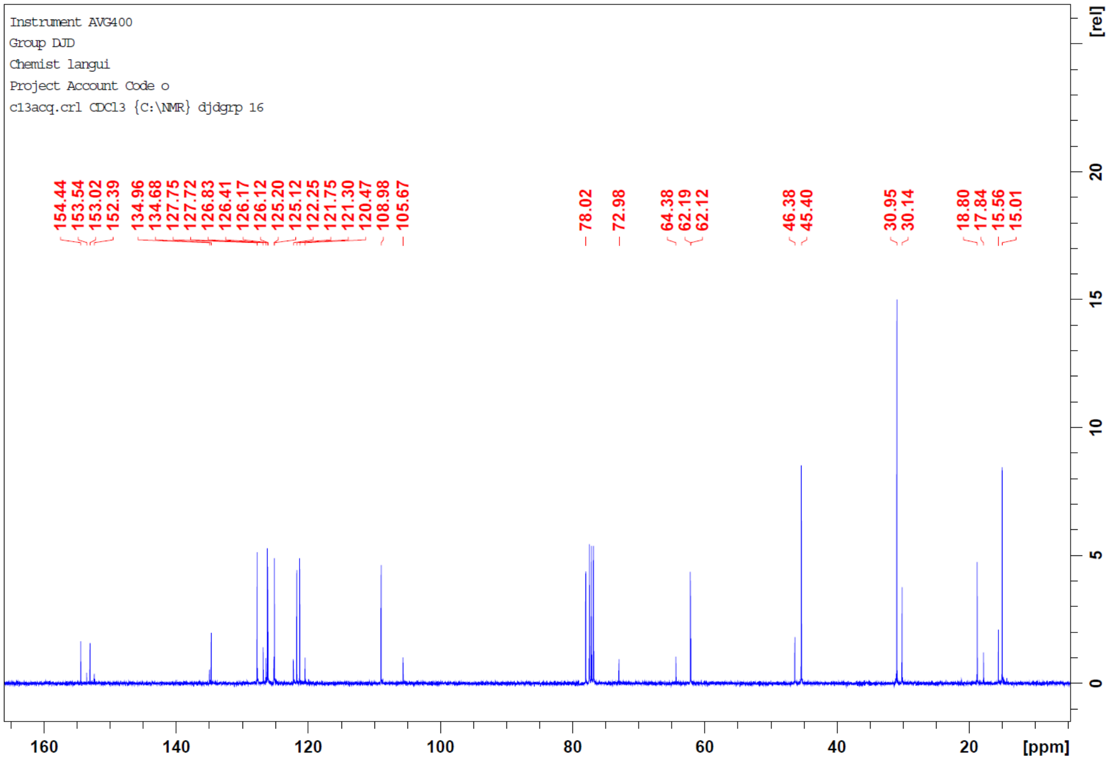

Supplementary Figure 61. ^{1}H NMR and ^{13}C NMR spectra of product **61**.

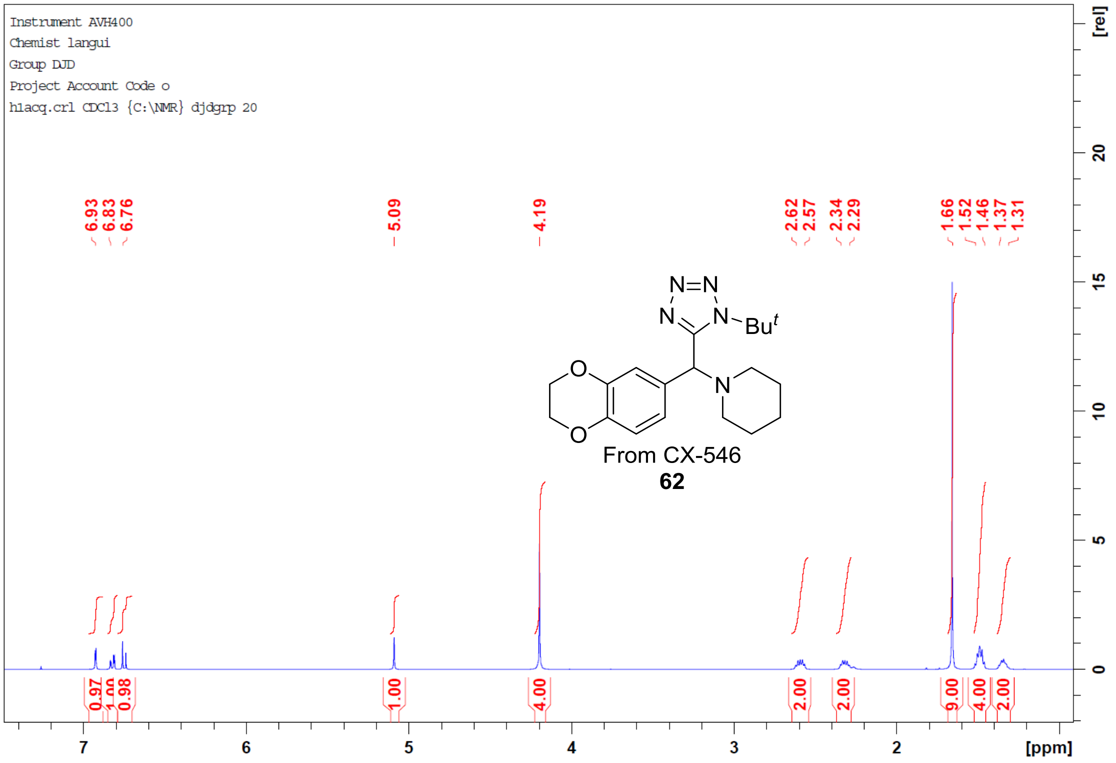

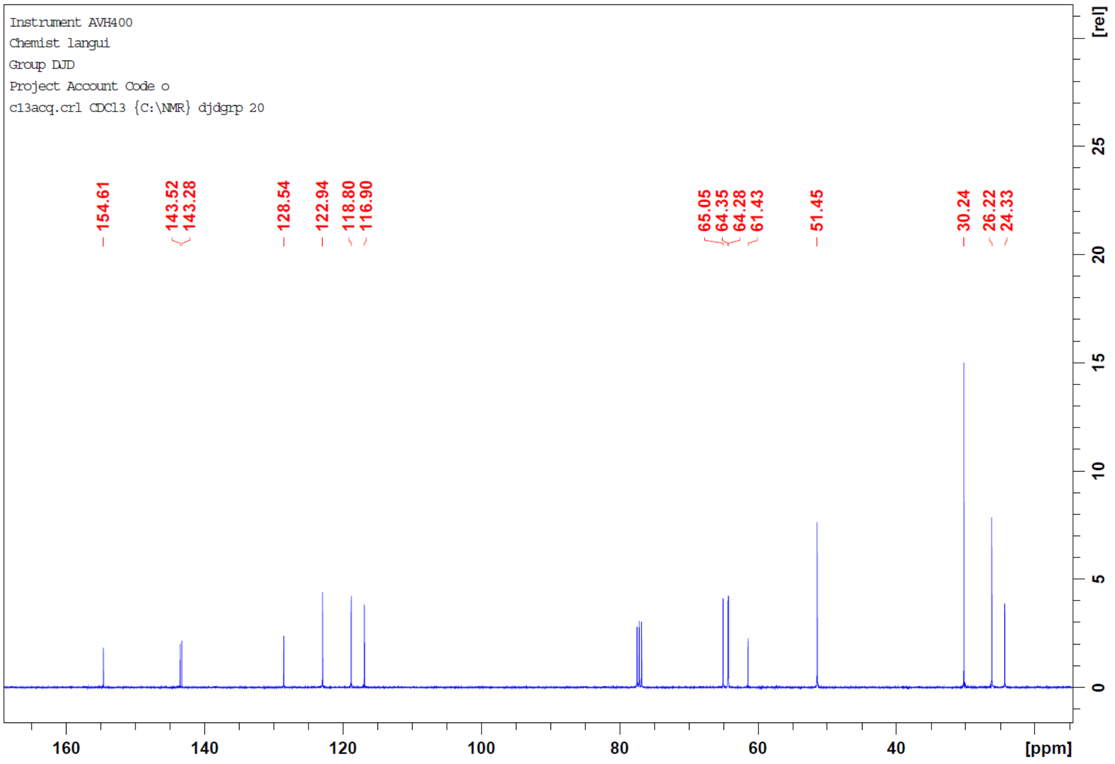

Supplementary Figure 62. ^{1}H NMR and ^{13}C NMR spectra of product **62**.

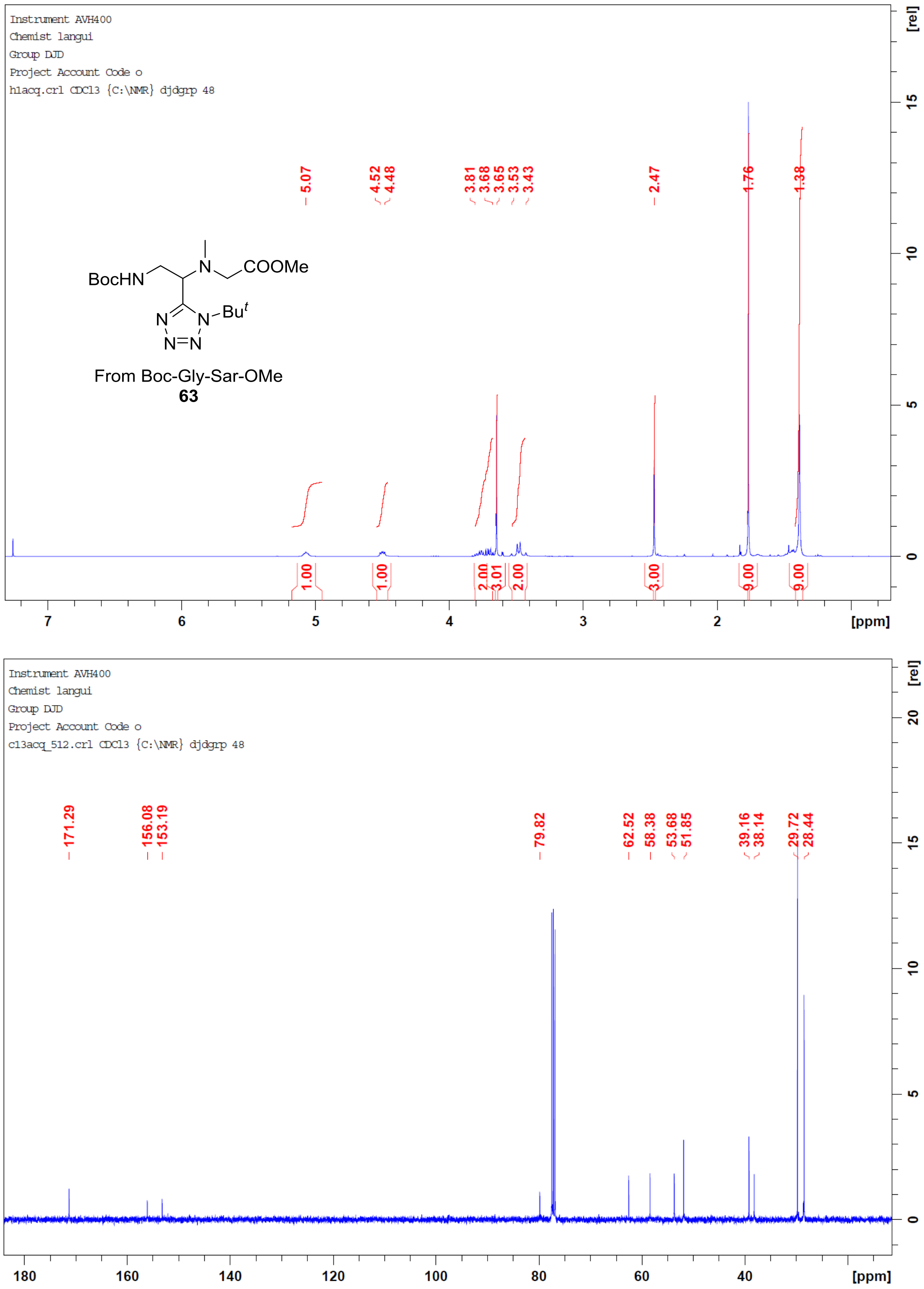

Supplementary Figure 63. ^{1}H NMR and ^{13}C NMR spectra of product **63**.

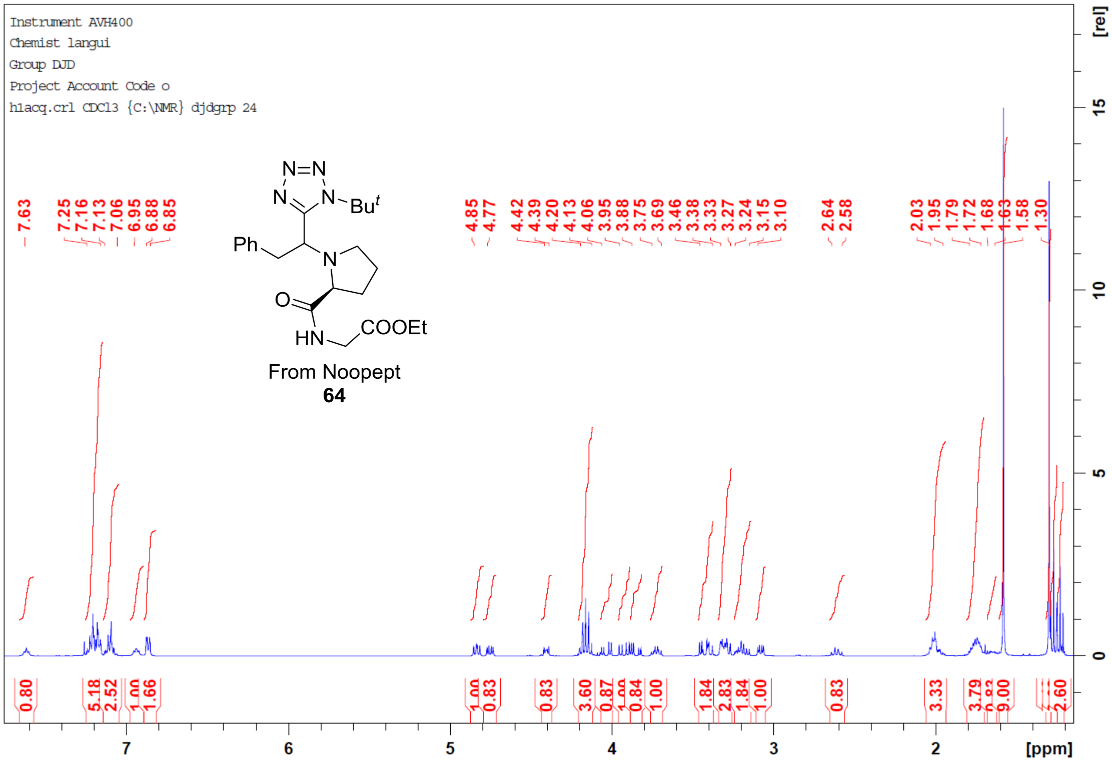

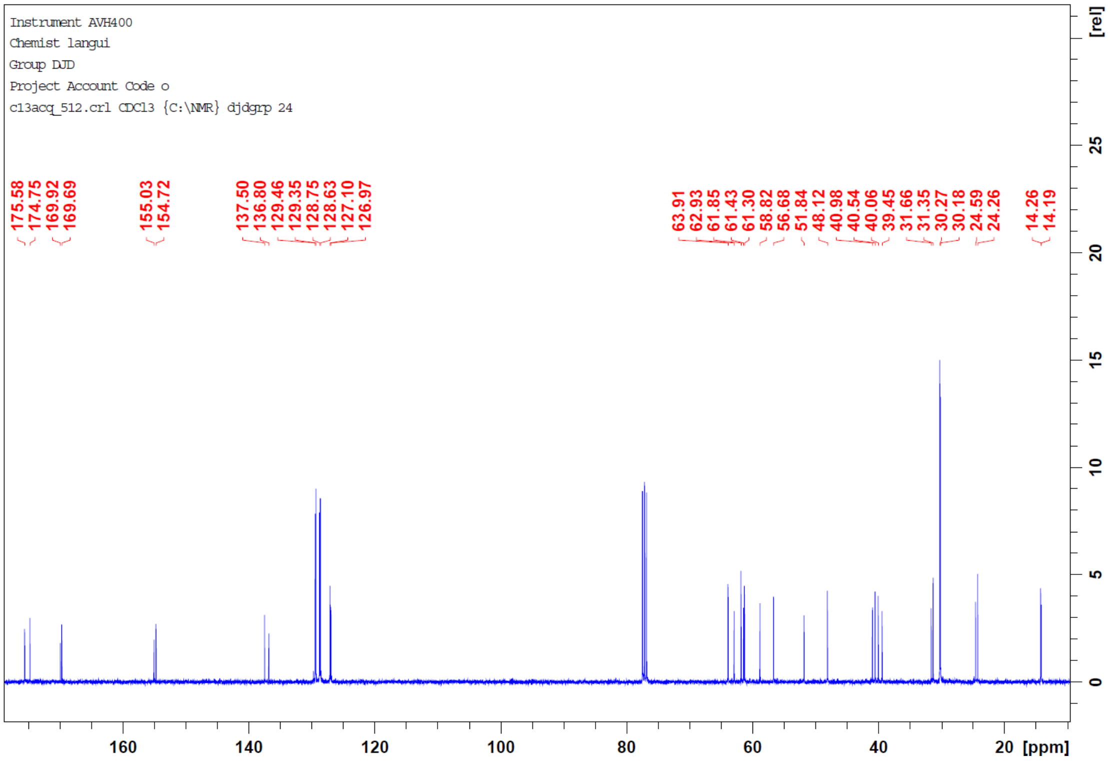

Supplementary Figure 64. ^{1}H NMR and ^{13}C NMR spectra of product **64**.

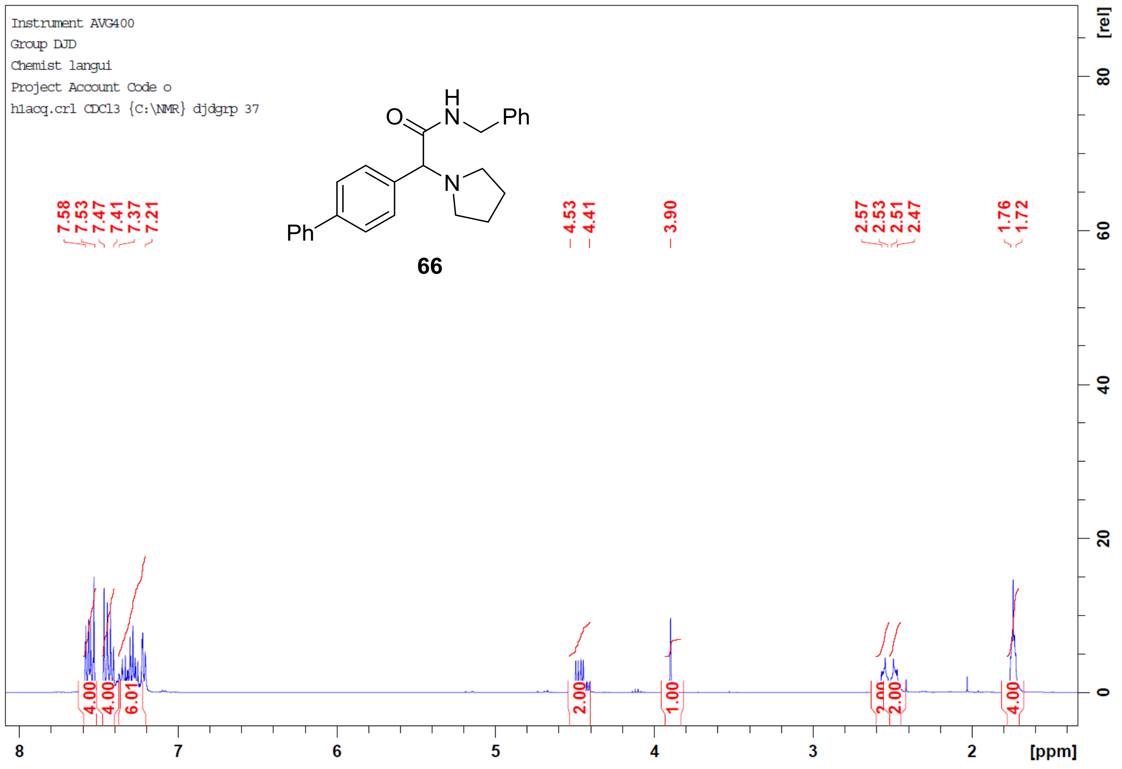

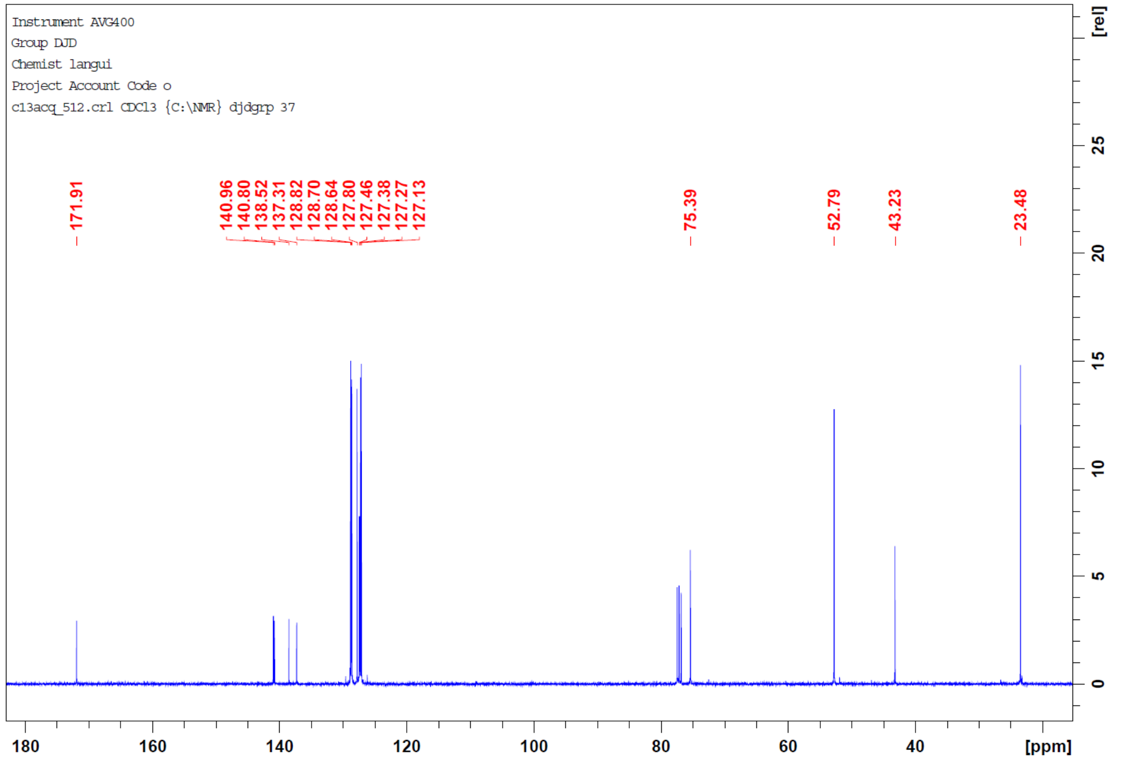

Supplementary Figure 65. ^{1}H NMR and ^{13}C NMR spectra of product **66**.

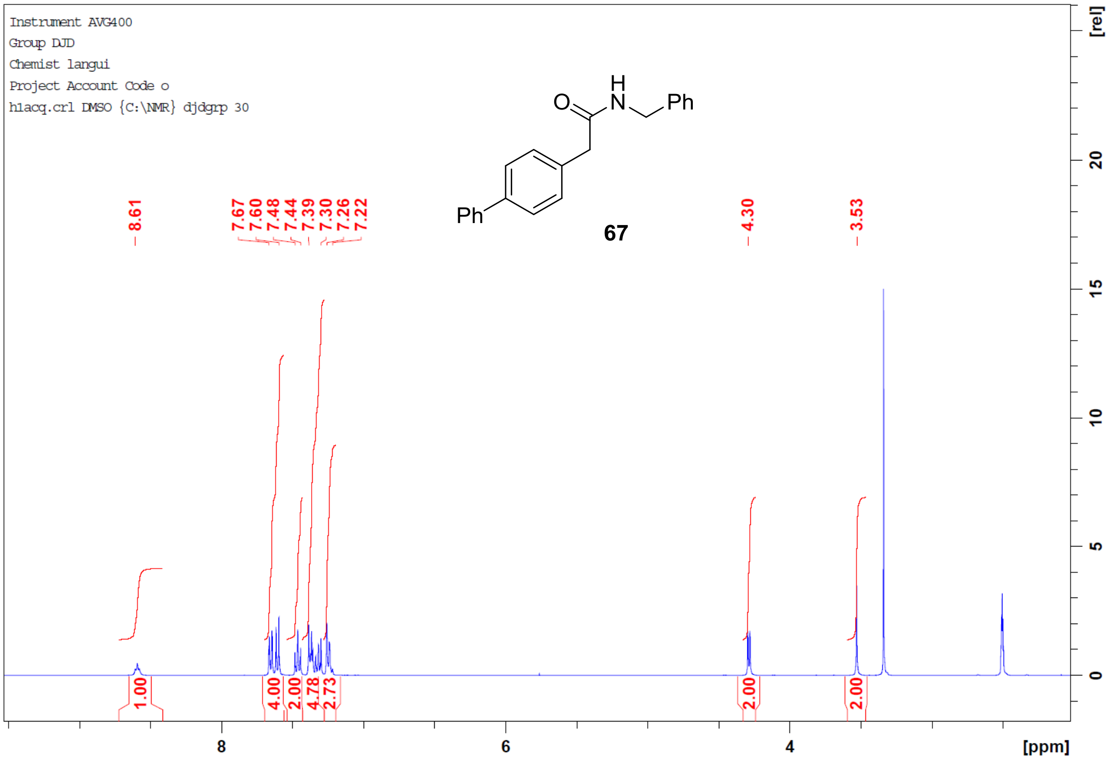

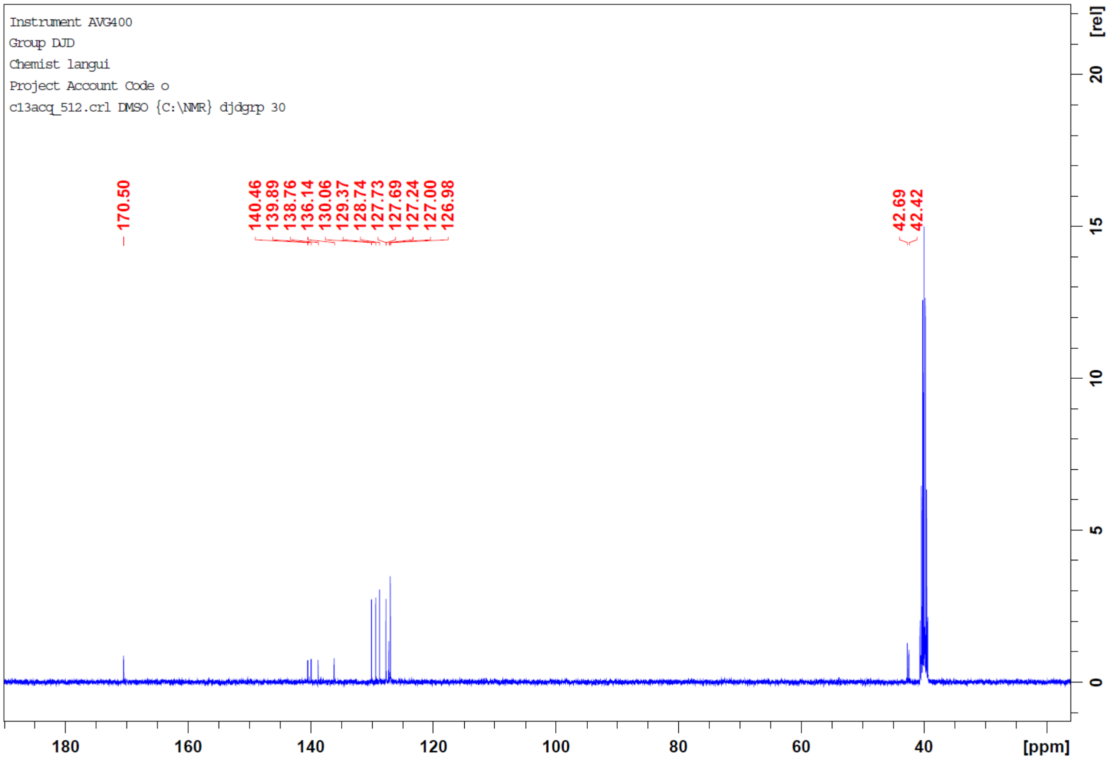

Supplementary Figure 66. ^{1}H NMR and ^{13}C NMR spectra of product **67**.

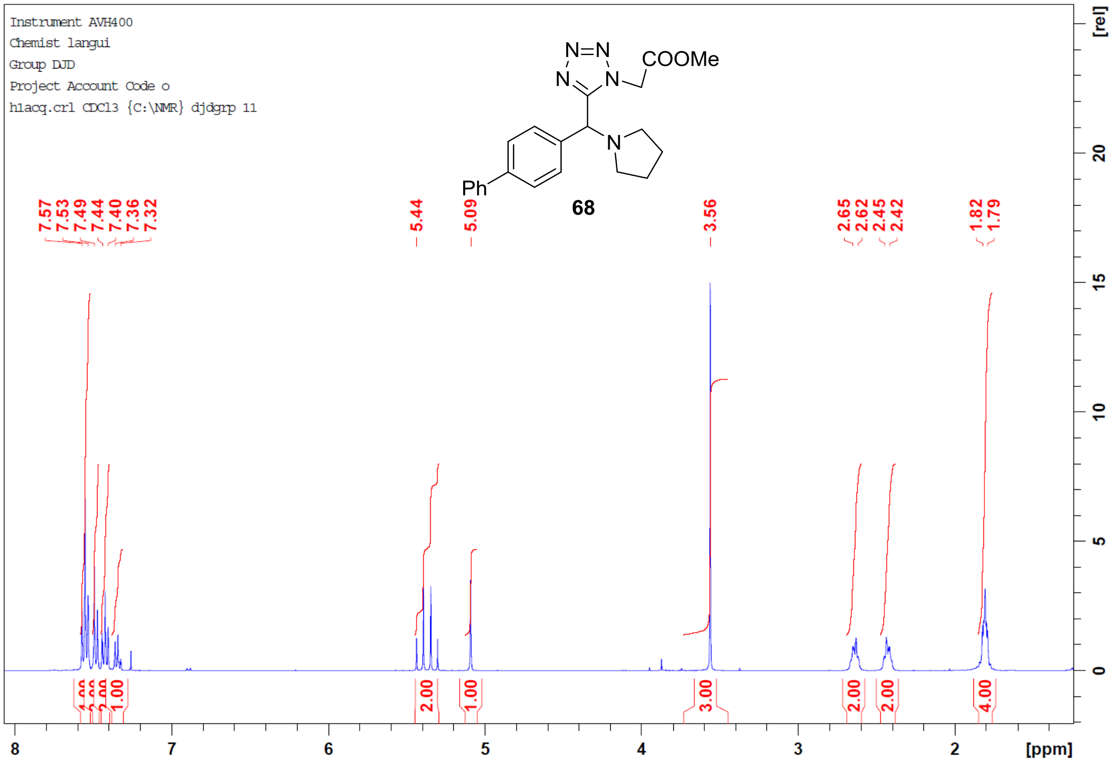

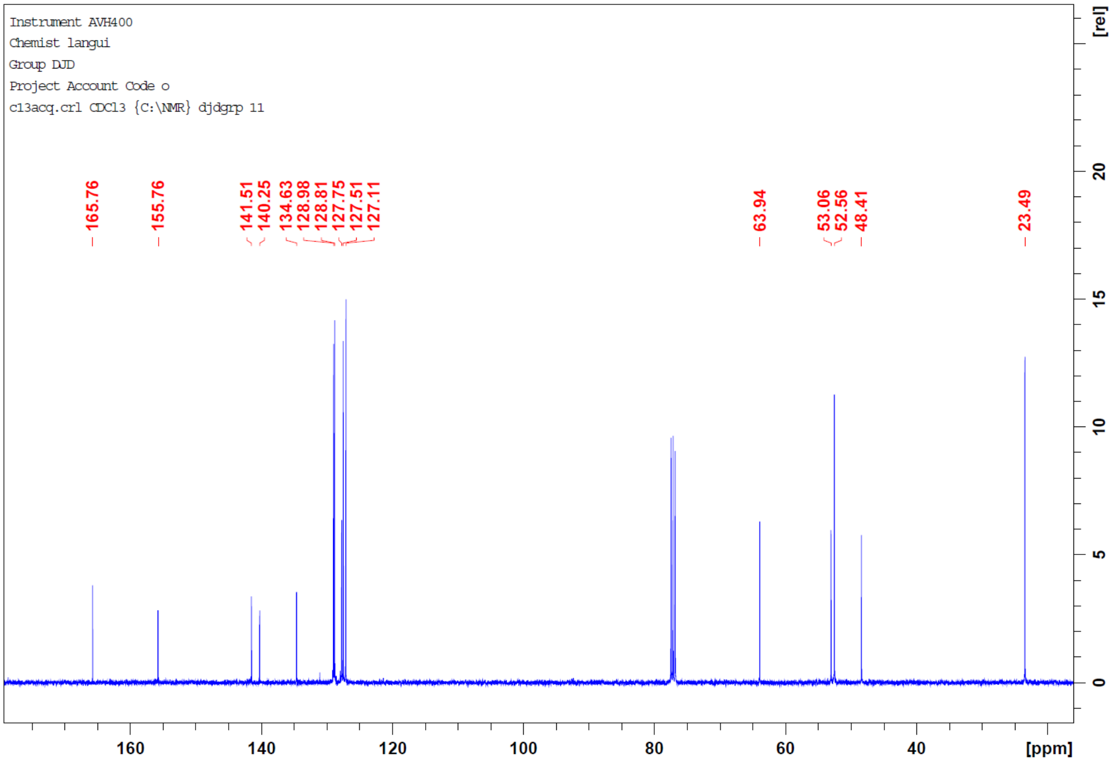

Supplementary Figure 67. ^{1}H NMR and ^{13}C NMR spectra of product **68**.

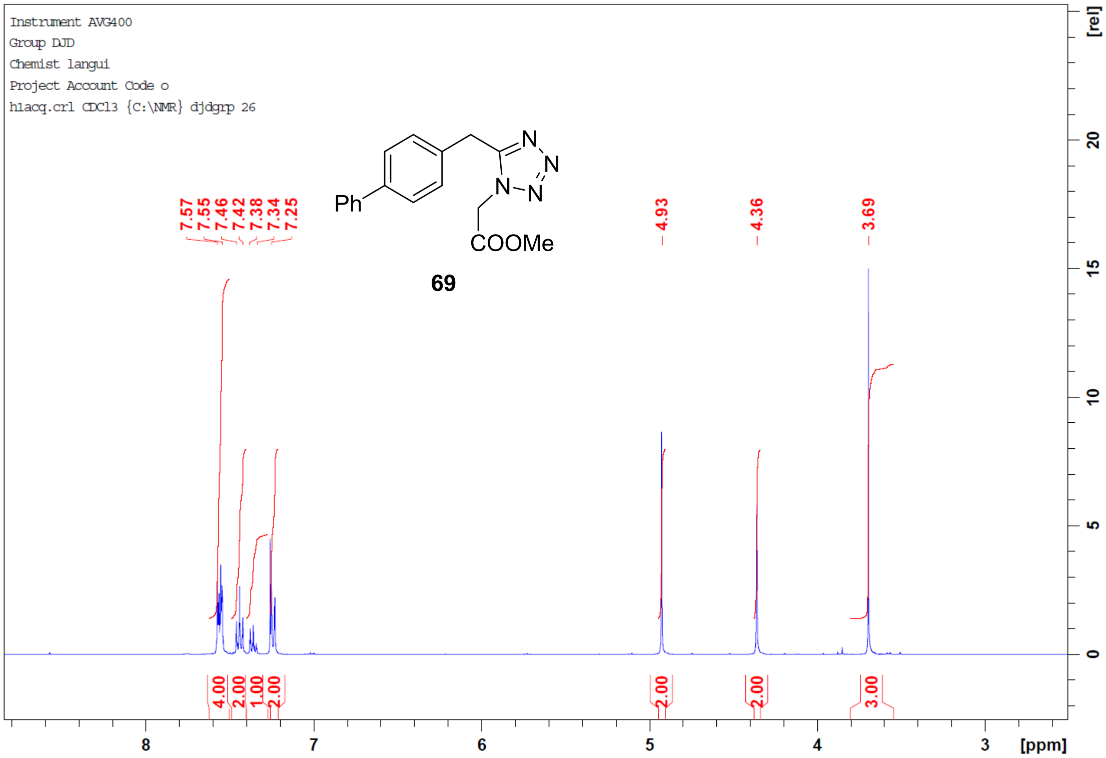

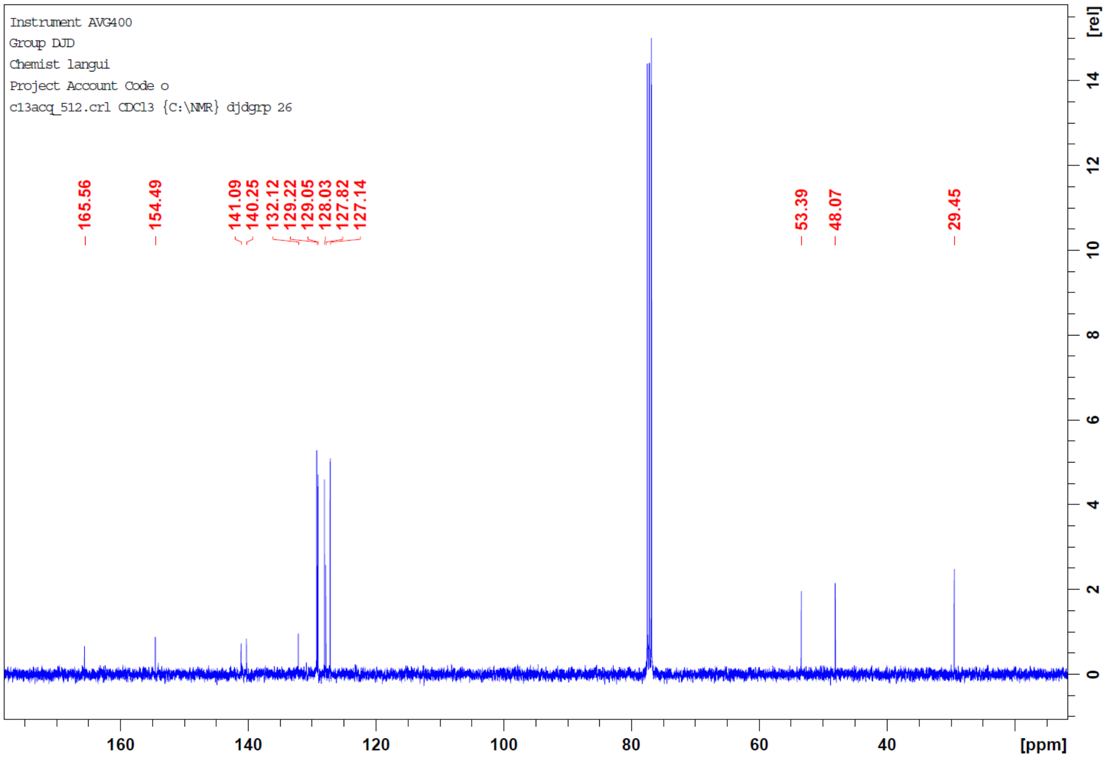

Supplementary Figure 68. ^{1}H NMR and ^{13}C NMR spectra of product **69**.

Supplementary Methods

General information

All reagents bought from commercial sources were used as received. Organic solvents were evaporated under reduced pressure using a Büchi rotary evaporator. All solvents were commercially supplied or dried by filtration through activated alumina (powder ~150 mesh, pore size 58 Å, basic, Sigma-Aldrich) columns. Petrol ether (PE) refers to distilled light petroleum of fraction 30 - 40 °C. Toluene was distilled twice over calcium hydride. All reactions were followed by thin-layer chromatography (TLC) when practical, using Merck Kieselgel 60 F_{254} fluorescent treated silica. Visualisation was accomplished under UV light (λmax= 254 nm) and by staining with $KMnO_4$ staining dip. Chromatographic purification was performed on VWR 60 silica gel 40-63 µm using HPLC grade solvents that were used as supplied. High resolution mass spectra (HRMS) were recorded on a Bruker Daltonics MicroTOF mass spectrometer equipped with an ESI source or on a Micromass GCT equipped with an EI source unless otherwise specified. Infrared absorption spectra (IR) were recorded on a Bruker Tensor 27 FT-IR spectrometer from a thin film on a diamond ATR module. Only selected bands (ν_{max}) are reported in wavenumbers (cm^{-1}). NMR spectra were recorded on Bruker spectrometers operating at 400 or 500 MHz (^{1}H resonance). Proton chemical shifts (δ) are given in parts per million (ppm) relative to tetramethylsilane (TMS) with the solvent resonance ($CDCl_3$, δ = 7.26 ppm) as internal standard. The following abbreviations are used to describe spin multiplicity: s = singlet, d = doublet, t = triplet, q = quartet, dd = doublet of doublets, dt = doublet of triplets, m = multiplet, br = broad signal. Coupling constants (*J*) are given in Hertz (Hz). ^{13}C-NMR spectra were recorded with complete proton decoupling. Carbon chemical shifts are reported in ppm (δ) relative to TMS with the solvent resonance ($CDCl_3$, δ = 77.16 ppm) as internal standard.

Preparation of amides

General procedure for the synthesis of amides

Procedure **A**: To a solution of the amine (1.1 eq) and Et_3N (1.5 eq) in dichloromethane (2 mL/mmol), acyl chloride (1 eq) was added in dropwise at 0 °C; Procedure **B**: Amine (1.1 eq) was added to the mixture of acid (1.0 eq), EDCI (1.1 eq), DMAP (0.1 eq) and Et_3N (1.2 eq) in dichloromethane (2 mL/mmol) at 0 °C. The

reaction mixture was stirred overnight at room temperature. The solution was then washed with 1N HCl, dried over Na_2SO_4 and concentrated under reduced pressure for silica gel column. Known amides are consistent with reported NMR spectru.

Synthesis of pyrrolidin-1-yl(*p*-tolyl)methanone (S1)[1]

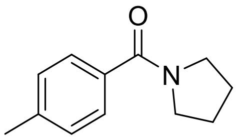

According to the general procedure **A**, using *p*-toluoyl Chloride (1546 mg, 10 mmol) and pyrrolidine (853 mg, 12 mmol), pyrrolidin-1-yl(*p*-tolyl)methanone was obtained as a white solid after flash column chromatography on silica gel (1834 mg, 97% yield).

Synthesis of *N,N*-dimethyl-4-propoxybenzamide (S2)

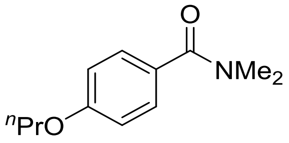

According to the general procedure **A**, using 4-propoxybenzoyl chloride (596 mg, 3 mmol) and dimethylamine hydrochloride (269 mg, 3.3 mmol), *N,N*-dimethyl-4-propoxybenzamide was obtained as a colorless oil after flash column chromatography on silica gel (565 mg, 91% yield).

IR (neat) ν_{max}: 2935, 1627, 1606, 1245, 841;

^{1}H-NMR (400 MHz, $CDCl_3$): δ 7.36-7.34 (m, 2H), 6.87-6.85 (m, 2H), 3.92 (t, *J* = 6.6 Hz, 2H), 3.02 (brs, 6H), 1.82-1.74 (m, 2H), 1.02 (t, *J* = 7.4 Hz, 3H);

^{13}C-NMR (100 MHz, $CDCl_3$): δ 171.6, 160.2, 129.1, 128.2, 114.1, 69.6, 39.8, 35.6, 22.5, 10.5;

HRMS (ESI+): exact mass calculated for $[M+H]^+$ ($C_{21}H_{28}N$) requires *m/z* 208.1332, found *m/z* 208.1332.

Synthesis of *N,N*-dimethyl-4-cyano-benzamide (S3)[2]

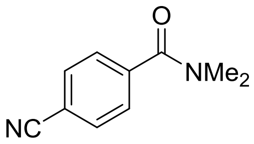

According to the general procedure **A**, using 4-cyanobenzoyl chloride (497 mg, 3 mmol) and dimethylamine hydrochloride (269 mg, 3.3 mmol), *N,N*-dimethyl-4-cyano-benzamide was obtained as a white crystalline solid after flash column chromatography on silica gel (423 mg, 81% yield).

Synthesis of *N,N*-dimethyl-3,5-bis(trifluoromethyl)benzamide (S4)

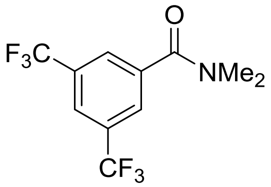

According to the general procedure **A**, using 3,5-bis(trifluoromethyl)benzoyl chloride (830 mg, 3 mmol) and dimethylamine hydrochloride (269 mg, 3.3 mmol), *N,N*-dimethyl-3,5-bis(trifluoromethyl)benzamide was obtained as a colorless oil after flash column chromatography on silica gel (719 mg, 84% yield).

IR (neat) ν_{max}: 2920, 1646, 1364, 1279, 1174, 1130, 905, 682;

1H-NMR (400 MHz, CDCl3): δ 7.92 (s, 1H), 7.89 (s, 2H), 3.15 (s, 3H), 2.99 (s, 3H);

13C-NMR (100 MHz, CDCl3): δ 168.4, 138.3, 132.5 (q, J_{CF} = 33.4 Hz), 127.5 (2C), 124.3 (q, J_{CF} = 273.0 Hz), 123.4 (q, J_{CF} = 3.9 Hz), 39.4, 35.5;

19F-NMR (376 MHz, CDCl3): δ -63.0;

HRMS (ESI+): exact mass calculated for $[M+H]^+$ ($C_{11}H_{10}ONF_6$) requires m/z 286.0661, found m/z 286.0660.

Synthesis of *N,N*-dimethyl-4-bromo-benzamide (S5)[2]

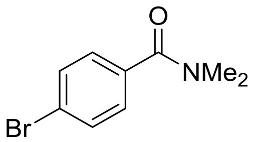

According to the general procedure **A**, using 4-bromobenzoyl chloride (658 mg, 3 mmol) and dimethylamine hydrochloride (269 mg, 3.3 mmol), *N,N*-dimethyl-4-bromo-benzamide was obtained as a white solid after flash column chromatography on silica gel (617 mg, 91% yield).

Synthesis of *N,N*-dimethyl-4-iodo-benzamide (S6)[3]

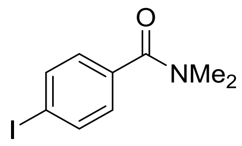

According to the general procedure **A**, using 4-iodobenzoyl chloride (799 mg, 3 mmol) and dimethylamine hydrochloride (269 mg, 3.3 mmol), *N,N*-dimethyl-4-iodo-benzamide was obtained as a white solid after flash column chromatography on silica gel (776 mg, 94% yield).

Synthesis of *N,N*-dimethyl-1-naphthamide (S7)[3]

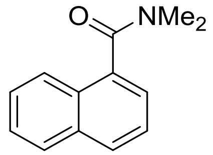

According to the general procedure **A**, using 1-naphthoyl chloride (572 mg, 3 mmol) and dimethylamine hydrochloride (269 mg, 3.3 mmol), *N,N*-dimethyl-1-naphthamide was obtained as a white solid after flash column chromatography on silica gel (776 mg, 94% yield).

Synthesis of *N,N*-dimethylfuran-2-carboxamide (S8)[4]

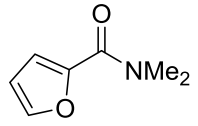

According to the general procedure **A**, using 2-furoyl chloride (295 µL, 3 mmol) and dimethylamine hydrochloride (270 mg, 3.3 mmol), *N,N*-dimethylfuran-2-carboxamide was obtained as a colorless oil after flash column chromatography on silica gel (356 mg, 85% yield).

Synthesis of (6,7-dimethoxy-3,4-dihydroisoquinolin-2(1*H*)-yl)(phenyl)methanone (S9)[5]

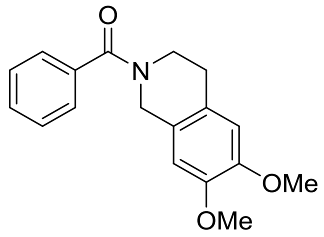

According to the general procedure **A**, using benzoyl chloride (422 mg, 3 mmol) and 6,7-dimethoxy-1,2,3,4-tetrahydroisoquinoline (638 mg, 3.3 mmol), (6,7-dimethoxy-3,4-dihydroisoquinolin-2(1*H*)-yl)(phenyl)methanone was obtained as a white solid after flash column chromatography on silica gel (794 mg, 89% yield).

Synthesis of *N*-benzoyl-L-proline methyl ester (S10)[6]

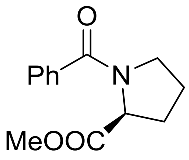

According to the general procedure **A**, using benzoyl chloride (169 mg, 1.2 mmol) and L-proline methyl ester hydrocloride (166 mg, 1 mmol), *N*-benzoyl-L-proline methyl ester was obtained as colorless oil after flash column chromatography on silica gel (191 mg, 82% yield).

Synthesis of 1-(pyrrolidin-1-yl)decan-1-one (S11)[7]

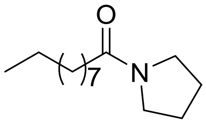

According to the general procedure **A**, using decanoyl chloride (622 µL, 3 mmol) and pyrrolidine (235 mg, 3.3 mmol), 1-(pyrrolidin-1-yl)decan-1-one was obtained as a colorless oil after flash column chromatography on silica gel (602 mg, 89% yield).

Synthesis of *N,N*-dimethylcyclohexanecarboxamide (S12)[8]

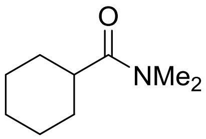

According to the general procedure **A**, using cyclohexyl carbonyl chloride (267 µL, 2 mmol) and dimethylamine hydrochloride (180 mg, 2.2 mmol), *N,N*-dimethylcyclohexylcarboxamide was obtained yield as a colorless oil after flash column chromatography on silica gel (263 mg, 85% yield).

Synthesis of (adamantan-1-yl)(pyrrolidin-1-yl)methanone (S13)[9]

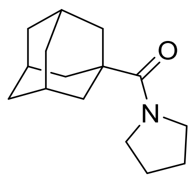

According to the general procedure **A**, using adamantane-1-carbonyl chloride (596 mg, 3 mmol) and pyrrolidine (235 mg, 3.3 mmol), *N,N*-dimethylcyclohexylcarboxamide was obtained yield as a white solid after flash column chromatography on silica gel (574 mg, 82% yield).

Synthesis of *N,N*-dimethyl-3-phenylpropanamide (S14)[10]

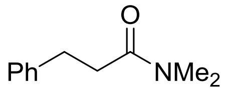

According to the general procedure **A**, using hydrocinnamoyl chloride (506 mg, 3 mmol) and dimethylamine hydrochloride (270 mg, 3.3 mmol), *N,N*-dimethyl-3-phenylpropanamide was obtained as a colorless oil after flash column chromatography on silica gel (479 mg, 90% yield).

Synthesis of 1-(pyrrolidin-1-yl)pent-4-en-1-one (S15)[11]

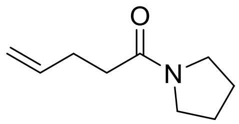

According to the general procedure **B**, using 4-pentenoic acid (300 mg, 3 mmol) and pyrrolidine (235 mg, 3.3 mmol), 1-(pyrrolidin-1-yl)pent-4-en-1-one was obtained as a colorless oil after flash column chromatography on silica gel (377 mg, 82% yield).

Synthesis of (4-iodophenyl)(1,4-dioxa-8-azaspiro[4.5]decan-8-yl)methanone (S16)

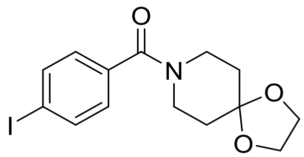

According to the general procedure **A**, using 4-iodobenzoyl chloride (799 mg, 3 mmol) and 1,4-dioxa-8-azaspiro[4.5]decane (473 mg, 3.3 mmol), (4-iodophenyl)(1,4-dioxa-8-azaspiro[4.5]decan-8-yl)methanone was obtained as a white solid after flash column chromatography on silica gel (1052 mg, 94% yield).

M.P. = 116-118 ˚C;

IR (neat) ν_{max}: 2876, 1629, 1587, 1434, 1086, 916;

^{1}H-NMR (400 MHz, $CDCl_3$): δ 7.75 (d, *J* = 8.3 Hz, 2H), 7.14 (d, *J* = 8.2 Hz, 2H), 3.97 (s, 4H), 3.82 (br, 2H), 3.45 (br, 2H), 1.78 (br, 2H), 1.63 (br, 2H);

^{13}C-NMR (100 MHz, $CDCl_3$): δ 169.5, 137.8, 135.5, 128.7, 106.9, 95.9, 64.6, 45.8, 40.5, 35.8, 34.8;

HRMS (ESI+): exact mass calculated for $[M+H]^+$ ($C_{21}H_{28}N$) requires *m/z* 374.0248, found *m/z* 374.0246.

Synthesis of morpholino(4-nitrophenyl)methanone (S17)[12]

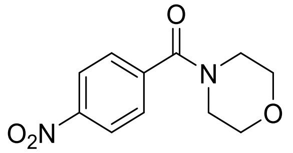

According to the general procedure **A**, using 4-nitrobenzoyl chloride (557 mg, 3 mmol) and morpholine (287 mg, 3.3 mmol), morpholino(4-nitrophenyl)methanone was obtained as a colorless oil after flash column chromatography on silica gel (638 mg, 90% yield).

Synthesis of 2-methoxy-*N,N*-dimethylbenzamide (S18)[13]

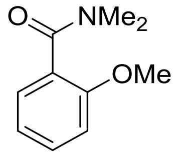

According to the general procedure, using 2-methoxybenzoyl chloride (512 mg, 3 mmol) and dimethylamine hydrochloride (270 mg, 3.3 mmol), 2-methoxy-*N,N*-dimethylbenzamide was obtained as a colorless oil after flash column chromatography on silica gel (441 mg, 82% yield).

Synthesis of *N*-benzyl-*N*-methylbenzamide (S19)[1]

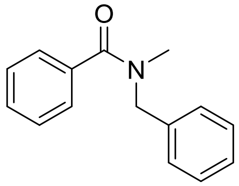

According to the general procedure, using benzoyl chloride (422 mg, 3 mmol) and *N*-methylbenylamine (400 mg, 3.3 mmol), *N*-benzyl-*N*-methylbenzamide was obtained as a colorless oil after flash column chromatography on silica gel (588 mg, 87% yield).

Synthesis of (4-chlorophenyl)(4-cinnamylpiperazin-1-yl)methanone (S20)

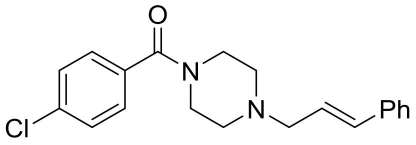

According to the general procedure **A**, using 4-chlorobenzoyl chloride (525 mg, 3 mmol) and *trans*-1-cinnamylpiperazine (668 mg, 3.3 mmol), (4-chlorophenyl)(4-cinnamylpiperazin-1-yl)methanone was obtained as a yellowish solid after flash column chromatography on silica gel (930 mg, 91% yield).

M.P. = 96 ˚C;

IR (neat) ν_{max}: 2807, 1633, 1597, 1431, 1259, 1013, 745;

^{1}H-NMR (400 MHz, $CDCl_3$): δ 7.38-7.05 (m, 6H), 7.33-7.28 (m, 2H), 7.25-7.21 (m, 1H), 6.54 (d, *J* = 15.8 Hz, 1H), 3.29 (dt, *J* = 15.8, 6.8 Hz, 2H), 3.80 (br, 2H), 3.44 (br, 2H), 3.20 (dd, *J* = 6.8, 1.1 Hz, 2H), 2.57 (br, 2H), 2.46 (br, 2H);

^{13}C-NMR (100 MHz, $CDCl_3$): δ 169.3, 136.7, 135.8, 134.2, 133.8, 128.8, 128.7 (2C), 127.8, 126.4, 125.6, 60.9, 53.2, 52.8, 47.7, 42.3;

HRMS (ESI+): exact mass calculated for $[M+H]^+$ ($C_{20}H_{22}N_2OCl$) requires *m/z* 341.1415, found *m/z* 341.1415.

Synthesis of Linoleic carboxamide (S21)

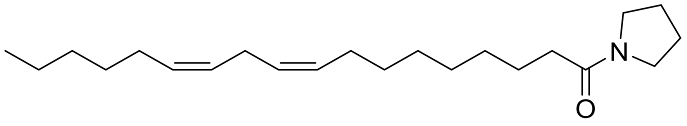

According to the general procedure **B**, using Linoleic acid (841 mg, 3 mmol) and pyrrolidine (235 mg, 3.3 mmol), Linoleic carboxamide was obtained as a colorless oil after flash column chromatography on silica gel (841 mg, 84% yield).

IR (neat) ν_{max}: 2924, 2854, 1645, 1427, 723;

^{1}H-NMR (400 MHz, $CDCl_3$): δ 5.38-5.26 (m, 4H), 3.45 (t, *J* = 6.8 Hz, 2H), 3.39 (t, *J* = 6.8 Hz, 2H), 2.76 (t, *J* = 6.5 Hz, 2H), 2.24 (t, *J* = 7.7 Hz, 2H), 2.04-1.99 (m, 4H), 1.93-1.88 (m, 2H), 1.85-1.78 (m, 2H), 1.63-1.58 (m, 2H), 1.36-1.23 (m, 14H), 0.88 (t, *J* = 7.1 Hz, 3H);

^{13}C-NMR (100 MHz, $CDCl_3$): δ 171.9, 130.3, 130.2, 128.1, 128.0, 46.7, 45.6, 34.9, 31.6, 29.7, 29.6, 29.4 (2C), 29.3, 27.3 (2C), 26.2, 25.7, 25.0, 24.5, 22.6, 14.1;

HRMS (ESI+): exact mass calculated for $[M+H]^+$ ($C_{22}H_{40}NO$) requires *m/z* 334.3104, found *m/z* 334.3103.

Synthesis of Lithocholic carboxamide (S22)

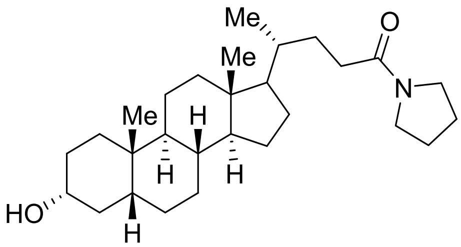

According to the general procedure **B**, using Lithocholic acid (1130 mg, 3 mmol) and pyrrolidine (235 mg, 3.3 mmol), Lithocholic carboxamide was obtained as a white solid after flash column chromatography on silica gel (1031 mg, 80% yield).

M.P. = 136 ˚C;

IR (neat) $ν_{max}$: 3402, 2929, 2864, 1626, 1446, 1042, 733;

^{1}H-NMR (400 MHz, $CDCl_3$): δ 3.58-3.52 (m, 1H), 3.41-3.34 (m, 4H), 2.48 (br, 1H), 2.28-2.21 (m, 1H), 2.12-2.04 (m, 1H), 1.91-1.86 (m, 3H), 1.82-1.66 (m, 7H), 1.61-0.85 (m, 26H), 0.58 (s, 3H);

^{13}C-NMR (100 MHz, $CDCl_3$): δ 172.3, 71.6, 56.5, 56.1, 46.6, 45.7, 42.7, 42.1, 40.4, 40.2, 36.4, 35.8, 35.6, 35.4, 34.6, 31.7, 30.9, 30.5, 28.2, 27.2, 26.4, 26.1, 24.4, 24.2, 23.4, 20.8, 18.5, 12.1;

HRMS (ESI+): exact mass calculated for $[M+H]^+$ ($C_{28}H_{48}NO_2$) requires *m/z* 430.3680, found *m/z* 430.3677.

Synthesis of *N*-(3-(10,11-dihydro-5H-dibenzo[a,d][7]annulen-5-ylidene)propyl)-*N*-methyl-3-phenylpropanamide (S23)

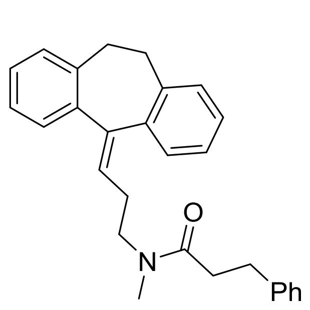

According to the general procedure **A**, using hydrocinnamoyl chloride (506 mg, 3 mmol) and Nortriptyline hydrochloride (989 mg, 3.3 mmol), title compound was obtained as a colorless viscous oil after flash column chromatography on silica gel (1068 mg, 90% yield).

IR (neat) ν_{max}: 2920, 1641, 1485, 1452, 1120, 754, 700;

^{1}H-NMR (400 MHz, $CDCl_3$): δ 7.31-7.06 (m, 13H), 5.89 (t, *J* = 7.5 Hz, 1H), 3.29 (t, *J* = 7.2 Hz, 2H), 2.99 (t, *J* = 8.0 Hz, 2H), 2.93 (t, *J* = 8.0 Hz, 2H), 2.85 (s, 3H), 2.61 (t, *J* = 7.6 Hz, 2H), 2.50-2.46 (m, 2H), 2.40-2.35 (dd, *J* = 14,4, 7.4 Hz, 2H);

^{13}C-NMR (100 MHz, $CDCl_3$): δ 172.0, 146.1, 144.5, 141.6, 141.2, 140.7, 139.5, 137.2, 130.3, 128.8, 128.6, 128.3, 128.0, 127.9, 127.5, 127.3, 126.5, 126.2, 125.9, 49.5, 35.7, 35.0, 33.9, 32.0, 31.7, 28.6;

HRMS (ESI+): exact mass calculated for $[M+H]^+$ ($C_{28}H_{30}NO$) requires *m/z* 396.2322, found *m/z* 396.2321.

Synthesis of Boc-Gly-Sar-OMe (S24)[14]

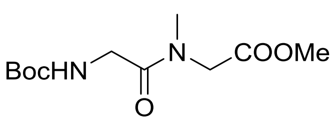

According to the general procedure **B**, using Boc-Gly-OH (526 mg, 3 mmol) and Sarcosine methyl ester hydrochloride (461 mg, 3.3 mmol), Boc-Gly-Sar-OMe was obtained as colorless oil after flash column chromatography on silica gel (609 mg, 78% yield).

Synthesis of [1,1'-biphenyl]-4-yl(pyrrolidin-1-yl)methanone (S25)[15]

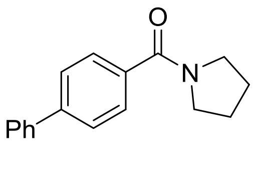

According to the general procedure **B**, using biphenyl-4-carboxylic acid (595 mg, 3 mmol) and pyrrolidine (235 mg, 3.3 mmol), [1,1'-biphenyl]-4-yl(pyrrolidin-1-yl)methanone was obtained as a white solid after flash column chromatography on silica gel (626 mg, 83% yield).

General procedure for the synthesis of α-amino amide from amide

Vaska's catalyst (2.4 mg, 1 mol%) and amide (0.3 mmol, 1.0 eq) was charged into a dry 25 mL flask. Vacuum and N_2 refilling was repeated for three times. Dry DCM (3 mL, 0.1 M) was injected by syringe, then TMDS (0.6 mmol, 2.0 eq) while stirring at room temperature. The result mixture was stirred for 20 mins, following by the addition of isocyanide (0.6 mmol, 2.0 eq) and acetic acid (0.36 mmol, 1.2 eq). The solution was stirred over night at room temperature and was then quenched with saturated aqueous $NaHCO_3$ solution and extracted with DCM (3 x 10 mL). The combined organic layers were dried over Na_2SO_4, filtrated and the solvent was removed under vacuum. The residue was purified by flash column chromatography.

General procedure for the synthesis of α-amino thioamide from amide

Vaska's catalyst (2.4 mg, 1 mol%) and amide (0.3 mmol, 1.0 eq) was charged into a dry 25 mL flask. Vacuum and N_2 refilling was repeated for three times. Dry DCM (3 mL, 0.1 M) was injected by syringe, then TMDS (0.6 mmol, 2.0 eq) while stirring at room temperature. The result mixture was stirred for 20 mins, following by the addition of isocyanide (0.6 mmol, 2.0 eq) and thioacetic acid (0.36 mmol, 1.2 eq). The solution was stirred over night at room temperature and was then quenched with saturated aqueous $NaHCO_3$ solution and extracted with DCM (3 x 10 mL). The combined organic layers were dried over Na_2SO_4, filtrated and the solvent was removed under vacuum. The residue was purified by flash column chromatography.

General procedure for the synthesis of α-amino tetrazole from amide

Vaska's catalyst (2.4 mg, 1 mol%) and amide (0.3 mmol, 1.0 eq) was charged into a dry 25 mL flask. Vacuum and N_2 refilling was repeated for three times. Dry DCM (3 mL, 0.1 M) was injected by syringe, then TMDS (0.6 mmol, 2.0 eq) while stirring at room temperature. The result mixture was stirred for 20 mins,

following by the addition of isocyanide (0.6 mmol, 2.0 eq) and $TMSN_3$ (0.6 mmol, 2.0 eq). The solution was stirred over night at room temperature and was then quenched with saturated aqueous $NaHCO_3$ solution and extracted with DCM (3 x 10 mL). The combined organic layers were dried over Na_2SO_4, filtrated and the solvent was removed under vacuum. The residue was purified by flash column chromatography. Known compounds were consistent with the reported NMR spectrum. Known compounds were consistent with the reported NMR spectrum.

Characterization of α-amino amide, thioamide and tetrazole products

***N*-(*tert*-Butyl)-2-(pyrrolidin-1-yl)-2-(*p*-tolyl)acetamide (2)**

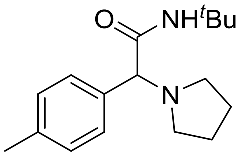

2

According to the general procedure for the synthesis of α-amino amide, pyrrolidin-1-yl(*p*-tolyl)methanone (56.8 mg, 0.3 mmol) and *tert*-butyl isocyanide (49.9 mg, 0.6 mmol) were used, Title compound was obtained as a white solid (64 mg, 78% yield).

M.P. = 119 ˚C;

IR (neat) ν_{max}: 3328, 2965, 2808, 1684, 1664, 1511, 1227, 736;

^{1}H-NMR (400 MHz, $CDCl_3$): δ 7.24-7.22 (m, 2H), 7.11-7.09 (m, 2H), 6.93 (br, 1H), 3.58 (s, 1H), 2.54-2.48 (m, 2H), 2.41-2.36 (m, 2H), 2.31 (s, 3H), 1.76-1.72 (m, 4H), 1.33 (s, 9H);

^{13}C-NMR (100 MHz, $CDCl_3$): δ 171.3, 137.5, 135.8, 129.2, 128.0, 76.2, 52.8, 50.6, 28.8, 23.5, 21.2;

HRMS (ESI+): exact mass calculated for $[M+H]^+$ ($C_{17}H_{27}N_2O$) requires *m/z* 275.2118, found *m/z* 275.2116.

***N*-(*tert*-Butyl)-2-(dimethylamino)-2-phenylacetamide (3)**

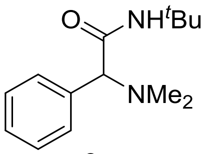

3

According to the general procedure for the synthesis of α-amino amide, *N,N*-dimethylbenzamide (44.8 mg, 0.3 mmol) and *tert*-butyl isocyanide (49.9 mg, 0.6 mmol) were used, Title compound was obtained as a white solid (52 mg, 74% yield).

M.P. = 89-91 ˚C;

IR (neat) ν_{max}: 3283, 2952, 2769, 1649, 1543, 1361, 1257, 729;

^{1}H-NMR (400 MHz, $CDCl_3$): δ 7.24-7.19 (m, 5H), 6.91 (br, 1H), 3.48 (s, 1H), 2.10 (s, 6H), 1.27 (s, 9H);

^{13}C-NMR (100 MHz, $CDCl_3$): δ 170.9, 137.1, 128.7, 128.5, 128.0, 78.0, 50.7, 44.0, 28.8;

HRMS (ESI+): exact mass calculated for $[M+H]^+$ ($C_{14}H_{23}N_2O$) requires *m/z* 235.1805, found *m/z* 235.1802.

***N*-(*tert*-Butyl)-2-(dimethylamino)-2-(4-propoxyphenyl)acetamide (4)**

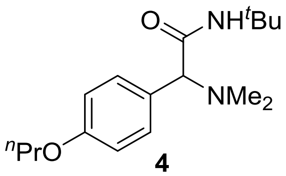

4

According to the general procedure for the synthesis of α-amino amide, *N,N*-dimethyl-4-propoxybenzamide (62.2 mg, 0.3 mmol) and *tert*-butyl isocyanide (49.9 mg, 0.6 mmol) were used, Title compound was obtained as a colorless solid (64 mg, 73% yield).

M.P. = 66-68 ˚C;

IR (neat) ν_{max}: 3320, 2963, 1667, 1509, 1243, 1178, 980, 796;

^{1}H-NMR (400 MHz, $CDCl_3$): δ 7.26-7.17 (m, 2H), 6.95 (br, 1H), 6.85-6.81 (m, 2H), 3.90 (t, *J* = 6.6 Hz, 2H), 3.47 (s, 1H), 2.15 (s, 6H), 1.82-1.74 (m, 2H), 1.34 (s, 9H), 1.03 (t, *J* = 7.4 Hz, 3H);

^{13}C-NMR (100 MHz, $CDCl_3$): δ 171.3, 158.9, 129.7, 129.2, 114.5, 77.3, 69.6, 50.6, 43.9, 28.8, 22.7, 10.6;

HRMS (ESI+): exact mass calculated for $[M+H]^+$ ($C_{17}H_{29}N_2O_2$) requires *m/z* 293.2224, found *m/z* 293.2220.

N-(tert-Butyl)-2-(4-cyanophenyl)-2-(dimethylamino)acetamide (5)

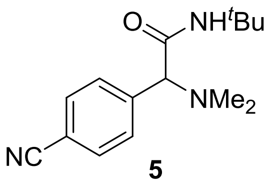

5

According to the general procedure for the synthesis of α-amino amide, *N,N*-dimethyl-4-cyano-benzamide (52.3 mg, 0.3 mmol) and *tert*-butyl isocyanide (49.9 mg, 0.6 mmol) were used, Title compound was obtained as a white solid (63 mg, 81% yield).

M.P. = 146 ˚C;

IR (neat) ν_{max}: 3326, 2961, 2782, 2228, 1661, 1514, 1226, 1040, 797;

^{1}H-NMR (400 MHz, $CDCl_3$): δ 7.62-7.60 (m, 2H), 7.41-7.39 (m, 2H), 6.95 (br, 1H), 3.69 (s, 1H), 2.16 (s, 6H), 1.32 (s, 9H);

^{13}C-NMR (100 MHz, $CDCl_3$): δ 169.4, 141.9, 132.2, 129.7, 118.7, 111.8, 77.0, 51.0, 43.6, 28.7;

HRMS (ESI+): exact mass calculated for $[M+H]^+$ ($C_{15}H_{22}N_3O$) requires *m/z* 260.1757, found *m/z* 260.1758.

2-(3,5-Bis(trifluoromethyl)phenyl)-N-(tert-butyl)-2-(dimethylamino)acetamide (6)

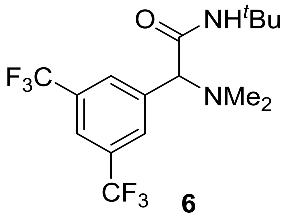

6

According to the general procedure for the synthesis of α-amino amide, *N,N*-dimethyl-3,5-bis(trifluoromethyl)benzamide (85.6 mg, 0.3 mmol) and *tert*-butyl isocyanide (49.9 mg, 0.6 mmol) were used, Title compound was obtained as a colorless crystal (93.3 mg, 84% yield).

M.P. = 128 ˚C;

IR (neat) ν_{max}: 3289, 2990, 1653, 1552, 1276, 1172, 1129, 682;

^{1}H-NMR (400 MHz, $CDCl_3$): δ 7.81 (s, 1H), 7.75 (s, 2H), 7.01 (br, 1H), 3.80 (s, 1H), 2.19 (s, 6H), 1.35 (s, 9H);

^{13}C-NMR (100 MHz, $CDCl_3$): δ 169.1, 139.1, 132.3 (q, J_{CF} = 33.3 Hz), 129.5, 127.4 (q, J_{CF} = 272.2 Hz), 122.2 (q, J_{CF} = 3.9 Hz), 76.6, 51.2, 43.6, 28.7;

^{19}F-NMR (376 MHz, $CDCl_3$): δ -62.9;

HRMS (ESI+): exact mass calculated for $[M+H]^+$ ($C_{16}H_{21}N_2OF_6$) requires *m/z* 371.1553, found *m/z* 371.1544.

2-(4-Bromophenyl)-*N*-(*tert*-butyl)-2-(dimethylamino)acetamide (7)

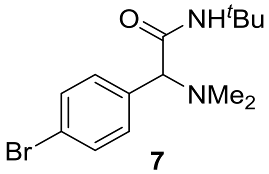

7

According to the general procedure for the synthesis of α-amino amide, *N,N*-dimethyl-4-bromo-benzamide (68.4 mg, 0.3 mmol) and *tert*-butyl isocyanide (49.9 mg, 0.6 mmol) were used, Title compound was obtained as a white solid (75.2 mg, 80% yield).

M.P. = 130-132 ˚C;

IR (neat) ν_{max}: 3323, 2963, 2780, 1658, 1487, 1226, 1011, 792;

^{1}H-NMR (400 MHz, $CDCl_3$): δ 7.45-7.42 (m, 2H), 7.18-7.15 (m, 2H), 6.95 (br, 1H), 3.54 (s, 1H), 2.15 (s, 6H), 1.32 (s, 9H);

^{13}C-NMR (100 MHz, $CDCl_3$): δ 170.3, 135.9, 131.6, 130.4, 122.0, 77.1, 50.8, 43.8, 28.7;

HRMS (ESI+): exact mass calculated for $[M+H]^+$ ($C_{14}H_{22}N_2OBr$) requires *m/z* 313.0910, found *m/z* 313.0909.

***N*-(*tert*-Butyl)-2-(dimethylamino)-2-(4-iodophenyl)acetamide (8)**

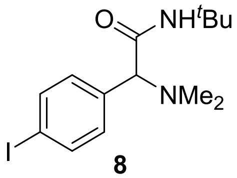

8

According to the general procedure for the synthesis of α-amino amide, *N,N*-dimethyl-4-iodo-benzamide (82.5 mg, 0.3 mmol) and *tert*-butyl isocyanide (49.9 mg, 0.6 mmol) were used, Title compound was obtained as a white solid (83.2 mg, 77% yield).

M.P. = 116-118 ˚C;

IR (neat) ν_{max}: 3336, 2963, 2781, 1663, 1511, 1483, 1363, 1188, 1006, 790;

^{1}H-NMR (400 MHz, $CDCl_3$): δ 7.65-7.62 (m, 2H), 7.05-7.02 (m, 2H), 6.93 (br, 1H), 3.52 (s, 1H), 2.15 (s, 6H), 1.32 (s, 9H);

^{13}C-NMR (100 MHz, $CDCl_3$): δ 170.3, 137.6, 136.6, 130.7, 93.7, 77.2, 50.8, 43.8, 28.7;

HRMS (ESI+): exact mass calculated for $[M+H]^+$ ($C_{14}H_{22}N_2OI$) requires *m/z* 361.0771, found *m/z* 361.0766.

***N*-(*tert*-Butyl)-2-(dimethylamino)-2-(naphthalen-1-yl)acetamide (9)**

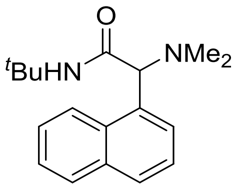

9

According to the general procedure for the synthesis of α-amino amide, *N,N*-dimethyl-1-naphthamide (59.8 mg, 0.3 mmol) and *tert*-butyl isocyanide (49.9 mg, 0.6 mmol) were used, Title compound was obtained as a white solid (56.3 mg, 66% yield).

M.P. = 122-123 ˚C;

IR (neat) ν_{max}: 3335, 2961, 2781, 1663, 1512, 1455, 1227, 776;

^{1}H-NMR (400 MHz, $CDCl_3$): δ 8.37 (d, *J* = 8.7 Hz, 1H), 7.85 (d, *J* = 8.2 Hz, 1H), 7.80 (d, *J* = 8.2 Hz, 1H), 7.61 (dd, *J* = 7.4, 1.0 Hz, 1H), 7.55-7.51 (m, 1H), 7.49-7.45 (m, 2H), 6.99 (br, 1H), 4.48 (s, 1H), 2.29 (s, 6H), 1.34 (s, 9H);

^{13}C-NMR (100 MHz, $CDCl_3$): δ 171.1, 134.2, 134.0, 132.6, 128.9, 128.5, 126.3, 125.8, 125.7, 125.3, 124.1, 72.7, 50.9, 44.2, 28.7;

HRMS (ESI+): exact mass calculated for $[M+H]^+$ ($C_{18}H_{25}N_2O$) requires *m/z* 285.1961, found *m/z* 285.1959.

_N_-(_tert_-Butyl)-2-(dimethylamino)-2-(furan-2-yl)acetamide (10)

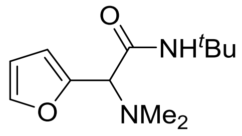

10

According to the general procedure for the synthesis of α-amino amide, *N,N*-dimethylfuran-2-carboxamide (41.8 mg, 0.3 mmol) and *tert*-butyl isocyanide (49.9 mg, 0.6 mmol) were used, Title compound was obtained as yellowish oil (47 mg, 70% yield).

IR (neat) ν_{max}: 3338, 2964, 2784, 1684, 1513, 1455, 1258, 1013, 738;

^{1}H-NMR (400 MHz, $CDCl_3$): δ 7.38-7.37 (m, 1H), 7.13 (br, 1H), 6.32 (dd, *J* = 3.2, 2.0 Hz, 1H), 6.29 (dd, *J* = 3.4, 0.7 Hz, 1H), 3.81 (s, 1H), 2.17 (s, 6H), 1.36 (s, 9H);

^{13}C-NMR (100 MHz, $CDCl_3$): δ 168.5, 150.2, 142.5, 110.3 (2C), 69.8, 50.9, 43.3, 28.8;

HRMS (ESI+): exact mass calculated for $[M+H]^+$ ($C_{12}H_{21}N_2O_2$) requires *m/z* 225.1598, found *m/z* 225.1595.

_N_-(_tert_-Butyl)-2-(6,7-dimethoxy-3,4-dihydroisoquinolin-2(1_H_)-yl)-2-phenylacetamide (11)

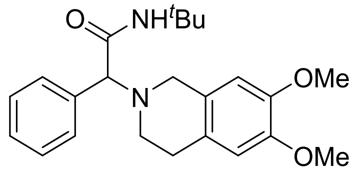

11

According to the general procedure for the synthesis of α-amino amide, (6,7-dimethoxy-3,4-dihydroisoquinolin-2(1*H*)-yl)(phenyl)methanone (89.2 mg, 0.3 mmol) and *tert*-butyl isocyanide (49.9 mg, 0.6 mmol) were used, Title compound was obtained as a yellowish solid (65.4 mg, 57% yield).

M.P. = 148 ˚C;

IR (neat) ν_{max}: 3332, 2961, 1655, 1516, 1494, 1285, 1255, 1125, 737;

^{1}H-NMR (400 MHz, $CDCl_3$): δ 7.38-7.31 (m, 5H), 7.15 (br, 1H), 6.60 (s, 1H), 6.40 (s, 1H), 3.85 (s, 1H), 3.84 (s, 3H), 3.78 (s, 3H), 3.50-3.38 (m, 2H), 2.90-2.75 (m, 3H), 2.67-2.61 (m, 1H), 1.35 (s, 9H);

^{13}C-NMR (100 MHz, $CDCl_3$): δ 170.8, 147.7, 147.4, 136.5, 128.8, 128.7, 128.2, 126.4, 125.8, 111.3, 109.6, 76.5, 56.0, 53.7, 50.8, 50.0, 29.1, 28.8;

HRMS (ESI+): exact mass calculated for $[M+H]^+$ ($C_{23}H_{31}N_2O_3$) requires *m/z* 383.2329, found *m/z* 383.2325.

Methyl ((S)-2-(tert-butylamino)-2-oxo-1-phenylethyl)-L-prolinate (12) [16]

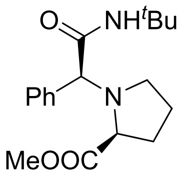

12

According to the general procedure for the synthesis of α-amino amide, *N*-benzoyl-L-proline methyl ester (70 mg, 0.3 mmol) and *tert*-butyl isocyanide (49.9 mg, 0.6 mmol) were used, Title compound was obtained as a colorless viscous oil (37.2 mg, 39% yield). (Major isomer, dr was estimated by ^{1}H analyse of the crude postreaction mixture)

$[\alpha]_D^{25}$ = - 45.5 (c 1.03, $CHCl_3$);

IR (neat) ν_{max}: 3297, 2964, 1735, 1664, 1519, 1454, 1202, 701;

^{1}H-NMR (400 MHz, $CDCl_3$): δ 7.66 (br, 1H), 7.26-7.19 (m, 5H), 4.03 (s, 1H), 3.44 (s, 3H), 3.27 (dd, *J* = 9.6, 4.0 Hz, 1H), 3.20-3.15 (m, 1H), 2.68-2.62 (m, 1H), 2.02-1.93 (m, 1H), 1.87-1.74 (m, 3H), 1.36 (s, 9H);

^{13}C-NMR (100 MHz, $CDCl_3$): δ 176.1, 171.2, 137.4, 129.3, 128.6, 128.4, 74.1, 61.7, 54.5, 51.8, 50.8, 30.9, 28.9, 24.4;

HRMS (ESI+): exact mass calculated for $[M+H]^+$ ($C_{18}H_{27}N_2O_3$) requires *m/z* 319.2016, found *m/z* 319.2013.

***N*-(*tert*-Butyl)-2-(pyrrolidin-1-yl)undecanamide (13)**

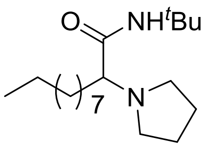

13

According to the general procedure for the synthesis of α-amino amide, 1-(pyrrolidin-1-yl)decan-1-one (67.6 mg, 0.3 mmol) and *tert*-butyl isocyanide (49.9 mg, 0.6 mmol) were used, Title compound was obtained as colorless viscous oil (73.6 mg, 79% yield).

IR (neat) ν_{max}: 3337, 2924, 2854, 1661, 1510, 1228, 721;

^{1}H-NMR (400 MHz, $CDCl_3$): δ 6.52 (br, 1H), 2.55-2.48 (m, 5H), 1.73-1.64 (m, 5H), 1.54-1.45 (m, 1H), 1.32 (s, 9H), 1.22 (brm, 14H), 0.86 (t, *J* = 6.9 Hz, 3H);

^{13}C-NMR (100 MHz, $CDCl_3$): δ 172.9, 70.6, 51.8, 50.4, 32.5, 32.0, 29.9, 29.6, 29.5, 29.4, 28.9, 25.3, 23.4, 22.8, 14.2;

HRMS (ESI+): exact mass calculated for $[M+H]^+$ ($C_{19}H_{39}N_2O$) requires *m/z* 311.3057, found *m/z* 311.3053.

***N*-(*tert*-Butyl)-2-cyclohexyl-2-(dimethylamino)acetamide (14)**

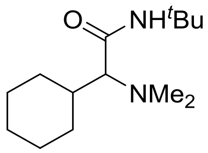

14

According to the general procedure for the synthesis of α-amino amide, *N,N*-dimethylcyclohexanecarboxamide (46.6 mg, 0.3 mmol) and *tert*-butyl isocyanide (49.9 mg, 0.6 mmol) were used, Title compound was obtained as a colorless crystal (36.8 mg, 51% yield).

M.P. = 122-124 ˚C;

IR (neat) ν_{max}: 3324, 2926, 2850, 1640, 1541, 1209, 1035, 660;

^{1}H-NMR (400 MHz, $CDCl_3$): δ 6.13 (br, 1H), 2.28 (d, *J* = 5.6 Hz, 1H), 2.22 (s, 6H), 1.76-1.63 (m, 6H), 1.34 (s, 9H), 1.23-1.05 (m, 4H), 1.01-0.92 (m, 1H);

^{13}C-NMR (100 MHz, $CDCl_3$): δ 170.8, 76.6, 50.9, 43.1, 38.0, 31.0, 29.0, 28.3, 26.9, 26.5;

HRMS (ESI+): exact mass calculated for $[M+H]^+$ ($C_{14}H_{29}N_2O$) requires *m/z* 241.2274, found *m/z* 241.2275.

2-(Adamantan-1-yl)-*N*-(*tert*-butyl)-2-(pyrrolidin-1-yl)acetamide (15)

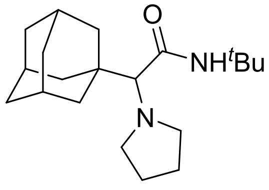

15

According to the general procedure for the synthesis of α-amino amide, (adamantan-1-yl)(pyrrolidin-1-yl)methanone (70 mg, 0.3 mmol) and *tert*-butyl isocyanide (49.9 mg, 0.6 mmol) were used, Title compound was obtained as a white solid (25.8 mg, 27% yield).

M.P. = 78-80 ˚C;

IR (neat) ν_{max}: 3319, 2904, 1671, 1545, 1453, 1225, 734;

^{1}H-NMR (400 MHz, $CDCl_3$): δ 5.97 (br, 1H), 2.81-2.79 (m, 2H), 2.68-2.65 (m, 2H), 2.38 (s, 1H), 1.96 (br, 3H), 1.89-1.86 (m, 4H), 1.73-1.59 (m, 12H), 1.36 (s, 9H);

^{13}C-NMR (100 MHz, $CDCl_3$): δ 170.9, 79.8, 53.7, 51.2, 40.1, 39.4, 37.2, 36.9, 36.1, 29.1, 29.0, 28.4, 23.5;

HRMS (ESI+): exact mass calculated for $[M+H]^+$ ($C_{20}H_{35}N_2O$) requires *m/z* 319.2744, found *m/z* 319.2744.

***N*-(*tert*-Butyl)-2-(dimethylamino)-4-phenylbutanamide (16)**

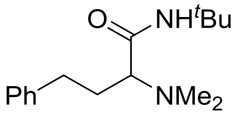

16

According to the general procedure for the synthesis of α-amino amide, *N,N*-dimethyl-3-phenylpropanamide (53.2 mg, 0.3 mmol) and *tert*-butyl isocyanide (49.9 mg, 0.6 mmol) were used, Title compound was obtained as a colorless solid (72.4 mg, 92% yield).

M.P. = 52-54 ˚C;

IR (neat) ν_{max}: 3326, 1671, 1509, 1189, 884, 747;

^{1}H-NMR (400 MHz, $CDCl_3$): δ 7.29-7.25 (m, 2H), 7.21-7.16 (m, 3H), 6.77 (br, 1H), 2.81-2.73 (m, 1H), 2.67-2.58 (m, 2H), 2.24 (s, 6H), 1.94-1.87 (m, 2H), 1.38 (s, 9H);

^{13}C-NMR (100 MHz, $CDCl_3$): δ 172.4, 142.2, 128.5, 128.4, 125.9, 70.0, 50.6, 42.6, 33.0, 30.3, 28.9;

HRMS (ESI+): exact mass calculated for $[M+H]^+$ ($C_{19}H_{39}N_2O$) requires *m/z* 263.2118, found *m/z* 263.2117.

***N*-(*tert*-Butyl)-2-(pyrrolidin-1-yl)hex-5-enamide (17)**

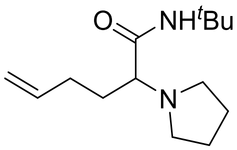

17

According to the general procedure for the synthesis of α-amino amide, 1-(pyrrolidin-1-yl)pent-4-en-1-one (46.0 mg, 0.3 mmol) and *tert*-butyl isocyanide (49.9 mg, 0.6 mmol) were used, Title compound was obtained as a colorless viscous oil (37.9 mg, 53% yield).

IR (neat) $ν_{max}$: 3324, 2964, 2805, 1657, 1511, 1228, 907;

^{1}H-NMR (400 MHz, $CDCl_3$): δ 6.54 (br, 1H), 5.84-5.74 (m, 1H), 5.02-4.91 (m, 2H), 2.61-2.58 (m, 1H), 2.54-2.50 (m, 4H), 2.11-2.03 (m, 2H), 1.86-1.77 (m, 1H), 1.76-1.71 (m, 4H), 1.66-1.57 (m, 1H), 1.33 (s, 9H);

^{13}C-NMR (100 MHz, $CDCl_3$): δ 172.6, 138.4, 114.8, 69.9, 51.7, 50.5, 31.6, 29.8, 28.9, 23.4;

HRMS (ESI+): exact mass calculated for $[M+H]^+$ ($C_{14}H_{27}N_2O$) requires *m/z* 239.2118, found *m/z* 239.2117.

***N*-(*tert*-Butyl)-2-(piperidin-1-yl)acetamide (18)**

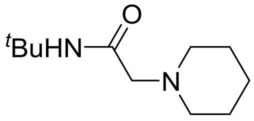

18

According to the general procedure for the synthesis of α-amino amide, 1-formylpiperidine (34.0 mg, 0.3 mmol) and tert-butyl isocyanide (49.9 mg, 0.6 mmol) were used, Title compound was obtained as a colorless viscous oil (52.4 mg, 88% yield).

IR (neat) $ν_{max}$: 3339, 2934, 1678, 1512, 1228, 863;

^{1}H-NMR (400 MHz, $CDCl_3$): δ 7.17 (br, 1H), 2.82 (s, 2H), 2.41 (brm, 4H), 1.59-1.53 (m, 4H), 1.43-1.39 (m, 2H), 1.34 (s, 9H);

^{13}C-NMR (100 MHz, $CDCl_3$): δ 170.0, 63.1, 54.9, 50.4, 28.9, 26.4, 23.9;

HRMS (ESI+): exact mass calculated for $[M+H]^+$ ($C_{19}H_{39}N_2O$) requires *m/z* 199.1805, found *m/z* 199.1806.

***N*-Pentyl-2-(pyrrolidin-1-yl)-2-(*p*-tolyl)acetamide (19)**

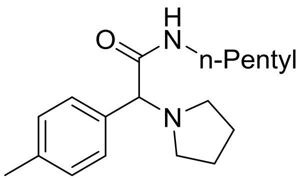

19

According to the general procedure for the synthesis of α-amino amide, pyrrolidin-1-yl(*p*-tolyl)methanone (56.8 mg, 0.3 mmol) and 1-pentyl isocyanide (58.3 mg, 0.6 mmol) were used, Title compound was obtained as a colorless viscous oil (71.0 mg, 82% yield).

IR (neat) ν_{max}: 3295, 2928, 1652, 1511, 1231, 1134, 724;

^{1}H-NMR (400 MHz, $CDCl_3$): δ 7.25 (d, *J* = 8.0 Hz, 2H), 7.10 (d, *J* = 7.9 Hz, 2H), 7.02 (br, 1H), 3.70 (s, 1H), 3.25 (dd, *J* = 13.3, 6.2 Hz, 2H), 2.53-2.48 (m, 2H), 2.43-2.37 (m, 2H), 2.30 (s, 3H), 1.75-1.72 (m, 4H), 1.53-1.45 (m, 2H), 1.32-1.21 (m, 4H), 0.88 (t, *J* = 7.1 Hz, 3H);

^{13}C-NMR (100 MHz, $CDCl_3$): δ 172.0, 137.6, 135.5, 129.2, 128.0, 75.4, 52.8, 39.0, 29.4, 29.2, 23.4, 22.4, 21.2, 14.1;

HRMS (ESI+): exact mass calculated for $[M+H]^+$ ($C_{18}H_{29}N_2O$) requires *m/z* 289.2274, found *m/z* 289.2271.

***N*-(3-Morpholinopropyl)-2-(pyrrolidin-1-yl)-2-(*p*-tolyl)acetamide (20)**

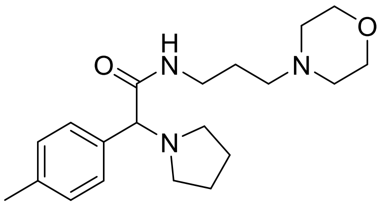

20

According to the general procedure for the synthesis of α-amino amide, pyrrolidin-1-yl(*p*-tolyl)methanone (56.8 mg, 0.3 mmol) and 4-(3-isocyanopropyl)morpholine (92.5 mg, 0.6 mmol) were used, Title compound was obtained as a yellowish viscous oil (93.3 mg, 90% yield).

IR (neat) $ν_{max}$: 3330, 2958, 2808, 1553, 1510, 1272, 1116, 732;

^{1}H-NMR (400 MHz, $CDCl_3$): δ 7.57 (br, 1H), 7.31 (d, *J* = 8.1 Hz, 2H), 7.14 (d, *J* = 7.9 Hz, 2H), 3.78 (t, *J* = 4.7 Hz, 4H), 3.72 (s, 1H), 3.37-3.32 (m, 2H), 2.55-2.52 (m, 2H), 2.47-2.44 (m, 6H), 2.41 (t, *J* = 6.8 Hz, 2H), 2.33 (s, 3H), 1.79-1.76 (m, 4H), 1.72-1.66 (m, 2H);

^{13}C-NMR (100 MHz, $CDCl_3$): δ 172.2, 137.6, 135.7, 129.1, 127.9, 75.9, 67.0, 57.5, 53.9, 52.8, 38.4, 25.7, 23.5, 21.1;

HRMS (ESI+): exact mass calculated for $[M+H]^+$ ($C_{20}H_{32}N_3O_2$) requires *m/z* 346.2489, found *m/z* 346.2489.

Ethyl (2-(pyrrolidin-1-yl)-2-(*p*-tolyl)acetyl)glycinate (21)

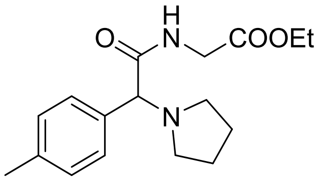

21

According to the general procedure for the synthesis of α-amino amide, pyrrolidin-1-yl(*p*-tolyl)methanone (56.8 mg, 0.3 mmol) and ethyl isocyanoacetate (67.9 mg, 0.6 mmol) were used, Title compound was obtained as a white solid (63.0 mg, 69% yield).

M.P. = 130-132 ˚C;

IR (neat) $ν_{max}$: 3340, 2967, 2801, 1748, 1668, 1510, 1190, 1024, 750;

^{1}H-NMR (400 MHz, $CDCl_3$): δ 7.57 (br, 1H), 7.29 (d, *J* = 8.1 Hz, 2H), 7.12 (d, *J* = 7.9 Hz, 2H), 4.22-4.11 (m, 3H), 3.97 (dd, *J* = 18.3, 5.1 Hz, 1H), 3.78 (s, 1H), 2.61-2.58 (m, 2H), 2.44-2.41 (m, 2H), 2.30 (s, 3H), 1.77-1.74 (m, 4H), 1.28 (t, *J* = 7.1 Hz, 3H);

^{13}C-NMR (100 MHz, $CDCl_3$): δ 172.5, 170.2, 137.8, 135.0, 129.2, 128.2, 75.0, 61.5, 52.7, 40.9, 23.5, 21.2, 14.2;

HRMS (ESI+): exact mass calculated for $[M+H]^+$ ($C_{17}H_{25}N_2O_3$) requires *m/z* 305.1860, found *m/z* 305.1856.

2-(Pyrrolidin-1-yl)-*N*-((tetrahydrofuran-2-yl)methyl)-2-(*p*-tolyl)acetamide (22)

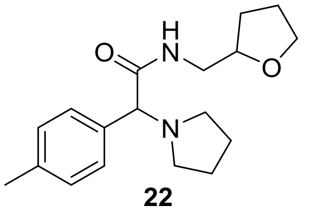

22

According to the general procedure for the synthesis of α-amino amide, pyrrolidin-1-yl(*p*-tolyl)methanone (56.8 mg, 0.3 mmol) and 2-(isocyanomethyl)tetrahydrofuran (66.7 mg, 0.6 mmol) were used, Title compound was obtained as a colorless viscous oil (72.6 mg, 80% yield).

IR (neat) ν_{max}: 3250, 2966, 2802, 1668, 1510, 1194, 1073, 737;

^{1}H-NMR (400 MHz, $CDCl_3$): δ 7.30-7.27 (m, 3H), 7.12 (d, *J* = 7.9 Hz, 2H), 3.97-3.84 (m, 2H), 3.80-3.74 (m, 2H), 3.57-3.48 (m, 1H), 2.56-2.52 (m, 2H), 2.45-2.40 (m, 2H), 2.32 (s, 3H), 1.96-1.74 (m, 8H), 1.56-1.35 (m, 1H);

^{13}C-NMR (100 MHz, $CDCl_3$): δ 172.4, 137.6, 135.6 (135.5), 129.2 (129.1), 128.1 (128.0), 78.1 (78.0), 75.6 (75.4), 68.2, 52.8 (52.7), 42.7 (42.3), 28.7 (28.3), 25.9, 23.5, 21.2;

HRMS (ESI+): exact mass calculated for $[M+H]^+$ ($C_{18}H_{27}N_2O_2$) requires *m/z* 303.2067, found *m/z* 303.2066.

***N*-Cyclohexyl-2-(pyrrolidin-1-yl)-2-(*p*-tolyl)acetamide (23)**

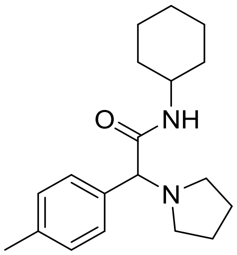

23

According to the general procedure for the synthesis of α-amino amide, pyrrolidin-1-yl(*p*-tolyl)methanone (56.8 mg, 0.3 mmol) and cyclohexyl isocyanide (65.5 mg, 0.6 mmol) were used, Title compound was obtained as a white solid (73.0 mg, 81% yield).

M.P. = 136 ˚C;

IR (neat) ν_{max}: 3296, 2929, 2797, 1650, 1510, 1195, 738;

^{1}H-NMR (400 MHz, $CDCl_3$): δ 7.25 (d, *J* = 8.1 Hz, 2H), 7.10 (d, *J* = 7.9 Hz, 2H), 6.93 (d, *J* = 8.8 Hz, 1H), 3.80-3.70 (m, 1H), 3.67 (s, 1H), 2.54-2.49 (m, 2H), 2.42-2.36 (m, 2H), 2.30 (s, 3H), 1.95-1.91 (m, 1H), 1.81-1.58 (m, 8H), 1.42-1.30 (m, 2H), 1.26-1.13 (m, 3H);

^{13}C-NMR (100 MHz, $CDCl_3$): δ 171.0, 137.6, 135.7, 129.2, 128.0, 75.6, 52.8, 47.4, 33.3, 33.1, 25.7, 24.9 (2C), 23.5, 21.2;

HRMS (ESI+): exact mass calculated for $[M+H]^+$ ($C_{19}H_{29}N_2O$) requires *m/z* 301.2274, found *m/z* 301.2275.

***N*-Benzyl-2-(pyrrolidin-1-yl)-2-(*p*-tolyl)acetamide (24)**

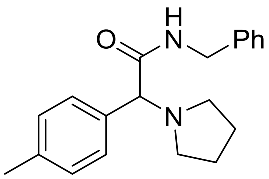

24

According to the general procedure for the synthesis of α-amino amide, pyrrolidin-1-yl(*p*-tolyl)methanone (56.8 mg, 0.3 mmol) and benzyl isocyanide (70.3 mg, 0.6 mmol) were used, Title compound was obtained as a white solid (80.5 mg, 87% yield).

M.P. = 88-90 ˚C;

IR (neat) ν_{max}: 3309, 3029, 2801, 1655, 1510, 1194, 698;

^{1}H-NMR (400 MHz, $CDCl_3$): δ 7.39 (br, 1H), 7.33-7.25 (m, 5H), 7.22-7.20 (m, 2H), 7.13 (d, *J* = 7.9 Hz, 2H), 4.50-4.39 (m, 2H), 3.84 (s, 1H), 2.54-2.50 (m, 2H), 2.45-2.41 (m, 2H), 2.33 (s, 3H), 1.74-1.70 (m, 4H);

^{13}C-NMR (100 MHz, $CDCl_3$): δ 172.1, 138.5, 137.8, 135.2, 129.2, 128.7, 128.1, 127.8, 127.4, 75.3, 52.7, 43.2, 23.4, 21.2;

HRMS (ESI+): exact mass calculated for $[M+H]^+$ ($C_{20}H_{25}N_2O$) requires *m/z* 309.1961, found *m/z* 309.1960.

(*N*-((*S*)-1-Phenylethyl)-2-(pyrrolidin-1-yl)-2-(*p*-tolyl)acetamide (25)

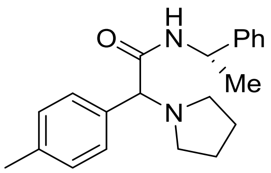

25

According to the general procedure for the synthesis of α-amino amide, pyrrolidin-1-yl(*p*-tolyl)methanone (56.8 mg, 0.3 mmol) and (*S*)-(−)-α-methylbenzyl isocyanide (78.7 mg, 0.6 mmol) were used, Title compound was obtained as a white solid (77.4 mg, 80% yield).

M.P. = 104-106 ˚C;

IR (neat) ν_{max}: 3290, 2930, 1652, 1511, 1134, 724;

^{1}H-NMR (400 MHz, $CDCl_3$): δ 7.30-7.19 (m, 8H), 7.09 (d, *J* = 7.9 Hz, 2H), 5.18-5.10 (m, 1H), 3.76 (s, 1H), 2.57-2.52 (m, 2H), 2.47-2.41 (m, 2H), 2.31 (s, 3H), 1.77-1.74 (m, 4H), 1.55 (d, *J* = 6.9 Hz, 3H);

^{13}C-NMR (100 MHz, $CDCl_3$): δ 171.1, 143.1, 137.6, 135.2, 129.3, 129.1, 128.5, 128.4, 128.0, 127.1, 126.3, 126.2, 75.4, 53.1, 52.7, 51.8, 47.7, 23.4, 23.1, 21.5, 21.1;

HRMS (ESI+): exact mass calculated for $[M+H]^+$ ($C_{21}H_{27}N_2O$) requires *m/z* 323.2118, found *m/z* 323.2120.

***N*-(Naphthalen-2-yl)-2-(pyrrolidin-1-yl)-2-(*p*-tolyl)acetamide (26)**

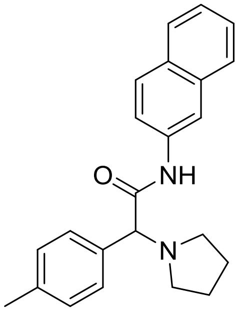

26

According to the general procedure for the synthesis of α-amino amide, pyrrolidin-1-yl(*p*-tolyl)methanone (56.8 mg, 0.3 mmol) and 2-naphthyl isocyanide (91.9 mg, 0.6 mmol) were used, Title compound was obtained as a yellowish solid (55.8 mg, 54% yield).

M.P. = 126 ˚C;

IR (neat) ν_{max}: 3311, 2964, 2808, 1671, 1527, 1499, 1188, 745;

^{1}H-NMR (400 MHz, $CDCl_3$): δ 9.28 (br, 1H), 8.25 (d, *J* = 2.2 Hz, 1H), 7.81 (t, *J* = 8.8 Hz, 3H), 7.60 (dd, *J* = 8.8, 2.2 Hz, 1H), 7.47-7.36 (m, 4H), 7.17 (d, *J* = 7.8 Hz, 2H), 3.98 (s, 1H), 2.66 (br, 2H), 2.59 (br, 2H), 2.33 (s, 3H), 1.87-1.83 (m, 4H);

^{13}C-NMR (100 MHz, $CDCl_3$): δ 170.4, 138.1, 135.4, 134.0, 130.7, 129.4, 128.8, 128.2, 127.7, 127.6, 126.5, 125.0, 119.8, 116.3, 75.6, 52.8, 23.6, 21.2;

HRMS (ESI+): exact mass calculated for $[M+H]^+$ ($C_{23}H_{25}N_2O$) requires *m/z* 345.1961, found *m/z* 345.1965.

2-(Pyrrolidin-1-yl)-*N*-(1,2,3,6-tetrahydro-[1,1'-biphenyl]-4-yl)-2-(*p*-tolyl)acetamide (27)

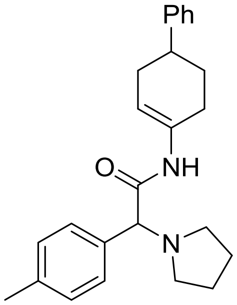

27

According to the general procedure for the synthesis of α-amino amide, pyrrolidin-1-yl(*p*-tolyl)methanone (56.8 mg, 0.3 mmol) and (4-isocyano-3-cyclohexen-1-yl)benzene (110 mg, 0.6 mmol) were used, Title compound was obtained as a white solid (84.3 mg, 75% yield).

M.P. = 78-80 ˚C;

IR (neat) ν_{max}: 3280, 3026, 2835, 1671, 1509, 1195, 731, 699;

^{1}H-NMR (400 MHz, $CDCl_3$): δ 8.16 (br, 1H), 7.33-7.20 (m, 7H), 7.15 (d, *J* = 7.8 Hz, 2H), 6.19 (s, 1H), 3.78 (s, 1H), 2.80 (br, 1H), 2.57 (br, 2H), 2.49 (br, 3H), 2.34 (s, 3H), 2.29-2.20 (m, 2H), 2.03-1.99 (m, 1H), 1.93-1.79 (m, 6H);

^{13}C-NMR (100 MHz, $CDCl_3$): δ 170.2, 146.5, 137.8, 135.0, 132.3 (132.2), 129.3, 128.5, 128.1, 126.9, 126.2, 112.0 (111.8), 75.7, 52.7, 39.8 (39.7), 32.3, 29.5, 28.7, 23.5, 21.2;

HRMS (ESI+): exact mass calculated for $[M+H]^+$ ($C_{25}H_{31}N_2O$) requires *m/z* 375.2431, found *m/z* 375.2427.

N-(tert-Butyl)-2-(pyrrolidin-1-yl)undecanethioamide (28)

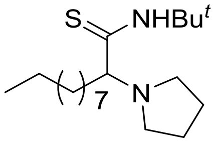

28

According to the general procedure for the synthesis of α-amino thioamide, 1-(pyrrolidin-1-yl)decan-1-one (67.6 mg, 0.3 mmol) and *tert*-butyl isocyanide (49.9 mg, 0.6 mmol) were used, Title compound was obtained as a colorless crystal (49.0 mg, 50% yield).

M.P. = 113-115 ˚C;

IR (neat) ν_{max}: 3236, 2923, 2853, 1515, 1407, 1384, 1215, 722;

^{1}H-NMR (400 MHz, $CDCl_3$): δ 8.83 (br, 1H), 3.14-3.12 (m, 1H), 2.55-2.49 (m, 4H), 1.94-1.89 (m, 1H), 1.75 (br, 4H), 1.66-1.60 (m, 1H), 1.54 (s, 9H), 1.24 (brm, 14H), 0.88 (t, *J* = 6.9 Hz, 3H);

^{13}C-NMR (100 MHz, $CDCl_3$): δ 201.7, 79.0, 54.7, 51.6, 34.2, 32.0, 30.0, 29.7, 29.6, 29.4, 27.8, 24.3, 23.4, 22.8, 14.2;

HRMS (ESI+): exact mass calculated for $[M+H]^+$ ($C_{19}H_{39}N_2S$) requires *m/z* 327.2829, found *m/z* 327.2827.

N-(tert-Butyl)-2-(dimethylamino)-4-phenylbutanethioamide (29)

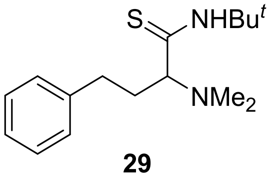

29

According to the general procedure for the synthesis of α-amino thioamide, *N,N*-dimethyl-3-phenylpropanamide (53.2 mg, 0.3 mmol) and *tert*-butyl isocyanide (49.9 mg, 0.6 mmol) were used, Title compound was obtained as a yellowish viscous oil (36.0 mg, 43% yield).

IR (neat) ν_{max}: 3219, 2958, 2785, 1699, 1514, 1362, 1214, 699;

^{1}H-NMR (400 MHz, $CDCl_3$): δ 8.73 (br, 1H), 7.08-7.04 (m, 2H), 6.99-6.95 (m, 3H), 2.88-2.85 (m, 1H), 2.39-2.31 (m, 2H), 2.06 (s, 6H), 2.03-1.95 (m, 1H), 1.80-1.71 (m, 1H), 1.38 (s, 9H);

^{13}C-NMR (100 MHz, $CDCl_3$): δ 201.3, 141.9, 128.5, 128.4, 126.1, 79.6, 54.8, 43.2, 34.3, 31.2, 27.7;

HRMS (ESI+): exact mass calculated for $[M+H]^+$ ($C_{16}H_{27}N_2S$) requires *m/z* 279.1890, found *m/z* 279.1890.

***N*-(*tert*-Butyl)-2-(pyrrolidin-1-yl)hex-5-enethioamide (30)**

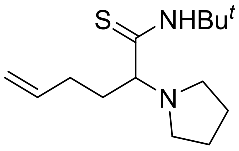

30

According to the general procedure for the synthesis of α-amino thioamide, 1-(pyrrolidin-1-yl)pent-4-en-1-one (46.0 mg, 0.3 mmol) and *tert*-butyl isocyanide (49.9 mg, 0.6 mmol) were used, Title compound was obtained as a yellowish oil (39.7 mg, 52% yield).

IR (neat) ν_{max}: 3237, 2962, 2808, 1680, 1516, 1385, 1214, 909;

^{1}H-NMR (400 MHz, $CDCl_3$): δ 8.80 (br, 1H), 5.84-5.74 (m, 1H), 5.03-4.93 (m, 2H), 3.16-3.14 (m, 1H), 2.55-2.50 (m, 4H), 2.06-1.97 (m, 3H), 1.76-1.71 (m, 5H), 1.55 (s, 9H);

^{13}C-NMR (100 MHz, $CDCl_3$): δ 201.4, 138.3, 114.9, 78.5, 54.8, 51.6, 33.5, 28.7, 27.8, 23.4;

HRMS (ESI+): exact mass calculated for $[M+H]^+$ ($C_{14}H_{27}N_2S$) requires *m/z* 255.1890, found *m/z* 255.1889.

***N*-(*tert*-Butyl)-2-(piperidin-1-yl)ethanethioamide (31)**

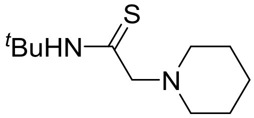

31

According to the general procedure for the synthesis of α-amino thioamide, 1-formylpiperidine (34.0 mg, 0.3 mmol) and *tert*-butyl isocyanide (49.9 mg, 0.6 mmol) were used, Title compound was obtained as a yellowish oil (23,8 mg, 37% yield).

IR (neat) ν_{max}: 3201, 2935, 1519, 1406, 1248, 991;

^{1}H-NMR (400 MHz, $CDCl_3$): δ 9.46 (br, 1H), 3.29 (s, 2H), 2.44 (brm, 4H), 1.56 (brm, 13H), 1.46-1.44 (m, 2H);

^{13}C-NMR (100 MHz, $CDCl_3$): δ 197.0, 71.6, 54.6, 54.0, 27.8, 26.5, 23.8;

HRMS (ESI+): exact mass calculated for $[M+H]^+$ ($C_{11}H_{23}N_2S$) requires *m/z* 215.1577, found *m/z* 215.1578.

1-(*tert*-Butyl)-5-(pyrrolidin-1-yl(*p*-tolyl)methyl)-1H-tetrazole (32)

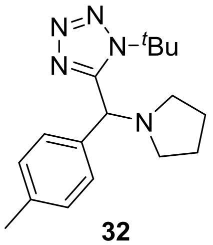

32

According to the general procedure for the synthesis of α-amino tetrazole, pyrrolidin-1-yl(*p*-tolyl)methanone (56.8 mg, 0.3 mmol) and *tert*-butyl isocyanide (49.9 mg, 0.6 mmol) were used, Title compound was obtained as a white solid (88.0 mg, 98% yield).

M.P. = 108-110 ˚C;

IR (neat) ν_{max}: 2973, 2802, 1514, 1375, 1107, 795, 733;

^{1}H-NMR (400 MHz, $CDCl_3$): δ 7.32-7.30 (m, 2H), 7.09-7.07 (m, 2H), 5.09 (s, 1H), 2.57-2.52 (m, 2H), 2.50-2.47 (m, 2H), 2.27 (s, 3H), 1.72 (brm, 4H), 1.62 (s, 9H);

^{13}C-NMR (100 MHz, $CDCl_3$): δ 155.4, 138.2, 133.8, 129.3, 129.2, 63.8, 61.3, 51.8, 30.2, 23.3, 21.1;

HRMS (ESI+): exact mass calculated for $[M+H]^+$ ($C_{17}H_{26}N_5$) requires *m/z* 300.2183, found *m/z* 300.2181.

1-(1-(*tert*-Butyl)-1*H*-tetrazol-5-yl)-*N,N*-dimethyl-1-phenylmethanamine (33)

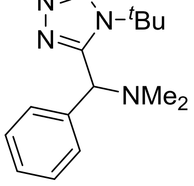

33

According to the general procedure for the synthesis of α-amino tetrazole, *N,N*-dimethylbenzamide (44.8 mg, 0.3 mmol) and *tert*-butyl isocyanide (49.9 mg, 0.6 mmol) were used, Title compound was obtained as a white solid (73.1 mg, 94% yield).

M.P. = 100-102 ˚C;

IR (neat) ν_{max}: 2939, 2782, 1452, 1403, 1238, 1022, 744, 701;

^{1}H-NMR (400 MHz, $CDCl_3$): δ 7.41-7.38 (m, 2H), 7.35-7.29 (m, 3H), 5.18 (s, 1H), 2.31 (s, 6H), 1.67 (s, 9H);

^{13}C-NMR (100 MHz, $CDCl_3$): δ 154.5, 135.8, 129.7, 128.6, 128.5, 65.5, 61.4, 42.6, 30.2;

HRMS (ESI+): exact mass calculated for $[M+H]^+$ ($C_{14}H_{22}N_5$) requires *m/z* 260.1870, found *m/z* 260.1870.

1-(1-(*tert*-Butyl)-1*H*-tetrazol-5-yl)-*N,N*-dimethyl-1-(4-propoxyphenyl)methanamine (34)

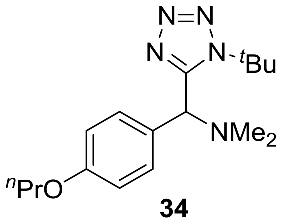

34

According to the general procedure for the synthesis of α-amino tetrazole, *N,N*-dimethyl-4-propoxybenzamide (62.2 mg, 0.3 mmol) and *tert*-butyl isocyanide (49.9 mg, 0.6 mmol) were used, Title compound was obtained as colorless viscous oil (91.4 mg, 96% yield).

IR (neat) ν_{max}: 2937, 2781, 1610, 1510, 1243, 1020, 806;

^{1}H-NMR (400 MHz, $CDCl_3$): δ 7.18-7.16 (m, 2H), 6.71-6.69 (m, 2H), 4.97 (s, 1H), 3.76 (t, *J* = 6.6 Hz, 2H), 2.16 (s, 6H), 1.68-1.59 (m, 2H), 1.53 (s, 9H), 0.89 (t, *J* = 7.4 Hz, 3H);

^{13}C-NMR (100 MHz, $CDCl_3$): δ 159.2, 154.8, 130.9, 127.6, 114.4, 69.5, 65.0, 61.3, 42.6, 30.2, 22.6, 10.5;

HRMS (ESI+): exact mass calculated for $[M+H]^+$ ($C_{17}H_{28}N_5O$) requires *m/z* 318.2288, found *m/z* 318.2288.

1-(4-Bromophenyl)-1-(1-(*tert*-butyl)-1*H*-tetrazol-5-yl)-*N,N*-dimethylmethanamine (35)

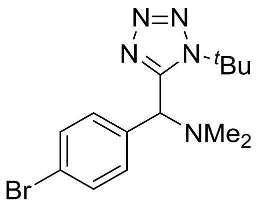

35

According to the general procedure for the synthesis of α-amino tetrazole, *N,N*-dimethyl-4-bromo-benzamide (68.4 mg, 0.3 mmol) and *tert*-butyl isocyanide (49.9 mg, 0.6 mmol) were used, Title compound was obtained as a white solid (94.4 mg, 93% yield).

M.P. = 119 ˚C;

IR (neat) ν_{max}: 2987, 2782, 1486, 1216, 803, 735;

^{1}H-NMR (400 MHz, $CDCl_3$): δ 7.44-7.42 (m, 2H), 7.29-7.27 (m, 2H), 5.13 (s, 1H), 2.25 (s, 6H), 1.65 (s, 9H);

^{13}C-NMR (100 MHz, $CDCl_3$): δ 154.0, 134.8, 131.7, 131.4, 122.7, 64.7, 61.5, 42.4, 30.2;

HRMS (ESI+): exact mass calculated for $[M+H]^+$ ($C_{14}H_{21}N_5Br$) requires *m*/*z* 338.0975, found *m*/*z* 338.0975.

1-(1-(*tert*-Butyl)-1H-tetrazol-5-yl)-1-(4-iodophenyl)-*N,N*-dimethylmethanamine (36)

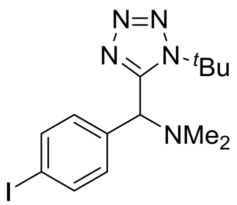

36

According to the general procedure for the synthesis of α-amino tetrazole, *N,N*-dimethyl-4-iodo-benzamide (82.5 mg, 0.3 mmol) and *tert*-butyl isocyanide (49.9 mg, 0.6 mmol) were used, Title compound was obtained as white solid (111 mg, 96% yield).

M.P. = 126 ˚C;

IR (neat) ν_{max}: 2988, 2781, 1585, 1483, 1185, 1006, 800;

^{1}H-NMR (400 MHz, $CDCl_3$): δ 7.64-7.62 (m, 2H), 7.16-7.14 (m, 2H), 5.11 (s, 1H), 2.25 (s, 6H), 1.65 (s, 9H);

^{13}C-NMR (100 MHz, $CDCl_3$): δ 154.0, 137.6, 135.5, 131.6, 94.5, 64.8, 61.5, 42.4, 30.2;

HRMS (ESI+): exact mass calculated for $[M+H]^+$ ($C_{14}H_{21}N_5I$) requires *m/z* 386.0836, found *m/z* 386.0832.

8-((1-(*tert*-Butyl)-1*H*-tetrazol-5-yl)(4-iodophenyl)methyl)-1,4-dioxa-8-azaspiro[4.5]decane (37)

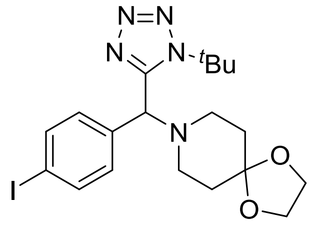

37

According to the general procedure for the synthesis of α-amino tetrazole, (4-iodophenyl)(1,4-dioxa-8-azaspiro[4.5]decan-8-yl)methanone (112 mg, 0.3 mmol) and *tert*-butyl isocyanide (49.9 mg, 0.6 mmol) were used, Title compound was obtained as a white solid (136 mg, 94% yield).

M.P. = 133-135 ˚C;

IR (neat) ν_{max}: 2955, 2832, 1586, 1485, 1215, 1077, 1007, 735;

^{1}H-NMR (400 MHz, $CDCl_3$): δ 7.64-7.62 (m, 2H), 7.21-7.19 (m, 2H), 5.17 (s, 1H), 3.86 (s, 4H), 2.71-2.66 (m, 2H), 2.47-2.41 (m, 2H), 1.68 (m, 13H);

^{13}C-NMR (100 MHz, $CDCl_3$): δ 154.0, 137.7, 135.1, 131.7, 106.8, 94.6, 64.4, 64.3, 61.6, 48.5, 35.1, 30.3;

HRMS (ESI+): exact mass calculated for $[M+H]^+$ ($C_{19}H_{27}N_5O_2I$) requires *m/z* 484.1204, found *m/z* 484.1198.

4-((1-(*tert*-Butyl)-1*H*-tetrazol-5-yl)(4-nitrophenyl)methyl)morpholine (38)

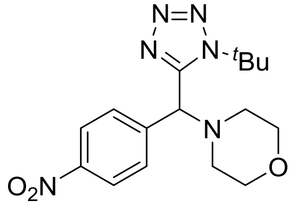

38

According to the general procedure for the synthesis of α-amino tetrazole, morpholino(4-nitrophenyl)methanone (70.9 mg, 0.3 mmol) and *tert*-butyl isocyanide (49.9 mg, 0.6 mmol) were used, Title compound was obtained as a yellowish solid (83.1 mg, 80% yield).

M.P. = 142 ˚C;

IR (neat) ν_{max}: 2858, 1607, 1522, 1347, 1114, 733;

^{1}H-NMR (400 MHz, $CDCl_3$): δ 8.20-8.18 (m, 2H), 7.71-7.69 (m, 2H), 5.36 (s, 1H), 3.66 (t, *J* = 4.7 Hz, 4H), 2.69-2.64 (m, 2H), 2.44-2.38 (m, 2H), 1.72 (s, 9H);

^{13}C-NMR (100 MHz, $CDCl_3$): δ 152.9, 148.1, 142.2, 130.9, 123.8, 67.0, 64.5, 61.9, 50.8, 30.5;

HRMS (ESI+): exact mass calculated for $[M+H]^+$ ($C_{16}H_{23}N_6O_3$) requires *m/z* 347.1826, found *m/z* 347.1827.

1-(1-(*tert*-Butyl)-1*H*-tetrazol-5-yl)-1-(2-methoxyphenyl)-*N,N*-dimethylmethanamine (39)

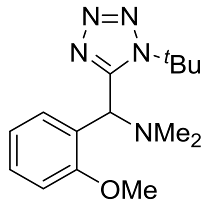

39

According to the general procedure for the synthesis of α-amino tetrazole, 2-methoxy-*N,N*-dimethylbenzamide (53.8 mg, 0.3 mmol) and *tert*-butyl isocyanide (49.9 mg, 0.6 mmol) were used, Title compound was obtained as a colorless viscous oil (40.8 mg, 47% yield).

IR (neat) ν_{max}: 2940, 2781, 1600, 1492, 1243, 1024, 757;

^{1}H-NMR (400 MHz, $CDCl_3$): δ 7.53 (dd, *J* = 7.9, 1.8 Hz, 1H), 7.29-7.25 (m, 1H), 6.93-6.89 (m, 2H), 5.83 (s, 1H), 3.90 (s, 3H), 2.35 (s, 6H), 1.63 (s, 9H);

^{13}C-NMR (100 MHz, $CDCl_3$): δ 156.8, 155.0, 131.6, 129.7, 124.0, 120.9, 110.8, 61.3, 56.2, 55.8, 42.6, 29.9;

HRMS (ESI+): exact mass calculated for $[M+H]^+$ ($C_{15}H_{24}N_5O$) requires *m/z* 290.1975, found *m/z* 290.1975.

***N*-Benzyl-1-(1-(*tert*-butyl)-1*H*-tetrazol-5-yl)-*N*-methyl-1-phenylmethanamine (40)**

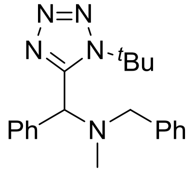

40

According to the general procedure for the synthesis of α-amino tetrazole, *N*-benzyl-*N*-methylbenzamide (67.6 mg, 0.3 mmol) and *tert*-butyl isocyanide (49.9 mg, 0.6 mmol) were used, Title compound was obtained as a white solid (93.6 mg, 93% yield).

M.P. = 98-100 ˚C;

IR (neat) ν_{max}: 2988, 1494, 1452, 1217, 1025, 739, 699;

^{1}H-NMR (400 MHz, $CDCl_3$): δ 7.38-7.23 (m, 10H), 5.64 (s, 1H), 3.85 (dd, *J* = 17.2, 3.4 Hz, 2H), 2.40 (s, 3H), 1.54 (s, 9H);

^{13}C-NMR (100 MHz, $CDCl_3$): δ 154.0, 139.3, 136.8, 129.5, 128.7, 128.6, 128.4 (2C), 127.1, 62.6, 61.5, 57.6, 39.0, 30.0;

HRMS (ESI+): exact mass calculated for $[M+H]^+$ ($C_{20}H_{26}N_5$) requires *m/z* 336.2183, found *m/z* 336.2182.

Methyl ((1-(*tert*-butyl)-1*H*-tetrazol-5-yl)(phenyl)methyl)-L-prolinate (41)

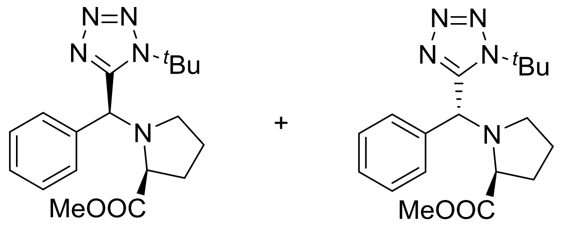

2 : 1

41

According to the general procedure for the synthesis of α-amino tetrazole, *N*-benzoyl-L-proline methyl ester (70 mg, 0.3 mmol) and *tert*-butyl isocyanide (49.9 mg, 0.6 mmol) were used, Title compound was obtained as a colorless viscous oil (77.3 mg, 75% yield). (dr was estimated by ^{1}H analyse of the crude postreaction mixture.)

Major isomer: $[\alpha]_D^{25}$ = - 105.6 (c 1.03, $CHCl_3$); Minor isomer: $[\alpha]_D^{25}$ = - 30.2 (c 1.03, $CHCl_3$);

IR (neat) ν_{max}: 2988, 1737, 1494, 1200, 1003, 734, 701;

^{1}H-NMR (400 MHz, $CDCl_3$): δ major isomer: 7.30-7.22 (m, 5H), 5.95 (s, 1H), 3.65 (s, 3H), 3.49-3.43 (m, 1H), 3.41-3.36 (m, 1H), 2.73-2.68 (m, 1H), 2.18-2.09 (m, 1H), 2.00-1.92 (m, 1H), 1.86-1.78 (m, 1H), 1.74-1.67

(m, 1H), 1.53 (s, 9H); minor isomer: 7.38-7.35 (m, 2H), 7.32-7.27 (m, 3H), 5.92 (s, 1H), 3.95 (dd, *J* = 9.3, 3.2 Hz, 1H), 3.46 (s, 3H), 3.23-3.18 (m, 1H), 2.70 (dd, *J* = 15.0, 7.4 Hz, 1H), 2.24-2.14 (m, 1H), 1.95-1.88 (m, 1H), 1.86-1.79 (m, 2H), 1.65 (s, 9H);

^{13}C-NMR (100 MHz, $CDCl_3$): δ major isomer: 174.8, 154.4, 137.6, 129.2, 128.7, 128.3, 61.8, 61.5, 59.1, 51.9, 48.8, 30.1, 30.0, 23.5; minor isomer: 175.5, 155.0, 135.9, 129.8, 128.7 (2C), 61.4, 60.7, 59.7, 51.5, 50.8, 30.2, 29.9, 23.8,

HRMS (ESI+): exact mass calculated for $[M+H]^+$ ($C_{18}H_{26}N_5O_2$) requires *m/z* 344.2081, found *m/z* 344.2084.

1-(*tert*-Butyl)-5-(1-(pyrrolidin-1-yl)decyl)-1*H*-tetrazole (42)

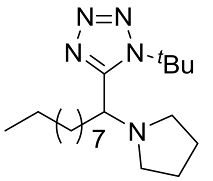

42

According to the general procedure for the synthesis of α-amino tetrazole, 1-(pyrrolidin-1-yl)decan-1-one (67.6 mg, 0.3 mmol) and *tert*-butyl isocyanide (49.9 mg, 0.6 mmol) were used, Title compound was obtained as colorless viscous oil (82.5 mg, 82% yield).

IR (neat) ν_{max}: 2924, 2854, 1459, 1374, 1234, 1135, 1024, 734;

^{1}H-NMR (400 MHz, $CDCl_3$): δ 4.33 (dd, *J* = 8.8, 3.7 Hz, 1H), 2.70-2.66 (m, 2H), 2.56-2.52 (m, 2H), 2.14-2.06 (m, 1H), 1.92-1.84 (m, 1H), 1.72 (s, 9H), 1.68-1.66 (m, 4H), 1.24-1.19 (m, 14H), 0.84 (t, *J* = 7.1 Hz, 3H);

^{13}C-NMR (100 MHz, $CDCl_3$): δ 155.5, 61.6, 56.0, 48.4, 31.9, 30.2, 29.7, 29.6, 29.5, 29.3, 29.0, 26.9, 23.6, 22.7, 14.2;

HRMS (ESI+): exact mass calculated for $[M+H]^+$ ($C_{19}H_{38}N_5$) requires *m/z* 336.3122, found *m/z* 336.3122.

1-(1-(*tert*-Butyl)-1*H*-tetrazol-5-yl)-*N,N*-dimethyl-3-phenylpropan-1-amine (43)

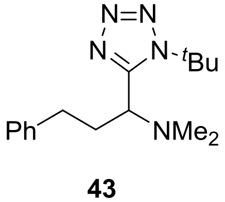

43

According to the general procedure for the synthesis of α-amino tetrazole, *N,N*-dimethyl-3-phenylpropanamide (53.2 mg, 0.3 mmol) and *tert*-butyl isocyanide (49.9 mg, 0.6 mmol) were used, Title compound was obtained as a white solid (68.1 mg, 79% yield).

M.P. = 98-100 ˚C;

IR (neat) ν_{max}: 2937, 2788, 1455, 1234, 1047, 751, 701;

^{1}H-NMR (400 MHz, $CDCl_3$): δ 7.38-7.35 (m, 2H), 7.30-7.23 (m, 3H), 4.13-4.10 (m, 1H), 2.89-2.82 (m, 1H), 2.61-2.52 (m, 2H), 2.35-2.26 (m, 7H), 1.75 (s, 9H);

^{13}C-NMR (100 MHz, $CDCl_3$): δ 154.2, 141.3, 128.5 (2C), 126.2, 61.9, 58.2, 40.3, 32.5, 29.7, 26.3;

HRMS (ESI+): exact mass calculated for $[M+H]^+$ ($C_{16}H_{26}N_5$) requires *m/z* 288.2183, found *m/z* 288.2184.

1-(*tert*-Butyl)-5-(cyclohexyl(pyrrolidin-1-yl)methyl)-1*H*-tetrazole (44)

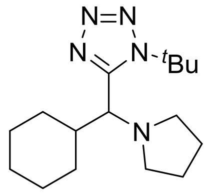

44

According to the general procedure for the synthesis of α-amino tetrazole, cyclohexyl(pyrrolidin-1-yl)methanone (54.4 mg, 0.3 mmol) and *tert*-butyl isocyanide (49.9 mg, 0.6 mmol) were used, Title compound was obtained as a colorless viscous oil (83.1 mg, 95% yield).

IR (neat) ν_{max}: 2928, 2852, 1683, 1450, 1374, 1234, 1027, 734;

^{1}H-NMR (400 MHz, $CDCl_3$): δ 4.23 (d, *J* = 10.3 Hz, 1H), 2.88-2.83 (m, 2H), 2.46-2.41 (m, 2H), 2.23-2.15 (m, 2H), 1.76-1.73 (m, 10H), 1.64-1.54 (m, 6H), 1.28-0.92 (m, 5H), 0.86-0.76 (m, 1H);

^{13}C-NMR (100 MHz, $CDCl_3$): δ 154.2, 61.9, 60.7, 48.3, 41.7, 31.4 (2C), 31.2, 26.6, 26.2 (2C), 23.3;

HRMS (ESI+): exact mass calculated for $[M+H]^+$ ($C_{16}H_{30}N_5$) requires *m/z* 292.2496, found *m/z* 292.2495.

5-((Adamantan-1-yl)(pyrrolidin-1-yl)methyl)-1-(*tert*-butyl)-1*H*-tetrazole (45)

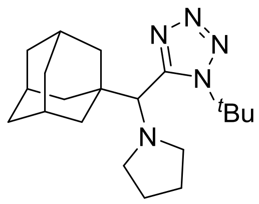

45

According to the general procedure for the synthesis of α-amino tetrazole, (adamantan-1-yl)(pyrrolidin-1-yl)methanone (70 mg, 0.3 mmol) and *tert*-butyl isocyanide (49.9 mg, 0.6 mmol) were used, Title compound was obtained as a viscous oil (38.1 mg, 37% yield).

IR (neat) ν_{max}: 2907, 2850, 1676, 1676, 1453, 1266, 1105, 733;

^{1}H-NMR (400 MHz, $CDCl_3$): δ 4.17 (s, 1H), 2.74-2.69 (m, 4H), 2.00-1.94 (m, 6H), 1.78 (s, 9H), 1.67-1.59 (m, 10H), 1.46-1.42 (m, 3H);

^{13}C-NMR (100 MHz, $CDCl_3$): δ 151.8, 64.8, 62.4, 52.4, 40.1, 37.0, 31.7, 28.7, 24.0;

HRMS (ESI+): exact mass calculated for $[M+H]^+$ ($C_{20}H_{34}N_5$) requires *m/z* 344.2809, found *m/z* 344.2809.

1-((1-(*tert*-Butyl)-1*H*-tetrazol-5-yl)methyl)piperidine (46)

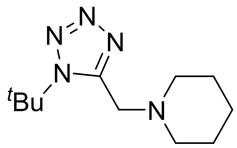

46

According to the general procedure for the synthesis of α-amino tetrazole, 1-formylpiperidine (34.0 mg, 0.3 mmol) and *tert*-butyl isocyanide (49.9 mg, 0.6 mmol) were used, Title compound was obtained as a colorless oil (64.3 mg, 96% yield).

IR (neat) ν_{max}: 2935, 2803, 1470, 1408, 1237, 1107, 799;

^{1}H-NMR (400 MHz, $CDCl_3$): δ 3.75 (s, 2H), 2.33 (br, 4H), 1.73 (s, 9H), 1.52-1.46 (m, 4H), 1.41 (br, 2H);

^{13}C-NMR (100 MHz, $CDCl_3$): δ 151.7, 62.1, 54.2, 52.9, 29.2, 25.7, 24.0;

HRMS (ESI+): exact mass calculated for $[M+H]^+$ ($C_{11}H_{22}N_5$) requires *m/z* 224.1870, found *m/z* 224.1869.

1-Butyl-2-(1-(*tert*-butyl)-1*H*-tetrazol-5-yl)azepane (47)

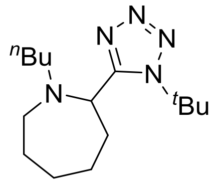

47

According to the general procedure for the synthesis of α-amino tetrazole, 1-butylazepan-2-one (50.9 mg, 0.3 mmol) and *tert*-butyl isocyanide (49.9 mg, 0.6 mmol) were used, Title compound was obtained as a colorless oil (34.4 mg, 41% yield).

IR (neat) ν_{max}: 2926, 2859, 1456, 1373, 1099, 805;

^{1}H-NMR (400 MHz, $CDCl_3$): δ 4.38 (dd, *J* = 10.8, 5.9 Hz, 1H), 3.37-3.30 (m, 1H), 2.74-2.67 (m, 1H), 2.47-2.44 (m, 2H), 2.34-2.24 (m, 1H), 2.01-1.94 (m, 1H), 1.84-1.66 (m, 13H), 1.59-1.52 (m, 2H), 1.30-1.05 (m, 4H), 0.78 (t, *J* = 7.2 Hz, 3H);

^{13}C-NMR (100 MHz, $CDCl_3$): δ 157.2, 61.4, 59.0, 49.7, 49.5, 33.0, 30.7, 30.1, 28.7, 28.2, 25.8, 20.3, 14.0;

HRMS (ESI+): exact mass calculated for $[M+H]^+$ ($C_{15}H_{30}N_5$) requires *m*/*z* 280.2496, found *m*/*z* 280.2496.

1-Pentyl-5-(pyrrolidin-1-yl(*p*-tolyl)methyl)-1*H*-tetrazole (48)

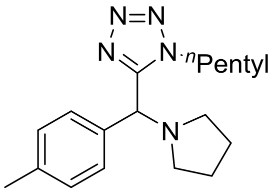

48

According to the general procedure for the synthesis of α-amino tetrazole, pyrrolidin-1-yl(*p*-tolyl)methanone (56.8 mg, 0.3 mmol) and 1-pentyl isocyanide (58.3 mg, 0.6 mmol) were used, Title compound was obtained as a colorless viscous oil (89.3 mg, 95% yield).

IR (neat) ν_{max}: 2956, 2801, 1513, 1458, 1127, 893, 781;

^{1}H-NMR (400 MHz, $CDCl_3$): δ 7.31 (d, *J* = 8.1 Hz, 2H), 7.11 (d, *J* = 8.0 Hz, 2H), 4.85 (s, 1H), 4.33-4.21 (m, 2H), 2.58-2.53 (m, 2H), 2.42-2.37 (m, 2H), 2.27 (s, 3H), 1.78-1.75 (m, 4H), 1.68-1.52 (m, 2H), 1.25-1.13 (m, 4H), 0.81 (t, *J* = 7.1 Hz, 3H);

^{13}C-NMR (100 MHz, $CDCl_3$): δ 155.2, 138.3, 133.9, 129.5, 128.2, 64.2, 52.8, 47.6, 28.9, 28.5, 23.4, 22.1, 21.1, 13.8;

HRMS (ESI+): exact mass calculated for $[M+H]^+$ ($C_{18}H_{28}N_5$) requires *m/z* 314.2339, found *m/z* 314.2337.

1-Benzyl-5-(pyrrolidin-1-yl(*p*-tolyl)methyl)-1*H*-tetrazole (49)

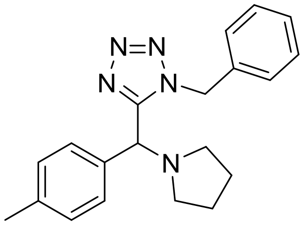

49

According to the general procedure for the synthesis of α-amino tetrazole, pyrrolidin-1-yl(*p*-tolyl)methanone (56.8 mg, 0.3 mmol) and benzyl isocyanide (70.3 mg, 0.6 mmol) were used, Title compound was obtained as a colorless viscous oil (97.0 mg, 97% yield).

IR (neat) ν_{max}: 2960, 2802, 1498, 1111, 893, 722;

^{1}H-NMR (400 MHz, $CDCl_3$): δ 7.25-7.19 (m, 5H), 7.04-7.00 (m, 4H), 5.61 (d, *J* = 15.3 Hz, 1H), 5.41(d, *J* = 15.3 Hz, 1H), 4.74 (s, 1H), 2.44-2.39 (m, 2H), 2.33-2.28 (m, 2H), 2.25 (s, 3H), 1.70-1.68 (m, 4H);

^{13}C-NMR (100 MHz, $CDCl_3$): δ 155.7, 138.3, 133.6, 133.4, 129.4, 128.9, 128.5, 128.4, 127.6, 64.0, 52.5, 51.0, 23.3, 21.1;

HRMS (ESI+): exact mass calculated for $[M+H]^+$ ($C_{20}H_{24}N_5$) requires *m/z* 334.2026, found *m/z* 334.3024.

5-(Pyrrolidin-1-yl(*p*-tolyl)methyl)-1-((tetrahydrofuran-2-yl)methyl)-1*H*-tetrazole (50)

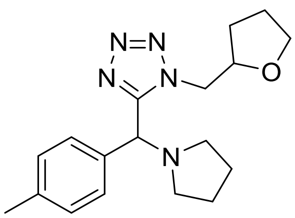

50

According to the general procedure for the synthesis of α-amino tetrazole, pyrrolidin-1-yl(*p*-tolyl)methanone (56.8 mg, 0.3 mmol) and 2-(isocyanomethyl)tetrahydrofuran (66.7 mg, 0.6 mmol) were used, Title compound was obtained as a colorless viscous oil (90.4 mg, 92% yield).

IR (neat) ν_{max}: 2940, 2809, 1490, 1200, 1022, 776;

^{1}H-NMR (400 MHz, $CDCl_3$): δ 7.33-7.29 (m, 2H), 7.12-7.10 (m, 2H), 5.02 (s, 1H, isolated signal for the two isomers), 4.47-4.13 (m, 3H), 3.74-3.61 (m, 2H), 2.56-2.46 (m, 4H), 2.29 (s, 3H), 2.02-1.94 (m, 1H), 1.86-1.57 (m, 7H);

^{13}C-NMR (100 MHz, $CDCl_3$): δ 156.3 (156.1), 138.3, 133.8 (133.7), 129.4, 128.7 (128.6), 76.8 (76.5), 68.5 (68.4), 63.6 (63.3), 52.3 (52.2), 51.1 (50.7), 29.1 (28.7), 25.7 (25.7), 23.5 (23.4), 21.2;

HRMS (ESI+): exact mass calculated for $[M+H]^+$ ($C_{18}H_{26}N_5O$) requires *m/z* 328.2132, found *m/z* 328.2131.

Ethyl 2-(5-(pyrrolidin-1-yl(*p*-tolyl)methyl)-1*H*-tetrazol-1-yl)acetate (51)

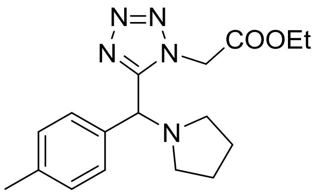

51

According to the general procedure for the synthesis of α-amino tetrazole, pyrrolidin-1-yl(*p*-tolyl)methanone (56.8 mg, 0.3 mmol) and ethyl isocyanoacetate (67.9 mg, 0.6 mmol) were used, Title compound was obtained as a white solid (59.3 mg, 60% yield).

M.P. = 118-120 ˚C;

IR (neat) ν_{max}: 2940, 2809, 1750, 1450, 1210, 1022, 781;

^{1}H-NMR (400 MHz, $CDCl_3$): δ 7.29 (d, *J* = 8.1 Hz, 2H), 7.13 (d, *J* = 7.9 Hz, 2H), 5.29 (s, 2H), 4.97 (s, 1H), 4.10-4.00 (m, 2H), 2.59-2.55 (m, 2H), 2.41-2.37 (m, 2H), 2.29 (s, 3H), 1.78-1.75 (m, 4H), 1.15 (t, *J* = 7.1 Hz, 3H);

^{13}C-NMR (100 MHz, $CDCl_3$): δ 165.4, 155.9, 138.4, 132.9, 129.5, 128.2, 64.1, 62.5, 52.5, 48.5, 23.5, 21.1, 13.9;

HRMS (ESI+): exact mass calculated for $[M+H]^+$ ($C_{17}H_{24}N_5O_2$) requires *m/z* 330.1925, found *m/z* 330.1922.

1-Cyclohexyl-5-(pyrrolidin-1-yl(*p*-tolyl)methyl)-1*H*-tetrazole (52)

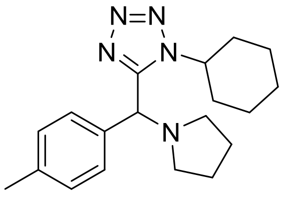

52

According to the general procedure for the synthesis of α-amino tetrazole, pyrrolidin-1-yl(*p*-tolyl)methanone (56.8 mg, 0.3 mmol) and cyclohexyl isocyanide (65.5 mg, 0.6 mmol) were used, Title compound was obtained as a colorless viscous oil (92.8 mg, 95% yield).

IR (neat) ν_{max}: 2935, 2801, 1450, 1266, 1126, 895, 782;

^{1}H-NMR (400 MHz, $CDCl_3$): δ 7.32 (d, *J* = 8.1 Hz, 2H), 7.11 (d, *J* = 7.8 Hz, 2H), 4.89 (s, 1H), 4.62-4.55 (m, 1H), 2.61-2.57 (m, 2H), 2.42-2.37 (m, 2H), 2.27 (s, 3H), 1.94-1.61 (m, 10H), 1.46-1.42 (m, 1H), 1.34-1.16 (m, 3H);

^{13}C-NMR (100 MHz, $CDCl_3$): δ 154.6, 138.2, 134.3, 129.4, 128.0, 64.4, 57.9, 53.0, 32.9, 32.6, 25.5, 25.3, 24.9, 23.5, 21.1;

HRMS (ESI+): exact mass calculated for $[M+H]^+$ ($C_{19}H_{28}N_5$) requires *m/z* 326.2339, found *m/z* 326.2338.

5-(Pyrrolidin-1-yl(*p*-tolyl)methyl)-1-(1,2,3,6-tetrahydro-[1,1'-biphenyl]-4-yl)-1*H*-tetrazole (53)

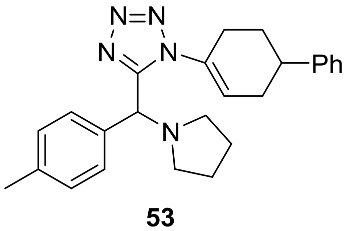

53

According to the general procedure for the synthesis of α-amino tetrazole, pyrrolidin-1-yl(*p*-tolyl)methanone (56.8 mg, 0.3 mmol) and (4-isocyano-3-cyclohexen-1-yl)benzene (110 mg, 0.6 mmol) were used, Title compound was obtained as a colorless viscous oil (115 mg, 96% yield).

IR (neat) ν_{max}: 2924, 1513, 1434, 1107, 733, 699;

^{1}H-NMR (400 MHz, $CDCl_3$): δ 7.32-7.20 (m, 7H), 7.11 (t, *J* = 7.5 Hz, 2H), 5.82-5.79 (m, 1H), 4.78 (s, 1H, isolated signal for the two isomers), 2.91-2.77 (m, 1H), 2.51-2.46 (m, 5H), 2.39-2.20 (m, 6H), 2.03-1.84 (m, 2H), 1.74 (brm, 4H);

^{13}C-NMR (100 MHz, $CDCl_3$): δ 155.3, 144.8, 138.3, 134.1 (134.0), 132.7 (132.6), 129.3, 128.9, 128.8, 128.7, 126.8 (126.7), 63.6 (63.5), 52.4, 38.7 (38.5), 32.3 (32.2), 29.3, 29.1 (29.0), 23.4, 21.2;

HRMS (ESI+): exact mass calculated for $[M+H]^+$ ($C_{25}H_{30}N_5$) requires *m/z* 400.2496, found *m/z* 400.2497.

1-(Naphthalen-2-yl)-5-(pyrrolidin-1-yl(*p*-tolyl)methyl)-1*H*-tetrazole (54)

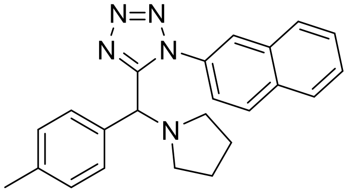

54

According to the general procedure for the synthesis of α-amino tetrazole, pyrrolidin-1-yl(*p*-tolyl)methanone (56.8 mg, 0.3 mmol) and 2-naphthyl isocyanide (91.9 mg, 0.6 mmol) were used, Title compound was obtained as a yellowish solid (101 mg, 91% yield).

M.P. = 126-128 ˚C;

IR (neat) ν_{max}: 2963, 2796, 1512, 1435, 1103, 814, 733;

^{1}H-NMR (400 MHz, $CDCl_3$): δ 7.98-7.93 (m, 2H), 7.82-7.80 (m, 1H), 7.68 (d, *J* = 2.1 Hz, 1H), 7.64-7.58 (m, 2H), 7.31 (dd, *J* = 8.6, 2.2 Hz, 1H), 7.24 (d, *J* = 8.1 Hz, 2H), 7.08 (d, *J* = 7.8 Hz, 2H), 4.72 (s, 1H), 2.49-2.46 (m, 4H), 2.30 (s, 3H), 1.75-1.72 (m, 4H);

^{13}C-NMR (100 MHz, $CDCl_3$): δ 156.2, 138.4, 133.7, 133.6, 132.7, 131.0, 129.9, 129.3, 128.9, 128.4, 128.1 (2C), 127.7, 125.4, 122.9, 63.3, 52.2, 23.3, 21.1;

HRMS (ESI+): exact mass calculated for $[M+H]^+$ ($C_{23}H_{24}N_5$) requires *m/z* 370.2026, found *m/z* 370.2029.

1-(2,6-Dimethylphenyl)-5-(pyrrolidin-1-yl(*p*-tolyl)methyl)-1*H*-tetrazole (55)

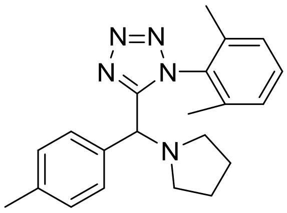

55

According to the general procedure for the synthesis of α-amino tetrazole, pyrrolidin-1-yl(*p*-tolyl)methanone (56.8 mg, 0.3 mmol) and 2,6-dimethylphenyl isocyanide (78.7 mg, 0.6 mmol) were used, Title compound was obtained as a colorless viscous oil (94.9 mg, 91% yield).

IR (neat) ν_{max}: 2965, 2793, 1474, 1103, 778, 702;

^{1}H-NMR (400 MHz, $CDCl_3$): δ 7.33 (t, *J* = 7.6 Hz, 1H), 7.19 (d, *J* = 7.5 Hz, 1H), 7.00-6.92 (m, 5H), 4.10 (s, 1H), 2.59-2.54 (m, 2H), 2.35-2.30 (m, 2H), 2.23 (s, 3H), 1.93 (s, 3H), 1.78-1.71 (m, 4H), 1.02 (s, 3H);

^{13}C-NMR (100 MHz, $CDCl_3$): δ 156.7, 138.4, 137.1, 134.9, 133.7, 131.7, 130.9, 129.2, 128.8, 128.7, 128.6, 64.8, 53.0, 23.3, 21.1, 17.5, 16.4;

HRMS (ESI+): exact mass calculated for $[M+H]^+$ ($C_{21}H_{26}N_5$) requires *m/z* 348.2183, found *m/z* 348.2182.

1-((4-Chlorophenyl)(1-(2,6-dimethylphenyl)-1*H*-tetrazol-5-yl)methyl)-4-cinnamylpiperazine (56)

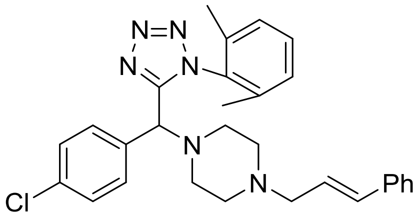

56

According to the general procedure for the synthesis of α-amino tetrazole, (4-chlorophenyl)(4-cinnamylpiperazin-1-yl)methanone (102 mg, 0.3 mmol) and 2,6-dimethylphenyl isocyanide (78.7 mg, 0.6 mmol) were used, Title compound was obtained as a white solid (112 mg, 75% yield).

M.P. = 108-110 ˚C;

IR (neat) ν_{max}: 2813, 1490, 1266, 1135, 1005, 734;

^{1}H-NMR (400 MHz, $CDCl_3$): δ 7.44-7.24 (m, 9H), 7.20-7.17 (m, 2H), 7.13 (d, *J* = 7.4 Hz, 1H), 6.54 (d, *J* = 15.9 Hz, 1H), 6.27 (dt, *J* = 15.9, 6.8 Hz, 1H), 4.27 (s, 1H), 3.17 (d, *J* = 6.8 Hz, 2H), 2.53 (br, 8H), 2.05 (s, 3H), 1.25 (s, 3H);

^{13}C-NMR (100 MHz, $CDCl_3$): δ 155.4, 136.9, 136.6, 135.4, 134.9, 133.4, 133.3, 131.6, 131.2, 130.8, 129.0 (2C), 128.9, 128.7, 127.7, 126.4, 126.3, 64.7, 60.8, 53.0, 51.3, 17.7, 16.9;

HRMS (ESI+): exact mass calculated for $[M+H]^+$ ($C_{29}H_{32}N_6Cl$) requires *m/z* 499.2372, found *m/z* 499.2369.

2-(4-(Benzo[d][1,3]dioxol-5-ylmethyl)piperazin-1-yl)-*N*-(*tert*-butyl)-3-(4-chlorophenoxy)propanamide (57)

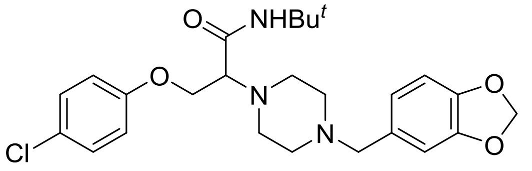

From Fipexide
57

According to the general procedure for the synthesis of α-amino amide, Fipexide (117 mg, 0.3 mmol) and *tert*-butyl isocyanide (49.9 mg, 0.6 mmol) were used, Title compound was obtained as a white solid (78.2 mg, 55% yield).

M.P. = 68-72 ˚C;

IR (neat) ν_{max}: 3330, 2813, 1671, 1489, 1242, 1037, 823;

^{1}H-NMR (400 MHz, $CDCl_3$): δ 7.45 (br, 1H), 7.21-7.19 (m, 2H), 6.84-6.81 (m, 3H), 6.72 (br, 2H), 5.93-5.91 (m, 2H), 4.49 (dd, *J* = 10.5, 3.2 Hz, 1H), 4.22 (dd, *J* = 10.5, 3.1 Hz, 1H), 3.40 (s, 2H), 3.35-3.33 (m, 1H), 2.79-2.78 (m, 2H), 2.67-2.64 (m, 2H), 2.44 (br, 4H), 1.35 (s, 9H);

^{13}C-NMR (100 MHz, $CDCl_3$): δ 169.4, 157.1, 147.7, 146.7, 132.0, 129.4, 125.9, 122.2, 115.9, 109.5, 107.9, 101.0, 67.7, 65.8, 62.8, 53.9, 50.6, 28.8;

HRMS (ESI+): exact mass calculated for $[M+H]^+$ ($C_{25}H_{33}N_3O_4Cl$) requires *m/z* 474.2154, found *m/z* 474.2147.

(10*Z*,13*Z*)-*N*-(*tert*-Butyl)-2-(pyrrolidin-1-yl)nonadeca-10,13-dienamide (58)

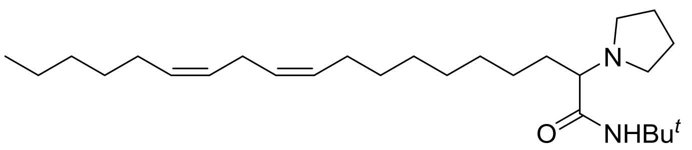

From Linoleic carboxamide
58

According to the general procedure for the synthesis of α-amino amide, Linoleic carboxamide (100 mg, 0.3 mmol) and *tert*-butyl isocyanide (49.9 mg, 0.6 mmol) were used, Title compound was obtained as a colorless viscous oil (107 mg, 85% yield).

IR (neat) ν_{max}: 3344, 2925, 2855, 1681, 1509, 1228, 722;

^{1}H-NMR (400 MHz, $CDCl_3$): δ 6.54 (br, 1H), 5.39-5.27 (m, 4H), 2.76 (t, *J* = 6.4 Hz, 2H), 2.57-2.51 (m, 5H), 2.05-1.99 (m, 4H), 1.75-1.71 (m, 5H), 1.54-1.47 (m, 1H), 1.33 (s, 9H), 1.26 (br, 16H), 0.88 (t, *J* = 7.0 Hz, 3H);

^{13}C-NMR (100 MHz, $CDCl_3$): δ 172.8, 130.3, 130.2, 128.1, 128.0, 70.6, 51.8, 50.5, 32.5, 31.6, 29.9, 29.7, 29.4, 29.3, 28.9, 27.3 (2C), 25.7, 25.4, 23.4, 22.7, 14.2;

HRMS (ESI+): exact mass calculated for $[M+H]^+$ ($C_{27}H_{51}N_2O$) requires *m/z* 419.3996, found *m/z* 419.3983.

(5*R*)-*N*-(*tert*-Butyl)-5-((3*R*,5*R*,8*R*,9*S*,10*S*,13*R*,14*S*)-3-hydroxy-10,13-dimethylhexadecahydro-1*H*-cyclopenta[a]phenanthren-17-yl)-2-(pyrrolidin-1-yl)hexanamide (59)

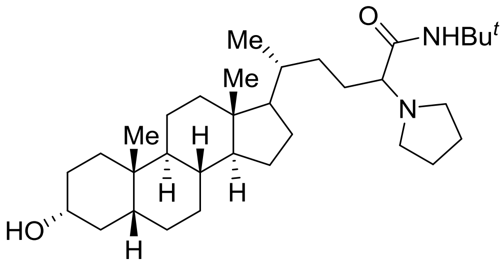

From Lithocholic carboxamide
59

According to the general procedure for the synthesis of α-amino amide, Lithocholic carboxamide (129 mg, 0.3 mmol) and *tert*-butyl isocyanide (49.9 mg, 0.6 mmol) were used, Title compound was obtained as a white solid (110 mg, 71% yield).

M.P. = 142 ˚C;

IR (neat) ν_{max}: 3325, 2934, 1669, 1518, 1265, 909, 732;

^{1}H-NMR (400 MHz, $CDCl_3$): δ 6.61 (br, 1H), 3.63-3.55 (m, 1H), 2.57 (br, 4H), 1.94-1.90 (m, 1H), 1.81-0.94 (m, 42H), 0.89 (s, 6H), 0.60 (s, 3H);

^{13}C-NMR (100 MHz, $CDCl_3$): δ 172.4, 71.9, 70.9 (2C), 56.6 (2C), 56.2, 55.9, 51.8 (2C), 50.6, 42.8 (2C), 42.2, 40.5, 40.3, 40.2, 36.6, 36.0 (2C), 35.7, 35.5, 34.7, 31.3, 31.2, 30.7, 28.9, 28.4, 28.3, 27.3, 26.5, 24.3, 23.5, 23.4, 20.9, 18.8, 18.7, 12.1 (2C);

HRMS (ESI+): exact mass calculated for $[M+H]^+$ ($C_{33}H_{59}N_2O_2$) requires *m/z* 515.4571, found *m/z* 515.4572.

***N*-(*tert*-Butyl)-2-((3-(10,11-dihydro-5*H*-dibenzo[a,d][7]annulen-5-ylidene)propyl)(methyl)amino)-4-phenylbutanamide (60)**

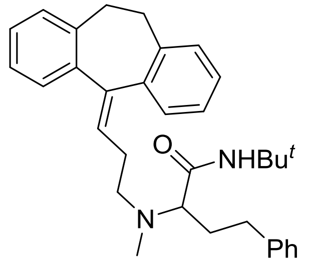

From Nortriptyline derivative
60

According to the general procedure for the synthesis of α-amino amide, *N*-(3-(10,11-dihydro-5*H*-dibenzo[a,d][7]annulen-5-ylidene)propyl)-*N*-methyl-3-phenylpropanamide (119 mg, 0.3 mmol) and *tert*-butyl isocyanide (49.9 mg, 0.6 mmol) were used, Title compound was obtained as a colorless viscous oil (128 mg, 89% yield).

IR (neat) ν_{max}: 3340, 3025, 2925, 1673, 1506, 1453, 1227, 734;

^{1}H-NMR (400 MHz, $CDCl_3$): δ 7.31-7.27 (m, 3H), 7.23-7.13 (m, 9H), 7.06-7.04 (m, 1H), 6.95 (br, 1H), 5.86 (t, *J* = 7.2 Hz, 1H), 3.42-3.31 (m, 2H), 3.02-2.82 (m, 4H), 2.65-2.57 (m, 1H), 2.48 (br, 2H), 2.29 (br, 2H), 2.12 (s, 3H), 1.96-1.84 (m, 2H), 1.25 (s, 9H);

^{13}C-NMR (100 MHz, $CDCl_3$): δ 172.6, 144.1, 142.3, 141.1, 140.0, 139.4, 137.0, 130.1, 129.1, 128.6, 128.4, 128.3, 128.2, 127.6, 127.2, 126.1, 125.9, 125.8, 68.0 (2C), 54.4 (2C), 50.3, 37.9 (2C), 34.0 (2C), 33.8, 32.1, 28.7, 28.3 (2C), 28.1;

HRMS (ESI+): exact mass calculated for $[M+H]^+$ ($C_{33}H_{41}N_2O$) requires *m/z* 481.3213, found *m/z* 481.3209.

1-(1-(*tert*-Butyl)-1*H*-tetrazol-5-yl)-*N,N*-diethyl-2-(naphthalen-1-yloxy)propan-1-amine (61)

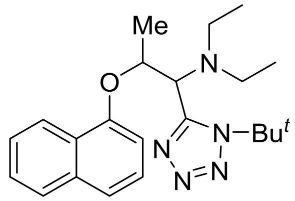

From Napropamid
61

According to the general procedure for the synthesis of α-amino tetrazole, Napropamid (81.4 mg, 0.3 mmol) and *tert*-butyl isocyanide (49.9 mg, 0.6 mmol) were used, Title compound was obtained as a colorless viscous oil (74.4 mg, 65% yield). (dr was estimated by ^{1}H analyse of the crude postreaction mixture.)

IR (neat) ν_{max}: 2974, 1578, 1397, 1265, 1096, 771, 730;

^{1}H-NMR (400 MHz, $CDCl_3$):

The integral ratios are given as observed.

δ 8.26-8.23 (m, 0.2H), 7.97-7.94 (m, 1H), 7.83-7.81 (m, 0.2H), 7.75-7.73 (m, 1H), 7.51-7.46 (m, 0.6H), 7.45-7.35 (m, 3.2H), 7.32-7.26 (m, 1H), 7.03 (d, *J* = 7.3 Hz, 0.2H), 6.89 (d, *J* = 7.5 Hz, 1H), 5.79-5.73 (m, 0.2H), 5.40-5.33 (m, 1H), 4.72 (d, *J* = 9.3 Hz, 1.2H), 3.25-3.16 (m, 0.4H), 3.01-2.92 (m, 2H), 2.89-2.84 (m, 0.4H), 2.82-2.74 (m, 2H), 1.87 (s, 1.8H), 1.82 (s, 9H), 1.54 (d, *J* = 6.1 Hz, 3H), 1.21 (d, *J* = 6.0 Hz, 0.6H), 1.06 (t, *J* = 7.1 Hz, 6H), 0.93 (t, *J* = 7.1 Hz, 1.2H);

^{13}C-NMR (100 MHz, $CDCl_3$):

major isomer: δ 154.4, 153.0, 134.7, 127.7, 126.2, 126.1, 125.1, 121.8, 121.3, 109.0, 78.0, 62.2, 62.1, 45.4, 31.0, 18.8, 15.0;

Minor isomer: δ 153.5, 152.4, 135.0, 127.8, 126.8, 126.4, 125.2, 122.3, 120.5, 105.7, 73.0, 64.4, 62.1, 46.4, 30.1, 17.8, 15.6;

HRMS (ESI+): exact mass calculated for $[M+H]^+$ ($C_{22}H_{32}N_5O$) requires *m/z* 382.2601, found *m/z* 382.2600.

1-((1-(*tert*-Butyl)-1H-tetrazol-5-yl)(2,3-dihydrobenzo[b][1,4]dioxin-6-yl)methyl)piperidine (62)

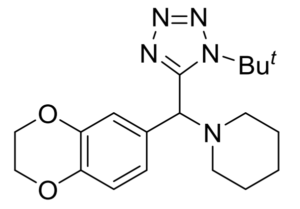

From CX-546
62

According to the general procedure for the synthesis of α-amino tetrazole, CX-546 (74.2 mg, 0.3 mmol) and *tert*-butyl isocyanide (49.9 mg, 0.6 mmol) were used, Title compound was obtained as a white solid (98.7 mg, 92% yield).

M.P. = 162-164 ˚C;

IR (neat) ν_{max}: 2934, 2852, 1590, 1506, 1285, 1102, 887;

^{1}H-NMR (400 MHz, $CDCl_3$): δ 6.93 (d, *J* = 2.1 Hz, 1H), 6.83 (dd, *J* = 8.3, 2.2 Hz, 1H), 6.76 (d, *J* = 8.3 Hz, 1H), 5.09 (s, 1H), 4.19 (s, 4H), 2.62-2.57 (m, 2H), 2.34-2.29 (m, 2H), 1.66 (s, 9H), 1.52-1.46 (m, 4H), 1.37-1.31 (m, 2H);

^{13}C-NMR (100 MHz, $CDCl_3$): δ 154.6, 143.5, 143.3, 128.5, 122.9, 118.8, 116.9, 65.1, 64.4, 64.3, 61.4, 51.5, 30.2, 26.2, 24.3;

HRMS (ESI+): exact mass calculated for $[M+H]^+$ ($C_{19}H_{28}N_5O_2$) requires *m/z* 358.2238, found *m/z* 358.2238.

Methyl *N*-(2-((tert-butoxycarbonyl)amino)-1-(1-(tert-butyl)-1*H*-tetrazol-5-yl)ethyl)-*N*-methylglycinate (63)

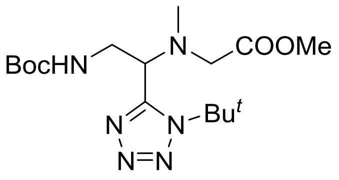

From Boc-Gly-Sar-OMe
63

According to the general procedure for the synthesis of α-amino tetrazole, Boc-Gly-Sar-OMe (78.1 mg, 0.3 mmol) and *tert*-butyl isocyanide (49.9 mg, 0.6 mmol) were used, Title compound was obtained as a colorless viscous oil (37.8 mg, 34% yield).

IR (neat) ν_{max}: 3390, 2952, 1741, 1698, 1366, 1165, 1050, 774;

^{1}H-NMR (400 MHz, $CDCl_3$): δ 5.07 (br, 1H), 4.52-4.48 (m, 1H), 3.81-3.68 (m, 2H), 3.65 (s, 3H), 3.53-3.43 (m, 2H), 2.47 (s, 3H), 1.76 (s, 9H), 1.38 (s, 9H);

^{13}C-NMR (100 MHz, $CDCl_3$): δ 171.3, 156.1, 153.2, 79.8, 62.5, 58.4, 53.7, 51.9, 39.2, 38.1, 29.7, 28.4;

HRMS (ESI+): exact mass calculated for $[M+H]^+$ ($C_{16}H_{31}N_6O_4$) requires *m/z* 371.2401, found *m/z* 371.2400.

Ethyl (1-(1-(*tert*-butyl)-1*H*-tetrazol-5-yl)-2-phenylethyl)-L-prolylglycinate (64)

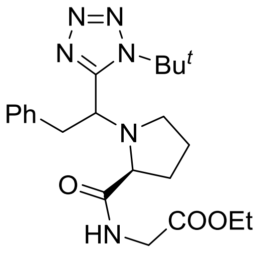

From Noopept
64 (dr ~1:1)

According to the general procedure for the synthesis of α-amino tetrazole, Noopept (95.5 mg, 0.3 mmol) and *tert*-butyl isocyanide (49.9 mg, 0.6 mmol) were used, Title compound was obtained as a colorless viscous oil (57.9 mg, 45% yield). (dr was estimated by ^{1}H analyse of the crude postreaction mixture.)

IR (neat) ν_{max}: 3350, 2940, 1746, 1669, 1510, 1198, 1026, 734, 702;

^{1}H-NMR (400 MHz, $CDCl_3$):

The integral ratios are given as observed.

δ 7.63 (t, *J* = 5.6 Hz, 0.83H), 7.25-7.16 (m, 5.18H), 7.13-7.16 (m, 2.52H), 6.95 (t, *J* = 6.1 Hz, 1H), 6.88-6.85 (m, 1.66H), 4.85 (dd, *J* = 8.7, 2.0 Hz, 1H), 4.77 (dd, *J* = 10.5, 4.6 Hz, 0.83H), 4.42-4.39 (m, 0.83H), 4.20-4.13 (m, 3.6H), 4.06 (dd, *J* = 18.3, 6.1 Hz, 0.83H), 3.95 (dd, *J* = 17.9, 7.1 Hz, 1H), 3.88 (dd, *J* = 18.3, 5.4 Hz, 0.83H), 3.75-3.69 (m, 1H), 3.46-3.38 (m, 1.83H), 3.33-3.27 (m, 2.83H), 3.24-3.15 (m, 1.83H), 3.10 (dd, *J* = 9.8, 4.4 Hz, 1H), 2.64-2.58 (m, 0.83H), 2.03-1.95 (m, 3.3H), 1.79-1.72 (m, 3.8H), 1.68-1.63 (m, 0.83H), 1.58 (s, 9H), 1.30 (s, 7.4H), 1.29 (t, *J* = 7.1 Hz, 3H), 1.25 (dd, *J* = 7.1 Hz, 2.6H);

^{13}C-NMR (100 MHz, $CDCl_3$): δ 175.6, 174.8, 169.9, 169.7, 155.0, 154.7, 137.5, 136.8, 129.5, 129.4, 128.8, 128.6, 127.1, 127.0, 63.9, 62.9, 61.9, 61.4, 61.3, 58.8, 56.7, 51.8, 48.1, 41.0, 40.5, 40.1, 39.5, 31.7, 31.4, 30.3, 30.2, 24.6, 24.3, 14.3, 14.2;

HRMS (ESI+): exact mass calculated for $[M+H]^+$ ($C_{22}H_{33}N_6O_3$) requires *m/z* 429.2608, found *m/z* 429.2607.

2-([1,1'-Biphenyl]-4-yl)-*N*-benzyl-2-(pyrrolidin-1-yl)acetamide (66)

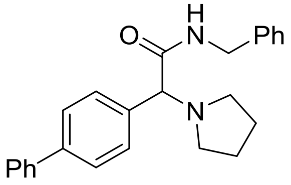

66

According to the general procedure for the synthesis of α-amino amide, [1,1'-biphenyl]-4-yl(pyrrolidin-1-yl)methanone (75.4 mg, 0.3 mmol) and benzyl isocyanide (70.3 mg, 0.6 mmol) were used, Title compound was obtained as a white solid (96.8 mg, 87% yield).

M.P. = 144 ˚C;

IR (neat) ν_{max}: 3329, 2813, 1684, 1490, 1090, 734;

^{1}H-NMR (400 MHz, $CDCl_3$): δ 7.58-7.53 (m, 4H), 7.47-7.41 (m, 4H), 7.37-7.21 (m, 6H), 4.53-4.41 (m, 2H), 3.90 (s, 1H), 2.57-2.53 (m, 2H), 2.51-2.47 (m, 2H), 1.76-1.72 (m, 4H);

^{13}C-NMR (100 MHz, $CDCl_3$): δ 171.9, 141.0, 140.8, 138.5, 137.3, 128.8, 128.7, 128.6, 127.8, 127.5, 127.4, 127.3, 127.1, 75.4, 52.8, 43.2, 23.5;

HRMS (ESI+): exact mass calculated for $[M+H]^+$ ($C_{25}H_{27}N_2O$) requires *m/z* 371.2118, found *m/z* 371.2119.

2-([1,1'-Biphenyl]-4-yl)-*N*-benzylacetamide (67)[17]

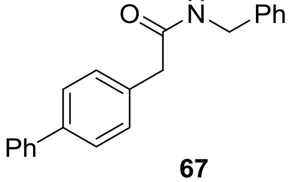

67

2-([1,1'-Biphenyl]-4-yl)-N-benzyl-2-(pyrrolidin-1-yl)acetamide (**66**) (20 mg, 0.054 mmol) and Pd/C (10 mg, 10 wt.%) was charged into a 25 mL flask with a septum. The flask was then vacuumed and refilled with a H_2 balloon, before methanol (2 mL) were injected. The result mixture was stirred for 12 h at room temperature. The mixture was filtrated with celite and purified by a flash column chromatography after removing the solvent under vacuum. Title compound was obtained as a white solid (15.6 mg, 95% yield).

^{1}H-NMR (400 MHz, DMSO-d^6): δ 8.61 (t, *J* = 5.8 Hz, 1H), 7.67-7.60 (m, 4H), 7.48-7.44 (m, 2H), 7.39-7.30 (m, 5H), 7.26-7.22 (m, 3H), 4.30 (d, *J* = 5.9 Hz, 2H), 3.53 (s, 2H);

^{13}C-NMR (100 MHz, DMSO-d^6): δ 170.5, 140.5, 139.9, 138.8, 136.1, 130.1, 129.4, 128.7, 127.7 (2C), 127.2, 127.0 (2C), 42.7, 42.4.

Methyl 2-(5-([1,1'-biphenyl]-4-yl(pyrrolidin-1-yl)methyl)-1*H*-tetrazol-1-yl)acetate (68)

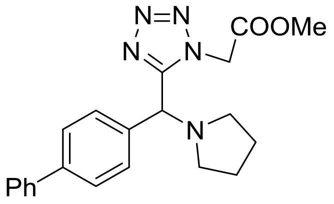

68

According to the general procedure for the synthesis of α-amino tetrazole, [1,1'-biphenyl]-4-yl(pyrrolidin-1-yl)methanone (75.4 mg, 0.3 mmol) and methyl isocyanoacetate (59.5 mg, 0.6 mmol) were used, Title compound was obtained as a white solid (80.5 mg, 71% yield).

M.P. = 156-158 ˚C;

IR (neat) ν_{max}: 2953, 2811, 1755, 1439, 1221, 759;

^{1}H-NMR (400 MHz, $CDCl_3$): δ 7.57-7.53 (m, 4H), 7.49 (d, *J* = 8.2 Hz, 2H), 7.44-7.40 (m, 2H), 7.36-7.32 (m, 1H), 5.44 (q, *J* = 18.0 Hz, 2H), 5.09 (s, 1H), 3.56 (s, 3H), 2.65-2.62 (m, 2H), 2.45-2.42 (m, 2H), 1.82-1.79 (m, 4H);

^{13}C-NMR (100 MHz, $CDCl_3$): δ 165.8, 155.8, 141.5, 140.3, 134.6, 129.0, 128.8, 127.8, 127.5, 127.1, 63.9, 53.1, 52.6, 48.4, 23.5;

HRMS (ESI+): exact mass calculated for $[M+H]^+$ ($C_{21}H_{24}N_5O_2$) requires *m/z* 378.1925, found *m/z* 378.1926.

Methyl 2-(5-([1,1'-biphenyl]-4-ylmethyl)-1*H*-tetrazol-1-yl)acetate (69)[18]

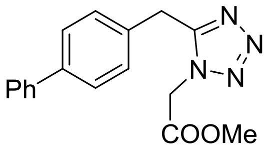

69

Methyl 2-(5-([1,1'-biphenyl]-4-yl(pyrrolidin-1-yl)methyl)-1*H*-tetrazol-1-yl)acetate (**68**) (20 mg, 0.053 mmol) and Pd/C (10 mg, 10 wt.%) was charged into a 25 mL flask with a septum. The flask was then vacuumed and refilled with a H_2 balloon, before methanol (2 mL) were injected. The result mixture was stirred for 12 h at room temperature. The mixture was filtrated with celite and purified by a flash column chromatography after removing the solvent under vacuum. Title compound was obtained as a colorless film (16.0 mg, 97% yield).

^{1}H-NMR (400 MHz, $CDCl_3$): δ 7.57-7.55 (m, 4H), 7.46-7.42 (m, 2H), 7.38-7.34 (m, 1H), 7.25 (d, *J* = 8.1 Hz, 2H), 4.93 (s, 2H), 4.36 (s, 2H), 3.69 (s, 3H);

^{13}C-NMR (100 MHz, $CDCl_3$): δ 165.6, 154.5, 141.1, 140.3, 132.1, 129.2, 129.1, 128.0, 127.8, 127.1, 53.4, 48.1, 29.5.

Supplementary References

1. Gu, J. et al. A two-step continuous flow synthesis of amides from alcohol using a metal-free catalyst. *RSC Adv.* **5**, 95014–95019 (2015).

2. Liu, Z., Zhang, J., Chen, S., Shi, E., Xu, Y. & Wan, X. Cross coupling of acyl and aminyl radicals: direct synthesis of amides catalyzed by Bu_4NI with TBHP as an oxidant. *Angew. Chem. Int. Ed.* **51**, 3231–3235 (2012).

3. Huang, H., Yuan, G., Li, X. & Jiang, H. Electrochemical synthesis of amides: direct transformation of methyl ketones with formamides. *Tetrahedron Lett.* **54**, 7156–7159 (2013).

4. Zhu, M., Fujita, K. & Yamaguchi, R. Aerobic oxidative amidation of aromatic and cinnamic aldehydes with secondary amines by CuI/2-pyridonate catalytic system. *J. Org. Chem.* **77**, 9102–9109 (2012).

5. Venkov, A. P. & Lukanov, L. K. New modification of the intramolecular α-amidoalkylation for the synthesis of 2-acyl-1,2,3,4-tetrahydroisoquinolines. *Synthesis* 59–61 (1989).

6. Ghosh, S. C., Ngiam, J. S. Y., Seayad, A. M., Tuan, D. T., Johannes, C. W. & Chen, A. Tandem oxidative amidation of benzyl alcohols with amine hydrochloride salts catalysed by iron nitrate. *Tetrahedron Lett.* **54**, 4922–4925 (2013).

7. Yao, W., Ma, X., Guo, L., Jia, X., Hua, A. & Huang, Z. A highly efficient catalytic α-alkylation of unactivated amides using primary alcohols. *Tetrahedron Lett.* **57**, 2919–2921 (2016).

8. Gao, L., Kojima, K. & Nagashima, H. Transition metal nanoparticles stabilized by ammonium salts of hyperbranched polystyrene: effect of metals on catalysis of the biphasic hydrogenation of alkenes and arenes. *Tetrahedron* **71**, 6414–6423 (2015).

9. Li, J., Lear, M. J. & Hayashi, Y. Sterically demanding oxidative amidation of α - substituted malononitriles with amines using O_2. *Angew. Chem. Int. Ed.* **55**, 9060–9064 (2016).

10. Hanada, S., Tsutsumi, E., Motoyama, Y. & Nagashima, H. Practical access to amines by platinum-catalyzed reduction of carboxamides with hydrosilanes: synergy of dual Si−H groups leads to high efficiency and selectivity. *J. Am. Chem. Soc.* **131**, 15032–15040 (2009).

11. Moeller, K. D., Wang, P. W., Tarazi, S., Marzabadi, M. R. & Wong, P. L. Anodic amide oxidations in the presence of electron-rich phenyl rings: evidence for an intramolecular electron-transfer mechanism. *J. Org. Chem.* **56**, 1058–1067 (1991).

12. Ekoue-Kovi, K. & Wolf, C. Metal-free one-pot oxidative amination of aldehydes to amides. *Org. Lett.* **9**, 3429–3432 (2007).

13. Bechi, B. et al. Catalytic bio–chemo and bio–bio tandem oxidation reactions for amide and carboxylic acid synthesis. *Green Chem.* **16**, 4524–4529 (2014).

14. López-Cobeñas, A. et al. Microwave-assisted synthesis of 2,5-piperazinediones under solvent-free conditions. *Synthesis* 3412–3422 (2005).

15. Hua, X., Masson-Makdissi, J., Sullivan, R. J. & Newman, S. G. Inherent vs apparent chemoselectivity in the Kumada–Corriu cross-coupling reaction. *Org. Lett.* **18**, 5312–5315 (2016).

16. Dawidowski, M., Lewandowski, W. & Turło, J. Synthesis of new perhydropyrrolo[1,2-a]pyrazine derivatives and their evaluation in animal models of epilepsy. *Molecules* **19**, 15955–15981 (2014).

17. Laraia, L., Stokes, J., Emery, A., McKenzie, G. J., Venkitaraman, A. R. & Spring, D. R. High content screening of diverse compound libraries identifies potent modulators of tubulin dynamics. *ACS Med. Chem. Lett.* **5**, 598–603 (2014).

18. Ortar, G. et al. New tetrazole-based selective anandamide uptake inhibitors. *Bioorg. Med. Chem. Lett.* **18**, 2820–2824 (2008).
